# Second-Generation of Deuterium-Substituted Glutamate Uptake Enhancers Exhibit Superior Drug-Like Properties in Preclinical Evaluation

**DOI:** 10.1021/acscentsci.6c00080

**Published:** 2026-05-29

**Authors:** Michał Abram, Marcin Jakubiec, Małgorzata Szafarz, Anna Rapacz, Magdalena Kolasa, Agata Faron-Górecka, Szczepan Mogilski, Kaliana Veros, Simran K. Gill, Angela Di Iacovo, Katarzyna Socała, Gniewomir Latacz, Joanna Karnafał, Krzysztof Pociecha, Justyna Kalinowska-Tłuścik, Melissa Barker-Haliski, Elżbieta Wyska, Andréia C. K. Fontana, Piotr Wlaź, Rafał M. Kamiński, Cristina Roseti, Elena Bossi, Karen S. Wilcox, Krzysztof Kamiński

**Affiliations:** † Department of Medicinal Chemistry, Faculty of Pharmacy, Jagiellonian University Medical College, Medyczna 9, 30-688 Krakow, Poland; ‡ Department of Pharmacokinetics and Physical Pharmacy, Faculty of Pharmacy, Jagiellonian University Medical College, Medyczna 9, 30-688 Krakow, Poland; § Department of Pharmacodynamics, Faculty of Pharmacy,Jagiellonian University Medical College, Medyczna 9, 30-688 Krakow, Poland; ∥ Department of Pharmacology, Maj Institute of Pharmacology Polish Academy of Sciences, Smetna 12, 31-343 Krakow, Poland; ⊥ Department of Pharmacology and Toxicology,University of Utah, Salt Lake City, Utah 84112, United States; # Department of Pharmacology and Physiology, 12312 Drexel University College of Medicine, Philadelphia, Pennsylvania 19102, United States; ∇ Department of Biotechnology and Life Sciences (DBSV),University of Insubria, 21100 Varese, Italy; ○ Centre for Neuroscience,University of Insubria, 21100 Varese, Italy; ◆ Biomedical Research Laboratory, Institute of Biological Sciences, Maria Curie-Skłodowska University, Akademicka 19, 20-033 Lublin, Poland; ¶ Department of Technology and Biotechnology of Drugs, Faculty of Pharmacy, Jagiellonian University Medical College, Medyczna 9, 30-688 Krakow, Poland; ⋈ Pharmacokinetics and Preliminary Toxicological Analysis Laboratory, Centre for the Development of Therapies for Civilization and Age-Related Diseases, Jagiellonian University Medical College, Medyczna 9, 30-688 Krakow, Poland; ⧓ Department of Crystal Chemistry and Crystal Physics, Faculty of Chemistry, Jagiellonian University, Gronostajowa 2 30-387, Krakow, Poland; ⧖ Department of Pharmaceutics, School of Pharmacy, University of Washington, Seattle, Washington 98195, United States

## Abstract

Strategic deuterium–hydrogen exchange applied to the first-in-class positive allosteric modulators (PAMs) of the glutamate transporter EAAT2/GLT-1, **(** ***R*** **)-AS-1** and **(** ***R*** **)-AS-7**, yielded novel analogues with improved drug-like properties. Specifically, incorporation of deuterium into the pyrrolidine-2,5-dione ring significantly prolonged the elimination half-life and increased both plasma and brain exposure in mice. These enhancements translated into more sustained antiseizure activity and a more favorable pharmacokinetic/pharmacodynamic (PK/PD) relationship. Similar to their nondeuterated counterparts, the new deuterated analogues displayed broad-spectrum antiseizure efficacy across multiple *in vivo* mouse seizure models, including maximal electroshock (MES), 6 Hz (32/44 mA), acute pentylenetetrazole (PTZ), and PTZ-induced kindling. Among these compounds, **d**_**6**_ **-(** ***R*** **)-AS-7** demonstrated the most robust antiseizure effects and the most advantageous overall pharmacokinetic profile following both intraperitoneal and oral administration. Mechanistic studies revealed that **d**_**6**_ **-(** ***R*** **)-AS-7** markedly enhanced glutamate uptake in COS-7 cells expressing EAAT2 as well as in primary astrocyte cultures. Furthermore, electrophysiological recordings in acute mouse hippocampal slices, together with two-electrode voltage-clamp recordings in *Xenopus laevis* oocytes expressing EAAT2, confirmed increased transporter-mediated currents. Collectively, these findings identify **d**_**6**_ **-(** ***R*** **)-AS-7** as a potent EAAT2 PAM with improved pharmacokinetic properties and strong antiseizure efficacy, supporting its further development as a therapeutic candidate for epilepsy and other disorders associated with glutamate excitotoxicity.

## Graphical abstract

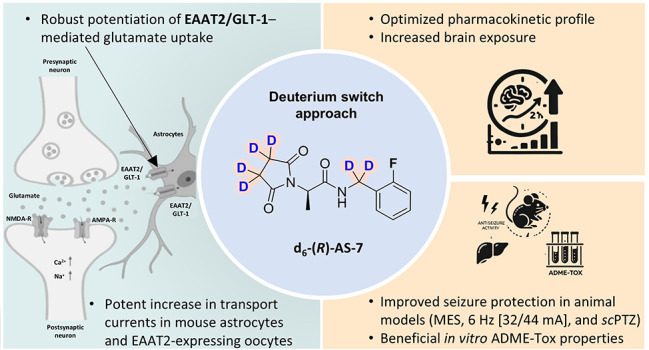

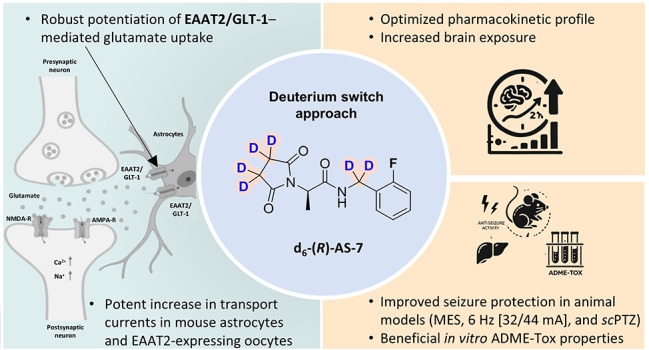

## Introduction

Intense research efforts during the past few decades brought numerous antiseizure medications (ASMs) to the market.^1^ Despite this unquestionable progress, over one-third of people with epilepsy still suffer from uncontrolled seizures and their life-threatening consequences.^2^ The complexity and poor understanding of the etiology of drug-resistant epilepsy (DRE) continue to propel drug discovery efforts focused on the identification of new molecular targets, and as a result, new chemical entities with a potential to improve DRE therapy.^3^ Accordingly, in our most recent studies, we have discovered a novel, first-in-class drug candidate compound **(** ***R*** **)-AS-1** (Figure 1), that is a selective positive allosteric modulator (PAM) of EAAT2 transporter of glutamate (named GLT-1 in rodents).^4,5^ The EAAT2 protein is mostly found in astrocytes, the non-neuronal cells in the central nervous system (CNS), and accounts for about 90% of glutamate (l-glutamate) uptake in the brain.^6,7^ Thus, direct glutamate uptake enhancement by targeting EAAT2 through PAMs is of great interest as it could provide first-in-class therapeutic approach for several neurological, neurodegenerative, and psychiatric diseases or conditions associated with increased glutamatergic tone (excitotoxicity)—in particular, epilepsy, neuropathic pain, amyotrophic lateral sclerosis, Alzheimer’s, Parkinson’s, Huntington’s diseases, ischemia, schizophrenia, anxiety, depression, addiction, autism, as well as traumatic brain injury.^6,8^ Previously obtained results with **(*R*)-AS‑1** demonstrated robust and potent protection across a broad panel of rodent models, including electrically evoked seizures (e.g., maximal electroshock [MES] and 6 Hz [32 and 44 mA] seizure models), chemically induced seizures (e.g., subcutaneous pentylenetetrazole [scPTZ] model), as well as seizures associated with viral infection of the brain (e.g., Theiler’s murine encephalomyelitis virus [TMEV]-induced seizure model).^4,5^ Despite having beneficial drug-like properties and satisfying *in vitro* metabolic stability in mouse and human liver microsomes, **(** ***R*** **)-AS-1** has a relatively short elimination half-life in mice.^4^ This observation indicates that in addition to hepatic biotransformation, a rapid renal clearance may be involved in its elimination, among others factors.

**Figure 1:**
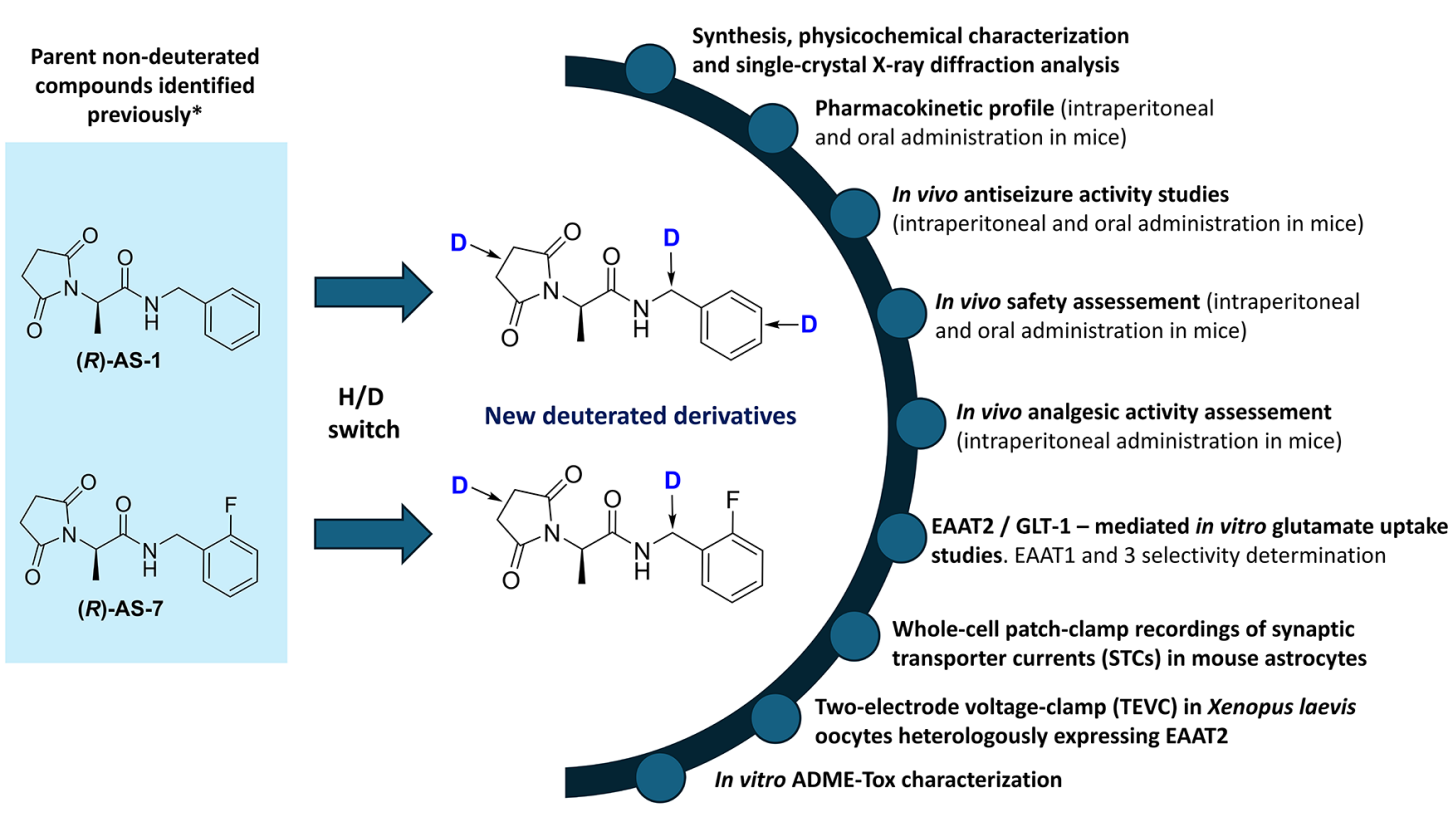
Development strategy leading to the identification of new deuterium containing PAMs of EAAT2/GLT-1 with potent antiseizure activity. [* Indicates that data for the parent (nondeuterated) molecules were described previously.^4,5^ ]

Several approaches can be explored in order to improve the pharmacokinetic (PK) and safety profile of drug candidates, such as bioisosteric modifications, controlled-release formulations, or prodrugs.^9–13^ Bioisosteric modifications often involve deuteration or fluorination of the parent molecule. While the deuteration is typically the most neutral bioisosteric replacement with negligible impact on physicochemical properties and ligand-protein complex formation, fluorination can lead to different modes of action and influence the ligand’s molecular conformation and its selectivity.^14,15^ Furthermore, fluorine-containing drugs may be associated with safety issues in humans and negative environmental impact.^16^ Thus, rationally designed deuteration process may be the most straightforward and efficient strategy to improve metabolic stability of drug candidates.

Aiming to optimize the PK profile and antiseizure properties of our drug candidates, we developed herein a focused series of deuterated analogues of **(** ***R*** **)-AS-1** and its fluorine counterpart, **(** ***R*** **)-AS-7**, disclosed previously (see Figure 1).^4,5^ For a more comprehensive understanding of the influence of the deuterium-switch approach on PK, antiseizure, and metabolic stability profiles, we performed targeted modifications by incorporation of different numbers of deuterium atoms (from 4 to 11), localized in different parts of these molecules.

To validate the deuterium-switch strategy as an effective chemical approach for lead optimization within a series of glutamate uptake enhancers bearing the (*R*)-*N*-benzyl-2-(2,5-dioxopyrrolidin-1-yl)­propanamide core,^4,5^ both the parent compounds **(** ***R*** **)-AS-1** and **(** ***R*** **)-AS-7** and their corresponding deuterated derivatives were extensively evaluated in *in vivo* PK studies and acute seizure models in male CD-1 mice. The optimized compound was further evaluated in a 6 Hz (32 mA) focal seizure model in C57BL/6J male and female mice. To confirm the mechanism of action, the compounds obtained in this study were tested in glutamate uptake assays under various experimental conditions using COS-7 cell lines expressing EAAT2 as well as rodent astrocytes. Furthermore, to gain a more detailed understanding of the mechanism of action, the influence of the lead compound on transporter currents was assessed using two electrophysiological approaches: whole-cell patch clamp recordings from astrocytes in acute mouse hippocampal slices and two-electrode voltage clamp (TEVC) in *Xenopus laevis* oocytes heterologously expressing the human glutamate transporter EAAT2. Importantly, for selected compounds, we performed a more detailed *in vivo* characterization in chronic seizure models (PTZ-kindling) and pain models in CD-1 mice, including the formalin and capsaicin tests, as well as models of neuropathic pain induced by oxaliplatin (OXPT) or streptozotocin (STZ). The *in vivo* efficacy studies were supplemented by safety profiling in models assessing motor coordination, neuromuscular strength, and the effect on locomotor activity of mice. Finally, the drug-like properties of selected molecules were assessed *in vitro*, including metabolic stability in mouse and human microsomes, liver S9 fractions, and hepatocytes; hepatotoxicity and neurotoxicity assays; induction of phospholipidosis, CYP profiling; parallel artificial membrane permeability assay (PAMPA); Caco-2 absorption model; and plasma protein binding.

## Results

### Synthesis

The starting noncommercial benzylamine derivatives (**A1**–**A4**) and target compounds, **d**_**4**_ **-(** ***R*** **)-AS-1**, **d**_**5**_ **-(** ***R*** **)-AS-1**, **d**_**6**_ **-(** ***R*** **)-AS-1**, **d**_**9**_ **-(** ***R*** **)-AS-1**, **d**_**11**_ **-(** ***R*** **)-AS-1**, **d**_**4**_ **-(** ***R*** **)-AS-7**, **d**_**6**_ **-(** ***R*** **)-AS-7**, were synthesized following the procedure illustrated in Scheme 1. Initially, the corresponding nitrile was reduced to the benzylamine derivatives (**A1**–**A4**) using LiAlH_4_ or LiAlD_4_ in anhydrous THF. The reduction was performed in an inert gas (argon) atmosphere and the reaction progress was monitored via HPLC. Upon completion, the reaction mixture was quenched, neutralized with 10% NaOH, and the resulting amine intermediate was isolated through extraction. The obtained deuterated benzylamines (**A1**–**A4**) were used in further synthesis without additional purification. The **A1**–**A4** preparation details are described in the Supporting Information (SI).

**Scheme 1:**
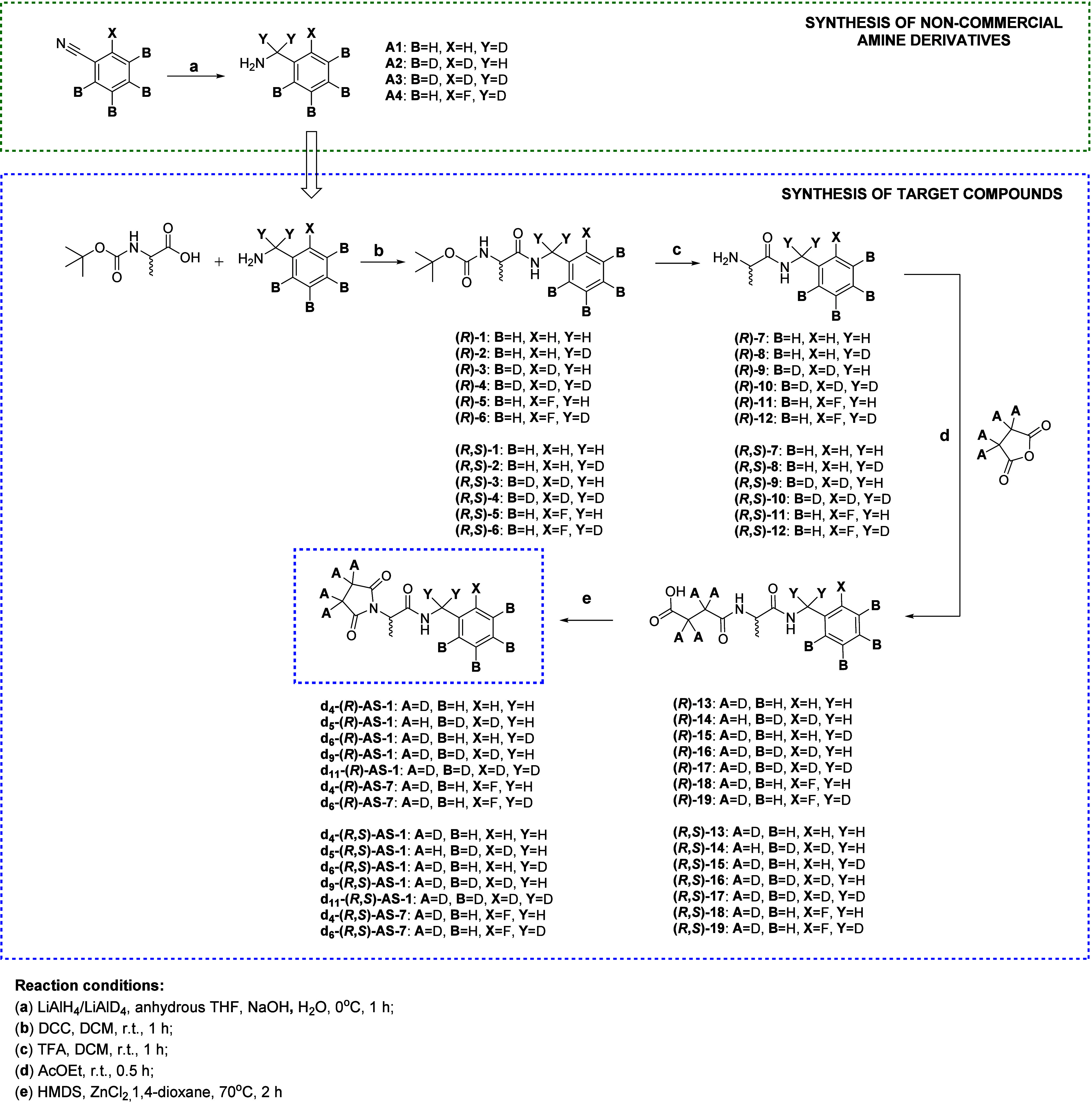
Synthesis of Starting Noncommercial Benzylamine Derivatives and Target Deuterium Containing Compounds

In the next step, Boc-d-alanine or Boc-d,l-alanine was coupled with the benzylamine or respective noncommercial benzylamine derivatives (**A1**–**A4**). The coupling reaction, carried out in the presence of dicyclohexylcarbodiimide (DCC), yielded the corresponding amide derivatives, (***R***)**-1**–(***R***)**-6** and (***R***, ***S***)**-1**–(***R***, ***S***)**-6**. Subsequently, the Boc group in (***R***)**-1**–(***R***)**-6** and (***R***, ***S***)**-1**–(***R***, ***S***)**-6** was removed using trifluoroacetic acid (TFA), followed by neutralization with ammonium hydroxide to generate the amine derivatives (***R***)**-7**–(***R***)**-12** and (***R***, ***S***)**-7**–(***R***, ***S***)**-12**. These amine intermediates were further reacted with equimolar amounts of succinic anhydride or *d*_4_-succinic anhydride to form the corresponding amidoacids (***R***)**-13**–(***R***)**-19** and (***R***, ***S***)**-13**–(***R***, ***S***)**-19**. Next, amidoacids (***R***)**-13**–(***R***)**-19** and (***R***, ***S***)**-13**–(***R***, ***S***)**-19** were subjected to an HMDS-promoted cyclization reaction, to yield the final deuterium-containing compounds **d**_**4**_ **-(** ***R*** **)-AS-1**, **d**_**5**_ **-(** ***R*** **)-AS-1**, **d**_**6**_ **-(** ***R*** **)-AS-1**, **d**_**9**_ **-(** ***R*** **)-AS-1**, **d**_**11**_ **-(** ***R*** **)-AS-1**, **d**_**4**_ **-(** ***R*** **)-AS-7**, **d**_**6**_ **-(** ***R*** **)-AS-7**, and **d**_**4**_ **-(** ***R***, ***S*** **)-AS-1**, **d**_**5**_ **-(** ***R***, ***S*** **)-AS-1**, **d**_**6**_ **-(** ***R***, ***S*** **)-AS-1**, **d**_**9**_ **-(** ***R***, ***S*** **)-AS-1**, **d**_**11**_ **-(** ***R***, ***S*** **)-AS-1**, **d**_**4**_ **-(** ***R***, ***S*** **)-AS-7**, **d**_**6**_ **-(** ***R***, ***S*** **)-AS-7**. In parallel to *R*-enantiomers, the corresponding racemates (*R*,*S*) were obtained for the purpose of determining enantiomeric purity.

The target compounds **d**_**4**_ **-(** ***R*** **)-AS-1**, **d**_**5**_ **-(** ***R*** **)-AS-1**, **d**_**6**_ **-(** ***R*** **)-AS-1**, **d**_**9**_ **-(** ***R*** **)-AS-1**, **d**_**11**_ **-(** ***R*** **)-AS-1**, **d**_**4**_ **-(** ***R*** **)-AS-7**, **d**_**6**_ **-(** ***R*** **)-AS-7** together with their racemates were synthesized with good yields (>80%) and their structures were confirmed by ^1^H NMR, ^13^C NMR, and LC-HRMS spectra. The purity of final compounds determined by use of chromatographic UPLC method was ≥99%. The enantiomeric purity of *R*-enantiomers, determined via chiral supercritical fluid chromatography (SFC) and chiral HPLC methods, exceeded 99%. The deuterium incorporation was ≥ 98%, as determined by ^1^H NMR spectral analysis. For proper visualization, representative overlays of the ^1^H NMR spectra of the parent compound **(** ***R*** **)-AS-1** and its deuterated analogues, **d**_**4**_ **-(** ***R*** **)-AS-1** (4 deuterium atoms) and **d**_**11**_ **-(** ***R*** **)-AS-1** (11 deuterium atoms), are provided in the SI (see the Section titled “1H NMR and 13C NMR Spectra for Final Compounds”, pages S67–S68). The absolute configuration was confirmed in a single-crystal X-ray diffraction (XRD) experiment, by the anomalous dispersion phenomenon. The results of the XRD analysis are shown in Figure S1 and Table S1. Furthermore, elemental analysis (C, H, and N) was performed for all final compounds. Details of the synthetic procedures and analytical data for the intermediates and final compounds are provided in the SI (“Materials and Methods” section).

### Pharmacokinetic Studies

Subsequently, we aimed to investigate the impact of the applied deuterium switch on PK properties by comparing the PK profiles and parameters of the deuterated derivatives with those of their nondeuterated parent compounds, **(** ***R*** **)-AS-1** and **(** ***R*** **)-AS-7**. Their concentrations in mouse serum and brain were determined by validated LC-MS/MS methods following intraperitoneal (*i.p*.) administration at two dose levels, 20 and 40 mg/kg. PK profiles of these compounds are shown in Figure 2, whereas PK parameters calculated based on concentration versus time data by the noncompartmental analysis are summarized in Tables S2 and S3.

**Figure 2:**
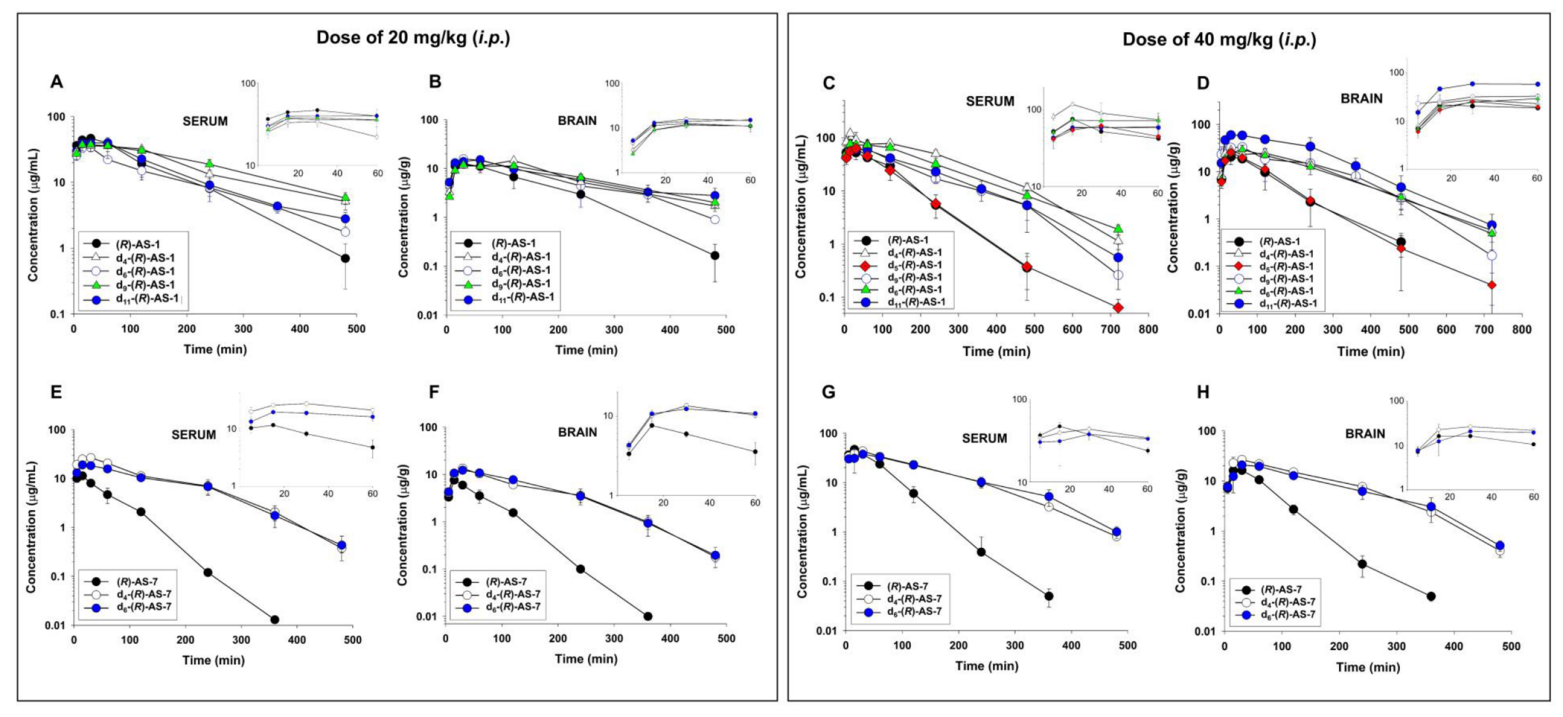
Serum (A, C, E, G) and brain (B, D, F, H) concentrations (± SD) of **(*R*)-AS‑1** and **(*R*)-AS‑7** and their deuterated derivatives as a function of time following *i.p.* administration (20 or 40 mg/kg) in male CD‑1 mice (*n* = 3–4). Insets in the top-right corners of each panel show concentration profiles at early time points (5–60 min).

As shown in Figure 2A–D and Table S2, **(** ***R*** **)-AS-1** and its deuterated derivatives reached their peak serum concentrations within 15–60 min after *i.p*. administration, indicating relatively rapid systemic exposure. For **(** ***R*** **)-AS-1**, *C*_max_ values increased proportionally with dose, whereas AUC values did not, demonstrating nonlinear PK. Specifically, a 2-fold dose increase led to only a 1.2-fold rise in AUC in serum and a 1.4-fold rise in brain. Interestingly, **d**_**5**_ **-(** ***R*** **)-AS-1** derivative, containing five deuterium atoms exclusively in the aromatic ring, showed concentration–time profiles in serum and brain that closely overlapped with those of the parent compound **(** ***R*** **)-AS-1**, resulting in similar PK parameters (Figure 2C and D, red points; Table S2). In contrast, the **d**_**4**_ **-(** ***R*** **)-AS-1** derivative displayed a disproportionate increase in serum exposure, with the AUC ratio rising to 2.8 at the higher dose, suggesting saturation of its elimination pathways (Table S2). For **d**_**6**_ **-(** ***R*** **)-AS-1** and **d**_**11**_ **-(** ***R*** **)-AS-1**, the brain AUC ratios were markedly higher, reaching 2.9 and 5.9, respectively, after administration of both doses. By comparison, **d**_**9**_ **-(** ***R*** **)-AS-1** exhibited linear PK, as doubling the dose resulted in an AUC ratio of 1.9 in both serum and brain. The elimination half-life of **(** ***R*** **)-AS-1** ranged from 47 to 75 min in both serum and brain (Table S2). Notably, all deuterated derivatives, except **d**_**5**_ **-(** ***R*** **)-AS-1**, showed much slower elimination, with significantly prolonged half-lives (Table S2, Figure 3A, B). For **d**_**6**_ **-(** ***R*** **)-AS-1** and **d**_**9**_ **-(** ***R*** **)-AS-1**, elimination half-lives were nearly doubled compared to **(** ***R*** **)-AS-1** at both 20 and 40 mg/kg doses. Moreover, deuteration generally resulted in higher AUC values (1.4–5.5-fold increases depending on tissue and dose) with exception of **d**_**5**_ **-(** ***R*** **)-AS-1**, **d**_**6**_ **-(** ***R*** **)-AS-1**, and **d**_**11**_ **-(** ***R*** **)-AS-1** at dose of 20 mg/kg (Figure 4A, B), while *C*_max_ remained largely unchanged. The brain-to-serum AUC ratios were in the range of 0.31–0.58 for the 20 mg/kg dose and 0.29–1.15 for the 40 mg/kg dose. The larger dispersion of these values for the higher dose is probably the result of a more pronounced saturation of the PK processes.

**Figure 3:**
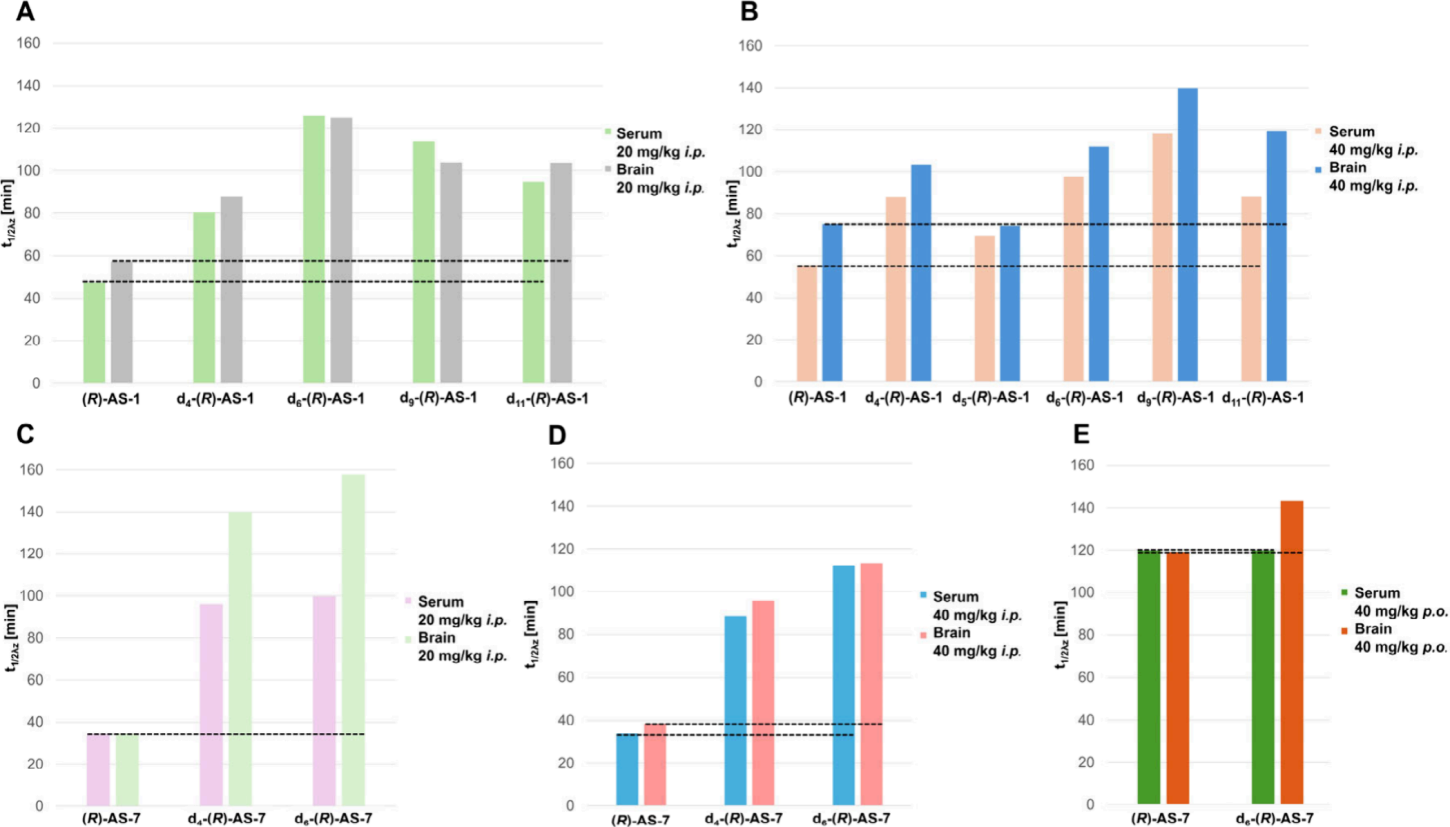
Elimination half‑lives of **(*R*)-AS‑1** (A, B) and **(*R*)-AS‑7** (C–E) and their deuterated derivatives in serum and brain of male CD‑1 mice following *i.p*. (20 and 40 mg/kg) or *p.o*. (40 mg/kg) administration.

**Figure 4:**
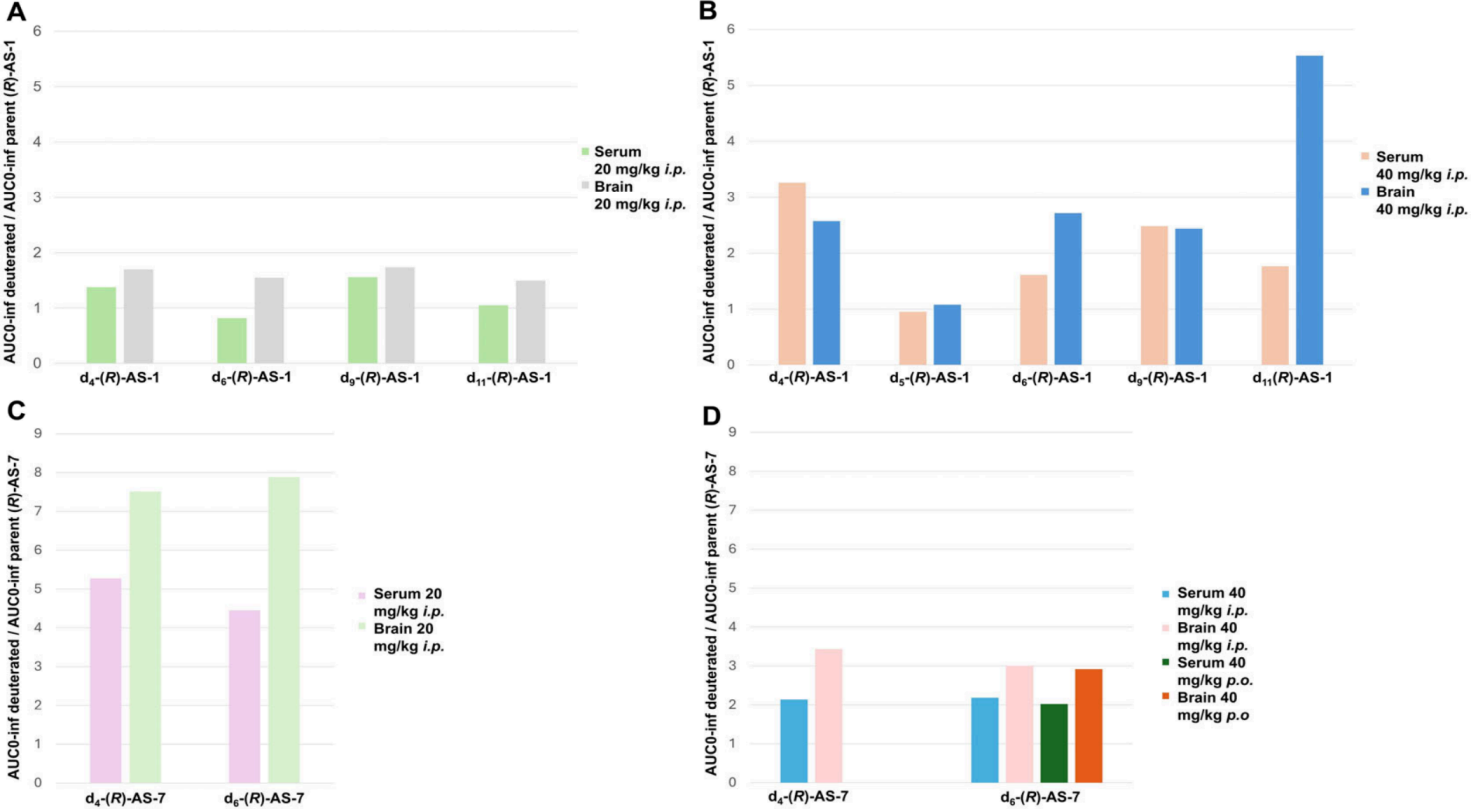
Ratios of AUC_0−**∞**_ values for deuterated analogues relative to their respective parent compounds, **(*R*)-AS‑1** (A, B) and **(*R*)-AS‑7** (C, D), in serum and brain of male CD‑1 mice following *i.p*. (20 and 40 mg/kg) or *p.o*. (40 mg/kg) administration.

In summary, deuteration of **(** ***R*** **)-AS-1**, particularly in the pyrrolidine-2,5-dione ring, led to prolonged systemic and brain exposure, reflected in longer elimination half-lives and higher AUC values. The only exception was **d**_**5**_ **-(** ***R*** **)-AS-1**, which behaved similarly to the parent compound. These findings underscore the impact of specific deuterium placement on the PK behavior of **(** ***R*** **)-AS-1** and highlight derivatives with improved exposure profiles. The percentage changes in selected PK parameters (*C*_max_, *t*_1/2λz_ and AUC_0‑∞_) of deuterated analogues, relative to parent **(** ***R*** **)-AS-1**, are summarized in Table S4.

As presented in Figures 2E–H and in Table S3, following *i.p.* administration, peak concentrations of **(** ***R*** **)-AS-7** and its deuterated derivatives (**d**_**4**_ **-(** ***R*** **)-AS-7** and **d**_**6**_ **-(** ***R*** **)-AS-7**) in serum and brain were generally reached within 15–30 min at both tested doses (20 and 40 mg/kg), indicating rapid absorption from the peritoneal cavity. In contrast, after oral dosing of **d**_**6**_ **-(** ***R*** **)-AS-7**, peak concentrations were delayed, occurring at 60 min in serum and 120 min in brain (Figure 5).

**Figure 5:**
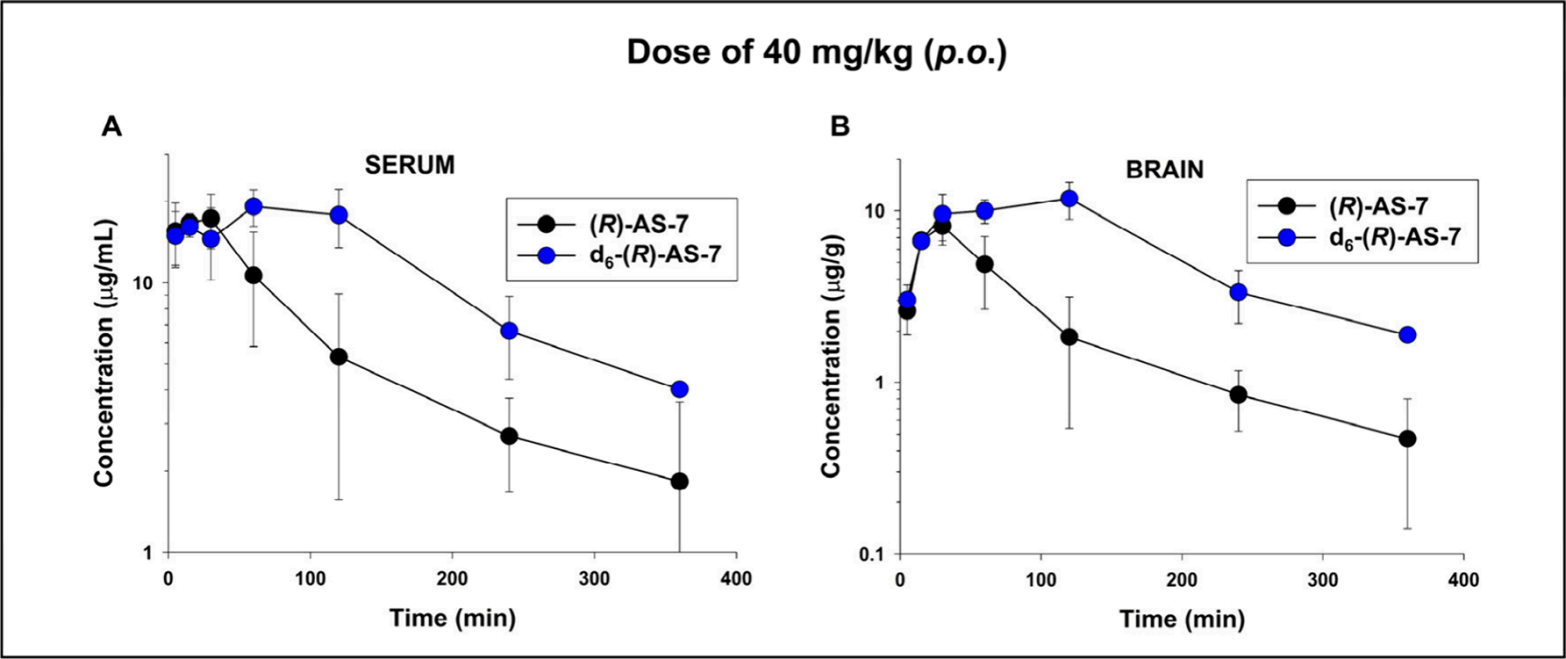
Serum (A) and brain (B) concentrations (± SD) of **(*R*)-AS‑7** and **d_6_-(*R*)-AS‑7** as a function of time following *p.o.* administration (40 mg/kg) in male CD‑1 mice (*n* = 3–4).

All tested compounds were able to cross the blood–brain barrier. For **(** ***R*** **)-AS-7**, brain-to-serum AUC ratios were 0.7 and 0.4 at 20 mg/kg and 40 mg/kg doses, respectively, while for the deuterated derivatives the ratios ranged from 0.56 to 1.23. Importantly, elimination of the deuterated derivatives was considerably slower than that of the parent compound. The terminal half-lives of **d**_**4**_ **-(** ***R*** **)-AS-7** and **d**_**6**_ **-(** ***R*** **)-AS-7** in serum and brain ranged from 88.7 to 157.8 min, compared to only 33.5–38.5 min for **(** ***R*** **)-AS-7** (Table S4, Figure 3C, D). Interestingly, such a distinct improvement in half-life was not observed after oral administration (Table S3, Figure 3E). The mean residence time (MRT) was slightly higher in brain than in serum for the deuterated compounds, which may help to reduce peripheral off-target effects. The estimated volume of distribution (*V*_*z*_/*F*) for **(** ***R*** **)-AS-7** was 1.215 L/kg, only slightly exceeding the total body water of mice (∼80% of fat-free wet weight), indicating moderate tissue distribution and limited tissue binding. For the deuterated derivatives, *V*_*z*_/*F* values were even lower. Total clearance (CL/*F*), calculated using a noncompartmental approach, was approximately 0.01 mL/min/kg, which is far below the hepatic blood flow in mice (2.25 mL/min). This suggests that **(** ***R*** **)-AS-7** is not extensively metabolized in the liver. However, since both *V*_*z*_ and CL after *i.p*. dosing depended on the fraction of dose absorbed (*F*), and bioavailability is not equal to 1 for extravascular routes, these parameters should be interpreted with caution. AUC ratios of **d**_**4**_ **-(** ***R*** **)-AS-7** and **d**_**6**_ **-(** ***R*** **)-AS-7** relative to the parent compound in serum ranged from 2.14 to 5.27 after *i.p.* administration and were about 2 after *p.o.* (Figure 4C, **D**). In brain, these AUC ratios were even higher: 3.0–7.88 after *i.p.* dosing and >3.0 for **d**_**6**_ **-(** ***R*** **)-AS-7** vs **(** ***R*** **)-AS-7** after *p.o.* administration. This demonstrates significantly greater brain exposure for the deuterated derivatives, regardless of the route of administration. The improved brain-to-serum AUC ratios of **d**_**4**_ **-(** ***R*** **)-AS-7** and **d**_**6**_ **-(** ***R*** **)-AS-7** indicate more favorable distribution to the target organ. Taken together, these results suggest that deuteration of **(** ***R*** **)-AS-7** prolongs systemic and brain exposure by slowing elimination, as evidenced by increased AUC values, particularly in the brain. This enhanced brain exposure may underlie stronger seizure protection of deuterated derivatives, especially at later time points (see Tables 1 and 2, and Figure 6), and could translate into therapeutic benefits. A summary of percentage changes in *C*_max_, *t*_1/2λz_, and AUC_0‑∞_ for **d**_**4**_ **-(** ***R*** **)-AS-7** and **d**_**6**_ **-(** ***R*** **)-AS-7**, relative to **(** ***R*** **)-AS-7**, is presented in Table S5.

**Table 1:** ED_50_, TD_50_, and PI Values in Male CD-1 Mice after *i.p.* Dosing of the Newly Obtained Deuterated Derivatives and Parent Compounds^a^

	ED_50_ MES [mg/kg]		ED_50_ 6 Hz (32 mA) [mg/kg]		ED_50_ 6 Hz (44 mA) [mg/kg]	
	Time point[Table-fn t1fn1a]		Time point[Table-fn t1fn1a]		Time point[Table-fn t1fn1a]	
Compound	0.5 h	2 h	0.5 h	2 h	0.5 h	2 h
**(*R*)-AS-1** ^b^	57.7 (45.5–73.1)	95.3 (79.6–114.1)	16.8 (11.6–24.2)	>130	77.3 (60.2–99.3)	>200
**d_4_-(R)-AS-1**	43.7 (38.3–50.0)	50.1 (44.4–73.1)	18.6 (11.5–30.2)	46.4 (40.5–53.3)	79.2 (62.9–99.8)	125.3 (98.9–158.6)
**d_6_-(*R*)-AS-1**	33.4 (29.5–37.7)	28.7 (17.3– 47.8)	27.4 (21.7–34.5)	33.0 (22.6–48.3)	85.6 (57.0–128.4)	50.8 (45.3–57.1)
**d_9_-(*R*)-AS-1**	45.0 (29.2–69.5)	57.7 (45.5–73.1)	18.2 (12.4–26.5)	68.0 (61.8–74.9)	78.3 (65.8-93.1)	109.8 (86.1–140.2)
**d_11_-(*R*)-AS-1**	29.5 (24.48–35.7)	33.8 (24.1–47.3)	15.7 (9.1–26.9)	41.6 (32.8–52.7)	57.6 (34.3–96.9)	70.4 (62.1–79.8)
**(*R*)-AS-7** ^b^	23.6 (13.8–40.4)	73.3 (57.4–93.5)	16.7 (11.6–24.1)	38.1 (27.7–52.5)	78.3 (85.8–93.1)	86.8 (79.3–95.2)
**d_4_-(*R*)-AS-7**	20.5 (16.8–25.0)	25.3 (20.8–30.8)	12.8 (10.5–16.0)	19.8 (13.3–29.4)	51.6 (31.3–85.2)	36.6 (24.8–53.1)
**d_6_-(*R*)-AS-7**	**13.4 (10.7–16.8)**	**14.1 (8.4–23.6)**	**12.5 (7.7–20.3)**	**13.4 (10.7–16.7)**	**25.1 (15.4–40.6)**	**33.5 (23.3–48.3)**

	TD_50_ rotarod [mg/kg]		PI (TD_50_/ED_50_)[Table-fn t1fn2a]	
	Time point[Table-fn t1fn1a]		Time point[Table-fn t1fn1a]	
Compound	0.5 h	2 h	0.5 h	2 h
**(*R*)-AS-1** ^b^	236.2 (225.7–247.1)	>300	4.1^c^	3.1^c^
			14.1^d^	2.3^d^
			3.1^e^	1.5^e^
**d_4_-(R)-AS-1**	176.7 (126.8–246.0)	>300	4.0^c^	6.0^c^
			9.5^d^	6.5^d^
			2.2^e^	2.4^e^
**d_6_-(*R*)-AS-1**	155.8 (141.6–171.4)	>300	4.7^c^	10.4^c^
			5.7^d^	9.1^d^
			1.8^e^	5.9^e^
**d_9_-(*R*)-AS-1**	165.4 (131.4–208.2)	>300	3.7^c^	5.2^c^
			9.1^d^	4.4^d^
			2.1^e^	2.7^e^
**d_11_-(*R*)-AS-1**	206.4 (178.9–238.2)	>300	7.0^c^	8.9^c^
			13.1^d^	7.2^d^
			3.6^e^	4.3^e^
**(*R*)-AS-7** ^b^	103.9 (91.2–118.5)	>300	4.4^c^	4.1^c^
			6.2^d^	7.9^d^
			1.3^e^	3.5^e^
**d_4_-(*R*)-AS-7**	96.7 (90.4–103.5)	119.8 (88.8–161.5)	4.7^c^	4.7^c^
			7.5^d^	6.1^d^
			1.9^e^	3.3^e^
**d_6_-(*R*)-AS-7**	**100.1 (92.0–108.8)**	**160.3 (152.6–168.4)**	**7.5^c^**	**11.4^c^**
			**8.0^d^**	**12.0^d^**
			**4.0^e^**	**4.8^e^**

a Results for the most effective compound are shown in bold for better visualization. Values in parentheses are 95% confidence intervals.

b Pretreatment time.

c Antiseizure activity results for **(R)-AS-1** and **(R)-AS-7** were also published previously in Abram *et al.* ^4^ (see compound **(R)-7 [(R)-AS-1]** and **(R)-8**, respectively).

d Protective indexes (TD_50_/ED_50_) in the:

e MES,

f 6 Hz (32 mA), and

g 6 Hz (44 mA) models. Discrepancies in the ED_50_ and TD_50_ values result from different formulations utilized in both studies, namely suspension in 1% Tween 80 (in Abram *et al.* ^4^) or solution in mixture of DMSO, PEG400, water for injection (1:4:5, *v*/*v*/*v*) herein.

**Table 2:** ED_50_, TD_50_, and PI Values in Male CD-1 Mice after *p.o.* Dosing of the **(** ***R*** **)-AS-7** and Its Deuterated Analogue **d**_**6**_ **-(** ***R*** **)-AS-7** ^a^

	ED_50_ MES [mg/kg]		ED_50_ 6 Hz (32 mA) [mg/kg]		ED_50_ 6 Hz (44 mA) [mg/kg]		TD_50_ rotarod [mg/kg]		PI (TD_50_/ED_50_)^c^	
	Time point^b^		Time point^b^		Time point^b^		Time point^b^		Time point^b^	
Compound	0.5 h	2.0 h	0.5 h	2.0 h	0.5 h	2.0 h	0.5 h	2.0 h	0.5 h	2.0 h
**(*R*)-AS-7**	28.4 (24.6–33.5)	62.9 (45.7–86.7)	30.8 (27.2–34.9)	66.1 (63.4–68.8)	50.9 (44.2–58.4)	103.8 (88.2–122.1)	246.6 (218.9–277.8)	>300	8.7^d^	4.8^d^
									8.0^e^	4.5^e^
									4.8^f^	2.9^f^
****d_6_**-**(*R*)-AS-7****	**22.6** **(20.1–25.3)**	**18.2** **(12.4–26.5)**	**16.7** **(11.6–24.2)**	**20.0 (16.6–23.9)**	**29.6 (24.5–35.8)**	**36.6 (24.9–53.1)**	**195.8 (164.6–232.8)**	**>300**	**8.7^d^**	16.5^d^
									**11.7^e^**	15.0^e^
									**6.6^f^**	**8.3^f^**

a Results for the most effective compound are shown in bold for better visualization. Values in parentheses are 95% confidence intervals.

b Pretreatment time.

c Protective indexes (TD_50_/ED_50_) in the:

d MES,

e 6 Hz (32 mA), and

f 6 Hz (44 mA) models.

**Figure 6:**
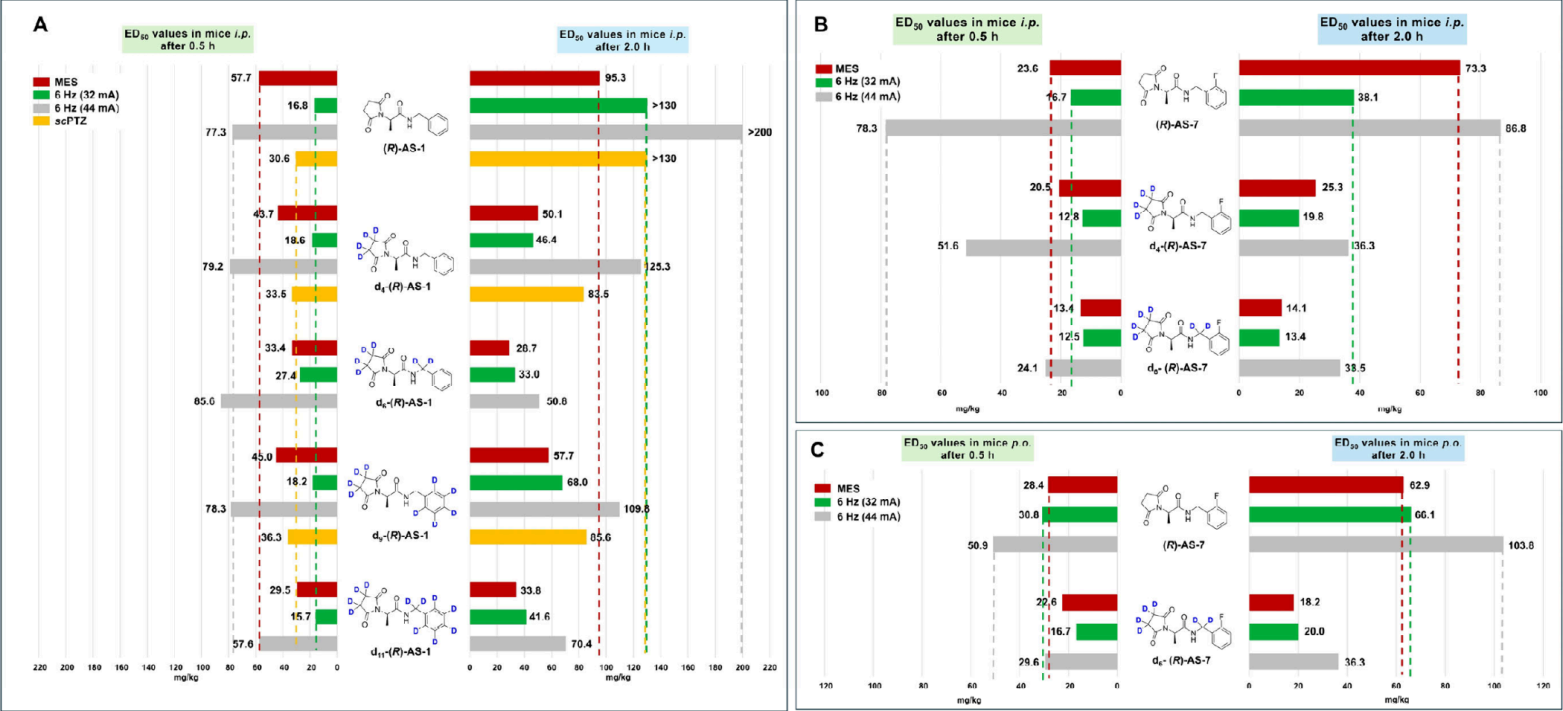
Comparison of ED_50_ values for **(** ***R*** **)-AS-1** and its deuterated analogues (A) and for **(** ***R*** **)-AS-7** and its deuterated congeners (B), administered *i.p.* to male CD-1 mice at 0.5 and 2 h. Bars represent data summarized in Table 1. (C) presents ED_50_ values for **(** ***R*** **)-AS-7** and its deuterated analogue **d**_**6**_ **-(** ***R*** **)-AS-7** administered *p.o.* to male CD-1 mice at 0.5 and 2 h, based on data from Table 2.

### *In Vivo* Antiseizure Activity in Acute Seizure Models

The antiseizure activity of the deuterated compounds, **d**_**4**_ **-(** ***R*** **)-AS-1**, **d**_**6**_ **-(** ***R*** **)-AS-1**, **d**_**9**_ **-(** ***R*** **)-AS-1**, **d**_**11**_ **-(** ***R*** **)-AS-1**, **d**_**4**_ **-(** ***R*** **)-AS-7**, **d**_**6**_ **-(** ***R*** **)-AS-7** was evaluated in three acute seizure models: the MES test, a model of generalized tonic-clonic seizures;^17,18^ the 6 Hz (32 mA) model of focal seizures,^19,20^ and the 6 Hz (44 mA) model of pharmacoresistant seizures.^18^ The compounds were administered *i.p.* to male CD-1 mice, and their effects were assessed at pretreatment times of 0.5 and 2 h. Moreover, for a head-to-head comparison, we also retested both parent molecules **(** ***R*** **)-AS-1** and (***R***)-**AS-7**, as their protective effects had not been studied after a longer pretreatment time (2 h). Notably, these additional data enabled the assessment of PK/PD relationships. Furthermore, aiming on more thorough investigation of antiseizure properties, selected compounds were also tested in the acute *sc*PTZ model of generalized absence seizures^21^ (**(** ***R*** **)-AS-1**, **d**_**4**_ **-(** ***R*** **)-AS-1**, and **d**_**9**_ **-(** ***R*** **)-AS-1**), as well as *iv*PTZ seizure threshold test and chronic PTZ kindling (**d**_**4**_ **-(** ***R*** **)-AS-1**). Additionally, a combination of one deuterated compound with its parent nondeuterated analogue was also evaluated after oral (*p.o.*) administration, similarly at 0.5 and 2 h pretreatment times. Lastly, a potential effect of compounds on the motor coordination of mice was studied in the standard fixed speed rotarod test as a part of an *in vivo* safety panel. Based on the aforementioned data, the protective index (PI = TD_50_/ED_50_), which describes the benefit-risk ratio of the candidate therapeutic agent, was calculated for each seizure model.

The results revealed that all compounds, including parent molecules and deuterated analogues, protected mice against seizures in the MES, 6 Hz (32 mA), and 6 Hz (44 mA) models (Table 1) in mice *i.p*. Furthermore, for better clarity and visualization of the differences in antiseizure activity between deuterated and nondeuterated compounds, the respective ED_50_ values at 0.5 and 2 h have been graphically compared in Figure 6A and B.

The *in vivo* data obtained at a shorter pretreatment time point of 0.5 h showed that, depending on the seizure model, deuterated compounds displayed similar or slightly better potency compared to both parent analogues, **(** ***R*** **)-AS-1** and (***R***)-**AS-7**. Importantly, the improved antiseizure activity of the deuterated derivatives correlated well with higher brain exposures obtained after treatment with these compounds (see AUC data discussion above and PK parameters summarized in Tables S2 and S3). Furthermore, as expected from the brain exposure data, more significant differences in ED_50_ values in all seizure models were observed 2 h after *i.p.* administration. At this longer pretreatment time, the deuterated analogues of parent **(** ***R*** **)-AS-1** were at least 1.6-fold more potent in the MES, 1.9-fold in the 6 Hz (32 mA), and 1.6-fold in the 6 Hz (44 mA) models. Similarly, results obtained for the deuterated analogues of **(** ***R*** **)-AS-7**, namely, **d**_**4**_ **-(** ***R*** **)-AS-7** and **d**_**6**_ **-(** ***R*** **)-AS-7**, showed clearly more potent protection at the longer time point of 2 h, at least of 2.9-fold in the MES, 1.5-fold in the 6 Hz (32 mA), and 2.4-fold in the 6 Hz (44 mA) vs parent compound. It should be stressed, however, that in parallel to more potent efficacy, the deuterated molecules, specifically **(** ***R*** **)-AS-1** derivatives, also induced stronger impairment of motor coordination in the rotarod test at 0.5 h, reflected in similar PI values, despite lower ED_50_ values. Importantly, both **(** ***R*** **)-AS-1** and its deuterated congeners did not produce symptoms of motor impairment up to a dose of 300 mg/kg at a time point of 2 h. It is also noteworthy that, compared to **(** ***R*** **)-AS-7**, **d**_**4**_ **-(** ***R*** **)-AS-7**, and **d**_**6**_ **-(** ***R*** **)-AS-7** did not show more pronounced motor coordination impairment at the shorter pretreatment time. Overall, the most potent protection in the electrically induced seizure models (MES, 6 Hz [32 and 44 mA]) was observed for deuterated and fluorine-containing molecules, **d**_**4**_ **-(** ***R*** **)-AS-7**, and in particular, **d**_**6**_ **-(** ***R*** **)-AS-7**. Notably, given the well-documented differences in antiseizure activity between mouse strains,^22^ the *in vivo* characterization of **d**_**6**_ **-(** ***R*** **)-AS-7** was also extended into an additional mouse strain (wild-type C57BL/6J) as well as female mice, as summarized in Figure S2. Consequently, the antiseizure activity of **d**_**6**_ **-(** ***R*** **)-AS-7**, particularly at a dose of 30 mg/kg, in the 6 Hz (32 mA) model in male and female C57BL/6J mice was comparable to that observed at established antiseizure doses of cannabidiol (CBD; 100 mg/kg) and levetiracetam (LEV; 25 mg/kg) in this test. Therefore, this novel compound demonstrated robust and time-dependent protection against 6 Hz (32 mA)-induced seizures in both male and female mice. There was no evidence of adverse side effects within either sex at the doses or time points tested. These data demonstrate that **d**_**6**_ **-(** ***R*** **)-AS-7** exhibits robust and reproducible antiseizure efficacy in both male and female mice with consistent effects observed across laboratories and over time.

In the next step of antiseizure properties evaluation, due to promising *i.p.* data in seizure models and beneficial PK profile, **d**_**6**_ **-(** ***R*** **)-AS-7** was subsequently tested in male CD-1 mice following *p.o.* administration at 0.5 and 2 h. For comparison purposes, similar studies were performed with its parent/nondeuterated compound—**(** ***R*** **)-AS-7**. Consequently, **d**_**6**_ **-(** ***R*** **)-AS-7** when given *p.o.* was effective in all seizure models (MES, 6 Hz [32/44 mA]), demonstrating potent and broad-spectrum protection with lower ED_50_ values than the parent compound **(** ***R*** **)-AS-7**, especially after 2 h of pretreatment (Table 2 and Figure 6C). Furthermore, at the time point of 2 h, **d**_**6**_ **-(** ***R*** **)-AS-7** showed distinctly more favorable PIs (>3 fold) vs **(** ***R*** **)-AS-7**. We observed only a slight decrease in potency in all seizure models after *p.o.* vs *i.p.*, indicating good oral bioavailability of **d**_**6**_ **-(** ***R*** **)-AS-7**, which evidently can achieve effective concentrations in the CNS when given orally.

It should be emphasized that, as shown in Table 3, compared to reference and clinically relevant ASMs, **d**_**6**_ **-(** ***R*** **)-AS-7** is distinctly more potent in each seizure model, compared to both CBD and VPA, which are recognized as multitarget ASMs used to treat different types of epilepsy, as well as provides wider spectrum of protection than LEV (SV2A protein ligand) which was active only in the 6 Hz (32 mA) model. These results also indicate similar efficacy and safety profiles (PIs) of **d**_**6**_ **-(** ***R*** **)-AS-7** as determined for LCS (sodium channel blocker).

**Table 3:** ED_50_, TD_50_, and PI Values in Male CD-1 Mice after *i.p.* Dosing for Reference and Mechanistically Diversified ASMs

Compound	PT(h) *^a^*	ED_50_ (MES) (mg/kg)	ED_50_ (6 Hz 32 mA) (mg/kg)	ED_50_ (6 Hz 44 mA) (mg/kg)	TD_50_ (rotarod) (mg/kg)	PI (TD_50_/ED_50_)^b^
**d**_**6**_ **-(** ***R*** **)-AS-7**	0.5	13.4 (10.7–16.8)	12.5 (7.7–20.3)	25.1 (15.4–40.6)	100.1 (92.0–108.8)	7.5 (MES)
						8.0 (6 Hz, 32 mA)
						4.0 (6 Hz, 44 mA)
**CBD** ^c^	1.0	80 (65.5–96.0)	144 (102–194)	173 (136–213)	272 (241–303)	3.4 (MES)
						1.9 (6 Hz, 32 mA)
						1.6 (6 Hz, 44 mA)
**LCS** ^d^	0.5	9.2 (8.5–10.0)	5.3 (3.5–7.8)	6.9 (5.4–8.6)	46.2 (44.5–48.0)	5.0 (MES)
						8.8 (6 Hz, 32 mA)
						6.7 (6 Hz, 44 mA)
**LEV** ^d^	1.0	>500	15.7 (10.4–23.7)	–	>500	>31.8 (6 Hz, 32 mA)
**VPA** ^d^	0.5	252.7 (220.1–290.2)	130.6 (117.6–145.2)	183.1 (143.5–233.7)	430.7 (407.9–454.9)	1.7 (MES)
						3.3 (6 Hz, 32 mA)
						2.3 (6 Hz, 44 mA)

a Pretreatment time.

b Protective Index (TD_50_/ED_50_). Reference ASMs:

c Cannabidiol (CBD) tested in male CF-1 mice, data from ref 23

d Lacosamide (LCS), levetiracetam (LEV) and valproate (VPA) tested in male CD-1 mice, data taken from own experiments.^4^ Values in parentheses are 95% confidence intervals.

Altogether, these data demonstrating potent antiseizure protection, together with a favorable PK profile and robust enhancement of EAAT2/GLT-1-mediated glutamate uptake (see below), indicate that **d**_**6**_ **-(** ***R*** **)-AS-7** is a highly promising EAAT2/GLT-1 PAM for further preclinical development in the epilepsy indication.

Next, two representative deuterated compounds, **d**_**4**_ **-(** ***R*** **)-AS-1** and **d**_**9**_ **-(** ***R*** **)-AS-1**, together with the parent comparator compound **(** ***R*** **)-AS-1**, were evaluated in the mouse *sc*PTZ model. As shown in Table S6 and Figure 6A, all compounds were equally active at 0.5 h, and more potent protection was provided by deuterated analogues after the longer pretreatment time point of 2 h. Apart from antiseizure activity, we also compared the latency time to the first seizure episode between the tested compounds and vehicle-treated groups. Thus, as shown in Figure S3, 0.5 h after *i.p.* administration, **d**_**4**_ **-(** ***R*** **)-AS-1** and **d**_**9**_ **-(** ***R*** **)-AS-1** prolonged the latency time to first seizure episode compared to the vehicle-treated group in a dose-dependent manner. Statistically significant results were obtained for both deuterated molecules at doses of 40 and 60 mg/kg. Slightly weaker activity and nondose proportional effects were observed for parent **(** ***R*** **)-AS-1**. Furthermore, 2 h after *i.p.* administration (Figure S4), **d**_**4**_ **-(** ***R*** **)-AS-1** and **d**_**9**_ **-(** ***R*** **)-AS-1** were more potent as they prolonged the latency time to first seizure episode compared to the vehicle-treated group at a dose of 100 mg/kg, whereas a similar statistically significant effect for **(** ***R*** **)-AS-1** was not observed until a dose of 130 mg/kg.

Notably, an example deuterated molecule—**d**_**4**_ **-(** ***R*** **)-AS-1**—was further characterized following *i.p.* administration in mice using the *iv*PTZ seizure threshold test, the PTZ kindling model, and a broad panel of pain models, including the formalin test and two neuropathic pain models of different origins, namely, peripheral neuropathy induced by OXPT or STZ. The results are summarized in the SI (see Section 3).

### Glutamate Uptake Studies in COS-7 Cell Line Mediated by EAAT2 and in Rodent Astrocytes

Our results indicate that compound **d**_**6**_ **-(** ***R*** **)-AS-7**, which demonstrated the most potent protection in seizure models, enhanced l-glutamate uptake mediated by EAAT2 in the COS-7 cell line, with a potency (EC_50_) of approximately 5 nM and a maximal efficacy (*E*_max_) of around 150%, as confirmed by two independent laboratories (Table 4). It is important to note that the modulatory activity of GT949, the early chemical prototype for glutamate uptake enhancers identified by our team, on EAAT2 has recently been called into question.^24^ These conflicting observations highlight the complexity of functionally validating EAAT2 modulation, which can be influenced by cellular context, assay conditions, and the choice of model systems. Therefore, EAAT2-mediated glutamate uptake results presented here for lead compound **d**_**6**_ **-(** ***R*** **)-AS-7** was independently confirmed and replicated in blinded studies conducted by two separate laboratories (see also Figures S5 and S6 for further details). The enhancement of glutamate uptake was subsequently corroborated in a more physiologically relevant model, using mouse (Table 4 and Figure S7) and rats (Table 4 and Figure S8) astrocyte cultures. Similarly, in these experimental systems, **d**_**6**_ **-(** ***R*** **)-AS-7** was effective glutamate uptake enhancer and showed potency (EC_50_) of ∼0.9 nM and a maximal efficacy (*E*_max_) of ∼130% in mouse astrocytes, as well as EC_50_ of ∼37 nM and *E*_max_ of ∼280% in rat astrocytes. Collectively, these results consistently demonstrate that **d**_**6**_ **-(** ***R*** **)-AS-7** enhances glutamate uptake across all assay systems, including COS-7 cells and primary mouse and rat astrocytes. Importantly, recent binding studies in rat astrocytes showed a potent interaction between **(** ***R*** **)-AS-7**, the direct nondeuterated chemical prototype for **d**_**6**_ **-(** ***R*** **)-AS-7** reported herein, and previously described as compound **(** ***R*** **)-8**,^4^ and EAAT2/GLT-1, with an IC_50_ of 33.6 ± 14.8 nM and a *K*_i_ of 31.4 ± 13.8 nM.^25^ These radioligand binding results strongly support the interaction of **(** ***R*** **)-AS-7** with the EAAT2 protein and further validate our functional assays.

**Table 4:** Glutamate Uptake Profile of the Lead Compound **d**_**6**_ **-(** ***R*** **)-AS-7** Determined in Three Experimental Systems: COS-7 Cells Overexpressing EAAT2, and Primary Astrocytes Derived from Mouse and Rat

	Glutamate uptake data^a^					
	COS-7 cell line with EAAT2 expression		In Mouse astrocyte cultures		In Rat astrocyte cultures	
Compound	EC_50_ ± SEM [nM]	*E*_max_ ± SEM [%]	EC_50_ ± SEM [nM]	*E*_max_ ± SEM [%]	EC_50_ ± SEM [nM]	*E*_max_ ± SEM [%]
**d_6_-(R)-AS-7**	5.8 ± 3.7^b^	154 ± 28^b^	0.92 ± 0.89^c^	130 ± 6^c^	37.7 ± 19^b^	284 ± 34^b^
	5.4 ± 4.5^c^	152 ± 10^c^				

a Results were normalized to a percentage of control and expressed as mean ± SEM from three to six independent experiments performed in technical triplicates; EC_50_ is the concentration at which the compound exerts 50% of its maximal effect; *E*_max_ represents the maximal glutamate uptake efficacy.

b Data obtained from The Department of Pharmacology and Physiology, Drexel University College of Medicine, Philadelphia, PA, 19102, USA.

c The Department of Pharmacology, Maj Institute of Pharmacology Polish Academy of Sciences, Krakow, Poland; parameters were calculated by using of GraphPad Prism 10.2.3 software.

The selectivity studies performed in COS-7 cells for **d**_**6**_ **-(** ***R*** **)-AS-7** showed that it does not modulate the glutamate uptake mediated by EAAT1 and EAAT3 (Figure S5). Thus, similar to its previously reported nondeuterated chemical prototypes, **(** ***R*** **)-AS-1** and in particular **(** ***R*** **)-AS-7**,^4^ **d**_**6**_ **-(** ***R*** **)-AS-7** acts as a selective enhancer of EAAT2 activity. Furthermore, kinetic analyses in EAAT2-mediated glutamate uptake in transfected COS-7 cells showed that 100 nM of **d**_**6**_ **-(** ***R*** **)-AS-7**, as well as **d**_**4**_ **-(** ***R*** **)-AS-7** significantly increase *V*_max_ by ∼145% of control (Figure S9).

Subsequently, kinetic analysis of glutamate uptake was also performed in primary rat astrocyte cultures in the presence of 10 and 100 nM compound **d**_**6**_ **-(** ***R*** **)-AS-7** (Figure 7). At these concentrations, **d**_**6**_ **-(** ***R*** **)-AS-7** increased *V*_max_ by approximately 132% and 208%, respectively. In contrast, *K*_m_ values remained unchanged under all conditions, indicating that the compound enhances glutamate transport through an allosteric mechanism without affecting the substrate affinity.

**Figure 7:**
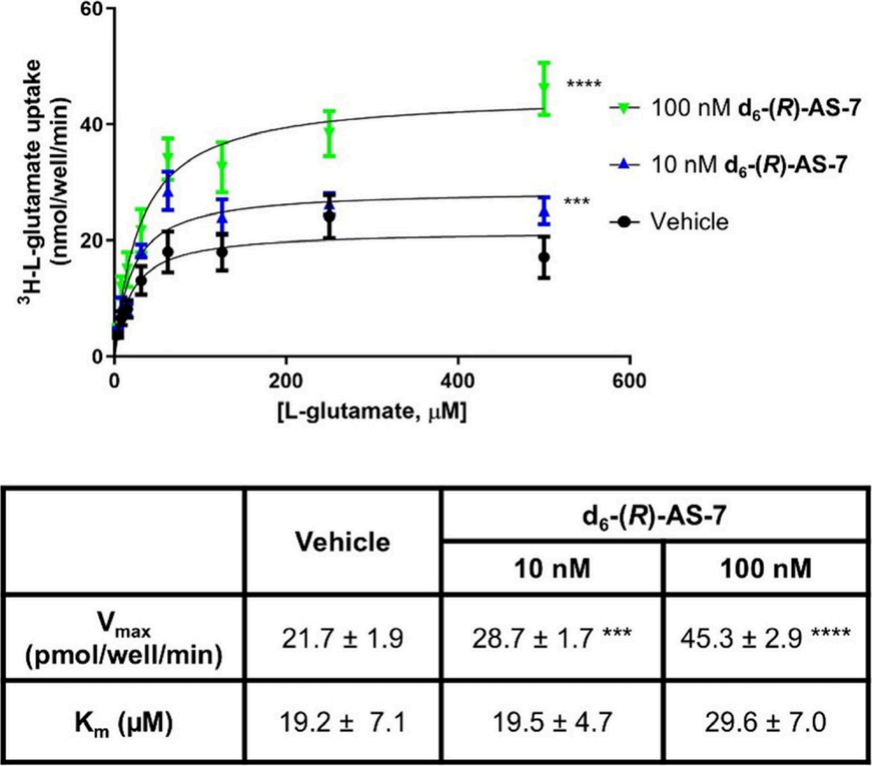
Kinetic analyses of the effect of compound **d**_**6**_ **-(** ***R*** **)-AS-7** in glutamate uptake in rat astrocyte cultures. Cells were preincubated with vehicle and 10 and 100 nM compounds for 10 min, then a range of concentrations of glutamate was added for an additional 10 min. Reactions were then terminated as described. *V*_max_ and *K*_m_ values are indicated in the table; *K*_m_ was not statistically different among conditions, whereas *V*_max_ is increased in the presence of compounds compared to the vehicle. Statistical analysis (GraphPad Prism 8.0.1): One-way ANOVA followed by Dunnett’s multiple comparisons *post hoc* test, *** *p* < 0.001 and **** *p* < 0.0001. Results are expressed in nmol/well/min (*V*_max_) and μM (K_m_) as the mean ± SEM of five independent experiments.

Similarly, other deuterated analogues acted as effective and selective EAAT2 enhancers, exhibiting nanomolar EC_50_ values and increased glutamate uptake in EAAT2-expressing COS-7 cells (Figures S5 and S6). As expected, these molecules also showed a robust enhancement of glutamate augmentation in astrocytes derived from both mice and rats (Figures S7 and S8).

### Influence on Transport Currents in Mouse Astrocytes

To further confirm that the compounds reported herein modulate transporter function and dynamics, we tested the influence of **d**_**6**_ **-(** ***R*** **)-AS-7** on transport currents in astrocytes recorded in mouse hippocampal brain slices. This compound was selected because it demonstrated potent enhancement of EAAT2/GLT-1-mediated glutamate uptake in COS-7 cells and rodent astrocytes and was also identified as the most effective antiseizure agent (see Table 1).

We recorded single stimulation-evoked synaptic transporter currents (STCs) in voltage-clamped astrocytes in the CA1 region of the hippocampus in acute brain slices to assess the effects of **d**_**6**_ **-(** ***R*** **)-AS-7** on glutamate STCs. Notably, when **d**_**6**_ **-(** ***R*** **)-AS-7** (10 μM) was perfused for approximately 15 min, STC amplitude (−50.7 pA ± 4.2) was significantly increased compared to baseline STC amplitudes (−28.4 pA ± 4.7; *p* < 0.0001; Figure 8C). STC amplitude was also significantly increased when normalized to baseline traces (*p* < 0.0005; data not shown). In addition to 10 μM **d**_**6**_ **-(** ***R*** **)-AS-7** perfusion, we also performed the same analyses following at least 15 min of 100 nM **d**_**6**_ **-(** ***R*** **)-AS-7** exposure. Similarly, when **d**_**6**_ **-(** ***R*** **)-AS-7** (100 nM) was perfused, STC amplitudes (−27.1 pA ± 4.2) were significantly increased compared to baseline STC amplitudes (−16.3 pA ± 4.1; *p* < 0.005; Figure 8B). Likewise, normalized STC amplitudes were also significantly increased following **d**_**6**_ **-(** ***R*** **)-AS-7** application (*p* < 0.005; data not shown). Together, STCs following perfusion of **d**_**6**_ **-(** ***R*** **)-AS-7** at either concentration (100 nM or 10 μM) significantly increased STC amplitude, suggesting an increase in the total amount of glutamate detected and taken up by the astrocytes.

**Figure 8:**
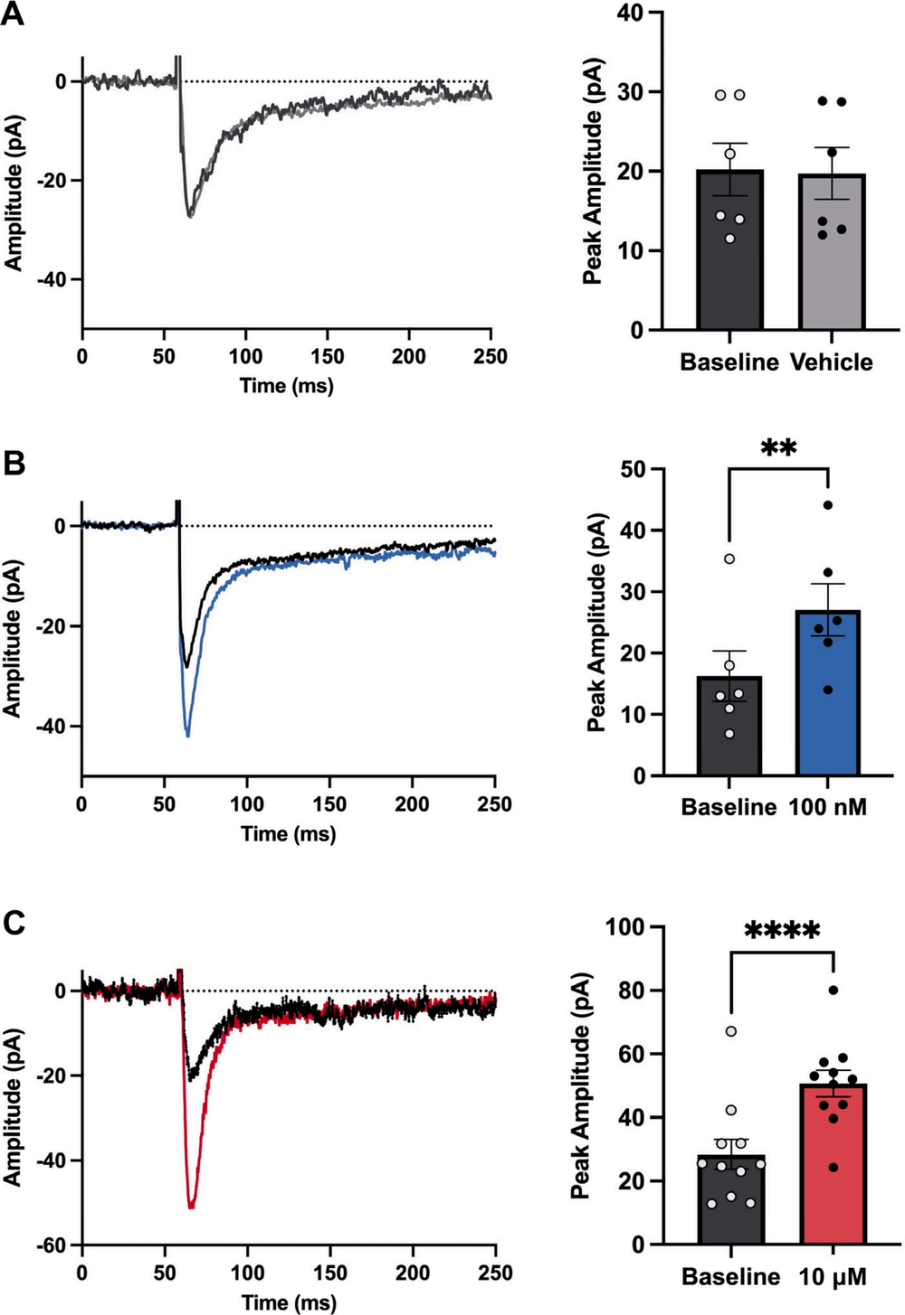
Synaptic transporter currents (STCs) following **d**_**6**_ **-(** ***R*** **)-AS-7** exposure exhibit increased peak amplitude. (A–C) Representative STC traces (left) from baseline (black line) and following **d**_**6**_ **-(** ***R*** **)-AS-7** or vehicle perfusion. Traces are represented as five averaged baseline and **d**_**6**_ **-(** ***R*** **)-AS-7** treated STCs from individual cells. Peak STC amplitude quantified as absolute values (right); (A) Vehicle control (0.01% *v*/*v* DMSO); (B) 100 nM **d**_**6**_ **-(** ***R*** **)-AS-7** (blue); (C) 10 μM **d**_**6**_ **-(** ***R*** **)-AS-7** (red). Statistical analysis (GraphPad Prism 8.0.1): Paired *t*-test; data represented as mean ± SEM; ** *p* < 0.01, **** *p* < 0.0001.

### Influence on Glutamate Transport Currents in EAAT2-Expressing *Xenopus laevis* Oocytes

To support the functional results obtained in astrocytes from mouse hippocampal slices, we measured the action of the PAM on the glutamate transport current (*I*_EAAT2_) in oocytes heterologously overexpressing EAAT2 in the presence and absence of **d**_**6**_ **-(** ***R*** **)-AS-7** at three different concentrations (Figure 9). To assess the effect of the compound, the oocytes expressing EAAT2 were perfused with **d**_**6**_ **-(** ***R*** **)-AS-7** alone for 30 s, then together with 1 mM glutamate. The effect at 100 nM **d**_**6**_ **-(** ***R*** **)-AS-7** was not clearly detectable. Increasing the concentration of **d**_**6**_ **-(** ***R*** **)-AS-7** to 1 μM increased the *I*_EAAT2_ amplitude significantly by 15%, compared to the value recorded before the treatment (*p* = 0.0004). The perfusion of 10 μM **d**_**6**_ **-(** ***R*** **)-AS-7** further amplified the effect, increasing the *I*_EAAT2_ in the presence of glutamate by 25% (*p* = 0.0002).

**Figure 9:**
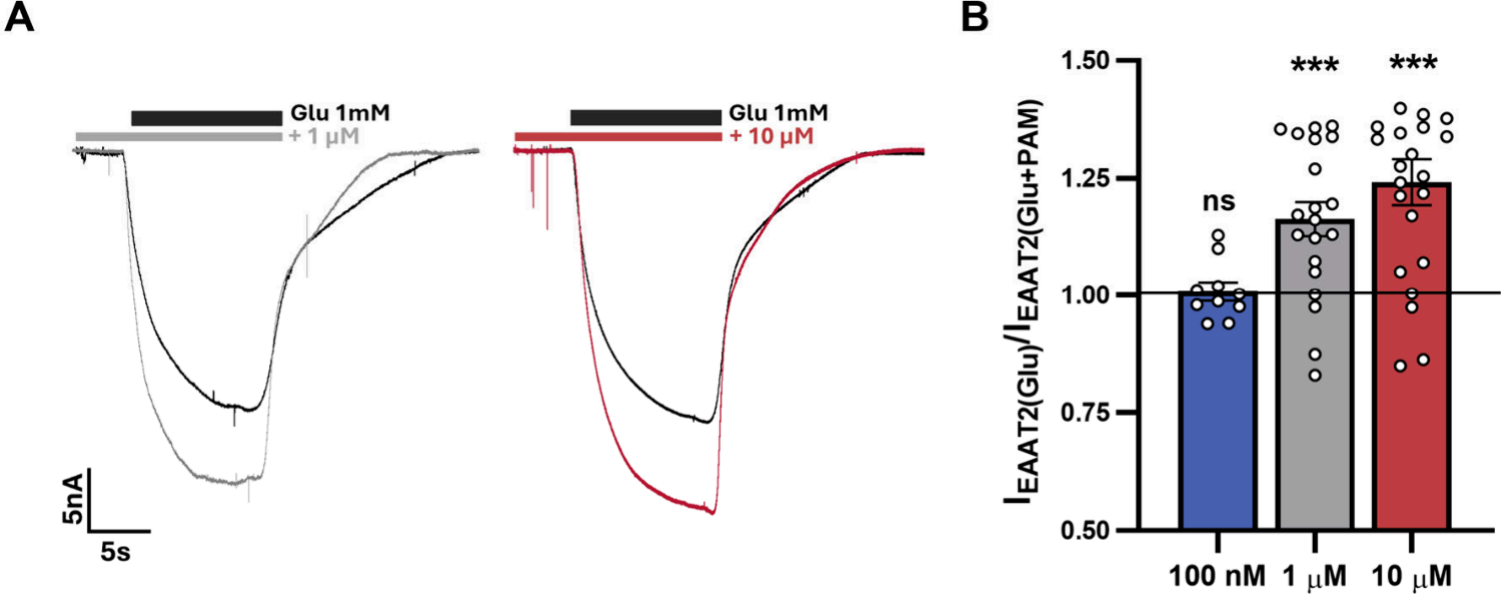
**d**_**6**_-(***R*** **)-AS-7** increases *I*_EAAT2_ in *Xenopus laevis* oocytes heterologously expressing the human glutamate transporter EAAT2. (A) Representative current traces elicited by 1 mM of glutamate, recorded in oocytes, before (black line) and after treatment with 1 μM (gray line) or 10 μM (red line) of **d**_**6**_-(***R*** **)-AS-7**. (B) The mean normalized amplitude of *I*_EAAT2_ recorded in the presence of **d**_**6**_-(***R*** **)-AS-7** at concentrations of 100 nM, 1 μM, and 10 μM. Statistical analysis (GraphPad Prism 8.4.3): Wilcoxon test comparing pre- and post-treatment values within each oocyte at each concentration; data are expressed as mean ± SEM; *** *p* < 0.001.

### *In Vitro* ADME-Tox Assays

The selected deuterated compounds, together with the parent nondeuterated molecules (used as comparators), were examined *in vitro* to determine key ADME-Tox parameters, including passive transport through biological membranes in the PAMPA assay, Caco-2 permeability assay, plasma protein binding, activity of CYP3A4, CYP2D6, and CYP2C9 isoforms, hepatotoxicity and neurotoxicity, phospholipidosis induction, and metabolic stability in mouse and human microsomes, liver S9 fraction, and hepatocytes. The obtained results are described in the SI (section 3).

## DISCUSSION AND CONCLUSIONS

Deuterium (D) is a natural hydrogen isotope which, in comparison to protium (H), displays a smaller molar volume (by 0.140 cm^3^ mol^–1^ per atom), a lower lipophilicity (Δlog *P*_oct_ – 0.006)^26^ and slightly altered p*K*_a_ value.^27^ Additionally, the C–D bond is shorter by 0.005 Å, which due to a 2-fold larger mass of D than H exhibits reduced vibrational stretching frequency compared with the C–H bond. Therefore, the C–D bond is more stable, with lower ground-state energy (by 1.2–1.5 kcal mol^–1^),^28^ which consequently requires greater activation energy for cleavage. This property is a drug development strategy to protect the molecule’s soft spots from CYP-mediated metabolism.^29,30^ The observed impact of deuterium on a drug candidate’s metabolic profile may lead to metabolic switching and metabolic shunting phenomenon.^31^ Moreover, deuterium incorporation at the stereocenter can reduce the enantiomerization and epimerization of a chiral compound.^32^ Although the difference in lipophilicity between H and D is negligible, the bioavailability of a drug candidate can be substantially modified in case of multiple substitutions within a molecule. Furthermore, deuteration may also affect blood–brain barrier penetration or the binding affinity to human serum albumin.^33,34^ The influence of deuterated analogues on ligand−target recognition and binding was also previously reported^35,36^ but this observation requires further investigations and wider scientific discussion. Notably, since the development of deutetrabenazine,^37^ the first deuterated drug approved by FDA in 2017, obtained through a deuterium switch approach, and designing of the first *de novo* deuterated drug deucravacitinib approved in 2022,^38^ many deuterium-containing bioactive molecules have been investigated at different drug development stages, including clinical trials.^31^

Substitution of a hydrogen atom with its stable isotope deuterium, known as deuteration, is especially useful to optimize PK and/or toxicity profile of drugs and drug candidates, potentially translating into increased efficacy or safety in comparison to the respective “hydrogen” parent compounds. Therefore, in the present chemical and pharmacological studies, we focused on a series of EAAT2/GLT-1 PAMs, which are deuterated analogues of previously described compounds.^4^ This study presents a comprehensive evaluation of the impact of deuterium incorporation on the PK profile and antiseizure efficacy of novel PAMs of the glutamate transporter EAAT2/GLT-1. The findings provide compelling evidence that hydrogen–deuterium exchange results in significantly improved PK profiles, enhanced brain penetration, and, consequently, more robust and sustained protection in seizure models compared to the nondeuterated parent compounds.

The incorporation of deuterium into specific molecular regions had a profound effect on PK properties. Consequently, the introduction of four deuterium atoms, particularly into the pyrrolidine-2,5-dione ring (site A, see compounds **d**_**4**_ **-(** ***R*** **)-AS-1**, **d**_**6**_ **-(** ***R*** **)-AS-1**, **d**_**9**_ **-(** ***R*** **)-AS-1**, **d**_**11**_ **-(** ***R*** **)-AS-1**, **d**_**4**_ **-(** ***R*** **)-AS-7**, **d**_**6**_ **-(** ***R*** **)-AS-7** in Scheme 1) resulted in a substantial extension of the terminal half-life (excluding **d_6_-(*R*)-AS-7** after *p.o*. administration), as well as a marked increase in both plasma and brain exposures after *i.p*. and *p.o*. administration in mice. This observation is likely due to protection of the heterocyclic ring against metabolic hydroxylation or the hydrogen-to-deuterium (H to D) isotope effect, which is well documented in keto–enol tautomerism characteristic for imides,^39–41^ where the equilibrium is shifted toward the keto form in the deuterated species.^42–45^ The preference for the keto form is related to stronger C–D vs C–H bond and the consequent weakening of the intramolecular chelate hydrogen bond upon deuteration, which was proven by the theoretical study based on multicomponent density functional theory.^46^ Consequently, stabilization of the keto form under *in vivo* conditions may protect the compounds from conjugation reactions during phase II of biotransformation. In addition, it may reduce the number of hydrogen bond donors (HBDs) from three (one enol and two amide groups) to two (amide groups), which can improve membrane permeability and lead to higher exposure in plasma and particularly in the brain. However, these assumptions warrant further and more detailed investigation, such as metabolic profiling or mass balance assays. Interestingly, hydrogen–deuterium exchange within aromatic ring (site B, see compound **d**_**5**_ **-(** ***R*** **)-AS-1** in Scheme 1) did not affect PK profile, while additional deuteration of the methylene fragment (site Y, see compounds **d**_**6**_ **-(** ***R*** **)-AS-1**, **d**_**9**_ **-(** ***R*** **)-AS-1**, **d**_**11**_ **-(** ***R*** **)-AS-1**, **d**_**6**_ **-(** ***R*** **)-AS-7** in Scheme 1) had inconclusive influence on concentration–time curves. Collectively, these findings support a critical role of imide ring deuteration in optimizing PK and antiseizure efficacy. Surprisingly, following oral administration, the deuterated analogue **d**_**6**_ **-(** ***R*** **)-AS-7** displayed terminal half-life and *C*_max_ values comparable to the parent **(** ***R*** **)-AS-7**, yet showed markedly increased exposure (AUC_0‑t_ and AUC_0**‑∞**_). The parallel decline of both compounds in the elimination phase indicates that deuteration did not modify the elimination rate but instead influenced the absorption process. The prolonged absorption phase, likely reflecting reduced first-pass metabolism and/or extended residence at the absorption site, resulted in substantially higher bioavailability of the deuterated analogue. This PK profile may offer clinical advantages by enabling less frequent dosing, minimizing peak-related adverse effects, and promoting more stable systemic drug levels.

The improved *in vivo* PK profile of the deuterated compounds compared to their hydrogen counterparts, however, did not correlate with the *in vitro* metabolic stability data obtained in mouse or human microsomes, hepatocytes, or S9 liver fractions, as both the deuterated and nondeuterated compounds exhibited high stability in these systems. Only an improvement was observed for the deuterium-containing molecule (**d**_**11**_ **-(** ***R*** **)-AS-1**), and this effect was limited to mouse hepatocytes. These results may indicate the presence of extrahepatic metabolism occurring outside the liver, involving other organs and tissues such as the gastrointestinal tract, kidneys, lungs, skin, brain, and plasma. Although the liver is the primary site of drug metabolism, extrahepatic sites can contribute significantly to drug elimination, disposition, and safety.^47,48^ While *in vitro* stability assays are indispensable tools early in drug discovery for screening metabolic stability and drug–drug interaction risks, their predictive accuracy and physiological representativeness are inherently limited by factors related to the biological system complexity, experimental conditions, and analytical methods used.^49,50^ Therefore, a more reliable and comprehensive evaluation of metabolism, along with a better understanding of the role of deuterium exchange in metabolic pathways, requires more detailed metabolic profiling in urine and feces, supported by *in silico* modeling.

In the acute mouse seizure models (MES, 6 Hz [32/44 mA], and *sc*PTZ), the deuterated analogues consistently demonstrated equal or greater antiseizure potency at 0.5 h and significantly enhanced protection at 2 h post-administration compared to their parent nondeuterated analogues, clearly supporting the PK/PD relationships. The strongest antiseizure protection was observed with **d**_**6**_ **-(** ***R*** **)-AS-7** following both *i.p.* and *p.o.* administration. This molecule was also effective in a second mouse strain in males (inbred, wild-type C57BL/6J), as well as in female C57BL/6J mice, further supporting its drug-like potential. Importantly, the robust and reproducible activity observed in this additional strain was consistent with that of prototypical ASMs, such as LEV and particularly CBD, which are known to be effective as adjunctive therapies in treatment-resistant epilepsy. This effect was seen in both sexes of mice, supporting the potential for future *in vivo* studies to justify first-in-human trials for difficult-to-treat epilepsy conditions. Notably, we herein demonstrate that **d**_**6**_ **-(** ***R*** **)-AS-7** is significantly more potent than several clinically relevant ASMs (including CBD, LEV and VPA), as well as **(** ***R*** **)-AS-1**, the first-in-class EAAT2/GLT-1 PAM described previously,^4,5^ in all mouse seizure models. It also provides a broader spectrum of protection than LEV, which was only effective in the 6 Hz (32 mA) test. The data obtained in these presently selected seizure and epilepsy models suggest that **d**_**6**_ **-(** ***R*** **)-AS-7** has a similar efficacy and safety profile (PI values) to LCS. It should be noted that prior findings for **(** ***R*** **)-AS-1** and **(** ***R*** **)-AS-7**, which share the same mechanism of action,^4,5^ along with data from **d**_**4**_ **-(** ***R*** **)-AS-1**, specifically its protection in PTZ-induced seizures, suppression of kindling development in the PTZ kindling model, reduction in tonic hindlimb extension in the *iv*PTZ test, as well as its analgesic activity in the formalin model and antinociceptive effects in OXPT and STZ-induced neuropathic pain models, suggest **d**_**6**_ **-(** ***R*** **)-AS-7** may also offer protective activity in a broader range of advanced seizure and pain models. In the mechanistic studies, **d**_**6**_ **-(** ***R*** **)-AS-7** demonstrated nanomolar EC_50_ values and enhanced glutamate uptake efficacy (*E*_max_) across COS-7 cells expressing EAAT2, as well as in primary mouse and rat astrocytes. The kinetic studies showed increased *V*_max_ without significant changes in *K*_m_ value, indicating that **d**_**6**_ **-(** ***R*** **)-AS-7** acts through an allosteric mechanism without affecting substrate affinity. Notably, these functional enhancements were further supported by transporter current studies in astrocytes, where **d**_**6**_ **-(** ***R*** **)-AS-7** elicited marked increases in GLT-1-mediated inward currents. Importantly, peak STC amplitude reflects the total amount of glutamate transported by the astrocyte. As such, increases in peak amplitude indicate an increase in glutamate uptake by astrocytes.^51,52^ Consistent with the observations in mouse hippocampal slices, heterologous expression of human EAAT2 in *Xenopus laevis* oocytes showed similar behavior. The increase in the amplitude of I_EAAT2_ was ∼15% and ∼25% for 1 μM and 10 μM of **d**_**6**_ **-(** ***R*** **)-AS-7**, respectively. The difference in effective PAM concentration likely reflects properties of the *Xenopus laevis* oocyte expression system. Oocytes are large cells with diffusion barriers^53^ and exhibit very high transporter expression, resulting in currents in the tens of nA range. As a result, PAM concentrations effective in cell-based assays may be insufficient in oocytes, where the activity, the transport currents, is several orders of magnitude higher.^54^ Together, these findings provide clear evidence that **d**_**6**_ **-(** ***R*** **)-AS-7** effectively potentiates EAAT2-mediated responses in both orthologues (human and mouse) of the transport protein. Importantly, selectivity profiling showed that **d**_**6**_ **-(** ***R*** **)-AS-7** did not modulate EAAT1- or EAAT3-mediated uptake, thereby preserving the desired selectivity toward EAAT2. Similarly, other deuterated analogues reported herein retained their potency as EAAT2 enhancers, demonstrating nanomolar EC_50_ values, increased glutamate uptake efficacy (*E*_max_) in COS-7 cells expressing EAAT2, as well as maintained EAAT2 selectivity.

These data, together with the distinctly improved PK profile and favorable *in vitro* drug-like properties (e.g., high metabolic stability in human/mouse microsomes, S9 liver fraction and hepatocytes, low hepatotoxicity and neurotoxicity potential, minimal influence on CYPs, and low risk of phospholipidosis induction), as well as potent enhancement of glutamate uptake, position **d**_**6**_ **-(** ***R*** **)-AS-7** as a highly promising EAAT2/GLT-1 PAM for further preclinical and clinical development in epilepsy and importantly nonepilepsy indications. Given its structural similarities to **(** ***R*** **)-AS-1** and previous data,^5^ it is also unlikely that **d**_**6**_ **-(** ***R*** **)-AS-7** will increase EAAT2/GLT-1 expression after chronic administration, though further studies are needed to confirm this.

It is important to recognize that despite rapid progress in pharmaceutical and medical sciences, the discovery of new ASM candidates still largely relies on a target-agnostic (phenotypic) approach using predictive *in vivo* models. Among these, the MES, 6 Hz, and *sc*PTZ seizure tests have been widely used as initial screening assays in ASM discovery and they were also employed in the present study for initial screening of antiseizure efficacy of the new compounds. However, it should be noticed that these acute models do not capture all mechanisms of antiseizure activity and may fail to identify compounds acting through pathways not adequately represented in these assays.^17^ In fact, the *sc*PTZ test has been deprioritized by the Epilepsy Therapy Screening Program (ETSP) of the National Institute of Neurological Diseases and Stroke (NIH, Bethesda, MD, USA) and the initial evaluation of candidate compounds submitted to the ETSP now occurs in two acute models, i.e., the MES and 6 Hz tests. Due to their limitations, more etiologically relevant disease models of chronic network hyperexcitability and/or spontaneous seizures have been incorporated in both the early (identification) and late phase of ETSP.^20,55^ Therefore, further studies using more disease-relevant and chronic epilepsy models are necessary to better characterize the therapeutic potential of the investigated compounds. Accordingly, the lead compound **d**_**6**_ **-(** ***R*** **)-AS-7** is currently studied in more advanced seizure models by the ETSP, i.e., in the lamotrigine-resistant amygdala kindling rat model and the chronic rat kainate model. Its effects on the progression of seizures in the amygdala kindling model in mice are also being evaluated. Furthermore, this compound is undergoing assessment unrelated to any ETSP studies using male and female mice with genetic variants in presenilin 2 (PSEN2), a genetic risk factor implicated in the pathology of Alzheimer’s disease.^56^ Additional directions for preclinical development will include *in vivo* models of neuropathic pain, depression, anxiety, amyotrophic lateral sclerosis, and stroke, all of which are conditions in which glutamate excitotoxicity is well documented. In parallel with the efficacy studies, detailed safety assessments and comprehensive DMPK investigations following both single and chronic dosing are planned in the near future.

Collectively, our studies demonstrate that rational deuteration is a powerful and practical strategy for lead optimization in the series of glutamate uptake enhancers described herein. We confirmed that deuterium incorporation led to improvements in both PK profiles and antiseizure efficacy in mice. Finally, as expected, the hydrogen–deuterium exchange strategy did not alter molecular properties regarding intermolecular and intramolecular interaction propensity nor did it affect the primary mechanism of action.

## LIMITATIONS

Rodents are known to metabolize drugs considerably faster than primates and especially humans; therefore, the metabolic stability and biotransformation pathways of the deuterated EAAT2/GLT-1 PAMs described here may differ markedly from those observed clinically. Consequently, although these compounds displayed improved PK profiles in mice compared to their nondeuterated prototypes, such pronounced effects may not fully translate to humans. This is particularly relevant because the metabolic stability of both parent and deuterated second-generation glutamate uptake enhancers may already be sufficient to achieve therapeutically meaningful plasma and brain concentrations with once- or twice-daily dosing in humans. Moreover, the limited translational value of standard *in vitro* metabolic stability systems, such as microsomes, S9 fractions, and hepatocytes, underscores the need for caution when extrapolating these findings. These assays failed to predict the *in vivo* improvements observed in rodents, suggesting a meaningful contribution from extrahepatic metabolic pathways that remain to be defined. Thus, only rigorous head-to-head comparator studies in humans will ultimately determine the true utility of the deuterium-switch strategy for improving the metabolic stability of EAAT2/GLT-1 PAMs. Comprehensive metabolite profiling and mass balance assessments will also be essential to elucidate the specific biotransformation routes affected by deuteration and to validate the relevance of the kinetic isotope effect in human physiology.

## Materials and Methods

The Supporting Information (SI) includes all of the information about the materials and methods used in this study. All animal experiments were conducted in accordance with European Directive 2010/63/EU for the protection of animals used for scientific purposes and the National Institutes of Health Guide for the Care and Use of Laboratory Animals. The experimental protocols were approved by the I Local Ethical Committee for Experiments on Animals at the Jagiellonian University in Krakow (Poland): Approval Nos. 270/2019 and 412/2020 for PK studies; Approval Nos. 463/2020 and 512A/2020 for the MES, 6 Hz (32/44 mA), *sc*PTZ, and rotarod models; and Approval Nos. 104/2015, 279/2019, and 614/2022 for antinociceptive models and the locomotor activity test. Additional experiments were approved by the Local Ethics Committee for Experiments on Animals in Lublin (Poland): Approval Nos. 13/2021 and 46/2021 for the *iv*PTZ seizure threshold test, grip strength test, and the PTZ-induced kindling model. The glutamate transporter studies in rat astrocytes were approved by the Drexel University Institutional Animal Care and Use Committee (IACUC) (Protocol No. LA-23–749) under U.S. OLAW. Assurance No. A3222–01. All efforts were made to minimize animal suffering and reduce the number of animals used.

## Supplementary Material



## Notes

Supplementary figures and tables; additional studies comprising single crystal XRD, crystal structure determination and analysis, *iv*PTZ seizure threshold test and PTZ-induced kindling model, *in vivo* antinociceptive activity, *in vitro* ADME-Tox assays; experimental procedures as well as compound characterization data including chiral SFC chromatograms, UPLC/HRMS traces, and ^1^H and ^13^C NMR spectra (PDF)
