# Supplementary material for: Second-Generation of Deuterium-Substituted Glutamate Uptake Enhancers Exhibit Superior Drug-Like Properties in Preclinical Evaluation
Source: ACS Cent Sci. 2026 May 29;12(6):807–23. doi: 10.1021/acscentsci.6c00080 (PMC13306594; doi:10.1021/acscentsci.6c00080)

Supporting Information

Second-Generation of Deuterium-Substituted Glutamate Uptake Enhancers Exhibit Superior Drug-Like Properties in Preclinical Evaluation

Michał Abram [1], Marcin Jakubiec [1], Małgorzata Szafarz [2], Anna Rapacz [3], Magdalena Kolasa [4], Agata Faron-Górecka [4], Szczepan Mogilski [3], Kaliana Veros [5], Simran K. Gill [6], Angela Di Iacovo [7,8], Katarzyna Socała [9], Gniewomir Latacz [10,11], Joanna Karnafał [10], Krzysztof Pociecha [2], Justyna Kalinowska-Tłuścik [12], Melissa Barker-Haliski [13], Elżbieta Wyska [2], Andréia C. K. Fontana [6], Piotr Wlaź [9], Rafał Kamiński [1], Cristina Roseti [7,8], Elena Bossi [7,8], Karen S. Wilcox [5] and Krzysztof Kamiński [1,*]

[1] Department of Medicinal Chemistry, Faculty of Pharmacy, Jagiellonian University Medical College, Medyczna 9, 30-688 Krakow, Poland

[2] Department of Pharmacokinetics and Physical Pharmacy, Faculty of Pharmacy, Jagiellonian University Medical College, Medyczna 9, 30-688 Krakow, Poland

[3] Department of Pharmacodynamics, Faculty of Pharmacy, Jagiellonian University Medical College, Medyczna 9, 30-688 Krakow, Poland

[4] Department of Pharmacology, Maj Institute of Pharmacology Polish Academy of Sciences, Smętna 12, 31-343 Krakow, Poland

[5] Department of Pharmacology and Toxicology, University of Utah, Salt Lake City, UT 84112, United States

[6] Department of Pharmacology and Physiology, Drexel University College of Medicine, Philadelphia, PA 19102, United States

[7] Department of Biotechnology and Life Sciences (DBSV), University of Insubria, Varese, Italy

[8] Centre for Neuroscience, University of Insubria, Varese, Italy

[9] Biomedical Research Laboratory, Institute of Biological Sciences, Maria Curie-Skłodowska University, Akademicka 19, 20–033 Lublin, Poland

[10] Department of Technology and Biotechnology of Drugs, Faculty of Pharmacy, Jagiellonian University Medical College, Medyczna 9, 30-688 Krakow, Poland

[11] Pharmacokinetics and Preliminary Toxicological Analysis Laboratory, Centre for the Development of Therapies for Civilization and Age-Related Diseases, Jagiellonian University Medical College, Medyczna 9, 30-688 Krakow, Poland

[12] Department of Crystal Chemistry and Crystal Physics, Faculty of Chemistry, Jagiellonian University, Gronostajowa 2, 30-387 Krakow, Poland

[13] Department of Pharmaceutics, School of Pharmacy, University of Washington, Seattle, WA 98195, United States

Corresponding Author

*K.K.: Phone, +48 12 620 54 59; fax, +48 12 620 54 58; e-mail, k.kaminski@uj.edu.pl.

Table of contents

1. Tables ... 4

Table S1. Crystal data and structure refinement results ... 4

Table S2. Pharmacokinetic parameters of **(*R*)-AS-1**, **d_4-(*R*)-AS-1**, **d_6-(*R*)-AS-1**, **d_9-(*R*)-AS-1** and **d_{11}-(*R*)-AS-1** in serum and brain tissue following *i.p.* administration of these compounds at a dose of 20 mg/kg and/or 40 mg/kg to male CD-1 mice ... 5

Table S3. Pharmacokinetic parameters of **(*R*)-AS-7**, **d_4-(*R*)-AS-7** and **d_6-(*R*)-AS-7** in serum and brain tissue following *i.p.* and *p.o.* administration of these compounds at a dose of 20 mg/kg and 40 mg/kg to male CD-1 mice. ... 6

Table S4. Percent change (%Δ) in selected pharmacokinetic parameters of deuterated analogs, relative to parent **(*R*)-AS-1**, in serum and brain tissues following intraperitoneal (*i.p.*) administration at doses of 20 mg/kg and 40 mg/kg in male CD-1 mice. ... 7

Table S5. Percent change (%Δ) in selected pharmacokinetic parameters of deuterated analogs, relative to **(*R*)-AS-7**, in serum and brain tissue following intraperitoneal (*i.p.*) or oral (*p.o.*) administration at doses of 20 mg/kg and 40 mg/kg in male CD-1 mice ... 8

Table S6. Antiseizure activity in the *sc*PTZ model following *i.p*. administration in male CD-1 mice ... 9

2. Figures ... 10

Figure S1. Asymmetric units (left column) and a single molecule geometry (right column) presenting the numbering scheme for the enantiopure crystal structures of **d_4-(*R*)-AS-1**, **d_6-(*R*)-AS-1**, **d_4-(*R*)-AS-7** and **d_6-(*R*)-AS-7**. ... 10

Figure S2. Antiseizure activity of **d_6-(*R*)-AS-7** in the 6 Hz (32 mA) seizure model following *i.p*. administration in male (M) and female (F) C57BL/6J mice ... 11

Figure S3. Effects of the deuterated analogues **d_4-(*R*)-AS-1**, **d_9-(*R*)-AS-1**, and parent **(*R*)-AS-1** on latency time to first clonus in the *sc*PTZ test (male CD-1 mice, *i.p.*) in the pretreatment time 0.5 h ... 12

Figure S4. Effects of the deuterated analogues **d_4-(*R*)-AS-1**, **d_9-(*R*)-AS-1**, and parent **(*R*)-AS-1** on latency time to first clonus in the *sc*PTZ test (male CD-1 mice, *i.p.*) in the pretreatment time 2 h ... 12

Figure S5. Glutamate uptake mediated by EAAT2, EAAT1 and EAAT3 in COS-7 cells transiently transfected with CMV, EAAT1, EAAT2 or EAAT3 ... 13

Figure S6. EAAT2-mediated glutamate uptake in COS-7 cells for representative deuterium-containing compounds (replication studies) ... 14

Figure S7. Representative deuterium-containing compounds enhance glutamate uptake in mouse astrocytes ... 15

Figure S8. Representative deuterium-containing compounds enhance glutamate uptake rat astrocytes ... 16

Figure S9. Kinetic analyses of the effect of **d_4-(*R*)-AS-1** and **d_6-(*R*)-AS-7** in L-glutamate uptake mediated by EAAT2 in transfected COS-7 cells ... 17

3. Additional studies ... 18

Single crystal XRD ... 18

Crystal structure determination and analysis ... 18

***iv*PTZ seizure threshold test and PTZ-induced kindling model ... 18**

***In vivo* antinociception activity ... 22**

***In vitro* ADME-Tox assays ... 23**

4. Material and methods ... 34

Chemistry ... 34

General information. ... 34

Method for the preparation of deuterated benzylamine derivatives **A1**–**A4** ... 34

Method for the preparation of intermediates **(*R*)-1**–**(*R*)-6** and **(*R,S*)-1**–**(*R,S*)-6** ... 35

Procedure for the preparation of intermediates **(*R*)-7**–**(*R*)-12** and **(*R,S*)-7**–**(*R,S*)-12** ... 35

Procedure for the preparation of intermediates **(*R*)-13**–**(*R*)-19** and **(*R,S*)-13**–**(*R,S*)-19** ... 36

Procedure for the preparation of the final compounds 37
Animal studies **40**
General Information. 40
Pharmacokinetic studies 40
Bioanalytical method 40
Preparation of calibration standards 42
Sample preparation. 42
Pharmacokinetic data analysis. 42
Acute seizure models and rotarod test 42
6 Hz (32 mA) model and minimal motor impairment test in male and female C57 WT mice 42
6 Hz (32 mA) model 43
Minimal motor impairment test 43
ivPTZ seizure threshold test, grip strength test, and PTZ-induced kindling model in mice 43
Antinociceptive models and locomotor activity test 43
Glutamate uptake studies in COS-7 cell lines expressing EAAT1, EAAT2 and EAAT3 .... **44**
Cells and transfection 44
Dose response assays 44
Kinetic assays .... **Błąd! Nie zdefiniowano zakładki.**
Glutamate transporter studies in rat and mouse astrocytes **44**
Rat astrocte cultures 44
Mouse astrocyte cultures 45
Dose response uptake assays 45
Data analysis and statistics 44
Influence on transporter current in mouse astrocytes **44**
Brain Slice Preparation 45
Electrophysiology 45
Data analysis and statistics 45
Influence on transporter current in oocytes expressing EAAT2 .... **46**
Heterologous protein expression and electrophysiological study in Xenopus laevis oocytes 46
The electrophysiological recordings. 46
Data analysis and statistics. 46
***In vitro* ADME-Tox studies** **47**
Permeability. 47
Absorption in Caco-2 model 47
Protein binding analyses 47
Metabolic stability. 47
In vitro MLMs or HLMs metabolic stability 47
In vitro S9 metabolic stability. 47
In vitro hepatocyte metabolic stability. 48
Toxicity assays. 48
Phospholipidosis induction assay. 49
References **50**
UPLC/HRMS traces for final compounds **53**
^{1}H NMR and ^{13}C NMR spectra for final compounds **67**
Chiral SFC chromatograms **83**

1. Tables

Table S1. Crystal data and structure refinement results.

	d$_4$-(*R*)-AS-1	d$_4$-(*R*)-AS-7	d$_6$-(*R*)-AS-1	d$_6$-(*R*)-AS-7
**Crystal data**				
Chemical formula	$C_{14}H_{12}D_4N_2O_3$	$C_{14}H_{11}D_4FN_2O_3$	$C_{14}H_{10}D_6N_2O_3$	$C_{14}H_9D_6FN_2O_3$
M_r	264.31	282.31	266.32	284.32
Crystal system, space group	Monoclinic, *P* 2_1	Orthorhombic, *P* $2_12_12_1$	Monoclinic, *P* 2_1	Orthorhombic, *P* $2_12_12_1$
Temperature (K)	100(2)	100(2)	100(2)	100(2)
a, *b*, *c* (Å)	9.2927 (2), 16.4970 (3), 13.0928 (2)	9.6365 (1), 15.9737 (1), 17.6770 (1)	9.2933 (1), 16.4942 (2), 13.0967 (2)	9.6365 (1), 15.9767 (1), 17.6800 (1)
α, β, γ (°)	90, 96.620 (2), 90	90, 90, 90	90, 96.648 (1), 90	90, 90, 90
V (Å^3)	1993.77 (6)	2721.03 (4)	1994.04 (4)	2722.00 (4)
Z	2 (Z'=3)	4 (Z'=2)	2 (Z'=3)	4 (Z'=2)
Radiation type	Cu *K*α	Cu *K*α	Cu *K*α	Cu *K*α
μ (mm^{-1})	0.76	0.889	0.76	0.889
Crystal size (mm)	0.2 × 0.1 × 0.05	0.50 × 0.30 × 0.2	0.4 × 0.25 × 0.03	0.5 × 0.3 × 0.25
**Data collection**				
Diffractometer	XtaLAB Synergy, Dualflex, HyPix			
Absorption correction	Multi-scan *CrysAlis PRO* 1.171.42.100a (Rigaku Oxford Diffraction, 2023) Empirical absorption correction using spherical harmonics, implemented in SCALE3 ABSPACK scaling algorithm.			
T_{min}, T_{max}	0.355, 1	0.316, 1	0.633, 1	0.389, 1
No. of measured, independent and observed [$I > 2\sigma(I)$] reflections	72670, 8473, 7972	108256, 5894, 5783	71266, 8507, 8036	101292, 5889, 5783
R_{int}	0.0779	0.0758	0.0532	0.0594
Theta range for data collection	4.329 to 80.125°	3.730 to 80.465°	4.329 to 80.401°	3.729 to 80.060°
**Refinement results**				
$R[F^2 > 2\sigma(F^2)]$, $wR(F^2)$, *S*	0.0286, 0.0716, 1.062	0.0282, 0.0747, 1.072	0.0295, 0.0754, 1.062	0.0272, 0.0702, 1.053
No. of reflections	8473	5894	8507	5889
No. of parameters	578	414	602	430
No. of restraints	1	0	1	0
H-atom treatment	Mixed: independent and constrained	Mixed: independent and constrained	Mixed: independent and constrained	Mixed: independent and constrained
$\Delta\rho_{max}$, $\Delta\rho_{min}$ (e Å^{-3})	0.138, −0.163	0.213, −0.184	0.159, −0.160	0.227, −0.159
Absolute structure	Flack x determined using 3637 quotients [(I+)-(I-)]/[(I+)+(I-)]	Flack x determined using 2485 quotients [(I+)-(I-)]/[(I+)+(I-)	Flack x determined using 3689 quotients [(I+)-(I-)]/[(I+)+(I-)]	Flack x determined using 2474 quotients [(I+)-(I-)]/[(I+)+(I-)]
Absolute structure parameter	0.04 (6)	0.01 (4)	0.00 (5)	−0.05 (3)

Table S2. Pharmacokinetic parameters of **(*R*)-AS-1**, **d_4-(*R*)-AS-1**, **d_6-(*R*)-AS-1**, **d_9-(*R*)-AS-1** and **d_{11}-(*R*)-AS-1** in serum and brain tissue following *i.p.* administration of these compounds at a dose of 20 mg/kg and/or 40 mg/kg to male CD-1 mice.

Compound	Tissue	Dose [mg/kg]	t_{max} [min]	C_{max} [µg/mL(g)]	λ_z [min^{-1}]	$t_{1/2\lambda z}$ [min]	AUC_{0-t} [µg·min/mL(g)]	$AUC_{0-\infty}$ [µg·min/mL(g)]	V_z/F [L/kg]	CL/F [L/min/kg]	MRT [min]	Kp*
**(*R*)-AS-1**	Serum	20	30	46.60	0.015	47.31	7000.2	7000.89	0.19	0.003	112.18	0.30
	Brain		30	12.55	0.012	57.26	2133.99	2134.08			123.75	
	Serum	40	15	76.75	0.012	55.62	8172.95	8174.16	0.39	0.005	93.47	0.37
	Brain		15	21.87	0.016	75.11	3011.26	3014.90			105.34	
**d_4-(*R*)-AS-1**	Serum	20	15	36.60	0.009	80.23	9619.05	9641.41	0.24	0.002	173.61	0.38
	Brain		120	14.61	0.008	87.82	3626.38	3626.44			183.97	
	Serum	40	15	120.50	0.008	88.04	26473.86	26640.04	0.19	0.002	181.79	0.29
	Brain		30	27.93	0.007	103.39	7761.75	7762.42			192.77	
**d_5-(*R*)-AS-1**	Serum	40	30	62.76	0.01	69.50	7780.40	7785.46	0.52	0.005	95.18	0.42
	Brain		30	25.72	0.009	74.38	3252.46	3256.03			103.55	
**d_6-(*R*)-AS-1**	Serum	20	30	33.83	0.006	125.78	4959.15	5715.14	0.64	0.003	173.08	0.58
	Brain		30	15.75	0.006	124.93	2829.45	3305.98			184.252	
	Serum	40	60	60.97	0.007	97.52	13178.86	13179.30	0.43	0.003	158.36	0.62
	Brain		60	33.08	0.006	111.98	8189.80	8190.85			185.06	
**d_9-(*R*)-AS-1**	Serum	20	30	37.46	0.006	113.61	10733.91	10915.33	0.3	0.002	200.02	0.34
	Brain		30	11.89	0.007	103.84	3703.0	3703.37			208.09	
	Serum	40	15	75.05	0.006	118.0	19973.31	20299.55	0.34	0.002	183.07	0.36
	Brain		60	29.36	0.005	140.0	7367.63	7368.22			189.95	
**d_{11}-(*R*)-AS-1**	Serum	20	60	39.80	0.007	94.77	6792.25	7355.09	0.37	0.003	139.15	0.43
	Brain		60	15.08	0.007	103.67	2879.95	3193.55			165.04	
	Serum	40	30	59.73	0.008	88.19	14442.43	14442.69	0.35	0.003	171.57	1.15
	Brain		30	59.50	0.006	119.42	16670.98	16674.91			189.41	

Pharmacokinetic parameters were calculated from mean concentration versus time profiles: C_{max} – maximum serum/brain concentration; t_{max} – time to reach C_{max}; [illegible]$_z$ – terminal slope; $t_{1/2\lambda z}$ – terminal half-life; $AUC_{0-\infty}$ – area under the curve; V_z/F – volume of distribution; CL/F – clearance; MRT – mean residence time. *Brain-to-serum $AUC_{0-\infty}$ ratio ($AUC_{0-\infty}$ brain / $AUC_{0-\infty}$ serum).

Table S3. Pharmacokinetic parameters of **(*R*)-AS-7**, **d_4-(*R*)-AS-7** and **d_6-(*R*)-AS-7** in serum and brain tissue following *i.p.* and *p.o.* administration of these compounds at a dose of 20 mg/kg and 40 mg/kg to male CD-1 mice.

Compound	Tissue	Dose [mg/kg]	t_{max} [min]	C_{max} [μg/mL(g)]	λ_z [min^{-1}]	$t_{1/2\lambda z}$ [min]	AUC_{0-t} [μg·min/mL(g)]	$AUC_{0-\infty}$ [μg·min/mL(g)]	V_z/F [L/kg]	CL/F [L/min/kg]	MRT [min]	Kp*
**(*R*)-AS-7**	Serum	20	15	11.36	0.02	34.22	811.93	812.54	1.215	0.025	57.90	0.70
	Brain		15	7.58	0.02	34.50	564.47	564.967			62.20	
	Serum	40	15	46.93	0.021	33.50	3361.93	3363.98	0.57	0.01	52.83	0.41
	Brain		15	16.23	0.018	38.53	1368.32	1370.46			58.60	
	Serum (*p.o.*)	40	30	17.37	0.006	122.56	2108.53	2393.0	2.91	0.02	155.31	0.40
	Brain (*p.o.*)		30	8.20	0.006	121.90	803.15	883.05			132.64	
**d_4-(*R*)-AS-7**	Serum	20	30	26.57	0.007	96.0	3964.95	4285.43	0.652	0.005	141.73	0.99
	Brain		30	13.45	0.005	140.03	3322.35	4240.96			253.32	
	Serum	40	30	43.43	0.008	88.73	6737.80	7183.46	0.72	0.01	133.32	0.65
	Brain		30	26.57	0.007	95.78	4335.42	4703.70			148.65	
**d_6-(*R*)-AS-7**	Serum	20	15	18.97	0.007	99.73	3312.12	3615.74	0.807	0.006	154.28	1.23
	Brain		30	12.28	0.004	157.83	3346.32	4454.18			273.38	
	Serum	40	30	37.63	0.006	112.02	6524.33	7347.14	0.88	0.01	163.84	0.56
	Brain		30	20.78	0.006	113.19	3629.96	4122.65			172.05	
	Serum (*p.o.)*	40	30	19.20	0.006	122.55	4154.50	4834.99	1.45	0.01	186.54	0.57
	Brain (*p.o.*)		120	11.78	0.005	146.67	2348.23	2750.27			192.49	

Pharmacokinetic parameters were calculated from mean concentration versus time profiles: C_{max} – maximum serum/brain concentration; t_{max} – time to reach C_{max}; λ_z – terminal slope; $t_{1/2z}$ – terminal half-life; $AUC_{0-\infty}$ – area under the curve; V_z/F – volume of distribution; CL/F – clearance; MRT – mean residence time. *Brain-to-serum $AUC_{0-\infty}$ ratio ($AUC_{0-\infty}$ brain / $AUC_{0-\infty}$ serum). PK parameters after oral administration (*p.o.*) have been marked in green.

Table S4. Percent change (%Δ) in selected pharmacokinetic parameters of deuterated analogs, relative to parent ***(R)*-AS-1**, in serum and brain tissues following intraperitoneal (*i.p.*) administration at doses of 20 mg/kg and 40 mg/kg in male CD-1 mice.

Compound	Tissue	Dose [mg/kg]	%Δ C_{max}	%Δ $t_{1/2\lambda z}$	%Δ $AUC_{0-\infty}$
**d_4-(*R*)-AS-1**	Serum (*i.p.*)	20	-21.5%	69.6%	37.7%
	Brain (*i.p.*)		16.4%	53.4%	69.9%
	Serum (*i.p.*)	40	57.0%	58.3%	225.9%
	Brain (*i.p.*)		27.7%	37.7%	157.5%
**d_5-(*R*)-AS-1**	Serum (*i.p.*)	40	-18.2%	25.0%	-4.8%
	Brain (*i.p.*)		17.6%	-1.0%	8.0%
**d_6-(*R*)-AS-1**	Serum (*i.p.*)	20	-27.4%	165.9%	-18.4%
	Brain (*i.p.*)		25.5%	118.2%	54.9%
	Serum (*i.p.*)	40	-20.6%	75.3%	61.2%
	Brain (*i.p.*)		51.3%	49.1%	171.7%
**d_9-(*R*)-AS-1**	Serum (*i.p.*)	20	-19.6%	140.1%	55.9%
	Brain (*i.p.*)		-5.3%	81.3%	73.5%
	Serum (*i.p.*)	40	-2.2%	112.2%	148.3%
	Brain (*i.p.*)		34.3%	86.4%	144.4%
**d_{11}-(*R*)-AS-1**	Serum (*i.p.*)	20	-14.6%	100.3%	5.1%
	Brain (*i.p.*)		20.2%	81.1%	49.6%
	Serum (*i.p.*)	40	-22.2%	58.6%	76.7%
	Brain (*i.p.*)		172.1%	59.0%	453.1%

Percent change (Δ%) was calculated using the formula: $\Delta\% = ((X_{deut} - X_{ref}) / X_{ref}) \times 100\%$, where X_{deut} is the value for the deuterated analog and X_{ref} is the corresponding value for the reference compound (***(R)*-AS-1**), matched by tissue and dose. A positive value indicates an increase, and a negative value indicates a decrease in the parameter compared to the non-deuterated reference.

Table S5. Percent change (%Δ) in selected pharmacokinetic parameters of deuterated analogs, relative to **(*R*)-AS-7**, in serum and brain tissue following intraperitoneal (*i.p.*) or oral (*p.o.*) administration at doses of 20 mg/kg and 40 mg/kg in male CD-1 mice.

Compound	Tissue	Dose [mg/kg]	%Δ C_{max}	%Δ $t_{1/2\lambda z}$	%Δ $AUC_{0-\infty}$
**d_4-(*R*)-AS-7**	Serum (*i.p.*)	20	133.9%	180.5%	427.4%
	Brain (*i.p.*)		77.4%	305.9%	650.6%
	Serum (*i.p.*)	40	-7.5%	164.9%	113.5%
	Brain (*i.p.*)		63.7%	148.6%	243.2%
**d_6-(*R*)-AS-7**	Serum (*i.p.*)	20	67.0%	191.4%	345.0%
	Brain (*i.p.*)		62.0%	357.5%	688.4%
	Serum (*i.p.*)	40	-19.8%	234.4%	118.4%
	Brain (*i.p.*)		28.0%	193.8%	200.8%
	Serum (*p.o.)*	40	10.5%	-0.01%	102.0%
	Brain (*p.o.*)		43.7%	20.3%	211.5%

Percent change (Δ%) was calculated using the formula: $\Delta\% = ((X_{deut} - X_{ref}) / X_{ref}) \times 100\%$, where X_{deut} is the value for the deuterated analog and X_{ref} is the corresponding value for the reference compound (**(*R*)-AS-7**), matched by tissue, dose, and route of administration. A positive value indicates an increase, and a negative value indicates a decrease in the parameter compared to the non-deuterated reference.

Table S6. Antiseizure activity in the *sc*PTZ model following *i.p.* administration in male CD-1 mice.

**Compound**	**ED_{50} *sc*PTZ [mg/kg]**		**TD_{50} rotarod [mg/kg]**		**PI (TD_{50}/ED_{50}) [b]**	
	**Time point [a]**		**Time point [a]**		**Time point [a]**	
	**0.5 h**	**2.0 h**	**0.5 h**	**2.0 h**	**0.5 h**	**2.0 h**
**(*R*)-AS-1**	30.6 (14.0–66.8)	>130	236.2 (225.7–247.1)	>300	7.7	<1.8
**d_4-(*R*)-AS-1**	33.5 (23.3–48.3)	83.5 (65.9–105.7)	176.7 (126.8–246.0)	>300	5.3	>2.1
**d_9-(*R*)-AS-1**	36.3 (24.7–53.1)	85.6 (57.1–128.4)	165.4 (131.4–208.2)	>300	4.5	>1.9

Values in parentheses are 95% confidence intervals. [a] Pretreatment time; [b] Protective index (TD_{50}/ED_{50}) in the

2. Figures

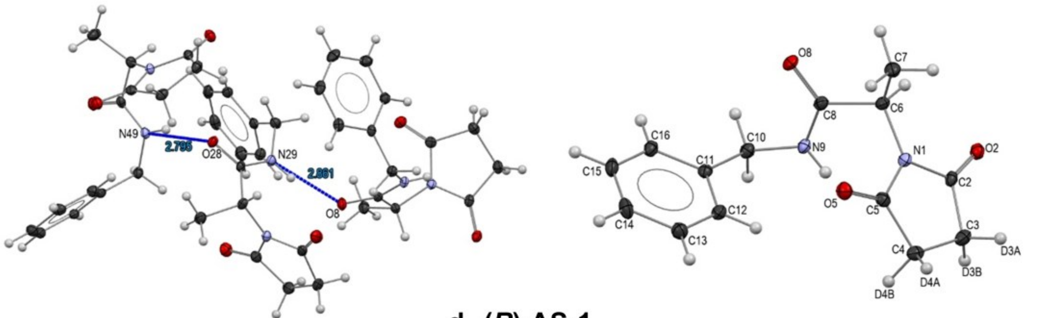

d$_4$-(*R*)-AS-1

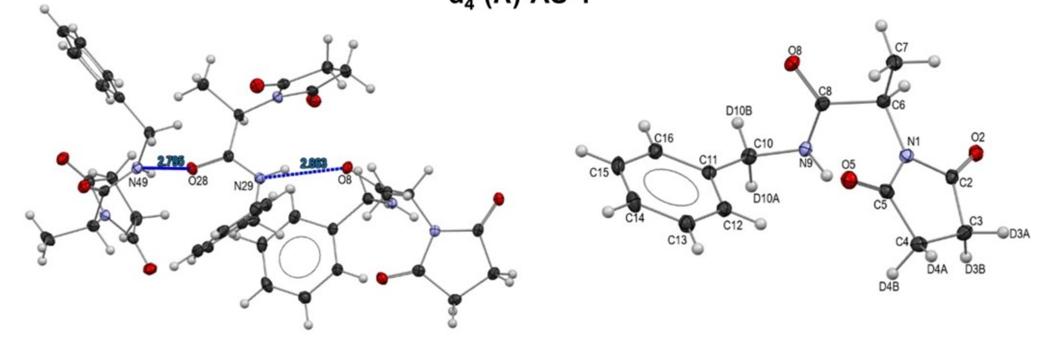

d$_6$-(*R*)-AS-1

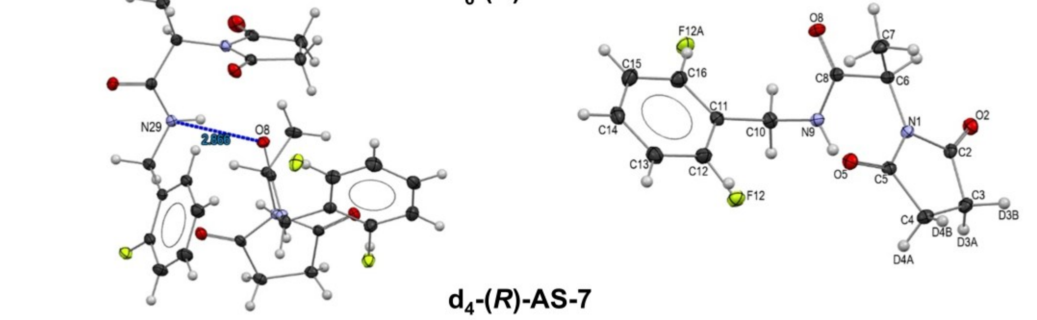

d$_4$-(*R*)-AS-7

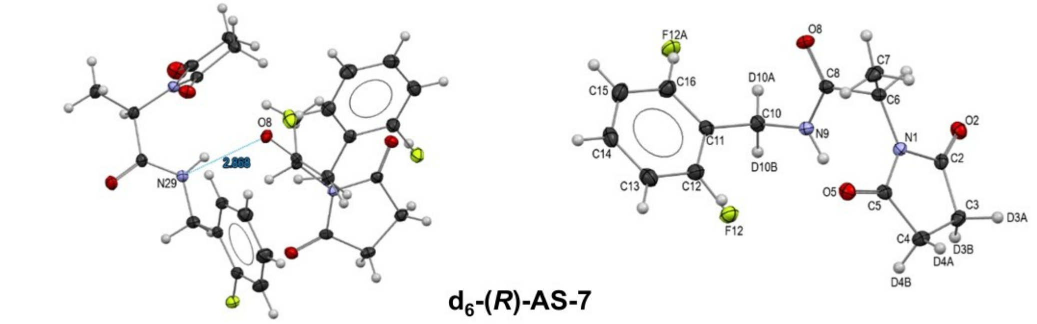

d$_6$-(*R*)-AS-7

Figure S1. Asymmetric units (left column) and a single molecule geometry (right column) presenting the numbering scheme for the enantiopure crystal structures of d$_4$-(*R*)-AS-1, d$_6$-(*R*)-AS-1, d$_4$-(*R*)-AS-7 and d$_6$-(*R*)-AS-7. In asymmetric units of the **d$_4$-(*R*)-AS-1** and **d$_6$-(*R*)-AS-1**, the three independent molecules are interacting *via* N-H...O hydrogen bond, which forms chains propagating in [001] direction. The asymmetric units of **d$_4$-(*R*)-AS-7** and **d$_6$-(*R*)-AS-7 d$_4$-(*R*)-AS-7** and **d$_6$-(*R*)-AS-7** consist of two molecules forming similar N-H...O interaction chain motif along the [001] axis. One molecule of the asymmetric unit for the fluoro- derivatives **d$_4$-(*R*)-AS-7** and **d$_6$-(*R*)-AS-7** exhibits the positional disorder of the F-substituent with the site occupancies approx. equal to 55% and 45% for F12 and F12A, respectively.

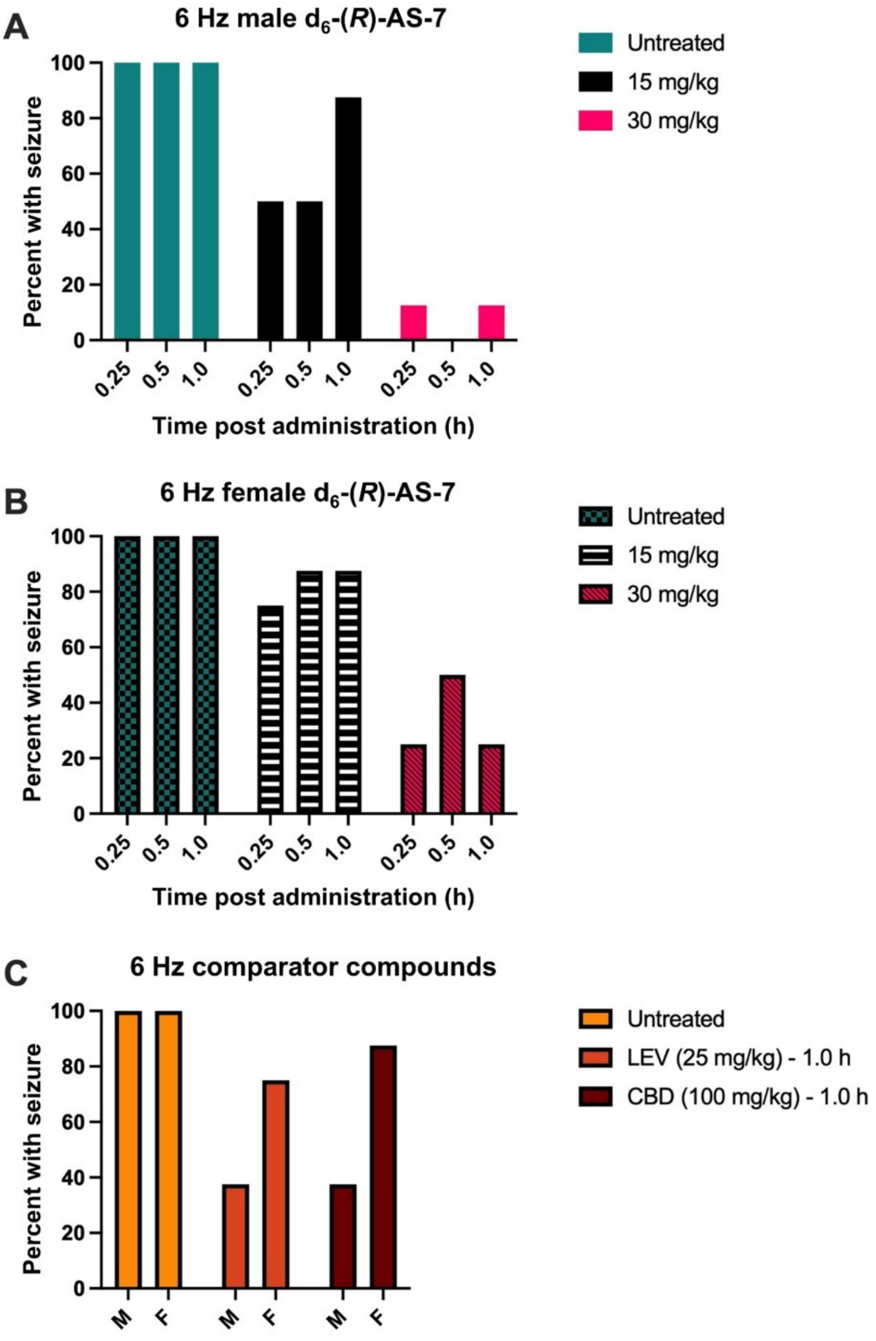

Figure S2. Antiseizure activity of d_6-(*R*)-AS-7 in the 6 Hz (32 mA) seizure model following *i.p.* administration in male (M) and female (F) C57BL/6J mice (conducted at University of Washington). (**A** and **B**) The investigational compound **d_6-(*R*)-AS-7** was administered to male and female C57BL/6J mice (aged 3-4 months) and mice were subsequently tested for time- and dose-related protection from evoked seizures in the 6 Hz 32 mA test. Untreated historical control mice in the same age and strain are included for comparison. There was a time- and dose-related reduction in the presentation of evoked seizures in both male and female C57BL/6J mice treated via the *i.p.* route with **d_6-(*R*)-AS-7** (15 and 30 mg/kg). (**C**) As an additional confirmation of the comparative pharmacological activity of **d_6-(*R*)-AS-7** in this mouse seizure model, two prototype antiseizure medicines (ASMs), levetiracetam (LEV) and cannabidiol (CBD), were administered at single *i.p.* doses and tested for antiseizure activity in parallel with the **d_6-(*R*)-AS-7**-treated mice, with the ASMs administered and tested at their known time of peak anticonvulsant activity.

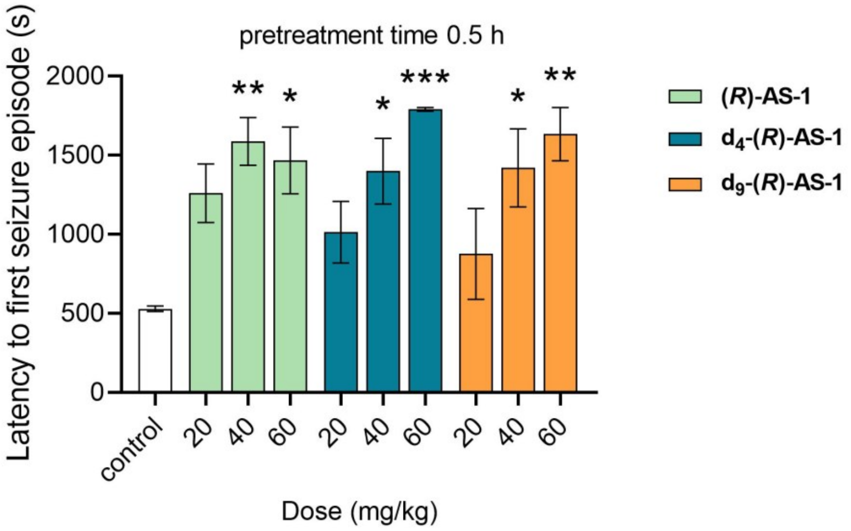

Figure S3. **Effects of the deuterated analogues d_4-(*R*)-AS-1, d_9-(*R*)-AS-1, and parent (*R*)-AS-1 on latency time to first clonus in the *sc*PTZ test (male CD-1 mice, *i.p.*) in the pretreatment time 0.5 h**. Data are shown as mean ± SEM (n=5-6 animals). Statistical significance was evaluated by a one-way ANOVA, followed by *Dunnet's post hoc* test: *p < 0.05, **p < 0.01, ***p < 0.001 (GraphPad Prism 8.0.1).

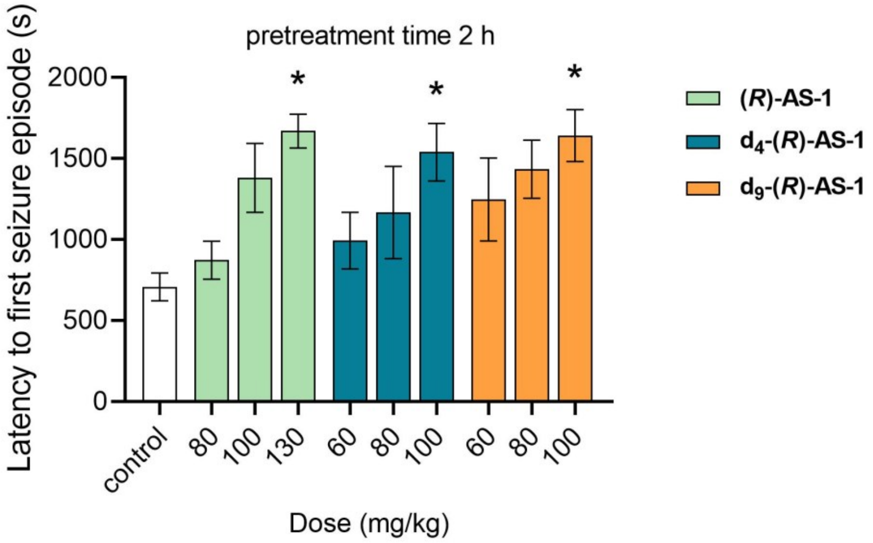

Figure S4. **Effects of the deuterated analogues d_4-(*R*)-AS-1, d_9-(*R*)-AS-1, and parent (*R*)-AS-1 on latency time to first clonus in the *sc*PTZ test (male CD-1 mice, *i.p.*) in the pretreatment time 2 h**. Data are shown as mean ± SEM (n=5-6 animals). Statistical significance was evaluated by a one-way ANOVA, followed by *Dunnet's post hoc* test: *p < 0.05 (one-way ANOVA, followed by Dunnett's *post hoc* test (GraphPad Prism 8.0.1).

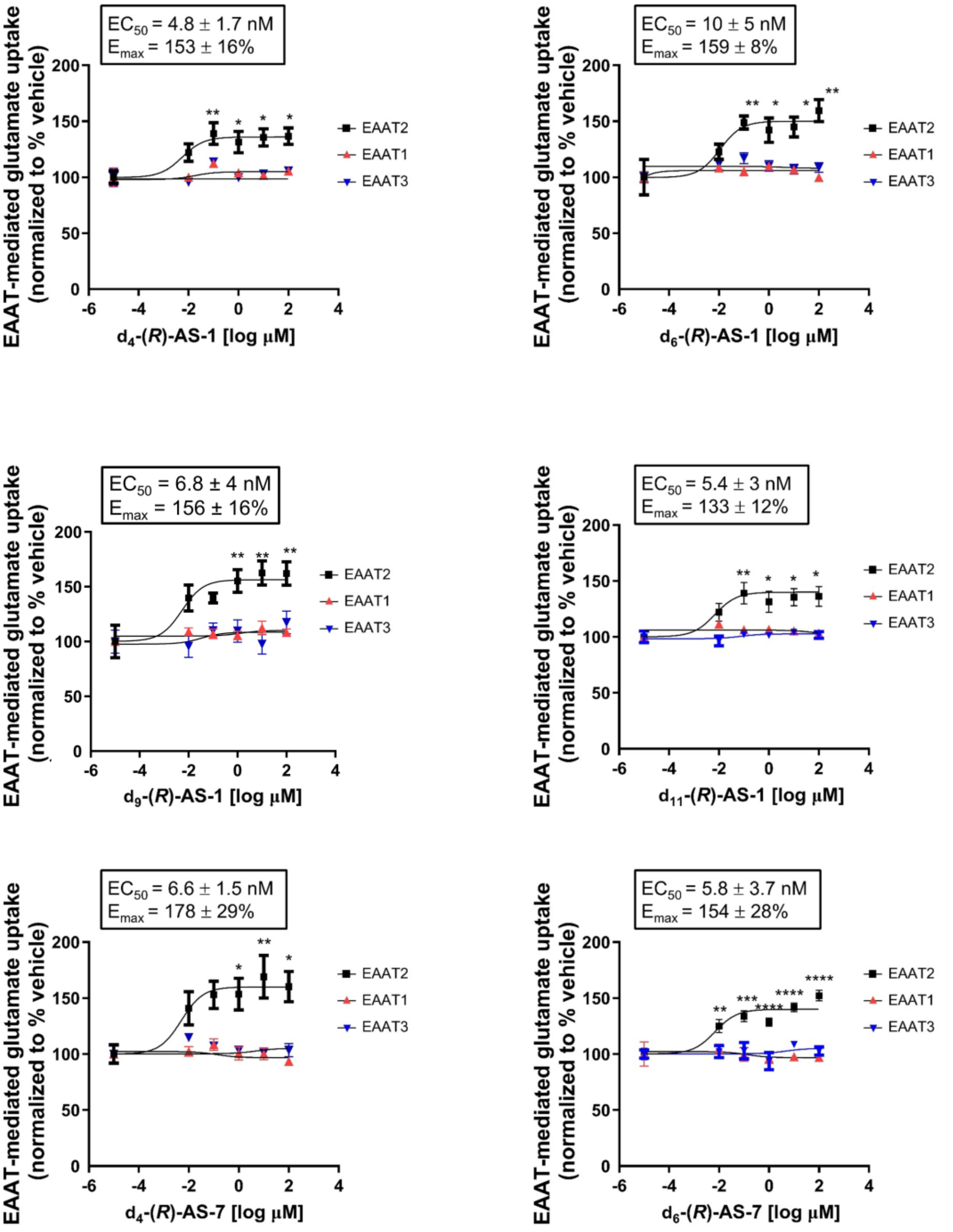

Figure S5. **Glutamate uptake mediated by EAAT2, EAAT1 and EAAT3 in COS-7 cells transiently transfected with CMV, EAAT1, EAAT2 or EAAT3**. Two days after the transfection, cells were pre-incubated for 10 min at 37 °C with either a vehicle or a range of concentrations of the indicated compounds (0.1 nM–100 µM). 50 nM of ^{3}H-L-glutamate (final concentration) was added, and 10 min later reactions were terminated. Dose response curves were plotted after subtraction of the background (CMV-transfected cells) and normalization of the effects to vehicle-control. Results are expressed as mean ± SEM from 3 to 6 independent experiments performed in technical triplicate. Statistical analysis (GraphPad Prism 8.0.1): *p < 0.05, **p < 0.01, ***p < 0.001, ****p < 0.0001 *vs.* vehicle (one Way ANOVA followed by Dunnett's *post hoc* test. EC_{50} – the concentration at which compound exerts 50% of its maximal effect; E_{max} – maximal glutamate uptake efficacy. Studies performed in the Department of Pharmacology and Physiology, Drexel University College of Medicine, Philadelphia, PA, 19102, United States.

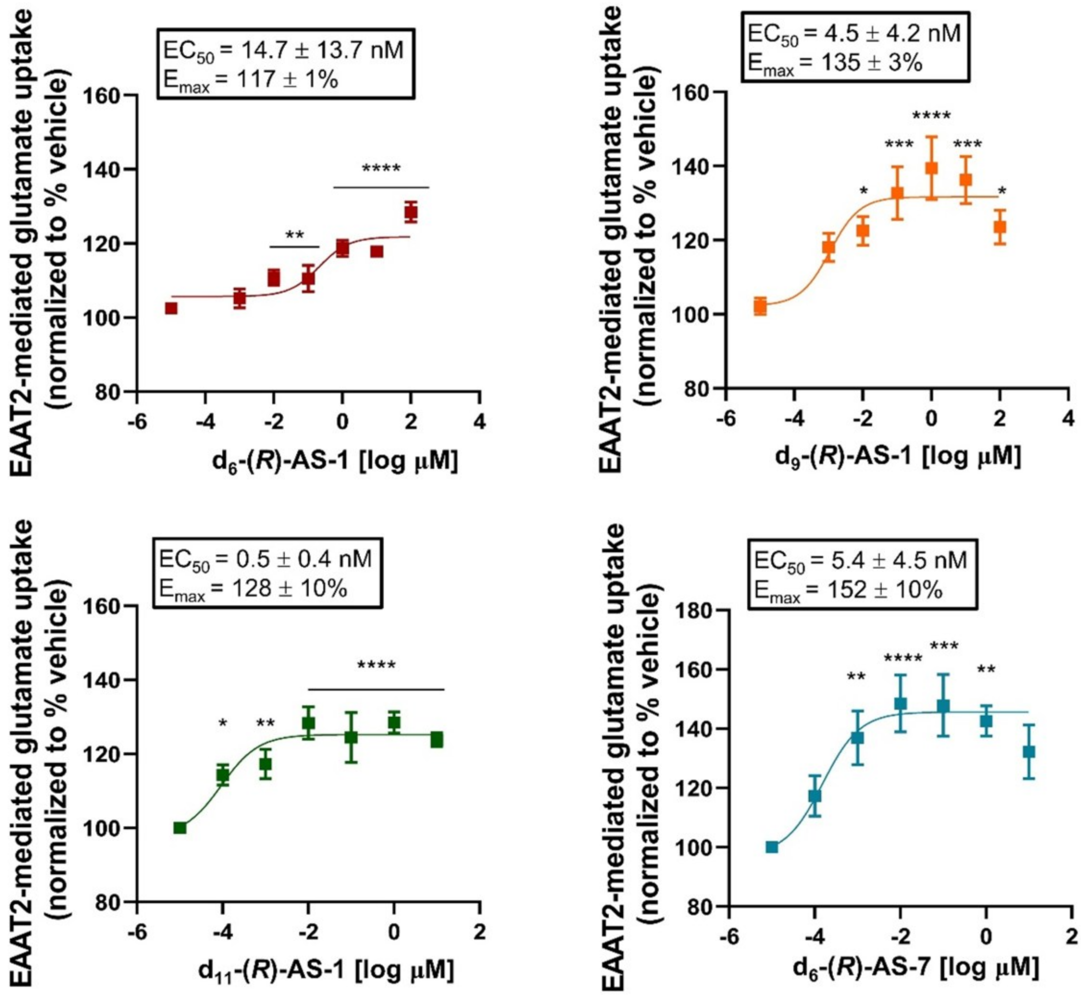

Figure S6. EAAT2-mediated glutamate uptake in COS-7 cells for representative deuterium-containing compounds (replication studies). After a 10 min pre-incubation with either a vehicle or compound at concentrations ranging from 0.1 nM–100 µM, uptake reactions were started by the addition of 50 nM (final concentration) of ^{3}H-L-glutamate. Results were normalized to a percentage of control (EAAT2) and expressed as mean ± SEM from three to six independent experiments performed in technical triplicate. Statistical analysis (GraphPad Prism 8.0.1): **$p < 0.01$, ***$p < 0.001$, ****$p < 0.0001$ *vs.* vehicle (one-way ANOVA followed by Dunnett's *post hoc* test). EC$_{50}$ – the concentration at which compound exerts 50% of its maximal effect; E$_{max}$ – maximal glutamate uptake efficacy. Studies performed in the Department of Pharmacology, Maj Institute of Pharmacology, Polish Academy of Sciences (PAS), Krakow, Poland.

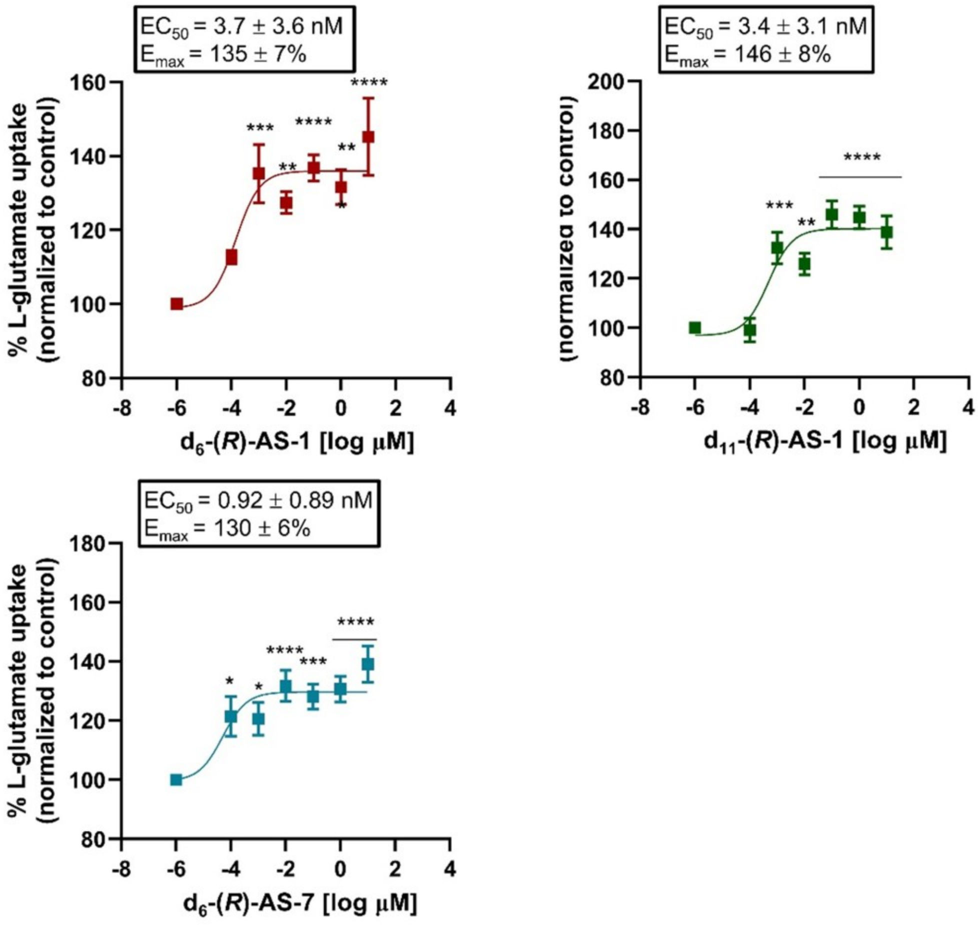

Figure S7. **Representative deuterium-containing compounds enhance glutamate uptake in mouse astrocytes**. Astrocytes were pre-incubated for 10 min with either a vehicle or compound at concentrations ranging from 0.01 nM–100 µM. Results were normalized to a percentage of control (vehicle) and expressed as mean ± SEM from three to six independent experiments performed in technical triplicate. Statistical analysis (GraphPad Prism 8.0.1): *p < 0.05, **p < 0.001, ***p < 0.001, ****p < 0.0001 *vs.* control (one-way ANOVA followed by Dunnett's *post hoc* test. EC_{50} – the concentration at which compound exerts 50% of its maximal effect; E_{max} – maximal glutamate uptake efficacy. Studies performed in the Department of Pharmacology, Maj Institute of Pharmacology Polish Academy of Sciences, Krakow, Poland.

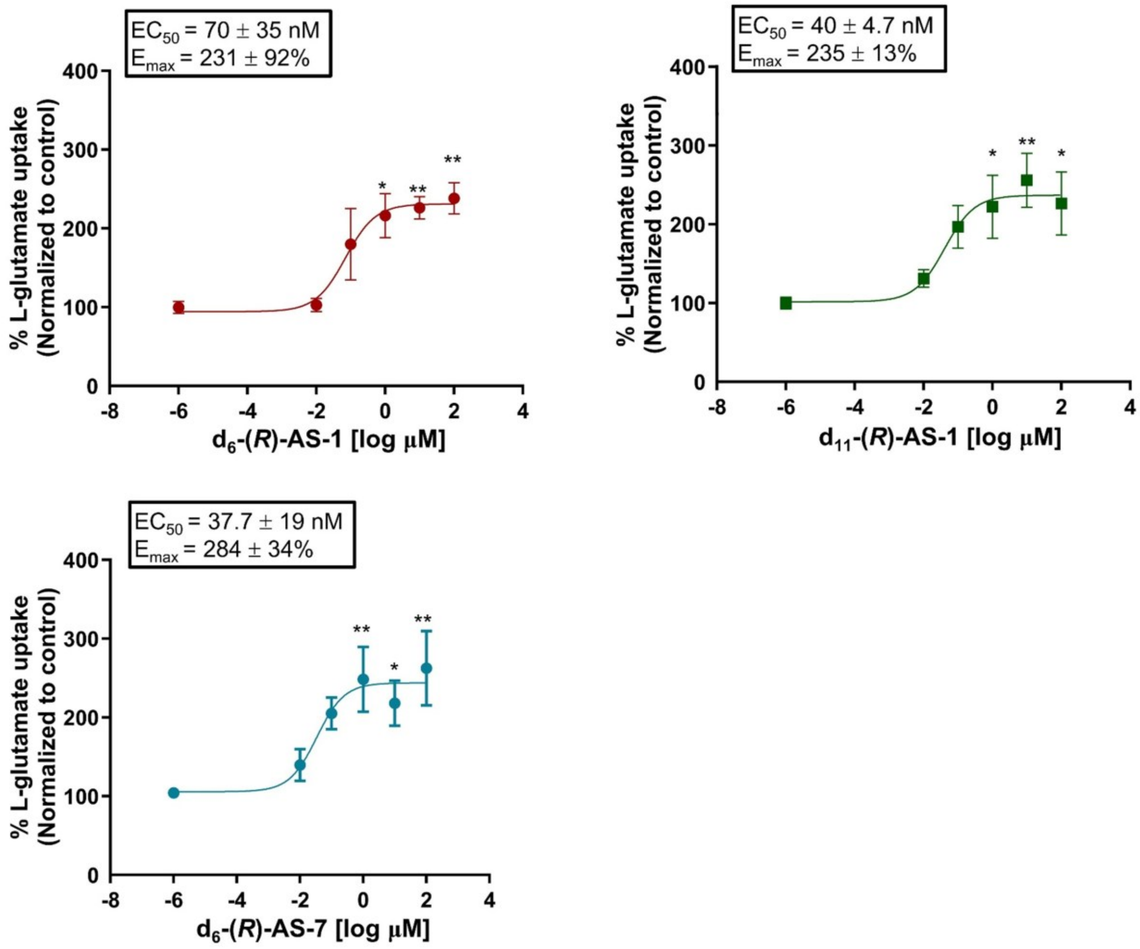

Figure S8. Representative deuterium-containing compounds enhance glutamate uptake rat astrocytes. Cells were pre-incubated for 10 min with either a vehicle or compound at concentrations ranging from 0.01 nM to 100 µM at 37°C and 10 min with 50 nM ^{3}H-Lglutamate. Nonspecific uptake (background) was obtained in the presence of EAAT inhibitor DL-TBOA (10 µM). EC_{50}s and efficacies are indicated. Results were normalized to a percentage of control (vehicle) and expressed as mean ± SEM from three to six independent experiments performed in technical triplicate. Statistical analysis (GraphPad Prism 8.0.1): *$p < 0.05$, **$p < 0.01$ *vs*. control (one-way ANOVA followed by Dunnett's *post hoc* test. EC_{50} – the concentration at which compound exerts 50% of its maximal effect; E_{max} – maximal glutamate uptake efficacy. Studies performed in the Department of Pharmacology and Physiology, Drexel University College of Medicine, Philadelphia, PA, 19102, United States.

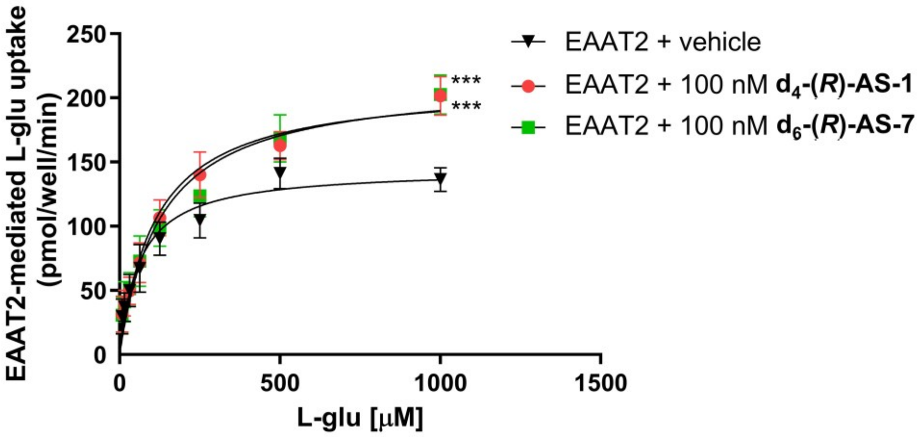

EAAT2	Vehicle	100 nM d_4-(*R*)-AS-1	100 nM d_6-(*R*)-AS-7
V_{max} (pmol/well/min)	144.7 ± 24.2	210.8 ± 32.7***	214.6 ± 43.5***
K_m (μM)	62.8 ± 41.9	112.8 ± 59.9	129.1 ± 87.8

Figure S9. **Kinetic analyses of the effect of d_4-(*R*)-AS-1 and d_6-(*R*)-AS-7 in L-glutamate uptake mediated by EAAT2 in transfected COS-7 cells**. Cells were preincubated with vehicle or 100 nM compounds. V_{max} and K_m values are indicated in the table; K_m was not statistically different between the conditions, of compounds, whereas V_{max} is increased in the presence of 100 nM compounds. Results are expressed in pmol/well/min (V_{max}) and μM (K_m) and expressed as mean ± SEM of three independent experiments. Statistical analysis (GraphPad Prism 8.0.1): ***$p < 0.001$ *vs.* vehicle (one-way ANOVA followed by Dunnett's multiple comparisons *post hoc* test).

3. Additional studies

Single crystal XRD

Crystal structure determination and analysis

The (*R*)-enantiomers of the selectively deuterated compounds **d_4-(*R*)-AS-1**, **d_6-(*R*)-AS-1** and their fluorinated derivatives **d_4-(*R*)-AS-7** and **d_6-(*R*)-AS-7** were crystallized by the slow-evaporation method from solution at ambient conditions. X-ray diffraction data for single, plate/prism-shaped crystals were collected using XtalLAB Synergy-S four circle diffractometer with a mirror monochromator and a microfocus CuKα radiation source (λ = 1.5418 Å) to ensure observation of anomalous diffraction effect for absolute structure confirmation. Additionally, the diffractometer was equipped with the CryoStream cryostat system allowing low-temperature experiments, performed at 100(2) K. The obtained data sets were processed with CrysAlisPro software.[1] The phase problem was solved with direct methods using SIR2014.[2] Parameters of the obtained models were refined by full-matrix least-squares on F^2 using SHELXL-AS-114/6.[3] Calculations were performed using WinGX integrated system (ver. 2014.1).[3] Figures were prepared with Mercury 4.0 software.[4]

All non-hydrogen atoms were refined anisotropically. All hydrogen atoms attached to carbon atoms were positioned with the idealized geometry and refined using the riding model with the isotropic displacement parameter $U_{iso}[H] = 1.2\ U_{eq}[C]$ for all but methyl group, for which $U_{iso}[H] = 1.5\ U_{eq}[C]$ was applied. Hydrogen atoms bound to nitrogen atoms of the amide moiety as well as position of the pre-defined deuterium atoms were located on the Fourier difference map and refined with no restraints. The absolute configuration for the analyzed compounds has been confirmed by the anomalous dispersion phenomenon, based on Parsons method (intensity quotients).[5] Crystal data and refinement results are shown in **Table S1**.

Crystallographic data have been deposited with the Cambridge Crystallographic Data Centre as supplementary publication nos.: CCDC 2400562 (**d_4-(*R*)-AS-1**), CCDC 2400563 (**d_6-(*R*)-AS-7**), CCDC 2400564 (**d_4-(*R*)-AS-7**) and CCDC 2400565 (**d_6-(*R*)-AS-1**). Copies of the data can be obtained, free of charge, on application to CCDC, 12 Union Road, Cambridge CB2 1EZ, UK (e-mail: deposit@ccdc.cam.ac.uk).

All the reported crystal structures of the deuterated compounds (irrespective of the deuterium substitution level) are isostructural with the previously reported protio forms confirming the negligible influence of the deuterium presence on the molecular properties in terms of inter- and intramolecular interactions propensity.[6]

The enantiopure crystal of **d_4-(*R*)-AS-1** and **d_6-(*R*)-AS-1** crystallize in the monoclinic (non-centrosymmetric space group $P2_1$) and their fluorinated derivatives **d_4-(*R*)-AS-7** and **d_6-(*R*)-AS-7** in orthorhombic (non-centrosymmetric space group $P2_12_12_1$) crystal systems. The studied crystal structures are stabilized mainly by N-H...O hydrogen bonds, formed between peptide fragments of neighboring molecules (**Figure S1**). This interaction creates chain motifs, propagating in [001] direction. Additionally, several weak intermolecular interactions are observed.

*iv*PTZ seizure threshold test and PTZ-induced kindling model

The timed *iv*PTZ test was employed to evaluate the influence of **d_4-(*R*)-AS-1** on the thresholds for three different types of seizures. Acute administration of **d_4-(*R*)-AS-1** at the dose of 50 mg/kg caused a slight increase of the thresholds for the onset of the first myoclonic twitch and generalized clonic seizure with loss of righting reflex ($p < 0.01$ and $p < 0.05$, respectively) but did not affect the threshold for tonic forelimb extension (**Figure S10**).

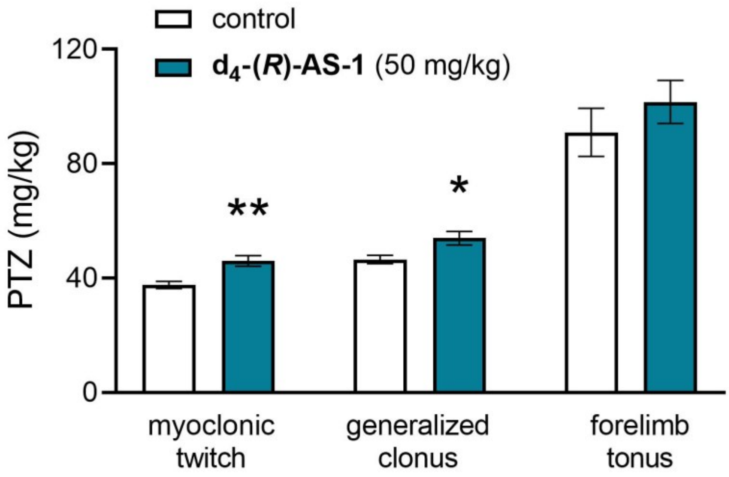

Figure S10. **Effect of acute treatment with d_4-(*R*)-AS-1 on seizure thresholds in the *iv*PTZ seizure test in mice**. Compound was administered *ip* at the dose of 50 mg/kg, 30 min before seizure induction. Control animals received vehicle (1% Tween). Data are presented as means (mg/kg PTZ) ± SEM (n = 9–12 male Swiss mice). Statistical analysis: *$p < 0.05$, **$p < 0.01$ vs. control group, unpaired Student's t test (GraphPad Prism 8.0.1).

In the *iv*PTZ test, forelimb tonic extension is usually followed by hindlimb tonus. Although **d_4-(*R*)-AS-1** did not raise the threshold for forelimb tonus, it diminished the occurrence of tonic hindlimb extension in 6 out of 10 mice ($p < 0.05$ vs. control group). In addition, **d_4-(*R*)-s71** at 50 mg/kg did not alter neuromuscular strength, as assessed in the grip-strength test (**Figure S11**), confirming favorable safety profile of the compounds.

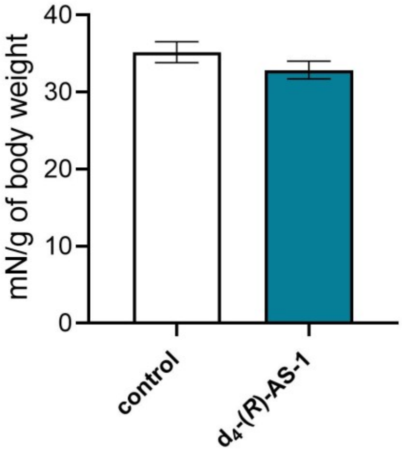

Figure S11. **Effect of acute treatment with d_4-(*R*)-AS-1 on neuromuscular strength assessed in the grip-strength test in mice.** Compound was administered *i.p.* at the dose of 50 mg/kg, 30 min before the test. Control animals received vehicle (1% Tween). Data are presented as means of grip strength expressed in mN/g of body weight ± SEM (n = 10 male Swiss mice). Statistical analysis: unpaired Student's t test (GraphPad Prism 8.0.1

Next, the antiseizure potential of **d_4-(*R*)-AS-1** was investigated in the PTZ-induced kindling model in mice (**Figure S12**). After repeated injections of PTZ, the mean seizure severity score in the control group increased from 1.00 ± 0.00 (on the first day of kindling) to 4.12 ± 0.40 (on the last day of kindling). Compound **d_4-(*R*)-AS-1** given at the highest dose tested (80 mg/kg) significantly suppressed kindling development ($p < 0.05$).

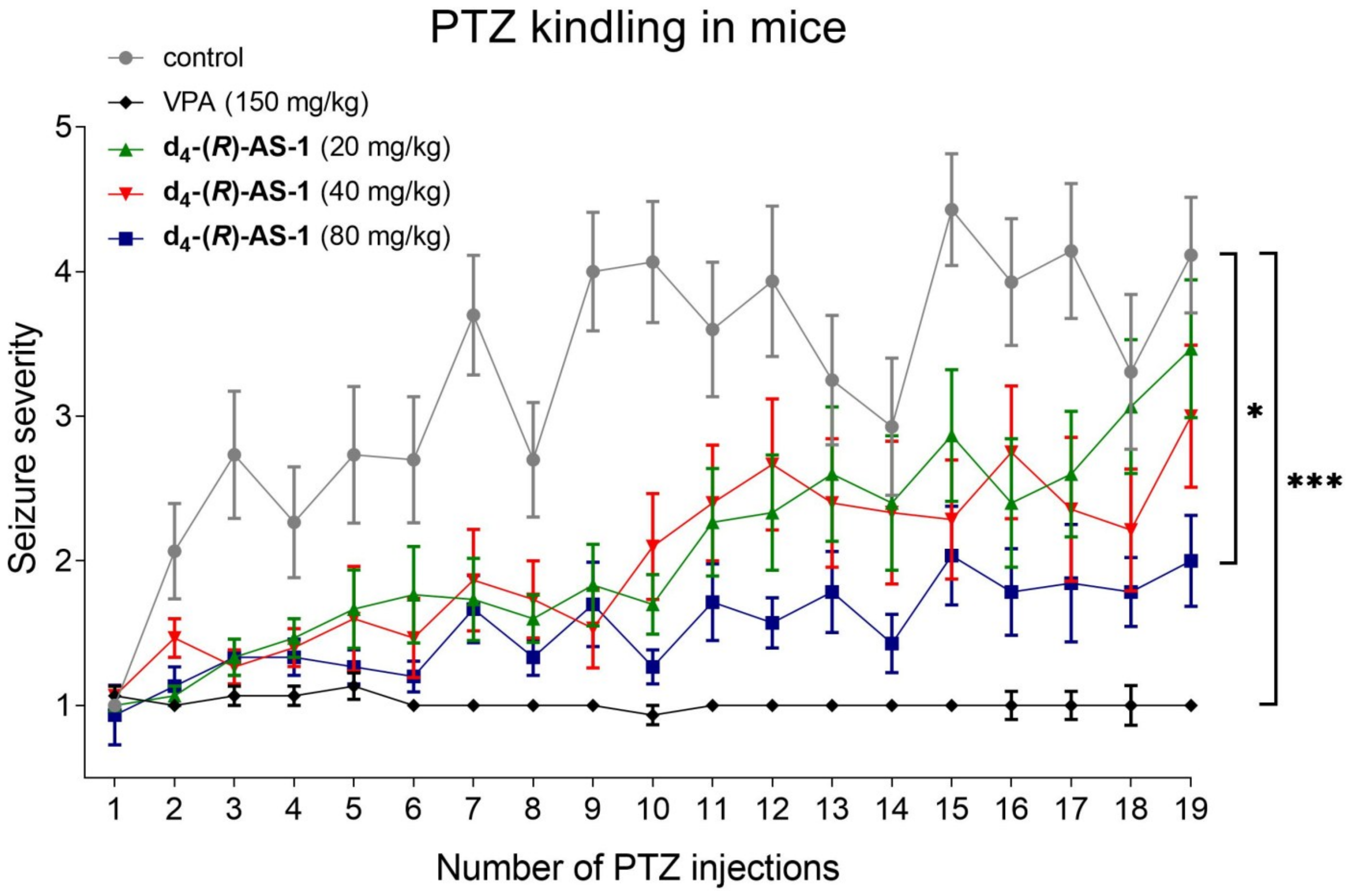

Figure S12. **Effect of d_4-(*R*)-AS-1 on the progression of the PTZ-induced kindling in mice**. Compound **d_4-(*R*)-AS-1**, valproate (VPA, positive control), or vehicle were administered *i.p.* every 24 hours. Seizures were induced by *i.p.* administration of PTZ (40 mg/kg) three times a week. On the day of kindling, **d_4-(*R*)-AS-1** VPA, or vehicle were given 30 min before PTZ administration. Data are presented as means of seizure severity ± SEM (n = 13–15 male Swiss mice). Statistical analysis: *p < 0.05, ***p < 0.001 vs. control group (mixed effects model for repeated measures followed by Bonferroni's *post hoc* test (GraphPad Prism 8.0.1).

The mean seizure severity score in animals pre-treated with **d_4-(*R*)-AS-1** was 0.93 ± 0.21 on the first day of kindling and 2.00 ± 0.31 on the last day. Lower doses (20 and 40 mg/kg) were ineffective, whereas valproic acid (VPA, positive control) at dose of 150 mg/kg completely inhibited kindling progression ($p < 0.001$). No significant alterations in spontaneous locomotor activity, anxiety-related, and stress-coping behavior in kindled animals were observed (**Figure S13**).

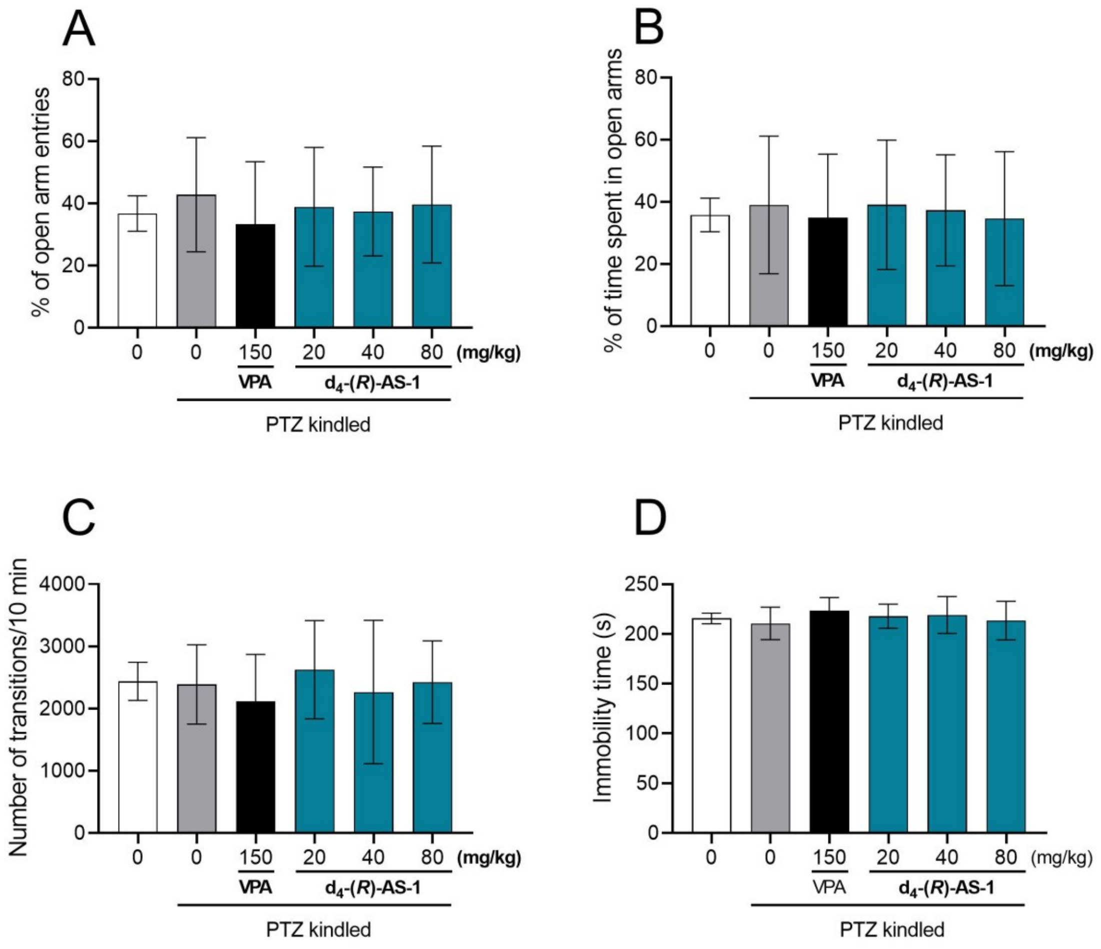

Figure S13. **Effect of d_4-(*R*)-AS-1 on (A) the percentage of time spent in the open arms in the elevated plus maze test, (B) the percentage of open arm entries in the elevated plus maze test, (C) spontaneous locomotor activity (D), and total immobility duration in the forced swim tests in PTZ kindled mice**. Compound **d_4-(*R*)-AS-1**, valproate (VPA, positive control), or vehicle were administered *i.p.* every 24 hours. Seizures were induced by *i.p.* administration of PTZ (40 mg/kg) three times a week. On the day of kindling, **d_4-(*R*)-AS-1** VPA, or vehicle were given 30 min before PTZ administration. Behavioral tests were performed 24 h after the last PTZ injection. Data are presented as means ± SEM (n=10–15 male Swiss mice). Statistical analysis: one-way ANOVA (GraphPad Prism 8.0.1).

The PTZ kindling model, in contrast to acute seizure tests (such as MES, 6 Hz, *sc*PTZ or *iv*PTZ test), is a chronic model in which repeated administration of a subconvulsive dose of PTZ increases seizure susceptibility and evokes some permanent changes in brain circuitry, resembling those occurring in human epilepsy.[7] Although this model mimics some aspects of epileptogenesis (i.e. the process of epilepsy development), it cannot be concluded that **d_4-(*R*)-AS-1** has anti-epileptogenic potential. The compound was given 0.5 h before seizure induction and thereby the anti-kindling effect may be more likely related to its acute antiseizure properties.

In vivo antinociception activity

Glutamate regulates multiple levels of pain transmission, including the dorsal horn of the spinal cord and the brain. It serves as the primary neurotransmitter for nociceptive sensory neurons that terminate in the spinal dorsal horn, where they synapse onto interneurons and spinothalamic tract neurons. Nociceptive stimuli are transmitted from the thalamus to the brain cortices involved in pain perception, including its emotional components, such as the somatosensory cortex, anterior cingulate cortex, and insular cortex.[8,9]

Novel deuterated analogs reported in this work could provide significant antinociception through a mechanism of action distinct from currently used analgesics. To test this hypothesis, we evaluated the effects of **d_4-(*R*)-AS-1** in various animal models of pain.

As shown in **Figure S14A**, the *i.p.* administration of **d_4-(*R*)-AS-1** significantly attenuated the nociceptive response in both phases of the formalin test, which is a widely used animal model for assessing both acute (phase I) and persistent inflammatory pain (phase II).[10] The ED_{50} values were 97.9 mg/kg and 71.5 mg/kg in phases I and II of the test, respectively. Moreover, **d_4-(*R*)-AS-1** attenuated the capsaicin-induced nociceptive response (**Figure S14B**) with an ED_{50} value of 17.5 mg/kg, indicating its potential to inhibit neurogenic pain resulting from the release of neuropeptides such as substance P and calcitonin gene-related peptide (CGRP) from nerve endings, leading to neurogenic inflammation and sensitization of peripheral nociceptors.[11]

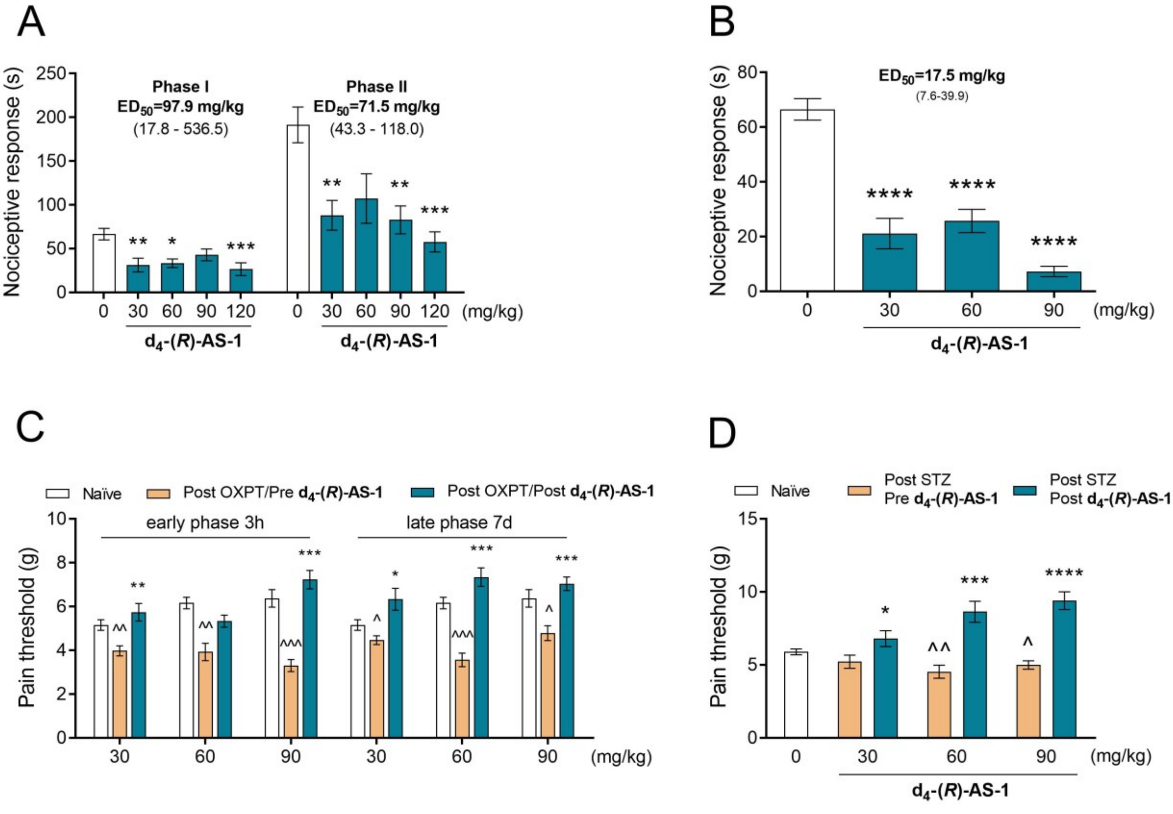

Figure S14. **The antinociceptive activity of d_4-(*R*)-AS-1 in the animal models of pain**. The compound was tested in the formalin test (**A**), capsaicin test (**B**), OXPT-induced (**C**) and STZ-induced (**D**) neuropathic pain models, where mechanical allodynia was measured using the electronic von Frey test (**C**, **D**). The results are presented as bar plots showing the mean ± SEM. Statistical analysis (GraphPad Prism 8.0.1): One-way ANOVA followed by Dunnett's *post hoc* test (**A**, **B**), * $p < 0.05$ ** $p < 0.01$, *** $p < 0.001$, **** $p < 0.0001$ when compared to vehicle-treated animals; two-way ANOVA followed by Tukey's *post hoc* test (**C**, **D**), * $p < 0.05$ ** $p < 0.01$, *** $p < 0.001$, **** $p < 0.0001$ when compared to OXPT- or STZ-treated animals; ^ $p < 0.05$, ^^ $p < 0.01$, ^^^ $p < 0.001$ when compared to naive animals, n = 8–10 male CD-1 mice per group.

Additionally, **d_4-(*R*)-AS-1** demonstrated potent antinociceptive activity in two neuropathic pain models of different origins: peripheral neuropathy induced by OXPT (**Figure S14C**) or STZ (**Figure S14D**). The OXPT-induced neuropathic pain model mimics chemotherapy-induced peripheral neuropathy (CIPN), resulting from structural and functional alterations in peripheral nerves.[12] In contrast, the STZ-induced neuropathic pain model is commonly used to study diabetic neuropathy.[13] In both models, OXPT or STZ single administration in naïve animals induced mechanical hyperalgesia and allodynia, characterized by a decreased pain threshold to mechanical stimuli. The administration of **d_4-(*R*)-AS-1** reversed these effects and even increased the pain threshold beyond the initial baseline observed in naïve animals, demonstrating its significant potential to control neuropathic pain of different origins.

Importantly, at the range of doses exhibiting analgesic activity, **d_4-(*R*)-AS-1** did not affect spontaneous locomotor activity (**Figure S15**). This provides evidence that the reduction in nociceptive responses was not due to **d_4-(*R*)-AS-1** induced sedation.

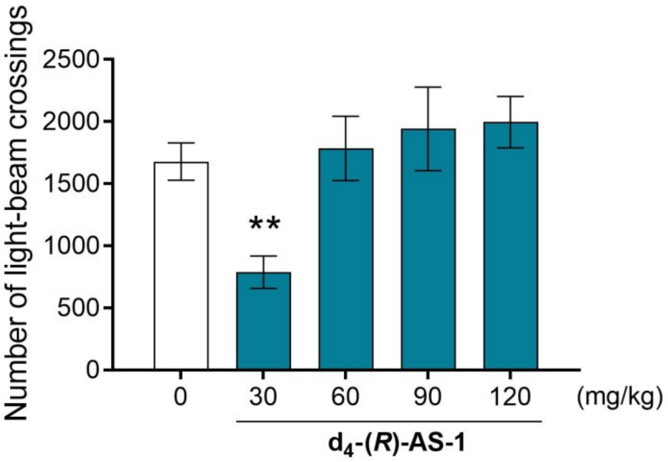

Figure S15. **Effect of d_4-(*R*)-AS-1 on the spontaneous locomotor activity**. Results are shown as number of light beam crossings during 30 min of observation beginning at 30 min after compound administration (*i.p.*). Each value represents the mean ± SEM (n=8-10 male CD-1 mice). Statistical analysis (GraphPad Prism 8.0.1): **$p < 0.01$ *vs*. vehicle-treated group (one-way ANOVA followed by Dunnett's *post hoc* test.

In vitro ADME-Tox assays

Compounds **d_{11}-(*R*)-AS-1** and **d_6-(*R*)-AS-7**, together with their corresponding non-deuterated prototypes, **(*R*)-AS-1** and **(*R*)-AS-7**, respectively, were examined *in vitro* to determine key ADME–Tox parameters. The results obtained are summarized in **Table S7**.

In general, PAMPA assay for determination of passive transport through biological membrane showed higher permeability coefficient (P_e) for all investigated compounds than recommended by manufacturer's guideline P_e value for permeable compound (1.5×10^{-6} cm/s).[14] However, the obtained P_e's were lower than P_e of the well-permeable reference compound (caffeine) (**Table S7**).

Table S7. The ADME-Tox parameters determined *in vitro*.

Compund	Caco-2 (A–B) $P_{app} \times 10^{-6}$ cm/s ± SD	Caco-2 (B–A) $P_{app} \times 10^{-6}$ cm/s ± SD	PAMPA permeability: $P_e \times 10^{-6}$ cm/s ± SD	Plasma protein binding f_b (%)	Effect on CYP3A4 % of control ± SD at 10 µM	Effect on CYP2D6 % of control ± SD at 10 µM	Effect on CYP2C9 % of control ± SD at 10 µM	HepG2 viability % of control ±SD at 100µM	SH-SY5Y viability % of control ± SD at 100 µM
**(*R*)-AS-1***	ND	ND	1.98±0.6	<80	100.4±4.2	105.3±1.0	67.9±3.6	94.3±7.1	127.3±7.3
**d_{11}-(*R*)-AS-1**	ND	ND	ND	<80	95.8±1.2	123.9±7.3	78.0±2.9	ND	ND
**(*R*)-AS-7**	63.2±4.3	36.8±1.1 *ER=0.6*	1.97±0.6	<80	102.8±5.8	115.7±7.9	98.6±4.4	86.4±9.1	92.8±3.9
**d_6-(*R*)-AS-7**	57.5±5.7	29.1±4.6 *ER=0.5*	2.08±0.8	<80	99.40±6.2	120.6±6.9	91.1±5.7	86.5±6.8	111.0±8.3
**References**	Caffeine 32.5±2.9	Caffeine 34.8±10.9 *ER=1.1*	Caffeine 6.32±1.1	Warfarin 98.5±2.1	Ketoconazole [1 µM] 5.1±0.2	Quinidine [1 µM] 4.4±0.7	Sulfaphenazole [1 µM] 9.5±0.3	Doxorubicin [1 µM] 21.5±3.0	Doxorubicin [1 µM] 9.9±1.6

* Results for **(*R*)-AS-1** were published previously.[6] ER – efflux ratio. ND – no data.

A different result from the PAMPA assay was obtained for **(*R*)-AS-7** and **d_6-(*R*)-AS-7** in the Caco-2 absorption model. Both compounds exhibited higher apparent permeability (P_{app}) values in the apical-to-basolateral (A–B) direction compared to the reference compound, caffeine (P_{app} = 32.5 × 10^{-6} cm/s; see **Table S7**). The P_{app} values for the parent compound and its deuterated analogue were similar: P_{app} = 63.2 × 10^{-6} cm/s for **(*R*)-AS-7** and P_{app} = 57.5 × 10^{-6} cm/s for **d_6-(*R*)-AS-7**. Moreover, both compounds demonstrated significantly lower absorption in the basolateral-to-apical (B–A) direction, with P_{app} values of 36.8 × 10^{-6} cm/s for **(*R*)-AS-7** and 29.1 × 10^{-6} cm/s for **d_6-(*R*)-AS-7**. Furthermore, the calculated very low efflux ratios indicate that drug efflux pumps, such as P-glycoprotein, are unlikely to be involved.

Plasma protein binding (PPB) is a crucial parameter for assessment of drug candidates' biological activity. All tested compounds showed desired low fraction bound (f_b<80%) PPB in comparison to warfarin used as a reference highly-bound compound (f_b =98.5%) (**Table S7**).

The drug–drug interaction studies were done by the measurement of CYP 3A4, 2D6 and 2C9 isoforms activity in the presence of the tested compounds. The obtained results did not show meaningful (≤50%) inhibition of the tested CYPs isoforms at concentration of 10 µM (**Table S7**). Almost all compounds showed slight activation of CYP2D6 at 10 and 25 µM, which were the highest concentrations tested (**Figures S16–S19**). Interestingly, a comparable effect was already observed for structurally similar series of functionalized amino acids derivatives with anticonvulsant activity.[15–17] Finally, no significant differences between **(*R*)-AS-1**, **(*R*)-AS-7** and their selected deuterated analogs were observed in CYPs activity tests.

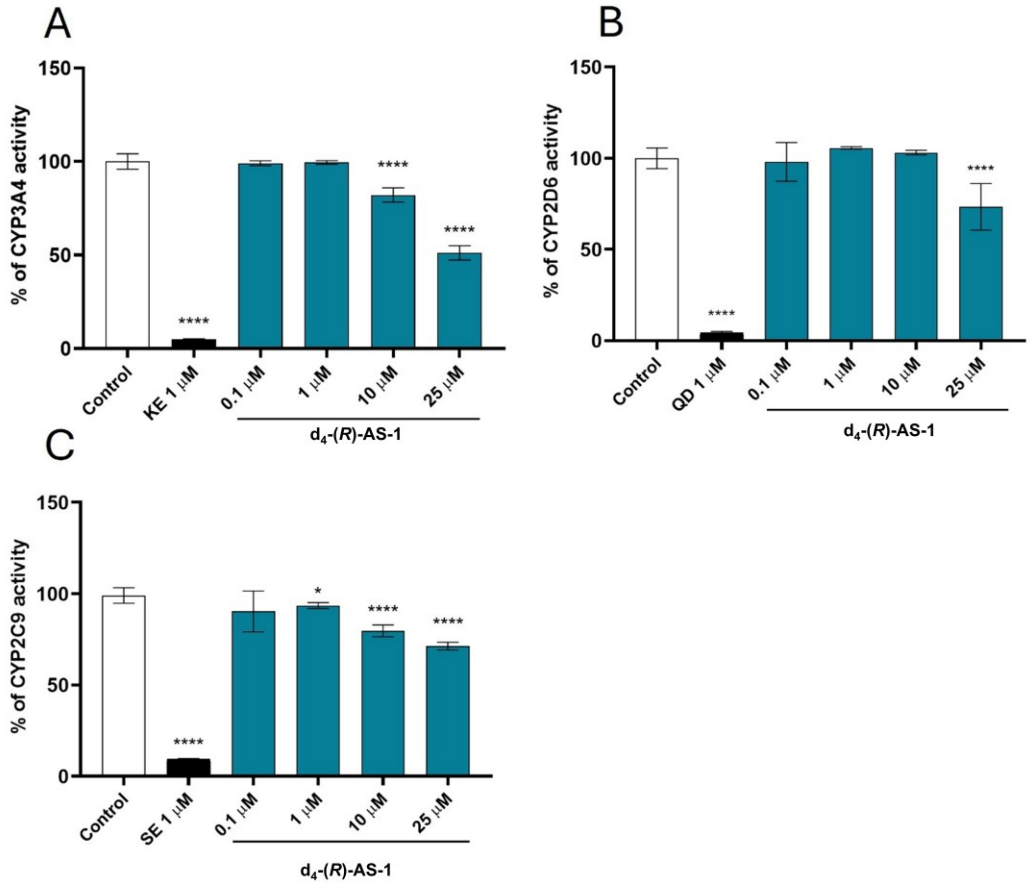

Figure S16. **The influence of d_4-(*R*)-AS-1 on CYPs activity**. (**A**) Influence on CYP3A4, ketoconazole (KE, 1 µM) was used as reference inhibitor. (**B**) Influence on CYP2D6 activity, quinidine (QD, 1 µM) was used as reference inhibitor. (**C**) Influence on CYP2C9 activity. Sulfaphenazole (SE, 1 µM) was used as reference inhibitor. Data are presented as means ± SD. Statistical analysis (GraphPad Prism 8.0.1): * < 0.05, ****p < 0.0001 *vs*. control activity (one-way ANOVA followed by Bonferroni's *post hoc* test).

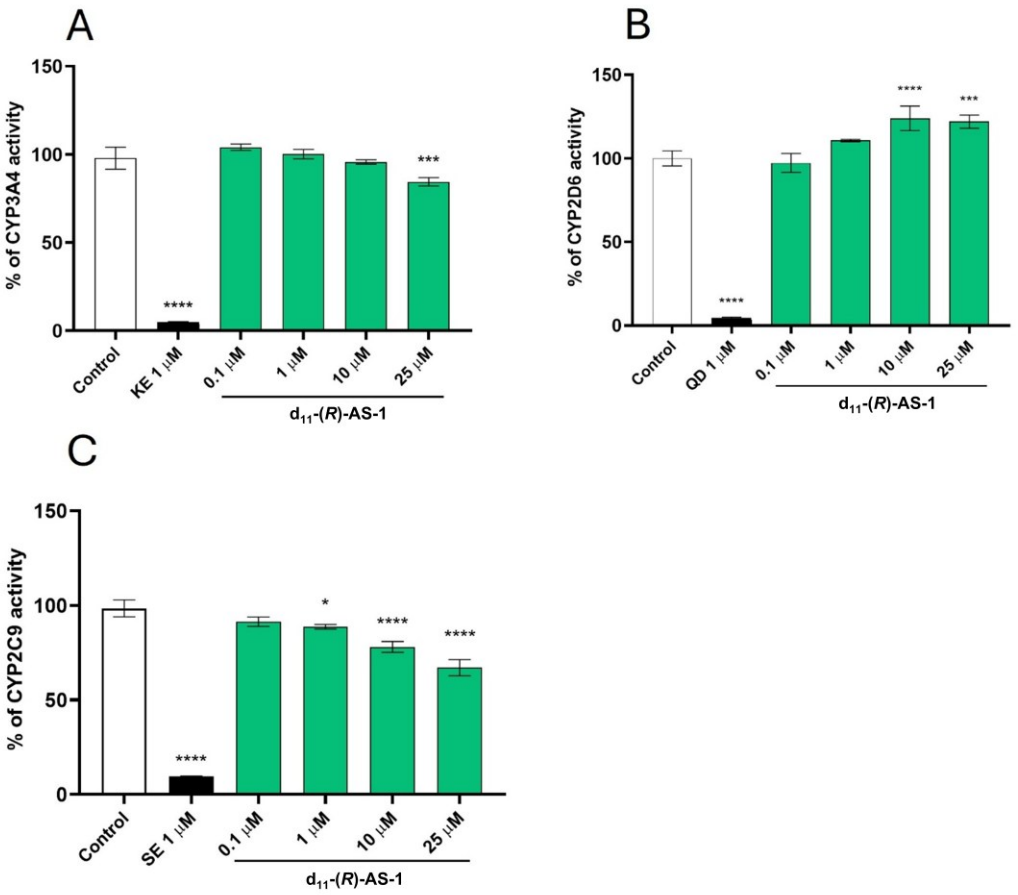

Figure S17. **The influence of d_{11}-(*R*)-AS-1 on CYPs activity**. (**A**) Influence on CYP3A4, ketoconazole (KE, 1 μM) was used as reference inhibitor. (**B**) Influence on CYP2D6 activity, quinidine (QD, 1 μM) was used as reference inhibitor. (**C**) Influence on CYP2C9 activity. Sulfaphenazole (SE, 1 μM) was used as reference inhibitor. Data are presented as means ± SD. Statistical analysis (GraphPad Prism 8.0.1): *p < 0.05, ***p < 0.001, ****p < 0.0001 *vs.* control activity (one-way ANOVA followed by Bonferroni's *post hoc* test).

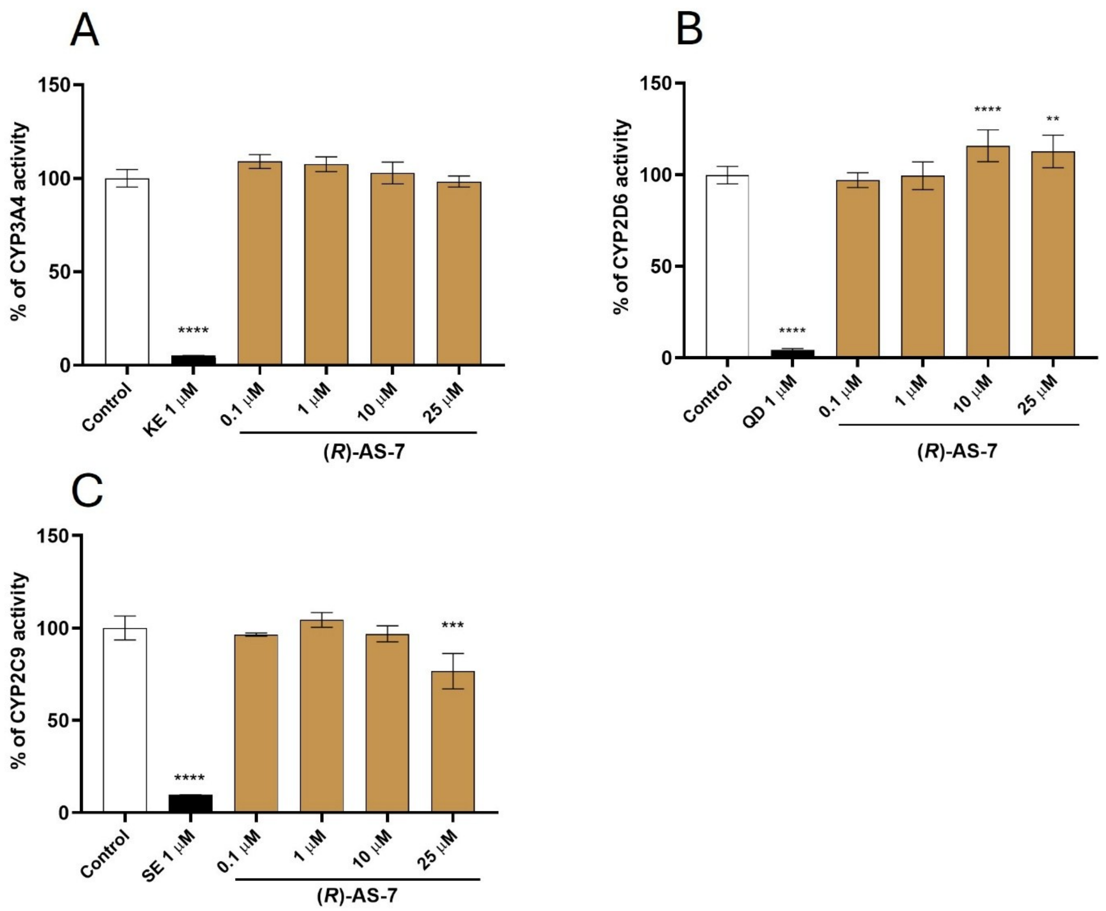

Figure S18. **The influence of (*R*)-AS-7 on CYPs activity**. (**A**) Influence on CYP3A4, ketoconazole (KE, 1 µM) was used as reference inhibitor. (**B**) Influence on CYP2D6 activity, quinidine (QD, 1 µM) was used as reference inhibitor. (**C**) Influence on CYP2C9 activity. Sulfaphenazole (SE, 1 µM) was used as reference inhibitor. Data are presented as means ± SD. Statistical analysis (GraphPad Prism 8.0.1): **$p < 0.01$, ***$p < 0.001$, ****$p < 0.0001$ *vs.* control activity (one-way ANOVA followed by Bonferroni's *post hoc* test).

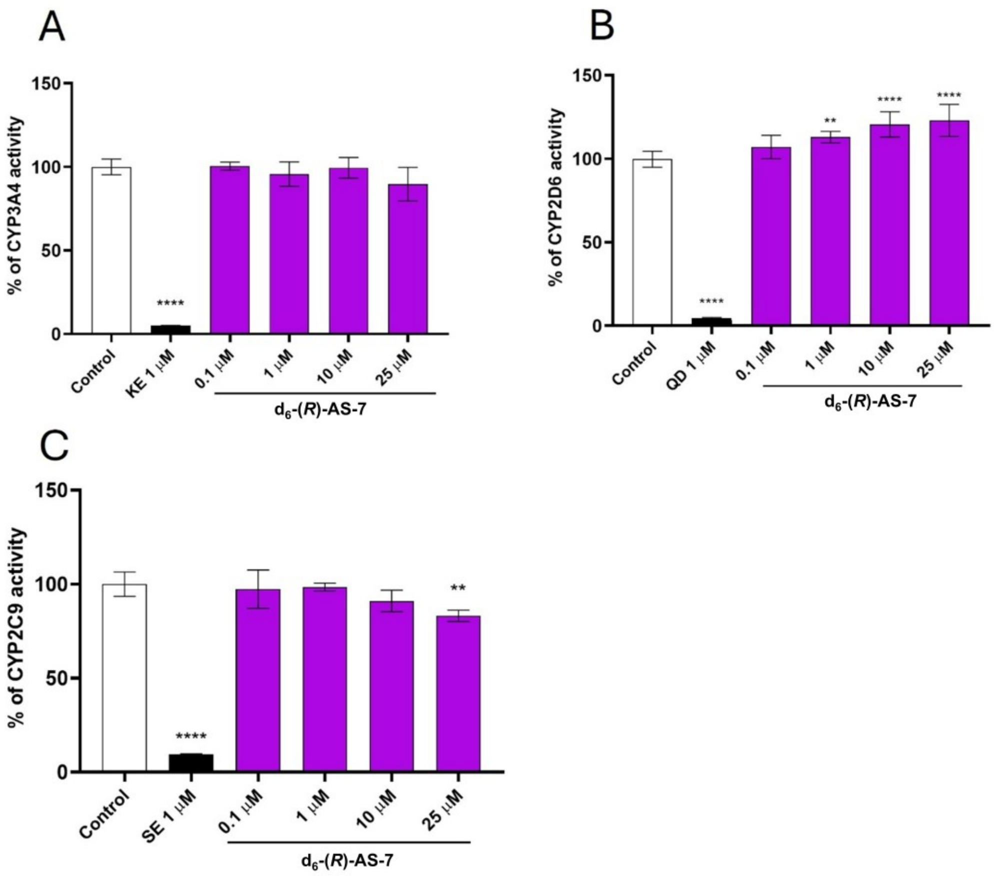

Figure S19. **The influence of d_6-(*R*)-AS-7 on CYPs activity**. (**A**) Influence on CYP3A4, ketoconazole (KE, 1 µM) was used as reference inhibitor. (**B**) Influence on CYP2D6 activity, quinidine (QD, 1 µM) was used as reference inhibitor. (**C**) Influence on CYP2C9 activity. Sulfaphenazole (SE, 1 µM) was used as reference inhibitor. Data are presented as means ± SD. Statistical analysis (GraphPad Prism 8.0.1): *p < 0.05, **p < 0.01, ***p < 0.001, ****p < 0.0001 vs. control activity (one-way ANOVA followed by Bonferroni's *post hoc* test).

Hepatotoxicity and neurotoxicity *in vitro* assays with use of hepatoma HepG2 and neuroblastoma SH-SY5Y cell lines confirmed high safety margins of the tested compounds, as no meaningful decrease of cells viability was observed even at the highest concentration of 100 µM. (**Table S7**) (**Figures S20–S22**).

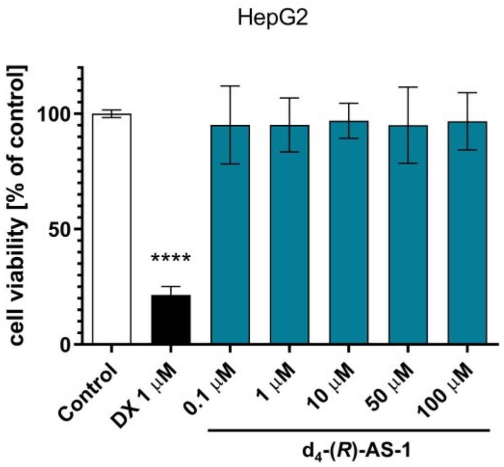

Figure S20. **The viability of hepatoma HepG2 cell line after incubation with d_4-(*R*)-AS-1 for 72 h**. Data are presented as means ± SD. Statistical analysis (GraphPad Prism 8.0.1): ****p < 0.0001 *vs.* negative control – medium with 1% DMSO (one-way ANOVA followed by Bonferroni's *post hoc* test).

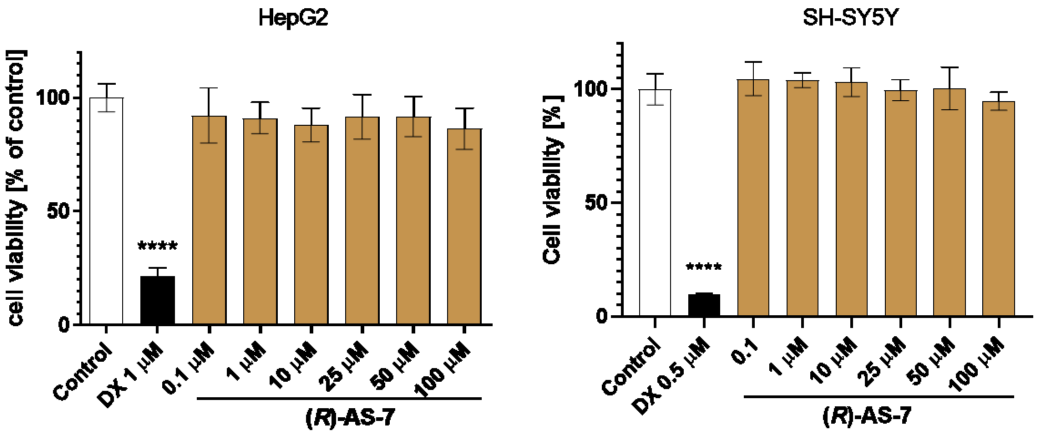

Figure S21. **The viability of hepatoma HepG2 cells and neuroblastoma SH-SY5Y cell line after incubation with (*R*)-AS-7 for 72 h and 48 h, respectively**. Doxorubicin (DX, 1 µM) was used as reference at concentration of 1 µM (left graph) or 0.5 µM (right) graph. Data are presented as means ± SD. Statistical analysis (GraphPad Prism 8.0.1): ****p < 0.0001 *vs.* negative control – medium with 1% DMSO (one-way ANOVA followed by Bonferroni's *post hoc* test).

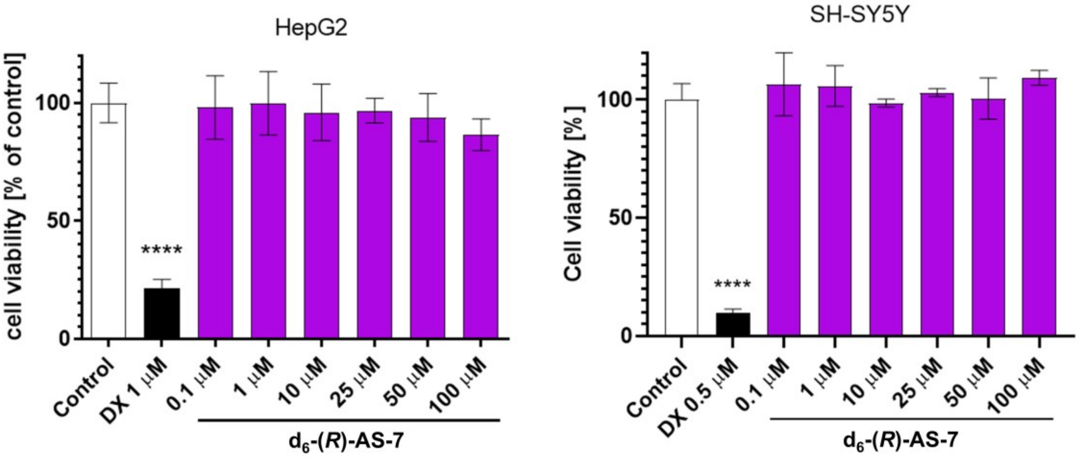

Figure S22. **The viability of hepatoma HepG2 cells and neuroblastoma SH-SY5Y cell line after incubation with d_6-(*R*)-AS-7 for 72 h and 48 h, respectively**. Doxorubicin (DX, 1 µM) was used as reference at concentration of 1 µM (left graph) or 0.5 µM (right) graph. Data are presented as means ± SD. Statistical analysis (GraphPad Prism 8.0.1): ****p < 0.0001 *vs*. negative control – medium with 1% DMSO (one-way ANOVA followed by Bonferroni's *post hoc* test).

As part of the further safety profiling of **d_6-(*R*)-AS-7**, the risk of phospholipidosis induction was assessed in HepG2 cells using the LYSO-ID® Red cytotoxicity kit, which allows fluorescence-based visualization of nuclei and lysosomal or lysosome-like organelle changes in live cells. The HepG2 cell line was exposed for 24 h to 10 µM and 50 µM of the reference compound verapamil (a phospholipidosis inducer) and to 50 µM and 100 µM concentrations of **d_6-(*R*)-AS-7**. No phospholipidosis induction was observed for **d_6-(*R*)-AS-7** compared with verapamil, either in the collected images or in the measurement of red fluorescent lysosomal signal intensity using Leica DMi8 microscopy (**Figures S23** and **S24**).

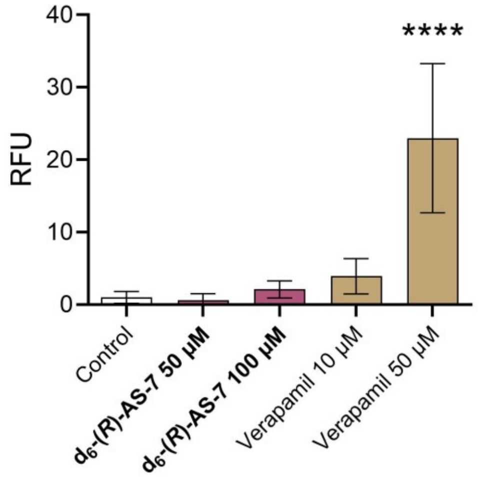

Figure S23. The red fluorescent lysosomal signal (RFU) intensity of the HepG2 cells treated with 50 µM, 100 µM of d_6-(*R*)-AS-7 and 10 µM, 50 µM of verapamil (phospholipidosis inductor). The cells were stained by LYSO-ID® Red cytotoxicity kit containing Dual Color Detection Reagent. The fluorescence was measured by Leica DMi8 microscopy. Statistical analysis (GraphPad Prism 8.0.1): One-way ANOVA, followed by Bonferroni's comparison test, **** p < 0.0001, compared with negative control).

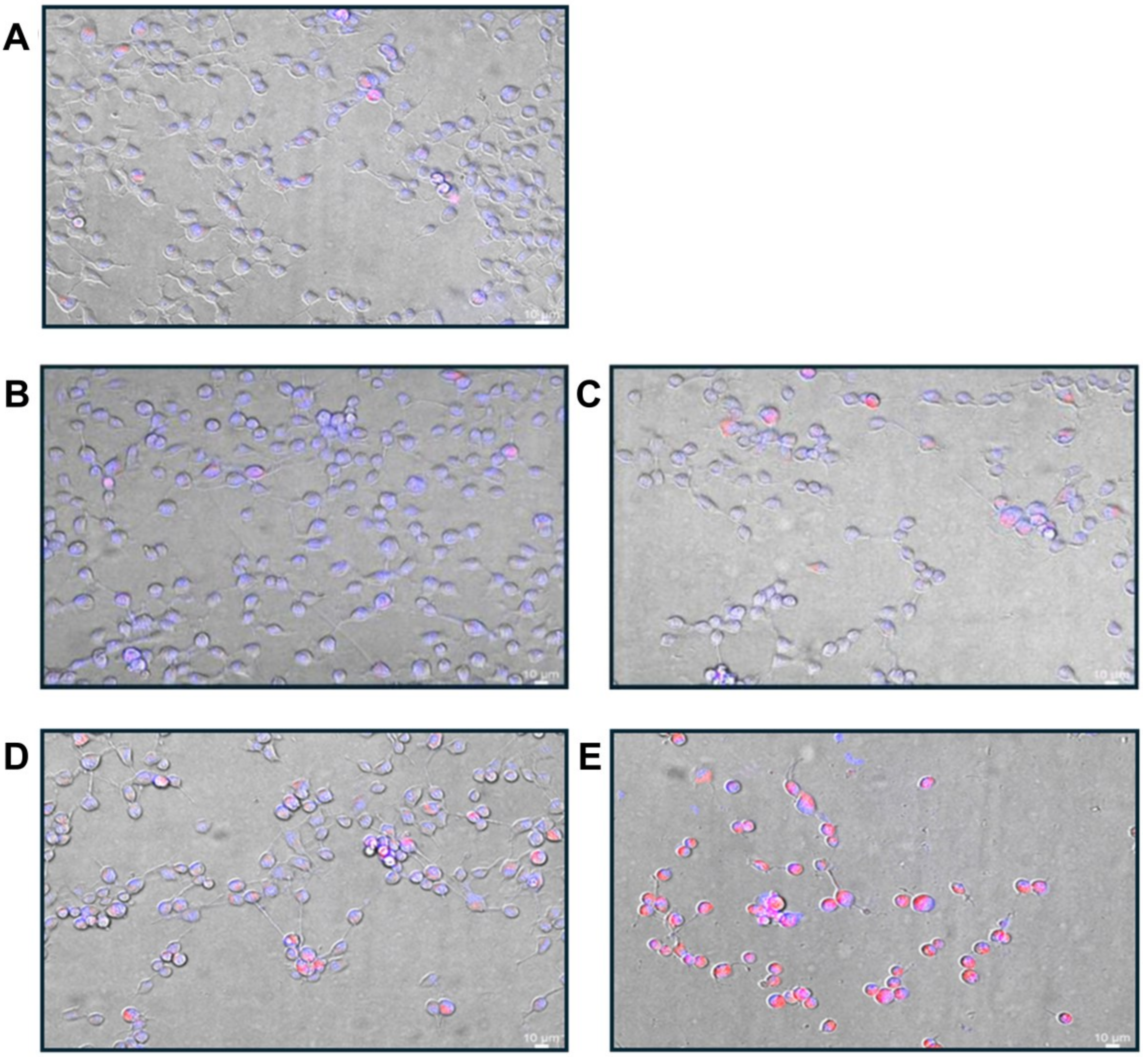

Figure S24. Microscopy images of HepG2 cells: not treated (A), exposed for 24 h to 50 µM (B), 100 µM (C) of d6-(R)-AS-7 and 10 µM (D), 50 µM (D) of verapamil (phospholipidosis inductor). The cells were stained by LYSO-ID® Red cytotoxicity kit containing Dual Color Detection Reagent. The red fluorescent lysosomal signal and the blue nuclear signal were registered by microscope Leica DMi8.

The metabolic stability data for parent **(*R*)-AS-1** and its analog containing eleven deuterium atoms – **d_{11}-(*R*)-AS-1**, showed that both compounds were stable in mouse liver microsomes (MLMs) and human liver microsomes (HLMs) with $t_{1/2}$ of >150 min and >300 min, respectively (**Table S8**). They exhibited nearly identical metabolic stability within a given species, with intrinsic clearance (Cl_{int}) values of 26.6 and 34.4 mL/min/kg in MLMs, and 1.8 and 3.8 mL/min/kg in HLMs, respectively. These results clearly indicate that **(*R*)-AS-1** and **d_{11}-(*R*)-AS-1** are more extensively metabolized by mouse microsomes than by human microsomes. For comparison, the *in vitro* PK parameters of the metabolically unstable reference compound, verapamil, incubated under the same conditions, were as follows: in HLMs, $t_{1/2}$ = 8 min and Cl_{int} = 153.2 mL/min/kg, whereas for MLMs $t_{½}$ = 22 min and Cl_{int} = 239.5 mL/min/kg.

Similarly, **(*R*)-AS-7** and **d_6-(*R*)-AS-7** demonstrated high metabolic stability during incubation with MLMs, with estimated half-lives ($t_{1/2}$) exceeding 300 min (**Table S8**). The calculated Cl_{int} values were also comparable: 14.9 mL/min/kg for (***R***)**-AS-7** and 14.1 mL/min/kg for **d_6-(*R*)-AS-7**. In the case of HLMs, both compounds were equally stable, with half-lives ($t_{1/2}$) greater than 300 min and Cl_{int} values of 5.0 mL/min/kg for **(*R*)-AS-7** and 2.5 mL/min/kg for **d_6-(*R*)-AS-7** (**Table S8**).

Table S8. Comparison of the metabolic stability in mouse and human microsomes, liver S9 fraction and hepatocytes.

Compound	Mouse microsomes		Human microsomes	
	$t_{½}$ [min][a]	Cl_{int} [mL/min/kg][b]	$t_{½}$ [min][a]	Cl_{int} [mL/min/kg][b]
**(*R*)-AS-1**	204	26.6	>300	1.8
**d_{11}-(*R*)-AS-1**	157	34.4	>300	3.8
**(*R*)-AS-7**	364	14.9	>300	5.0
**d_6-(*R*)-AS-7**	385	14.1	>300	2.5
**Verapamil**	22	239	8.0	153

Compound[#]	Mouse S9 liver fraction		Human S9 liver fraction	
	$t_{½}$ [min][a]	Cl_{int} [mL/min/kg][b]	$t_{½}$ [min][a]	Cl_{int} [mL/min/kg][b]
**(*R*)-AS-7**	282	52.2	1903	1.8
**d_6-(*R*)-AS-7**	244	60.4	2858	1.2
**7-hydroxycoumarin**	12.1	1215	22.9	158

Compound*	Mouse hepatocytes		Human hepatocytes	
	$t_{½}$ [min][a]	Cl_{int} [mL/min/kg][b]	$t_{½}$ [min][a]	Cl_{int} [mL/min/kg][b]
**(*R*)-AS-1**	214	38	>360	<1.0
**d_{11}-(*R*)-AS-1**	340	23	>1540	<1.0
**Verapamil**	3.8	2074	11.9	140

[a] $t_{1/2}$ – half-life; [b] Cl_{int} – intrinsic clearance. * **(*R*)-AS-7** and **d_6-(*R*)-AS-7** were not evaluated in hepatocytes. [#]**(*R*)-AS-1** and **d_{11}-(*R*)-AS-1** were not evaluated in S9 liver fraction.

To further elucidate the influence of hydrogen-deuterium exchange on compound stability, the metabolic stability study of the parent compound **(*R*)-AS-7** and its deuterated analog **d_6-(*R*)-AS-7** was conducted in the presence of mouse or human S9 liver fraction and cofactors of II phase metabolic reactions (UDPGA and GSH) (**Table S8**). Both compounds demonstrated low Cl_{int} in the presence of mouse S9 fraction (52.18 and 60.44 mL/min/kg for **(*R*)-AS-7** and **d_6-(*R*)-AS-7**, respectively). Calculated Cl_{int} values for reactions conducted in the presence of human S9 fraction were even lower (1.76 and 1.17 mL/min/kg for **(*R*)-AS-7** and **d_6-(*R*)-AS-7**, respectively). The data suggests a higher metabolic stability of **(*R*)-AS-7** in the presence of human S9 liver fraction compared to mouse S9 fraction ($t_{1/2}$ = 1903 min and 282 min, respectively). **d_6-(*R*)-AS-7** also showed increased stability with human S9 fraction ($t_{1/2}$ = 2858 and 244 min, respectively). Interestingly, deuterated analog was more stable in the presence of human S9 fraction and the parent compound with mouse S9 liver fraction. For comparison, a reference II phase conjugation substrate (7-hydroxycoumarin) was incubated under the same conditions as the tested compounds in the presence of mouse or human S9 fraction (**Table S8**). Calculated *in vitro* PK parameters suggest a lower metabolic stability of 7-hydroxycoumarin incubated with mouse S9 fraction compared to human S9 fraction ($t_{1/2}$ = 12.1 min and Cl_{int} = 1215 mL/min/kg with mouse S9 fraction and $t_{1/2}$ = 22.9 min and Cl_{int} = 158.8 mL/min/kg with human S9 fraction, respectively), which corresponds with a lower stability of the tested compounds incubated with mouse S9 fraction.

Finally, **d_{11}-(*R*)-AS-1** was also highly metabolically stable during incubation with mouse hepatocytes, resulting in an estimated $t_{1/2}$ > 340 min. The non-deuterated **(*R*)-AS-1** showed slightly lower hepatocyte stability ($t_{1/2}$ = 214 min) suggesting that hydrogen-deuterium exchange may protect compounds against complex biotransformation processes in hepatocytes (e.g. phase II conjugation reactions). Notably, both compounds showed excellent stability in mouse and human hepatocytes compared with metabolically non-stable reference drug, verapamil.

Collectively, the *in vitro* stability data strongly support the favorable drug-like properties of both the parent and deuterium-containing compounds.

4. Material and methods

Chemistry

General information. All chemicals and solvents were purchased from commercial suppliers and were used without further purification. Melting points (mp.) were determined in open capillaries on a Büchi 353 melting point apparatus (Büchi Labortechnik, Flawil, Switzerland) and are uncorrected. The purity and homogeneity of the compounds were assessed by thin-layer chromatography (TLC) and the gradient UPLC chromatography. Thin-layer TLC was performed in silica gel 60 F_{254} pre-coated aluminum sheets (Macherey-Nagel, Düren, Germany), using developing system that consisted of the following: S_1–DCM:MeOH (9:0.3; *v*/*v*), S_2–DCM:MeOH (9:0.5; *v*/*v*). Spots were detected by their absorption under UV light (λ = 254 nm). The UPLC and mass spectra (LC-MS) were obtained on Waters ACQUITY™ TQD system (Waters, Milford, CT, USA) with the MS-TQ detector and UV-Vis-DAD eλ detector. The ACQUITY UPLC BEH C18, 1.7 µm (2.1 × 100 mm) column was used with the VanGuard Acquity UPLC BEH C18, 1.7 µm (2.1 × 5 mm) (Waters, Milford, CT, USA). Standard solutions (1 mg/mL) of each compound were prepared in analytical grade MeCN/water mixture (1:1; *v*/*v*). Conditions applied were as follows: eluent A (water/0.1% HCOOH), eluent B (MeCN/0.1% HCOOH), a flow rate of 0.3 mL/min, a gradient of 5–100% B over 10 min, and an injection volume of 10 µL. The UPLC analyses and high-resolution mass spectra (LC-HRMS) were obtained on a Waters ACQUITY I-Class PLUS SYNAPT XS High-Resolution Mass Spectrometer (Waters, Milford, CT, USA) with an MS-Q-TOF detector and a UV–vis-DAD eλ detector. The UPLC retention times (t_R) are given in min. The purity of target compounds determined by use of chromatographic UPLC method was ≥99%. Preparative column chromatography was performed using silica gel 60 (particle size 0.063–0.200; 70–230 Mesh ATM) purchased from Merck (Darmstadt, Germany). ^{1}H NMR and ^{13}C NMR spectra were obtained in a JEOL-500 spectrometer (JEOL USA, Inc. MA, USA), in $CDCl_3$ operating at 500 MHz (^{1}H NMR) 126 MHz (^{13}C NMR). Chemical shifts are reported in δ values (ppm) relative to TMS δ = 0 (^{1}H), as internal standard. The J values are expressed in Hertz (Hz). Signal multiplicities are represented by the following abbreviations: br s (broad singlet), d (doublet), ddd (doublet of doublet of doublets), td (triplet of doublets), q (quartet), qd (quartet of doublets) m (multiplet). Chiral SFC assays were conducted on Agilent 1260 Infinity II Analytical SFC. The Trefoil CEL2, column (2.5 µm, 150 × 2.1 mm) was used. Standard solutions (1 mg/mL) of each compound were prepared in methanol. The conditions applied were as follows: CO_2/MeOH = 88/12 (v/v), flow rate: 0.7 mL/min, detection at λ = 210 nm. Oven temperature: 40°C. Determination of deuteration rate (deuterium incorporation) in percent [%] were measured by ^{1}H NMR as the decrease of the intensity of the signal(s) of respective proton(s) compared with non-deuterated parent molecules.[18]

The parent non-deuterated compounds (*R*)-AS-1 and (*R*)-AS-7 were obtained following the method described in our previous study.[6]

Method for the preparation of deuterated benzylamine derivatives A1–A4. In a dry round-bottom flask placed in an ice bath under an inert gas atmosphere (argon), a suspension of $LiAlH_4$ or $LiAlD_4$ (3 eq) in 20 mL of anhydrous THF was prepared. Then, a solution of the appropriate nitrile (1 eq) in 20 mL anhydrous THF was added dropwise. After 10 min of cooling, the reaction was continued at room temperature under an argon atmosphere. The reaction progress was monitored using HPLC. After 30 min, the reaction mixture was cooled in an ice bath again, and sequentially, 2 mL of distilled water, 4 mL of 10% NaOH, and another 2 mL of distilled water were added. The mixture was stirred for 15 min, then filtered through Celite®, concentrated, and extracted with a DCM/water. The organic layer was dried over anhydrous Na_2SO_4, and the solvent was evaporated under reduced pressure. The deuterated benzylamine derivatives (A1–A4) were advanced to further reactions without purification.

Benzylamine-d$_2$ (A1). Light oil. Yield: 94% (5.50 g); UPLC (purity = 93.4%): t_R = 2.01 min. $C_7H_7D_2N$ (109.17). LC-MS (ESI): *m/z* calcd for $C_7H_7D_2N$ $(M+H)^+$ 110.09, found 110.2.

Benzylamine-d$_5$ (A2). Light oil. Yield: 92% (5.63 g); UPLC (purity = 89.1%): t_R = 2.03 min. $C_7H_4D_5N$ (112.19). LC-MS (ESI): *m/z* calcd for $C_7H_4D_5N$ $(M+H)^+$ 113.10, found 113.1.

Benzylamine-d$_7$ (A3). Light oil. Yield: 89% (5.41 g); UPLC (purity = 87.5%): t_R = 2.08 min. $C_7H_2D_7N$ (114.20). LC-MS (ESI): *m/z* calcd for $C_7H_2D_7N$ $(M+H)^+$ 115.12, found 115.2.

2-Fluorobenzylamine-d$_2$ (A4). Light oil. Yield: 91% (5.58 g); UPLC (purity = 87.6%): t_R = 2.25 min. $C_7H_6D_2FN$ (127.16). LC-MS (ESI): *m/z* calcd for $C_7H_6D_2FN$ $(M+H)^+$ 128.08, found 128.2.

Method for the preparation of intermediates (*R*)-1–(*R*)-6 and (*R,S*)-1–(*R,S*)-6. To a solution of Boc-D-alanine or Boc-D,L-alanine (5.0 g, 27 mmol, 1 eq) in DCM, (100 mL), DCC (6.81 g, 1.2 eq) dissolved in 10 mL of DCM was added gradually. After stirring for 15 min at room temperature, a solution of commercial benzylamine or 2-fluorobenzylamine, and non-commercial benzylamine-d$_2$ (A1), benzylamine-d$_5$ (A2), benzylamine-d$_7$ (A3), or 2-fluorobenzylamine-d$_2$ (A4) (1 eq) in 5 mL of DCM was added dropwise, and the reaction mixture was stirred for 1 h. The DCM was then evaporated under reduced pressure, and the resulting product was purified by column chromatography using a DCM/MeOH (9:0.3, *v/v*) solvent system.

(*R*)-*tert*-butyl-(1-(benzylamino)-1-oxopropan-2-yl)carbamate, (*R*)-1. Light oil. Yield: 91% (6.95 g); TLC: R_f = 0.43 (S_1); UPLC (purity >99%): t_R = 5.44 min. $C_{15}H_{22}N_2O_3$ (278.35). LC-MS (ESI): *m/z* calcd for $C_{15}H_{22}N_2O_3$ $(M+H)^+$ 279.16, found 279.3.

(*R*)-*tert*-butyl-(1-oxo-1-((phenylmethyl-d$_2$)amino)propan-2-yl)carbamate, (*R*)-2. Light oil. Yield: 93% (7.18 g); TLC: R_f = 0.43 (S_1); UPLC (purity >99%): t_R = 5.43 min. $C_{15}H_{20}D_2N_2O_3$ (280.36). LC-MS (ESI): *m/z* calcd for $C_{15}H_{20}D_2N_2O_3$ $(M+H)^+$ 281.18, found 281.2.

(*R*)-*tert*-butyl-(1-oxo-1-(((phenyl-d$_5$)methyl)amino)propan-2-yl)carbamate, (*R*)-3. Light oil. Yield: 92% (7.08 g); TLC: R_f = 0.43 (S_1); UPLC (purity >99%): t_R = 5.41 min. $C_{15}H_{17}D_5N_2O_3$ (283.38). LC-MS (ESI): *m/z* calcd for $C_{15}H_{17}D_5N_2O_3$ $(M+H)^+$ 284.19, found 284.1.

(*R*)-*tert*-butyl-(1-oxo-1-(((phenyl-d$_5$)methyl-d$_2$)amino)propan-2-yl)carbamate, (*R*)-4. Light oil. Yield: 94% (7.39 g); TLC: R_f = 0.43 (S_1); UPLC (purity >99%): t_R = 5.42 min. $C_{15}H_{15}D_7N_2O_3$ (285.39). LC-MS (ESI): *m/z* calcd for $C_{15}H_{15}D_7N_2O_3$ $(M+H)^+$ 286.21, found 286.3.

(*R*)-*tert*-butyl-(1-((2-fluorobenzyl)amino)-1-oxopropan-2-yl)carbamate, (*R*)-5. Light oil. Yield: 93% (7.28 g); TLC: R_f = 0.45 (S_1); UPLC (purity >99%): t_R = 5.54 min. $C_{15}H_{21}FN_2O_3$ (296.34). LC-MS (ESI): *m/z* calcd for $C_{15}H_{21}FN_2O_3$ $(M+H)^+$ 297.15, found 297.2.

(*R*)-*tert*-butyl-(1-(((2-fluorophenyl)methyl-d$_2$)amino)-1-oxopropan-2-yl)carbamate, (*R*)-6. Light oil. Yield: 93% (7.33 g); TLC: R_f = 0.45 (S_1); UPLC (purity >99%): t_R = 5.53 min. $C_{15}H_{19}D_2FN_2O_3$ (298.35). LC-MS (ESI): *m/z* calcd for $C_{15}H_{19}D_2FN_2O_3$ $(M+H)^+$ 299.17, found 299.2.

(*R,S*)-*tert*-butyl-(1-(benzylamino)-1-oxopropan-2-yl)carbamate, (*R,S*)-1. Light oil. Yield: 93% (7.16 g); TLC: R_f = 0.43 (S_1); UPLC (purity >99%): t_R = 5.42 min. $C_{15}H_{22}N_2O_3$ (278.35). LC-MS (ESI): *m/z* calcd for $C_{15}H_{22}N_2O_3$ $(M+H)^+$ 279.16, found 279.1.

(*R,S*)-*tert*-butyl-(1-oxo-1-((phenylmethyl-d$_2$)amino)propan-2-yl)carbamate, (*R,S*)-2. Light oil. Yield: 92% (7.10 g); TLC: R_f = 0.43 (S_1); UPLC (purity >99%): t_R = 5.43 min. $C_{15}H_{20}D_2N_2O_3$ (280.36). LC-MS (ESI): *m/z* calcd for $C_{15}H_{20}D_2N_2O_3$ $(M+H)^+$ 281.18, found 281.2.

(*R,S*)-*tert*-butyl-(1-oxo-1-(((phenyl-d$_5$)methyl)amino)propan-2-yl)carbamate, (*R,S*)-3. Light oil. Yield: 89% (6.85 g); TLC: R_f = 0.43 (S_1); UPLC (purity >99%): t_R = 5.43 min. $C_{15}H_{17}D_5N_2O_3$ (283.38). LC-MS (ESI): *m/z* calcd for $C_{15}H_{17}D_5N_2O_3$ $(M+H)^+$ 284.19, found 284.2.

(*R,S*)-*tert*-butyl-(1-oxo-1-(((phenyl-d$_5$)methyl-d$_2$)amino)propan-2-yl)carbamate, (*R,S*)-4. Light oil. Yield: 91% (7.15 g); TLC: R_f = 0.43 (S_1); UPLC (purity >99%): t_R = 5.41 min. $C_{15}H_{15}D_7N_2O_3$ (285.39). LC-MS (ESI): *m/z* calcd for $C_{15}H_{15}D_7N_2O_3$ $(M+H)^+$ 286.21, found 286.3.

(*R,S*)-*tert*-butyl-(1-((2-fluorobenzyl)amino)-1-oxopropan-2-yl)carbamate, (*R,S*)-5. Light oil. Yield: 90% (7.05 g); TLC: R_f = 0.45 (S_1); UPLC (purity >99%): t_R = 5.56 min. $C_{15}H_{21}FN_2O_3$ (296.34). LC-MS (ESI): *m/z* calcd for $C_{15}H_{21}FN_2O_3$ $(M+H)^+$ 297.15, found 297.1.

(*R,S*)-*tert*-butyl-(1-(((2-fluorophenyl)methyl-d$_2$)amino)-1-oxopropan-2-yl)carbamate, (*R,S*)-6. Light oil. Yield: 90% (7.09 g); TLC: R_f = 0.45 (S_1); UPLC (purity >99%): t_R = 5.54 min. $C_{15}H_{19}D_2FN_2O_3$ (298.35). LC-MS (ESI): *m/z* calcd for $C_{15}H_{19}D_2FN_2O_3$ $(M+H)^+$ 299.17, found 299.2.

Procedure for the preparation of intermediates (*R*)-7–(*R*)-12 and (*R,S*)-7–(*R,S*)-12. A solution of either (*R*)-1–(*R*)-6 and (*R,S*)-1–(*R,S*)-6 (20 mmol, 1 eq) in DCM (100 mL) was treated with TFA (6.84 g, 4.56 mL, 60 mmol, 3 eq) and stirred at room temperature for 1 h. Afterwards, the 25% ammonium hydroxide was carefully added to adjust the pH to 8. The aqueous layer was extracted with DCM (3 × 50 mL), dried over Na_2SO_4, and concentrated under vacuum to yield (*R*)-7–(*R*)-12 and (*R,S*)-7–(*R,S*)-12 as yellow oils, which were used directly in subsequent reactions without further purification.

(*R*)-2-amino-*N*-benzylpropanamide, (*R*)-7. Light oil. Yield: 89% (3.90 g); TLC: R_f = 0.21 (S_2); UPLC (purity = 96.8%): t_R = 2.11 min. $C_{10}H_{14}N_2O$ (178.24). LC-MS (ESI): *m/z* calcd for $C_{10}H_{14}N_2O$ $(M+H)^+$ 179.11, found 179.2.

(*R*)-2-amino-*N*-(phenylmethyl-d$_2$)propanamide, (*R*)-8. Light oil. Yield: 95% (4.30 g); TLC: R_f = 0.21 (S_2); UPLC (purity >99%): t_R = 2.12 min. $C_{10}H_{12}D_2N_2O$ (180.25). LC-MS (ESI): *m/z* calcd for $C_{10}H_{12}D_2N_2O$ $(M+H)^+$ 181.12, found 181.3.

(*R*)-2-amino-*N*-((phenyl-d$_5$)methyl)propanamide, (*R*)-9. Light oil. Yield: 94% (4.30 g); TLC: R_f = 0.21 (S_2); UPLC (purity = 96.3%): t_R = 2.16 min. $C_{10}H_9D_5N_2O$ (183.27). LC-MS (ESI): *m/z* calcd for $C_{10}H_9D_5N_2O$ $(M+H)^+$ 184.14, found 184.1.

(*R*)-2-amino-*N*-((phenyl-d$_5$)methyl-d$_2$)propanamide, (*R*)-10. Light oil. Yield: 93% (4.40 g); TLC: R_f = 0.21 (S_2); UPLC (purity >99%): t_R = 2.16 min. $C_{10}H_7D_7N_2O$ (185.28). LC-MS (ESI): *m/z* calcd for $C_{10}H_7D_7N_2O$ $(M+H)^+$ 186.15, found 186.2.

(*R*)-2-amino-*N*-(2-fluorobenzyl)propanamide, (*R*)-11. Light oil. Yield: 92% (4.40 g); TLC: R_f = 0.23 (S_2); UPLC (purity = 97.4%): t_R = 2.29 min. $C_{10}H_{13}FN_2O$ (196.23). LC-MS (ESI): *m/z* calcd for $C_{10}H_{13}FN_2O$ $(M+H)^+$ 197.10, found 197.2.

(*R*)-2-amino-*N*-((2-fluorophenyl)methyl-d$_2$)propanamide, (*R*)-12. Light oil. Yield: 95% (4.32 g); TLC: R_f = 0.23 (S_2); UPLC (purity >99%): t_R = 2.28 min. $C_{10}H_{11}D_2FN_2O$ (198.24). LC-MS (ESI): *m/z* calcd for $C_{10}H_{11}D_2FN_2O$ $(M+H)^+$ 199.11, found 199.2.

(*R,S*)-2-amino-*N*-benzylpropanamide, (*R,S*)-7. Light oil. Yield: 96% (3.90 g); TLC: R_f = 0.21 (S_2); UPLC (purity = 98.2%): t_R = 2.12 min. $C_{10}H_{14}N_2O$ (178.24). LC-MS (ESI): *m/z* calcd for $C_{10}H_{14}N_2O$ $(M+H)^+$ 179.11, found 179.3.

(*R,S*)-2-amino-*N*-(phenylmethyl-d$_2$)propanamide, (*R,S*)-8. Light oil. Yield: 93% (4.20 g); TLC: R_f = 0.21 (S_2); UPLC (purity > 99%): t_R = 2.12 min. $C_{10}H_{12}D_2N_2O$ (180.25). LC-MS (ESI): *m/z* calcd for $C_{10}H_{12}D_2N_2O$ $(M+H)^+$ 181.12, found 181.3.

(*R,S*)-2-amino-*N*-((phenyl-d$_5$)methyl)propanamide, (*R,S*)-9. Light oil. Yield: 93% (3.90 g); TLC: R_f = 0.21 (S_2); UPLC (purity = 96.8%): t_R = 2.14 min. $C_{10}H_9D_5N_2O$ (183.27). LC-MS (ESI): *m/z* calcd for $C_{10}H_9D_5N_2O$ $(M+H)^+$ 184.14, found 184.2.

(*R,S*)-2-amino-*N*-((phenyl-d$_5$)methyl-d$_2$)propanamide, (*R,S*)-10. Light oil. Yield: 94% (4.24 g); TLC: R_f = 0.21 (S_2); UPLC (purity > 99%): t_R = 2.14 min. $C_{10}H_7D_7N_2O$ (185.28). LC-MS (ESI): *m/z* calcd for $C_{10}H_7D_7N_2O$ $(M+H)^+$ 186.15, found 186.2.

(*R,S*)-2-amino-*N*-(2-fluorobenzyl)propanamide, (*R,S*)-11. Light oil. Yield: 94% (4.20 g); TLC: R_f = 0.23 (S_2); UPLC (purity = 98.2%): t_R = 2.31 min. $C_{10}H_{13}FN_2O$ (196.23). LC-MS (ESI): *m/z* calcd for $C_{10}H_{13}FN_2O$ $(M+H)^+$ 197.10, found 197.1.

(*R,S*)-2-amino-*N*-((2-fluorophenyl)methyl-d$_2$)propanamide, (*R,S*)-12. Light oil. Yield: 94% (4.32 g); TLC: R_f = 0.23 (S_2); UPLC (purity > 99%): t_R = 2.28 min. $C_{10}H_{11}D_2FN_2O$ (198.24). LC-MS (ESI): *m/z* calcd for $C_{10}H_{11}D_2FN_2O$ $(M+H)^+$ 199.11, found 199.2.

Procedure for the preparation of intermediates (*R*)-13–(*R*)-19 and (*R,S*)-13–(*R,S*)-19. To a solution of succinic anhydride (1.56 g, 15 mmol, 1 eq) or deuterated succinic anhydride -2,2,3,3-d$_4$ (1.56 g, 15 mmol, 1 eq) in ethyl acetate (10 mL) a solution of (*R*)-7–(*R*)-12 and (*R,S*)-7–(*R,S*)-12 (15 mmol, 1 eq) in ethyl acetate (40 mL) was added. The reaction mixture was stirred for 30 min, after which the ethyl acetate was evaporated to dryness. The succinamic acid derivatives, (*R*)-13–(*R*)-19 and (*R,S*)-13–(*R,S*)-19 were obtained as solid substances by washing with diethyl ether.

(*R*)-4-((1-(benzylamino)-1-oxopropan-2-yl)amino)-4-oxobutanoic-2,2,3,3-d$_4$ acid, (*R*)-13. White solid. Yield: 94% (3.31 g); Melting point: 129.5–131.6°C; TLC: R_f = 0.34 (S_2); UPLC (purity = 91.2%): t_R = 3.12 min. $C_{14}H_{14}D_4N_2O_4$ (282.33), LC-MS (ESI): *m/z* calcd for $C_{14}H_{14}D_4N_2O_4$ $(M+H)^+$ 283.15, found 283.4.

(*R*)-4-oxo-4-((1-oxo-1-(((phenyl-d$_5$)methyl)amino)propan-2-yl)amino)butanoic acid, (*R*)-14. White solid. Yield: 95% (3.20 g); Melting point: 129.9–131.8°C; TLC: R_f = 0.35 (S_2); UPLC (purity > 99%): t_R = 3.12 min. $C_{14}H_{13}D_5N_2O_4$ (283.34), LC-MS (ESI): *m/z* calcd for $C_{14}H_{13}D_5N_2O_4$ $(M+H)^+$ 284.15, found 284.1.

(*R*)-4-oxo-4-((1-oxo-1-((phenylmethyl-d$_2$)amino)propan-2-yl)amino)butanoic-2,2,3,3-d$_4$ acid, (*R*)-15. White solid. Yield: 95% (3.23 g); Melting point: 129.5–131.8°C; TLC: R_f = 0.34 (S_2); UPLC (purity > 99%): t_R = 3.13 min. $C_{14}H_{12}D_6N_2O_4$ (284.34), LC-MS (ESI): *m/z* calcd for $C_{14}H_{12}D_6N_2O_4$ $(M+H)^+$ 285.16, found 285.2.

(*R*)-4-oxo-4-((1-oxo-1-(((phenyl-d$_5$)methyl)amino)propan-2-yl)amino)butanoic-2,2,3,3-d$_4$ acid, (*R*)-16. White solid. Yield: 96% (3.28 g); Melting point: 129.7–131.3°C; TLC: R_f = 0.34 (S_2); UPLC (purity > 99%): t_R = 3.12 min. $C_{14}H_9D_9N_2O_4$ (287.36), LC-MS (ESI): *m/z* calcd for $C_{14}H_9D_9N_2O_4$ $(M+H)^+$ 288.18, found 288.2.

(*R*)-4-oxo-4-((1-oxo-1-(((phenyl-d$_5$)methyl-d$_2$)amino)propan-2-yl)amino)butanoic-2,2,3,3-d$_4$ acid, (*R*)-17. White solid. Yield: 94% (3.26 g); Melting point: 129.1–131.2°C; TLC: R_f = 0.34 (S_2); UPLC (purity > 99%): t_R = 3.12 min. $C_{14}H_7D_{11}N_2O_4$ (289.38), LC-MS (ESI): *m/z* calcd for $C_{14}H_7D_{11}N_2O_4$ $(M+H)^+$ 290.20, found 290.2.

(*R*)-4-((1-((2-fluorobenzyl)amino)-1-oxopropan-2-yl)amino)-4-oxobutanoic-2,2,3,3-d$_4$ acid, (*R*)-18. White solid. Yield: 93% (3.19 g); Melting point: 131.2–132.6°C; TLC: R_f = 0.36 (S_2); UPLC (purity = 95.7%): t_R = 3.32 min. $C_{14}H_{13}D_4FN_2O_4$ (300.32), LC-MS (ESI): *m/z* calcd for $C_{14}H_{13}D_4FN_2O_4$ $(M+H)^+$ 301.14, found 301.2.

(*R*)-4-((1-((2-fluorophenyl)methyl-d$_2$)amino)-1-oxopropan-2-yl)amino)-4-oxobutanoic-2,2,3,3-d$_4$ acid, (*R*)-19. White solid. Yield: 95% (3.44 g); Melting point: 131.3–132.5°C; TLC: R_f = 0.36 (S_2); UPLC (purity > 99%): t_R = 3.34 min. $C_{14}H_{11}D_6FN_2O_4$ (302.15), LC-MS (ESI): *m/z* calcd for $C_{14}H_{11}D_6FN_2O_4$ $(M+H)^+$ 303.34, found 303.3.

(*R,S*)-4-((1-(benzylamino)-1-oxopropan-2-yl)amino)-4-oxobutanoic-2,2,3,3-d$_4$ acid, (*R,S*)-13. White solid. Yield: 95% (3.22 g); Melting point: 89.3–90.9°C; TLC: R_f = 0.34 (S_2); UPLC (purity > 99%): t_R = 3.12 min. $C_{14}H_{14}D_4N_2O_4$ (282.33), LC-MS (ESI): *m/z* calcd for $C_{14}H_{14}D_4N_2O_4$ $(M+H)^+$ 283.15, found 283.2.

(*R,S*)-4-oxo-4-((1-oxo-1-(((phenyl-d$_5$)methyl)amino)propan-2-yl)amino)butanoic acid, (*R,S*)-14. White solid. Yield: 97% (3.26 g); Melting point: 89.4–91.4°C; TLC: R_f = 0.35 (S_2); UPLC (purity > 99%): t_R = 3.13 min. $C_{14}H_{13}D_5N_2O_4$ (283.34), LC-MS (ESI): *m/z* calcd for $C_{14}H_{13}D_5N_2O_4$ $(M+H)^+$ 284.15, found 284.2.

(*R,S*)-4-oxo-4-((1-oxo-1-((phenylmethyl-d$_2$)amino)propan-2-yl)amino)butanoic-2,2,3,3-d$_4$ acid, (*R,S*)-15. White solid. Yield: 94% (3.19 g); Melting point: 89.1–90.6°C; TLC: R_f = 0.34 (S_2); UPLC (purity > 99%): t_R = 3.12 min. $C_{14}H_{12}D_6N_2O_4$ (284.34), LC-MS (ESI): *m/z* calcd for $C_{14}H_{12}D_6N_2O_4$ $(M+H)^+$ 285.16, found 285.1.

(*R,S*)-4-oxo-4-((1-oxo-1-(((phenyl-d$_5$)methyl)amino)propan-2-yl)amino)butanoic-2,2,3,3-d$_4$ acid, (*R,S*)-16. White solid. Yield: 92% (3.14 g); Melting point: 89.1–90.6°C; TLC: R_f = 0.34 (S_2); UPLC (purity > 99%): t_R = 3.12 min. $C_{14}H_9D_9N_2O_4$ (287.36), LC-MS (ESI): *m/z* calcd for $C_{14}H_9D_9N_2O_4$ $(M+H)^+$ 288.18, found 288.2.

(*R,S*)-4-oxo-4-((1-oxo-1-(((phenyl-d$_5$)methyl-d$_2$)amino)propan-2-yl)amino)butanoic-2,2,3,3-d$_4$ acid, (*R,S*)-17. White solid. Yield: 95% (3.29 g); Melting point: 89.4–90.7°C; TLC: R_f = 0.34 (S_2); UPLC (purity > 99%): t_R = 3.13 min. $C_{14}H_7D_{11}N_2O_4$ (289.38), LC-MS (ESI): *m/z* calcd for $C_{14}H_7D_{11}N_2O_4$ $(M+H)^+$ 290.20, found 290.3.

(*R,S*)-4-((1-((2-fluorobenzyl)amino)-1-oxopropan-2-yl)amino)-4-oxobutanoic-2,2,3,3-d$_4$ acid, (*R,S*)-18. White solid. Yield: 93% (3.14 g); Melting point: 89.3–90.5°C; TLC: R_f = 0.36 (S_2); UPLC (purity > 99%): t_R = 3.32 min. $C_{14}H_{13}D_4FN_2O_4$ (300.32), LC-MS (ESI): *m/z* calcd for $C_{14}H_{13}D_4FN_2O_4$ $(M+H)^+$ 301.14, found 301.2.

(*R,S*)-4-((1-(((2-fluorophenyl)methyl-d$_2$)amino)-1-oxopropan-2-yl)amino)-4-oxobutanoic-2,2,3,3-d$_4$ acid, (*R,S*)-19. White solid. Yield: 96% (3.47 g); Melting point: 89.4–90.6°C; TLC: R_f = 0.36 (S_2); UPLC (purity > 99%): t_R = 3.33 min. $C_{14}H_{11}D_6FN_2O_4$ (302.15), LC-MS (ESI): *m/z* calcd for $C_{14}H_{11}D_6FN_2O_4$ $(M+H)^+$ 303.34, found 303.2

Procedure for the preparation of the final compounds d$_4$-(*R*)-AS-1, d$_5$-(*R*)-AS-1, d$_6$-(*R*)-AS-1, d$_9$-(*R*)-AS-1, d$_{11}$-(*R*)-AS-1, d$_4$-(*R*)-AS-7, d$_6$-(*R*)-AS-7 and d$_4$-(*R,S*)-AS-1, d$_5$-(*R,S*)-AS-1, d$_6$-(*R,S*)-AS-1, d$_9$-(*R,S*)-AS-1, d$_{11}$-(*R,S*)-AS-1, d$_4$-(*R,S*)-AS-7, d$_6$-(*R,S*)-AS-7. To a suspension of succinamic acids (*R*)-13–(*R*)-19 and (*R,S*)-13–(*R,S*)-19 (10 mmol, 1 eq) in 1,4-dioxane (50 mL) $ZnCl_2$ (1.35 g, 10 mmol, 1 eq) was added, and the mixture was heated to 70°C. Afterwards, a solution of HMDS (1.62 g, 2.1 mL, 10 mmol, 1.0 eq) in dry 1,4-dioxane (5 mL) was added dropwise over 30 min. The reaction mixture was heated at 70°C an additional 1.5 hours and then concentrated under reduced pressure. The resulting oil residue was dissolved in DCM (50 mL), and 10% hydrochloric acid was added. The aqueous layer was extracted with DCM (3 × 50 mL), dried over Na_2SO_4, and concentrated in a vacuum. The final

compounds were purified by column chromatography using a DCM/MeOH (9:0.3, *v/v*) solvent system and crystallized from 2-propanol.

(*R*)-*N*-benzyl-2-(2,5-dioxopyrrolidin-1-yl-3,3,4,4-d$_4$)propanamide, d$_4$-(*R*)-AS-1. White solid. Yield: 89% (2.34 g); Melting point: 138.2–138.9°C; TLC: R_f = 0.39 (S_1); UPLC (purity > 99%): t_R = 3.79 min. $C_{14}H_{12}D_4N_2O_3$ (264.32), LC-MS (ESI): *m/z* calcd for $C_{14}H_{12}D_4N_2O_3$ $(M+H)^+$ 265.14, found 265.2. UPLC/HRMS (purity > 99%): t_R = 4.18 min. HRMS (ESI-QTOF): m/z calcd for $C_{14}H_{12}D_4N_2O_3$ $(M+H)^+$ 265.1446, found 265.1528. ^1H NMR (500 MHz, $CDCl_3$) δ 1.57 (d, *J*=7.2 Hz, 3H), 4.39 (d, *J*=5.7 Hz, 2H), 4.77 (q, *J*=7.2 Hz, 1H), 6.41 (br s, 1H), 7.22–7.26 (m, 3H), 7.29–7.32 (m, 2H). ^{13}C NMR (126 MHz, $CDCl_3$) δ 14.5, 25.7, 34.0, 43.8, 49.8, 127.7, 128.8, 137.9, 168.6, 177.0. Deuterium incorporation: > 99% D by ^1H NMR. Chiral SFC: > 99% *ee* (t_R = 3.845 min).

(*R*)-2-(2,5-dioxopyrrolidin-1-yl)-*N*-((phenyl-d$_5$)methyl)propanamide, d$_5$-(*R*)-AS-1. White solid. Yield: 87% (2.30 g); Melting point: 138.9–140.2°C; TLC: R_f = 0.43 (S_1); UPLC (purity > 99%): t_R = 3.80 min. $C_{14}H_{11}D_5N_2O_3$ (265.32), LC-MS (ESI): *m/z* calcd for $C_{14}H_{11}D_5N_2O_3$ $(M+H)^+$ 266.15, found 266.2. UPLC/HRMS (purity > 99%): t_R = 4.16 min. HRMS (ESI-QTOF): m/z calcd for $C_{14}H_{11}D_5N_2O_3$ $(M+H)^+$ 266.1508, found 266.1593. ^1H NMR (500 MHz, $CDCl_3$) δ 1.58 (d, *J*=7.2 Hz, 3H), 2.67–2.71 (m, 4H), 4.42 (d, *J*=5.4 Hz, 2H), 4.76–4.79 (m, 1H), 6.42 (br s, 1H). ^{13}C NMR (126 MHz, $CDCl_3$) δ 14.5, 33.9, 49.8, 127.1, 127.3, 128.1, 137.7, 168.7, 177.0. Deuterium incorporation: > 99% D by ^1H NMR. Chiral SFC: > 99% *ee* (t_R = 3.879 min).

(*R*)-2-(2,5-dioxopyrrolidin-1-yl-3,3,4,4-d$_4$)-*N*-(phenylmethyl-d$_2$)propanamide, d$_6$-(*R*)-AS-1. White solid. Yield: 86% (2.28 g); Melting point: 138.3–139.1°C; TLC: R_f = 0.39 (S_1); UPLC (purity > 99%): t_R = 3.78 min. $C_{14}H_{10}D_6N_2O_3$ (266.33), LC-MS (ESI): *m/z* calcd for $C_{14}H_{10}D_6N_2O_3$ $(M+H)^+$ 267.15, found 267.2. UPLC/HRMS (purity > 99%): t_R = 4.17 min. HRMS (ESI-QTOF): m/z calcd for $C_{14}H_{10}D_6N_2O_3$ $(M+H)^+$ 267.1571, found 267.1613. ^1H NMR (500 MHz, $CDCl_3$) δ 1.58 (d, *J*=7.5 Hz, 3H), 4.78 (q, *J*=7.5 Hz, 1H), 6.35 (br s, 1H), 7.22–7.28 (m, 3H), 7.30–7.33 (m, 2H). ^{13}C NMR (126 MHz, $CDCl_3$) δ 14.5, 25.7, 34.0, 49.8, 115.3, 115.5, 124.5, 129.4, 129.5, 130.2, 168.8, 177.0. Deuterium incorporation: > 99% D by ^1H NMR. Chiral SFC: > 99% *ee* (t_R = 3.820 min).

(*R*)-2-(2,5-dioxopyrrolidin-1-yl-3,3,4,4-d$_4$)-*N*-((phenyl-d$_5$)methyl)propanamide, d$_9$-(*R*)-AS-1. White solid. Yield: 85% (2.29 g); Melting point: 139.3–140.7°C; TLC: R_f = 0.44 (S_1); UPLC (purity > 99%): t_R = 3.82 min. $C_{14}H_7D_9N_2O_3$ (269.35), LC-MS (ESI): *m/z* calcd for $C_{14}H_7D_9N_2O_3$ $(M+H)^+$ 270.17, found 270.1. UPLC/HRMS (purity > 99%): t_R = 4.15 min. HRMS (ESI-QTOF): m/z calcd for $C_{14}H_7D_9N_2O_3$ $(M+H)^+$ 270.1759, found 270.1851. ^1H NMR (500 MHz, $CDCl_3$) δ 1.58 (d, *J*=7.2 Hz, 3H), 4.41 (d, *J*=5.7 Hz, 2H), 4.79 (q, *J*=7.2 Hz, 1H), 6.42 (br s, 1H). ^{13}C NMR (126 MHz, $CDCl_3$) δ 14.6, 34.0, 43.8, 49.8, 127.1, 127.3, 128.1, 137.7, 168.7, 177.0. Deuterium incorporation: > 99% D by ^1H NMR. Chiral SFC: > 99% *ee* (t_R = 3.803 min).

(*R*)-2-(2,5-dioxopyrrolidin-1-yl-3,3,4,4-d$_4$)-*N*-((phenyl-d$_5$)methyl-d$_2$)propanamide, d$_{11}$-(*R*)-AS-1. White solid. Yield: 83% (2.40 g); Melting point: 138.1–139.0°C; TLC: R_f = 0.39 (S_1); UPLC (purity > 99%): t_R = 3.80 min. $C_{14}H_5D_{11}N_2O_3$ (271.36), LC-MS (ESI): *m/z* calcd for $C_{14}H_5D_{11}N_2O_3$ $(M+H)^+$ 272.19, found 272.2. UPLC/HRMS (purity = 98.53%): t_R = 4.15 min. HRMS (ESI-QTOF): m/z calcd for $C_{14}H_5D_{11}N_2O_3$ $(M+H)^+$ 272.1885, found 272.1932. ^1H NMR (500 MHz, $CDCl_3$) δ 1.59 (d, *J*=7.5 Hz, 3H), 4.79 (q, *J*=7.5 Hz, 1H), 6.32 (br s, 1H). ^{13}C NMR (126 MHz, $CDCl_3$) δ 14.6, 25.7, 34.0, 49.9, 127.2, 127.4, 128.1, 137.6, 168.6, 177.0. Deuterium incorporation: > 99% D by ^1H NMR. Chiral SFC: > 99% *ee* (t_R = 3.787 min).

(*R*)-2-(2,5-dioxopyrrolidin-1-yl-3,3,4,4-d$_4$)-*N*-(2-fluorobenzyl)propanamide, d$_4$-(*R*)-AS-7. White solid. Yield: 84% (2.36 g); Melting point: 157.2–157.5°C; TLC: R_f = 0.44 (S_1); UPLC (purity > 99%): t_R = 4.01 min. $C_{14}H_{11}D_4FN_2O_3$ (282.31), LC-MS (ESI): *m/z* calcd for $C_{14}H_{11}D_4FN_2O_3$ $(M+H)^+$ 283.13, found 283.2. UPLC/HRMS (purity > 99%): t_R = 4.34 min. HRMS (ESI-QTOF): m/z calcd for $C_{14}H_{11}D_4FN_2O_3$ $(M+H)^+$ 283.1351, found 283.1404. ^1H NMR (500 MHz, $CDCl_3$) δ 1.57 (d, *J*=7.3 Hz, 3H), 4.38–4.52 (m, 2H), 4.76 (q, *J*=7.3 Hz, 1H), 6.49 (br s, 1H), 6.98–7.04 (m, 1H), 7.08–7.11 (m, 1H), 7.20–7.24 (m, 1H), 7.30–7.31 (m, 1H). ^{13}C NMR (126 MHz, $CDCl_3$) δ 14.5, 25.7, 34.0, 38.0, 49.8, 115.4, 124.6, 129.4 129.4, 130.2, 168.8, 177.0. Deuterium incorporation: > 99% D by ^1H NMR. Chiral SFC: > 99% *ee* (t_R = 3.094 min).

(*R*)-2-(2,5-dioxopyrrolidin-1-yl-3,3,4,4-d$_4$)-*N*-((2-fluorophenyl)methyl-d$_2$)propanamide, d$_6$-(*R*)-AS-7. White solid. Yield: 84% (2.39 g); Melting point: 157.2–157.7°C; TLC: R_f = 0.44 (S_1); UPLC (purity > 99%): t_R = 3.79 min. $C_{14}H_9D_6FN_2O_3$ (284.32), LC-MS (ESI): *m/z* calcd for $C_{14}H_9D_6FN_2O_3$ $(M+H)^+$ 285.14, found

285.2. UPLC/HRMS (purity > 99%): t_R = 4.18 min. HRMS (ESI-QTOF): m/z calcd for $C_{14}H_9D_6FN_2O_3$ $(M+H)^+$ 285.1477, found 285.1564. ^{1}H NMR (500 MHz, $CDCl_3$) δ 1.57 (d, *J*=7.2 Hz, 3H), 4.76 (q, *J*=7.3 Hz, 1H), 6.44 (br s, 1H), 7.01 (ddd, *J*=10.2, 8.2, 1.0 Hz, 1H), 7.09 (td, *J*=7.6, 1.2 Hz, 1H), 7.21–7.26 (m, 1H), 7.31 (td, *J*=7.6, 1.7 Hz, 1H). ^{13}C NMR (126 MHz, $CDCl_3$) δ 14.6, 28.3, 43.9, 49.8, 127.7, 127.8, 128.8, 137.9, 168.8, 177.0. Deuterium incorporation: > 99% D by ^{1}H NMR. Chiral SFC: > 99% *ee* (t_R = 3.112 min).

(*R*,*S*)-*N*-benzyl-2-(2,5-dioxopyrrolidin-1-yl-3,3,4,4-d_4)propanamide, d_4-(*R*,*S*)-AS-1. White solid. Yield: 85% (2.23 g); Melting point: 83.4–84.2°C; TLC: R_f = 0.39 (S_1); UPLC (purity > 99%): t_R = 3.78 min. $C_{14}H_{12}D_4N_2O_3$ (264.32), LC-MS (ESI): *m/z* calcd for $C_{14}H_{12}D_4N_2O_3$ $(M+H)^+$ 265.14, found 265.2. ^{1}H NMR (500 MHz, $CDCl_3$) δ 1.55–1.59 (m, 3H), 4.40 (d, *J*=5.4 Hz, 2H), 4.76 (qd, *J*=7.4, 1.7 Hz, 1H), 6.41 (br s, 1H), 7.22–7.27 (m, 3H), 7.29–7.33 (m, 2H). ^{13}C NMR (126 MHz, $CDCl_3$) δ 14.5, 25.7, 34.0, 49.8, 127.7, 128.8, 137.9, 168.6, 177.0. Deuterium incorporation: > 99% D by ^{1}H NMR.

(*R*,*S*)-2-(2,5-dioxopyrrolidin-1-yl)-*N*-((phenyl-d_5)methyl)propanamide, d_5-(*R*,*S*)-AS-1. White solid. Yield: 84% (2.22 g); Melting point: 84.4–86.1°C; TLC: R_f = 0.43 (S_1); UPLC (purity > 99%): t_R = 3.82 min. $C_{14}H_{11}D_5N_2O_3$ (265.32), LC-MS (ESI): *m/z* calcd for $C_{14}H_{11}D_5N_2O_3$ $(M+H)^+$ 266.15, found 266.2. ^{1}H NMR (500 MHz, $CDCl_3$) δ 1.57 (d, *J*=7.5 Hz, 3H), 2.68 (s, 4H), 4.40 (d, *J*=5.7 Hz, 2H), 4.77 (q, *J*=7.2 Hz, 1H), 6.42 (br s, 1H). ^{13}C NMR (126 MHz, $CDCl_3$) δ 14.5, 25.7, 43.8, 49.8, 127.7, 128.9, 137.9, 168.6, 177.0. Deuterium incorporation: > 99% D by ^{1}H NMR.

(*R*,*S*)-2-(2,5-dioxopyrrolidin-1-yl-3,3,4,4-d_4)-*N*-(phenylmethyl-d_2)propanamide, d_6-(*R*,*S*)-AS-1. White solid. Yield: 82% (2.18 g); Melting point: 84.5–86.2°C; TLC: R_f = 0.39 (S_1); UPLC (purity > 99%): t_R = 3.78 min. $C_{14}H_{10}D_6N_2O_3$ (266.33), LC-MS (ESI): *m/z* calcd for $C_{14}H_{10}D_6N_2O_3$ $(M+H)^+$ 267.15, found 267.3. ^{1}H NMR (500 MHz, $CDCl_3$) δ 1.58 (d, *J*=7.2 Hz, 3H), 4.77 (q, *J*=7.5 Hz, 1H), 6.35 (br s, 1H), 7.23–7.27 (m, 3H), 7.30–7.33 (m, 2H). ^{13}C NMR (126 MHz, $CDCl_3$) δ 14.5, 34.0, 49.8, 124.5, 129.4, 129.5, 137.8, 168.8, 177.0. Deuterium incorporation: > 99% D by ^{1}H NMR.

(*R*,*S*)-2-(2,5-dioxopyrrolidin-1-yl-3,3,4,4-d_4)-*N*-((phenyl-d_5)methyl)propanamide, d_9-(*R*,*S*)-AS-1. White solid. Yield: 87% (2.34 g); Melting point: 84.1–85.5°C; TLC: R_f = 0.40 (S_1); UPLC (purity > 99%): t_R = 3.80 min. $C_{14}H_7D_9N_2O_3$ (269.35), LC-MS (ESI): *m/z* calcd for $C_{14}H_7D_9N_2O_3$ $(M+H)^+$ 270.17, found 270.1. ^{1}H NMR (500 MHz, $CDCl_3$) δ 1.57 (d, *J*=7.2 Hz, 3H), 4.40 (d, *J*=5.7 Hz, 2H), 4.77 (q, *J*=7.2 Hz, 1H), 6.40 (br s, 1H). ^{13}C NMR (126 MHz, $CDCl_3$) δ 14.6, 34.0, 43.8, 49.8, 127.1, 127.3, 128.1, 137.7, 168.7, 177.0. Deuterium incorporation: > 99% D by ^{1}H NMR.

(*R*,*S*)-2-(2,5-dioxopyrrolidin-1-yl-3,3,4,4-d_4)-*N*-((phenyl-d_5)methyl-d_2)propanamide, d_{11}-(*R*,*S*)-AS-1. White solid. Yield: 82% (2.37 g); Melting point: 84.2–86.3°C; TLC: R_f = 0.39 (S_1); UPLC (purity > 99%): t_R = 3.80 min. $C_{14}H_5D_{11}N_2O_3$ (271.36), LC-MS (ESI): *m/z* calcd for $C_{14}H_5D_{11}N_2O_3$ $(M+H)^+$ 272.19, found 272.2. ^{1}H NMR (500 MHz, $CDCl_3$) δ 1.58 (d, *J*=7.5 Hz, 3H), 4.79 (q, *J*=7.5 Hz, 1H), 6.32 (br s, 1H). ^{13}C NMR (126 MHz, $CDCl_3$) δ 14.6, 34.0, 49.9, 127.2, 127.4, 128.1, 137.5, 168.6, 177.0. Deuterium incorporation: > 99% D by ^{1}H NMR.

(*R*,*S*)-2-(2,5-dioxopyrrolidin-1-yl-3,3,4,4-d_4)-*N*-(2-fluorobenzyl)propanamide, d_4-(*R*,*S*)-AS-7. White solid. Yield: 85% (2.38 g); Melting point: 98.4–99.7°C; TLC: R_f = 0.44 (S_1); UPLC (purity > 99%): t_R = 3.95 min. $C_{14}H_{11}D_4FN_2O_3$ (282.31), LC-MS (ESI): *m/z* calcd for $C_{14}H_{11}D_4FN_2O_3$ $(M+H)^+$ 283.13, found 283.2. ^{1}H NMR (500 MHz, $CDCl_3$) δ 1.57 (d, *J*=7.5 Hz, 3H), 4.40–4.50 (m, 2H), 4.76 (q, *J*=7.2 Hz, 1H), 6.52 (br s, 1H), 6.98–7.03 (m, 1H), 7.08 (td, *J*=7.5, 1.0 Hz, 1H), 7.20–7.24 (m, 1H), 7.30 (td, *J*=7.6, 1.7 Hz, 1H). ^{13}C NMR (126 MHz, $CDCl_3$) δ 14.5, 34.0, 37.9, 49.8, 115.5, 124.5, 129.4, 130.2, 168.8, 177.0. Deuterium incorporation: > 99% D by ^{1}H NMR.

(*R*,*S*)-2-(2,5-dioxopyrrolidin-1-yl-3,3,4,4-d_4)-*N*-((2-fluorophenyl)methyl-d_2)propanamide, d_6-(*R*,*S*)-AS-7. White solid. Yield: 84% (2.35 g); Melting point: 98.3–99.8°C; TLC: R_f = 0.44 (S_1); UPLC (purity > 99%): t_R = 3.98 min. $C_{14}H_9D_6FN_2O_3$ (284.32), LC-MS (ESI): *m/z* calcd for $C_{14}H_9D_6FN_2O_3$ $(M+H)^+$ 285.14, found 285.2. ^{1}H NMR (500 MHz, $CDCl_3$) δ 1.57 (d, *J*=7.2 Hz, 3H), 4.76 (q, *J*=7.3 Hz, 1H), 6.44 (br s, 1H), 7.01 (ddd, *J*=10.2, 8.2, 1.0 Hz, 1H), 7.09 (td, *J*=7.6, 1.2 Hz, 1H), 7.22–7.26 (m, 1H), 7.31 (td, *J*=7.6, 1.7 Hz, 1H). ^{13}C NMR (126 MHz, $CDCl_3$) δ 14.6, 28.3, 43.9, 49.8, 127.7, 127.8, 128.8, 137.9, 168.8, 177.0. Deuterium incorporation: > 99% D by ^{1}H NMR.

Animal studies

General Information. All animal experiments were performed in accordance with Polish regulations and the European Union Directive of 22 September 2010 (2010/63/EU) on the protection of animals used for scientific purposes. Animals were obtained from accredited breeders and housed in groups at controlled laboratory conditions (temperature 21–24°C, relative humidity 45–65%, and artificial 12-h light/dark cycle) with unrestricted access to food and tap water. All procedures were approved by the Local Ethics Committees for Experiments on Animals in Poland.

Pharmacokinetic studies. Experiments were carried out on adult male CD-1 mice (28–32 g) obtained from the Faculty of Pharmacy, Jagiellonian University Medical College, (Krakow, Poland). Experimental protocols were approved by the I Local Ethical Committee for Experiments on Animals of the Jagiellonian University in Krakow, Poland (approvals no 270/2019 and 412/2020). The animals were fasted overnight prior to drug administration but had free access to water. Compounds were dissolved in a mixture of DMSO, PEG400, water for injection (1:4:5, *v*/*v*/*v*), and given *i.p.* at doses of 20 mg/kg and 40 mg/kg (n=3–4 per time point). **(*R*)-AS-7** and **d_6-(*R*)-AS-7** were also administered *p.o.* at dose of 40 mg/kg in the same formulation. Blood samples were collected at different time points following compound administration and allowed to clot at room temperature for 20 min. Moreover, brains were removed from skulls and washed with 0.9% NaCl. The blood samples were centrifuged for 10 min at the speed of 8000 rpm (Eppendorf miniSpin centrifuge, Germany). The obtained serum and brains were stored at –80°C until analysis.

Bioanalytical method. The concentrations of the compounds **(*R*)-AS-1**, **d_4-(*R)*-AS-1**, **d_6-(*R*)-AS-1**, **d_9-(*R*)-AS-1**, **d_{11}-(*R*)-AS-1**, as well as **(*R*)-AS-7**, **d_4-(*R*)-AS-7**, **d_6-(*R*)-AS-7** in murine serum and brain homogenates were measured using high-performance liquid chromatography coupled with mass spectrometry (HPLC-MS/MS). Analytes were separated on the Hypersil Gold^{TM} C18 analytical column (2.1 × 50 mm, 3 µm, Thermo Scientific, USA) using an AB Sciex Exion LC AC HPLC system (Danaher Corporation, USA). The oven temperature was set at 40°C. The initial mobile phase composition was 95% A (0.1% formic acid in water) and 5% B (0.1% formic acid in MeCN) for the first 2 min with a linear gradient to 5% A in the next 2 min, then isocratic mode for 2 min with the following rapid change back to 95% A in 0.1 min. The remaining time of elution was set at 95% A. The whole HPLC operation lasted 10 min, and the flow rate was set at the 0.4 mL/min. Detection was performed on the triple quadrupole mass spectrometer Sciex QTRAP 4500 (Danaher Corporation, USA) and electrospray ionization (ESI) in the positive ion mode was used for ion production. The mass spectrometer operated in unit resolution mode, monitoring the ion transitions shown in **Tables S9** and **S10**. The first pair was used for quantitative analysis, while the second pair was used to confirm identity. Valsartan was used as an internal standard (IS) for which the m/z 436 to 207 (CE=42 eV) was monitored. The parameters for the ion pathway shown in **Tables S9** and **S10** were optimized by continuous infusion (7 µL/min) of the analyte solution directly into the mass spectrometer using a syringe pump. Optimal ion source settings included an ion spray voltage of 5500V and a gas temperature of 500°C. The curtain gas pressure was set to 20 psi, and the collision gas was set to an average value. Data collection and integration were performed using Analyst software version 1.7. Calibration curves were prepared in the appropriate matrices (serum or brain homogenate) ranging from 0.001 to 2 µg/mL in serum, and from 0.005 to 10 µg/g in brain. The calibration curves were constructed by plotting the ratio of the peak area of the studied compound to IS versus drug concentration and generated by weighted ($1/x \cdot x$) linear regression analysis. The calculated precision and accuracy values fell within the range recommended in FDA guidelines for the validation of bioanalytical methods. No matrix effect was observed that could significantly influence the accuracy of the measurements. The analyzed compounds were stable during the sample preparation process and under autosampler conditions.

Table S9. Monitored transitions of parent ion/fragment ion and optimal parameters for the ion pathway of **(*R*)-AS-1**, **d_4-(*R*)-AS-1**, **d_6-(*R*)-AS-1**, **d_9-(*R*)-AS-1** and **d_{11}-(*R*)-AS-1**.

**Compound**	**(*R*)-AS-1**		**d_4-(*R*)-AS-1**		**d_6-(*R*)-AS-1**		**d_9-(*R*)-AS-1**		**d_{11}-(*R*)-AS-1**	
Monitored transition	**261/126**	**261/154**	**265/130**	**265/158**	**267/130**	**267/158**	**270/130**	**270/158**	**272/130**	**272/158**
Declustering potential (DP)	81	81	76	76	51	51	81	81	86	86
Entrance potential (EP)	10	10	10	10	10	10	10	10	10	10
Collision cell exit potential (CXP)	10	8	6	6	22	4	10	12	6	10
Collision Energy (CE)	25	17	17	17	21	21	25	29	27	19

Table S10. Monitored transitions of parent ion/fragment ion and optimal parameters for the ion pathway of **(*R*)-AS-7**, **d_4-(*R*)-AS-7** and **d_6-(*R*)-AS-7**.

**Compound**	**(*R*)-AS-7**		**d_4-(*R*)-AS-7**		**d_6-(*R*)-AS-7**	
Monitoredtransition	**279/126**	**279/109**	**283/130**	**283/109**	**285/130**	**285/111**
Declustering potential (DP)	71	71	96	96	96	96
Entrance potential (EP)	10	10	10	10	10	10
Collision cell exit potential (CXP)	10	8	10	16	6	8
Collision Energy (CE)	25	41	31	31	31	37

Preparation of calibration standards. Stock solutions of the compounds **(*R*)-AS-1**, **d_4-(*R*)-AS-1**, **d_6-(*R*)-AS-1**, **d_9-(*R*)-AS-1**, **d_{11}-(*R*)-AS-1**, as well as **(*R*)-AS-7**, **d_4-(*R*)-AS-7**, **d_6-(*R*)-AS-7** at a concentration of 1 mg/mL were prepared in methanol. Then, working solutions were prepared by serial dilutions at concentrations of 0.01; 0.1; 0.25; 0.5; 1; 5; 10 and 20 µg/mL (the effective concentrations of the calibration samples were 0.001; 0.01; 0.025; 0.05; 0.1; 0.5; 1 and 2 µg/mL). For the preparation of calibration curves, 45 µL of the appropriate matrix (serum or brain homogenate) was added to 5 µL of the working solution at a specified concentration of the analyte and mixed for 10 sec. The samples were then deproteinized using a solution of 0.1% formic acid in MeCN with the addition of an IS (1:3, *v*/*v*), shaken for 10 min on a shaker (IKA Vibrax VXR, Germany), and centrifuged for 5 min at a speed of 8000 × g (Eppendorf miniSpin centrifuge, Germany).

Sample preparation. Brains were homogenized in distilled water at a ratio of 1:4 (*w*/*v*) using the LabGen 125 homogenizer (Cole Parmer, UK). Brain homogenate or serum samples (50 µL) were deproteinized with a solution of 0.1% formic acid in MeCN with the addition of an IS (1:3, *v*/*v*). The samples were then shaken for 10 min on a shaker (IKA Vibrax VXR, Germany) and centrifuged for 5 min at a speed of 8000 × g (Eppendorf miniSpin centrifuge, Germany). The supernatant was transferred directly to chromatographic vials. Samples with the concentrations above the upper limit of quantification were diluted with the blank matrix (serum or brain homogenate). The autosampler temperature was set to 15°C, and 1 µL was injected onto the analytical column.

Pharmacokinetic data analysis. Serum and brain versus time profiles were analyzed by the non-compartmental approach. The maximum concentration (C_{max}) and the time to reach maximum concentration (t_{max}) were obtained directly from individual concentration versus time profiles. The linear trapezoidal rule was employed to calculate the area under the concentration versus time curve (AUC) from the time of dosing to infinity ($AUC_{0-\infty}$). The terminal slope (λ_z) was estimated by the linear regression and the terminal half-life ($t_{1/2\lambda z}$) was calculated as $\ln 2/\lambda_z$. The volume of distribution based on the terminal phase (V_z/F) was calculated as: Dose/(λ_z $AUC_{0-\infty}$) and clearance (CL/F) was obtained from the equation: Dose/$AUC_{0-\infty}$, where F is fraction of dose absorbed. The mean residence time (MRT) was calculated as: $AUMC_{0-\infty}/AUC_{0-\infty}$, where AUMC is the area under the first moment curve.

Acute seizure models and rotarod test. Experiments were carried out on adult male CD-1 mice (22–26 g), obtained from the Faculty of Pharmacy, Jagiellonian University Medical College, (Krakow, Poland). Experimental protocols were approved by the I Local Ethics Committee for Experiments on Animals in Krakow, Poland (approvals no. 463/2020 and 512A/2020). Compounds were dissolved in a mixture of DMSO, PEG400, water for injection (1:4:5, *v*/*v*/*v*), and administered *i.p.* and *p.o.* (**(*R*)-AS-1**, **d_6-(*R*)-AS-7**), 30 min and 2 h before the given test in a volume of 10 mL/kg (NOTE: The vehicle itself was inactive in MES and 6 Hz (44 mA) seizure models and did not affect motor coordination in the rotarod assay – data provided by the NIH ETSP). Fresh solutions were prepared daily for each experimentation session. The ED_{50} or TD_{50} parameters were estimated based on the results obtained in 3–4 groups of animals consisting of 6 mice. Detailed *in vivo* procedures, including the MES test, the 6 Hz (32 and 44 mA) tests, the *sc*PTZ test, and the rotarod test for acute neurological toxicity, have been described previously.[6] ED_{50} and TD_{50} values with 95% confidence limits were calculated by probit analysis.[19] PIs for the compounds investigated and reference ASMs were calculated by dividing the TD_{50} value, as determined in the rotarod test, by the respective ED_{50} value, as determined in the MES, *sc*PTZ, or 6 Hz (32 mA or 44 mA) tests.

6 Hz (32 mA) model and minimal motor impairment test in male and female C57 WT mice. Male and female C57BL/6J (WT) mice were acquired from the Jackson Labs at 4-5 weeks of age (male and female) and allowed to age within the University of Washington vivarium until testing at 3–4 months-old. All animals had free access to food (PicoLab Rodent Diet 20–5053; irradiated chow) and water (reverse osmosis purified, chlorinated, water provided in hydropacs or as automatic rack water), except during the periods of behavioral manipulation. Animals were housed five per cage in autoclaved individually ventilated cages (Allentown Inc., Allentown, NJ, USA) with autoclaved corn cob bedding (The Andersons, Maumee, OH, USA) and free access to nesting materials (Nestlets; Ancare, Bellmore, NY, USA).[20] Mice were maintained specific-pathogen free via a rodent health-monitoring program and

are certified to be free of specific rodent pathogens previously detailed.[20] All testing is conducted between the hours of 9 am and 4 pm. Animals were maintained on a 14:10 light:dark cycle in the University of Washington vivarium (lights on at 6 am, off at 8 pm). The animal house was maintained under controlled ambient temperature of 21 ± 3°C, and relative humidity between 30-70%. All animal use was approved by the University of Washington Institutional Animal Care and Use Committee (IACUC; protocol #4387-01 under US OLAW Assurance #A3464-01). All behavioral evaluations were conducted by an experimenter blinded to treatment condition.

6 Hz (32 mA) model. Mice were challenged 0.5 h after *i.p.* compound administration with a 1.5 × CC50 current (32 mA equivalent current) for 3 sec delivered through corneal electrodes to elicit a typical seizure. The 6 Hz seizure is characterized by an initial momentary stun followed immediately by forelimb clonus, twitching of the vibrissae, and Straub tail.[21] Animals not displaying these behaviors within the immediate (5-10 second) period post-stimulation were considered “protected”.

Minimal motor impairment test. The fixed-speed rotarod was used to identify minimal motor impairment (MMI) in mice immediately prior to seizure induction.[22–24] When a normal mouse is placed on a rod that rotates at a constant speed of 6 rpm, the animal can maintain its equilibrium for long periods of time. If a mouse fell off the rotarod three times during the 1-min trial period, it was considered “impaired”. Investigational compound testing. **d$_6$-(*R*)-AS-7** was administered at two doses (15 and 30 mg/kg, *i.p.*) and tested for anticonvulsant efficacy 0.25, 0.5 and 1.0 h later in the 6 Hz (32 mA) test to define a time and dose-related effect on seizure protection. The compound was administered in a volume of 0.01 mL/g. The testing was conducted over the course of 1–2 days with animals in each testing cohort randomized to receive a single dose of the investigational compound. For comparison, a single dose of LEV (25 mg/kg, *i.p.* – TCI Chemical, Tokyo, Japan) formulated in 0.5% methylcellulose and CBD (100 mg/kg, *i.p.* – Cayman Chemical Company, MI, USA) formulated in cremaphor : ethanol : water (1:1:18, *v*/*v*/*v*) were tested in parallel.

ivPTZ seizure threshold test, grip strength test, and PTZ-induced kindling model in mice. Naïve male albino Swiss mice obtained from the Laboratory Animals Breeding (Kołacz, Warsaw, Poland) were used for all experiments. The experimental protocols were approved by the Local Ethics Committee for Experiments on Animals in Lublin, Poland (approval no. 13/2021 and 46/2021). The *iv*PTZ seizure threshold test and the grip strength test were carried out as described in detail elsewhere.[25] Compound **d$_4$-(*R*)-AS-1** was suspended in a 1% Tween 80 and administered *i.p.*, 30 min prior to the tests. The neuromuscular strength in the grip strength test was measured shortly before the *iv*PTZ test.

The PTZ kindling procedure was carried out according to the method described in our previous studies.[17,26] The following experimental groups were used: (a) 1% Tween + saline (non-kindled control group), (b) 1% Tween + saline (non-kindled control group subjected to the forced swim test), (c) 1% Tween + PTZ (PTZ-kindled control group); (d) VPA at 150 mg/kg + PTZ (positive control group); (e)−(g) **d$_4$-(*R*)-AS-1** at doses of 20, 40 or 80 mg/kg + PTZ. Compound **d$_4$-(*R*)-AS-1** was suspended in 1% Tween 80, whereas VPA (sodium salt, positive control) and PTZ (both from Sigma Aldrich) were dissolved in saline. **d$_4$-(*R*)-AS-1**, VPA, and saline were administered *i.p.* every 24 hours, at a volume of 10 mL/kg. Seizures were induced three times a week by administration of PTZ (at a subconvulsive dose of 40 mg/kg, *i.p.*), 30 min after administration of the tested compound, VPA, or saline. Seizure severity was scored using the modified Racine’s scale.[17,26] Twenty four hours after the last PTZ injection, animals were subjected to the behavioral tests (locomotor activity test, the elevated plus maze test, and the forced swim test), as described in detail elsewhere.[17,27]

Antinociceptive models and locomotor activity test. Experiments were carried out on adult male CD-1 mice (18–25 g) obtained from the Faculty of Pharmacy, Jagiellonian University Medical College, (Krakow, Poland). Experimental protocols were approved by the I Local Ethics Committee for Experiments on Animals in Krakow, Poland (approval no. 104/2015, 279/2019, and 614/2022). Compound **d$_4$-(*R*)-AS-1** was suspended in 1% Tween 80 and administered *i.p.* 30 min before the test. Animals in the control group (negative control) received an appropriate dose of the vehicle (1% Tween 80, *i.p.*). The experimental in vivo procedures were previously reported for the formalin test;[28] compound **d$_4$-(*R*)-AS-1** was tested in four doses–30, 60, 90, and 120 mg/kg. Before formalin application, different groups of animals were injected *i.p.* with vehicle (10 mL/kg, negative control).

The in vivo procedure for the model of capsaicin-induced nociception was previously reported;[29] the animals were pretreated with vehicle (10 mL/kg, negative control) and the dose–response of investigated compound was evaluated at 30, 60, and 90 mg/kg. The detailed in vivo procedure for the OXPT-induced peripheral neuropathy model was described previously;[30] the mice with developed tactile allodynia were pretreated *i.p.* with test compound at doses of 30, 60, and 90 mg/kg or vehicle. The STZ-induced hyperglycemia model was reported elsewhere;[31] the mice with developed mechanical allodynia were pretreated *i.p.* with test compound at doses of 30, 60, and 90 mg/kg or vehicle.

The spontaneous locomotor activity test was carried out according to the procedure described elsewhere.[32] The animals were injected (*i.p.*) with **d_4-(*R*)-AS-1** as a 1% Tween 80 suspension in water at doses of 30, 60, 90, and 120 mg/kg and placed in the activity cages (Multiple Activity Cage; Ugo Basile, Gemonio VA, Italy) individually (30 min before experiment). The number of light-beam crossings was counted in each group during the next 30 min in 10-min intervals.

Glutamate uptake studies in COS-7 cell lines expressing EAAT1, EAAT2 and EAAT3

Cells and transfection. COS-7 cells (Kidney Fibroblast; African Green Monkey, ATCC-CRL-1651, ATCC, Manassas, VA, USA) were maintained in DMEM (ATCC, Manassas, VA, USA) containing 10% Fetal Bovine Serum (ATCC, Manassas, VA, USA), 100 units/mL of penicillin and 100 µg/mL streptomycin (Thermo Fisher Scientific, Waltham, MA, USA) in a humidified incubator with 5% CO_2 at 37 °C. Cells were transiently transfected with empty vector CMV (for background control) or CMV-hEAAT1, CMV-EAAT2 or CMV-EAAT3 (Addgene, Watertown, MA, USA) at a concentration of 100 ng cDNA per well, using TurboFect transfection reagent (Thermo Fisher Scientific, Waltham, MA, USA, Department of Pharmacology, Maj Institute of Pharmacology Polish Academy of Sciences) or 0.5 µg cDNA per well, using TransIT-LT1 transfection reagent (Mirus Bio LLC, Madison, WI, USA, Department of Pharmacology and Physiology, Drexel University). Cells were seeded at a density of 50,000 cells per well in 24-well plates, and incubated for 48 hours before uptake assays.

Dose response assays. Glutamate uptake assays were performed using [^{3}H]-L-glutamate (Revvity, Boston, USA, specific activity 47 Ci/mmol) at a final concentration of 50 nM, as previously described,[33] together with modifications.[34] Briefly, cells were washed with the phosphate-buffered saline with calcium and magnesium (PBS-CM) buffer, pH=7.4, then incubated with 200 µL of compound tested at specified concentrations (ranging from 10^{-4} M to 10^{-12} M), for 10 min at 37°C. Compounds were dissolved in 100% DMSO, with a maximum DMSO content of 0.01% at tested concentrations. After compound incubation, 100 µL of radioligand was added and incubated for 5 min at 37°C. Reactions were finished by washing the plates twice with PBS-CM, then the cells were lysed in lysis buffer (1% SDS/0.1 N NaOH; 20 min/ RT). Radioactivity, measured as disintegrations per min (DPM), was counted on a Beckman LS 650 scintillation counter (Beckman, USA).

Kinetic assays. For kinetic assays, cells are washed in PBS-CM buffer and pre-incubated in the presence of either vehicle or 10 or 100 nM of compounds tested, and uptake reactions are initiated by the addition of unlabeled L-glutamate and [^{3}H]-L-glutamate (1–1000 µM, final concentration, 99% unlabeled and 1% labeled) for 10 min at room temperature. Non-specific uptake was also obtained in the presence of DL-TBOA (100 µM, final concentration). Reactions were finished with two washes with buffer and the addition of 100 µL of scintillation fluid to each well. Radioactivity was counted in a Microplate Scintillation and Luminescence Counter (Wallac, Shelton, CT, USA).

Glutamate transporter studies in rat and mouse astrocytes

Rat astrocyte cultures. Animal use and experiments were carried out according to the protocols approved by the Drexel University Institutional Animal Care and Use Committee (IACUC protocol number LA-23-749) under US OLAW Assurance #A3222-01. Cultures were prepared according to a previous study,[35] with modifications. Briefly, cerebral cortices from 2–4-day old Sprague-Dawley rat pups were dissected under sterile conditions and placed in 60 mm dishes containing dissection medium (in mM: Glucose 16, Sucrose 22, NaCl 135, KCl 5, Na_2HPO_4 1, KH_2PO_4 0.22, HEPES 10, pH 7.4, Osmolarity 310+10 mOsm). Tissue was minced with curved scissors, digested for 15 min in 0.25% trypsin, and dissociated by passing through a serological plastic pipette several times in presence of 60

µg/mL DNAse. Cells were pelleted by centrifugation for 15 min. at 280 x g and resuspended in plating medium (90% DMEM, 10% FBS and 50 µg/mL gentamicin) and incubated in culture flasks at 37 °C (5-10% CO_2). After growth for 10 days *in vitro* (DIV), cells were detached with 0.05% trypsin, centrifuged, and plated at the density of 10,000 cells/well in poly-lysine coated 96-well plates. Plates are grown for 14 DIV before uptake assays.

Mouse astrocyte cultures. Cultures were isolated from cerebellum of C57BL/6 mice (#CRL-2541; ATCC, USA) and seeded at a density of 50,000 cells per well in a 24-well plate and incubated for 48 hours before uptake assays.

Dose response uptake assays. Dose-response assays were performed as described previously.[33–35] Briefly, cells were washed in PBS-CM buffer as above for 24-well plates, or using an Elx50® Biotek plate washer (Winooski, VT, USA) for 96 wells plates. Vehicle, and several concentrations of compounds tested were added and incubated for 10 min at 37°C. Uptake assays were initiated by addition of 50 nM [^{3}H]-L-glutamate, and incubation was carried on for 10 min at room temperature. Non-specific uptake was obtained in the presence of 10 µM DL-TBOA. Reactions were terminated and radioactivity measured as above.

Data analysis and statistics. Data was analyzed using GraphPad Prism version 10.1.2 for Windows (GraphPad Software, La Jolla, CA, USA). Nonspecific transport (background) obtained from transfection with empty vector or in presence of TBOA were subtracted. Dose−response curves were fitted by nonlinear regression analysis using the Hill equation [(log(agonist) vs. response (three parameters)]. EC_{50} values (as the concentration of compound resulting in 50% of the maximum observed stimulation, E_{max}), and efficacies are obtained by bottom and top parameters (best fit values) generated in GraphPad Prism and are given as means ± SEM of 3-6 independent assays performed in technical triplicate and normalized to percentage of control (vehicle). Michaelis−Menten kinetics was assumed for calculations of K_m and V_{max}. Statistical significance was determined by one-way ANOVA followed by Dunnett's (parametric data) or Dunn's (non-parametric data) multiple comparisons test, with the vehicle group as control (*$p < 0.05$, **$p < 0.01$, ***$p < 0.001$, ****$p < 0.0001$).

Influence on transporter current in mouse astrocytes

Brain Slice Preparation. Male and female C57BL/6 mice (6-10 weeks old) were used for patch clamp electrophysiology recordings. Mice were anesthetized with sodium pentobarbital (50 mg/kg) and immediately decapitated. Coronal brain slices (350 µm) containing the hippocampus were collected in an oxygenated ice-cold NMDG-HEPES cutting solution (in mM: 92 NMDG, 2.5 KCl, 1.2 NaH_2PO_4, 30 $NaHCO_3$, 20 HEPES, 25 glucose, 2 thiourea, 5 Na-ascorbate, 3 Na-pyruvate, 10 $MgSO_4$, and 0.5 $CaCl_2$). Slices were then transferred to a pre-warmed (32–34°C) holding chamber containing NMDG-HEPES for 30 min for a protective recovery period. A Na^+-dependent spike-in procedure was performed stepwise, according to an optimal age-dependent schedule.[36] Slices were then transferred to a holding chamber containing room-temperature oxygenated HEPES-aCSF holding solution (in mM: 92 NaCl, 2.5 KCl, 1.2 NaH_2PO_4, 30 $NaHCO_3$, 20 HEPES, 25 glucose, 2 thiourea, 5 Na-ascorbate, 3 Na-pyruvate, 2 $MgSO_4$, and 2 $CaCl_2$). Slices were allowed to recover for ~1 hr prior to use. To label astrocytes, slices were transferred to a pre-warmed (30 °C) holding chamber containing SR101 (0.5 µM; Sigma-Aldrich, MO, USA) in recording aCSF (in mM: 119 NaCl, 2.5 KCl, 1.25 NaH_2PO4, 24 $NaHCO_3$, 12.5 glucose, 2 $MgCl_2$, and 2 $CaCl_2$). All solutions were bubbled with 95% O_2/ 5% CO_2 throughout the experiment, pH corrected to 7.30–7.35, and adjusted for an osmolarity between 290–300 mOsm.

Electrophysiology. Whole cell patch-clamp recordings of synaptic transporter currents (STCs) were obtained through the voltage-clamp configuration using a Multiclamp 700B amplifier, a Digidata 1440A data acquisition board, and pClamp10 software (Molecular Devices, CA, USA). Slices were visualized using a 40× water immersion objective (NA 0.8; Carl Zeiss, NY, USA) and infrared differential interference contrast (IR-DIC) microscopy on an upright Axioskop3 microscope (Carl Zeiss, NY, USA). Astrocytes were identified through SR101 fluorescence (excitation 586 nm, emission 605 nm). Only astrocytes displaying SR101 fluorescence deep in the tissue were targeted for electrophysiology experiments with pipettes containing a K^+ gluconate intracellular solution (in mM: 120 K gluconate, 20 HEPES, 10 EGTA, and 0.2 Na_2GTP), pH corrected to 7.28–7.33, and adjusted for osmolarity between

290 –295 mOsm. Astrocytes were voltage-clamped at -70 mV and further distinguished from neurons by their hyperpolarized resting membrane potential, low input resistance, and lack of voltage-dependent inward currents at depolarized potentials. Resting membrane potential was determined in the I=0 mode and monitored throughout recordings. Membrane and access resistance were monitored throughout recordings and only stable recordings were included in the study. The whole-cell patch configuration and subsequent evoked responses were obtained in a pharmacological cocktail containing APV (50 µM), CNQX (10 µM), picrotoxin (50 µM), and $BaCl_2$ (200 µM) to isolate STCs.[37,38] A bipolar nichrome/formvar stimulating electrode placed 100–200 µm from the patch pipette in CA1 of the hippocampus was used to deliver a single stimulation event. Stimulation strength used for STC analyses was set as the intensity at which stimulation evoked a half-maximal response amplitude following an input-output curve with increasing stimulation intensities. Baseline STCs were collected for ~5 min prior to perfusion of **d_6-(*R*)-AS-7** (10 µM). STCs were evoked once per minute for baseline STCs and throughout drug perfusion. Only one astrocyte was patched per slice. Signals were acquired at 10 kHz and filtered at 2 kHz. All STC analyses were performed on baseline corrected traces in ClampFit (Molecular Devices, CA, USA).

Data analysis and statistics. Statistical analyses and figure preparation were performed with GraphPad Prism 8.0.1 (GraphPad Software, La Jolla, CA, USA). Data normality was assessed using the Shapiro–Wilk test to determine whether parametric or non-parametric tests were appropriate. Paired t-test was applied for the comparison of two conditions of the same group. Data are represented as mean ± SEM. Levels of significance were defined as ** $p < 0.01$, **** $p < 0.0001$.

Influence on transporter current in oocytes expressing EAAT2

Heterologous protein expression and electrophysiological study in Xenopus laevis oocytes. Experiments were performed in accordance with the protocol approved locally by the Ethical Committee of the Organismo Preposto al Benessere degli Animali (OPBA) of the University of Insubria (OPBA permit no. 06_20) and by the Italian Ministry of Health (permit no. 440/2021-PR). Oocytes were obtained from adult *Xenopus laevis* females. Frogs were anaesthetised by immersion in 0.1% (*w*/*v*) MS222 (tricaine methanesulfonate; Merck, Italy) solution in tap water adjusted at final pH 7.5 with bicarbonate. Abdomens were sterilized with antiseptic agent (Povidone-iodine 0.8%) and ovary portions were removed by laparotomy. The collected oocytes were treated with 0.5 mg/mL collagenase (Collagenase from Clostridium histolyticum; Merck, Italy) in calcium-free ND96 (96 mM NaCl, 2 mM KCl, 1 mM $MgCl_2$, 5 mM 4-(2-hydroxyethyl)-1 piperazine ethane sulfonic acid (HEPES); pH 7.6) for at least 1 h at 18°C. The healthy-looking stage V and VI oocytes were selected and stored at 18°C in NDE solution (ND96 plus 2.5 mM pyruvate, 0.05 mg/mL gentamicin sulfate, and 1.8 mM $CaCl_2$).[39] The cDNA encoding for human EAAT2 was cloned into pcDNA3 (kindly gifted by Laura Civiero), and cRNAs were *in vitro* transcribed and capped using T7 RNA polymerase. Oocytes were microinjected with 25 ng/50 nL of cRNA using a manual microinjection system (Drummond Scientific Company, Broomall, PA, USA).

The electrophysiological recordings were performed using a two-electrode voltage clamp (Oocyte Clamp OC-725 C or B; Warner Instruments, Hamden, CT, USA), the recorded currents were digitalized using Axon CNS 1440B Digidata system controlled by pClamp 11.2.2 (Molecular Devices, San Jose CA, CA, USA). The two microelectrodes were filled with 3 M KCl, and the bath electrodes were connected to the oocyte chamber via two agar bridges (3% agar in 3 M KCl). The external control solution contained 98 mM NaCl, 1 mM $MgCl_2$, 1.8 mM $CaCl_2$, 5 mM HEPES, adjusted to pH 7.6. Signals were filtered at 0.1 kHz and sampled at 200 Hz or 0.5 kHz and at 1 kHz. Transport current (I_{EAAT2}) was elicited by perfusion with 1 mM glutamate. To assess the effect of **d_6-(*R*)-AS-7**, oocytes were pre-treated with the compound for 30 seconds before co-perfusion with glutamate.

Data analysis and statistics. Clampex and Clampfit 10.7 (Molecular Devices) were used to run the experiments, and acquire and analyze the data. Statistical analyses and figure preparation were performed with OriginPro 8 (OriginLab Corporation, Northampton, MA, USA) and GraphPad Prism 8.4.3 (GraphPad Software, La Jolla, CA, USA)). Data normality was assessed using the Shapiro–Wilk test to determine whether parametric or non-parametric tests were appropriate. Paired t-test (or Wilcoxon

signed-rank test) was applied for the comparison of two conditions of the same group. Level of significance were defined as *** $p < 0.001$.

In vitro ADME-Tox studies

Permeability. Pre-coated PAMPA Plate System Gentest™ was provided by Corning, (Tewksbury, MA, USA). Compounds **d_4-(*R*)-AS-1**, **d_{11}-(*R*)-AS-1**, **(*R*)-AS-7** and **d_6-(*R*)-AS-7** were tested in similar way as **(*R*)-AS-1**.[6] The detailed procedure and proper formulas were provided by Corning.

Absorption in Caco-2 model were done with use of cell line HTB-37™ purchased directly from American Type Culture Collection (ATCC) (Manassas, VG, USA). The cells were cultivated in Dulbecco's Modified Eagle's Medium (DMEM) supplemented with 10% fetal bovine serum (FBS) in a humidified atmosphere of 5% CO_2. The medium was changed every two days, and the cells were subcultured at 70%–80% confluence. The Corning® 3413 Transwell® 6.5 mm polycarbonate membrane inserts with 0.4 µm pores were purchased from Sigma-Aldrich (Saint Louis, MO, USA). The cells were seeded at 2×10^4 concentration per insert in apical compartment, whereas 600 µL was added to the basolateral one. TEER (transepithelial electrical resistance) measurements was started from 18 days after seeding by Millicell ERS-2 Volt-Ohm Meter (Merck Millipore, Burlington, MA, USA). At the day of experiment, the monolayer was rinsed with HBSS (Hank's balanced salt solution) and tested compounds and the highly permeable reference caffeine were added at a 10 µM concentration with the HBSS into the apical chambers (A–B direction) or basolateral chambers (B–A direction). Lucifer yellow (5 µM) was also added as the membrane integrity marker. The plate was placed in the orbital shaker (60 rpm) for 2 h at 37 °C. The compounds' concentrations in apical and basolateral wells were analyzed using the UPLC-MS method with IS. To confirm the membrane integrity, the fluorescence of lucifer yellow was measured by using a Synergy H1 microplate reader (BioTek, Winooski, VT, U.S). The apparent permeability P_{app} was calculated from two experiments according to the following formula:

$P_{app}= dc/dt \times V/(A \times C_0)$

Protein binding analyses were performed with use of the commercial TRANSIL^{XL} PPB Assay (Sovicell, Leipzig, Germany) containing different concentrations of human serum albumin (HSA) and α1-acid glycoprotein (AGP) mixed in physiological ratio of 24:1. In brief the compounds were added to HSA and AGP at 5 µM final concentration and incubated on a plate shaker at 1000 rpm for 12 min. Next, the plate was centrifugated at 750 g for 10 min. The supernatants were collected and analyzed by LC/MS. The PPB parameters of tested compounds and highly bound reference warfarin were calculated using equations provided by the manufacturer.

Metabolic stability. Metabolic stability was tested in different experimental systems including mouse and human liver microsomes (MLMs, HLMs), liver S9 fraction and primary hepatocytes.

In vitro MLMs or HLMs metabolic stability. The reaction mixtures consisted of potassium phosphate buffer (100 mM, pH 7.4), liver microsomes (0.5 mg/mL), substrate (1 µM) and NADPH (1 mM). The reaction mixtures were incubated at 37°C. The five independent reactions were terminated at 0, 5, 15, 30 and 45 min by addition of cold methanol containing IS and centrifuged. Verapamil (Sigma-Aldrich, St. Louis, MO, USA) was used as the reference unstable drug. The $t_{1/2}$ values and intrinsic clearances (CL_{int}) were calculated by using the protocols and formulas proposed by Obach.[40] The microsomal protein/g of liver weight was considered as 45 mg and the liver weight/kg of body 88 g in mouse. The UPLC and mass spectra (LC-MS) were obtained on Waters ACQUITY™ TQD system (Waters, Milford, CT, USA) with the MS-TQ detector and UV-Vis-DAD eλ detector.

In vitro S9 metabolic stability. In order to study II phase metabolic stability compounds were incubated with mouse or human S9 liver fractions (Gibco™, Thermo-Fisher Scientific, Waltham, MA, USA) in the presence of Uridine 5'-diphosphoglucuronic acid trisodium salt (UDPGA) and Glutathione (GSH) (both purchased from Merck, Darmstadt, Germany). 7-hydroxycoumarin (LGC Limited, UK) was used as a reference substrate of II phase conjugation reactions. Tested and reference compounds were incubated at a final concentration of 1 µM in 24-well plates at 37°C in PBS buffer (100 mM, pH 7.4) in the presence of mouse or human S9 fraction (0.5 mg/mL), $MgCl_2$ (2 mM), UDPGA (0.5 mM), and GSH (2.5 mM) in a final volume of 0.5 mL per well. Aliquots of 50 µL were collected at 0, 15, 30, and 60 min and reaction was terminated by adding an aliquot of 150 µL ice cold acetonitrile with 0.1% formic acid

and valsartan (IS) (Merck, Darmstadt, Germany). Then, samples were mixed on a vortex and centrifuged (10 min, 10000 × g). Analytes were separated on the Hypersil Gold™ C18 analytical column (2.1 × 50 mm, 3 µm, Thermo Scientific, USA). Molar concentrations of parent compounds were determined in the tested samples using the triple quadrupole mass spectrometer Sciex QTRAP 4500 (Danaher Corporation, USA). Natural logarithm of the concentration was plotted against the time of incubation. The slopes were used to calculate elimination rate constant (k = - slope) and half-life values ($t_{1/2}$= 0.693/k). In vitro and in vivo Cl_{int} were calculated using the formulas:

$$\mathrm{Cl}_{int\ in\ vitro} = \frac{Incubation\ volume\ [ml]}{Protein\ in\ the\ incubation\ [mg]} x \frac{0.693}{t_{1/2}}$$

$$\mathrm{Cl}_{int\ in\ vivo} = \frac{Incubation\ volume\ [ml]}{Protein\ in\ the\ incubation\ [mg]} x \frac{0.693}{t_{1/2}} x \frac{Protein\ S9\ [mg]}{Gram\ of\ liver\ [g]} x \frac{Liver\ weight\ [g]}{Standard\ body\ weight\ [kg]}$$

The protein S9/gram of liver ratio was considered 121 mg/g. The liver weight/standard body weight ratios were considered 20 g and 88 g for human and mouse, respectively.

In vitro hepatocyte metabolic stability. These assays were performed commercially in Sai Life Sciences Limited (Hyderabad, Telangana, India), applying validated procedures. HLMs were obtained from Corning® life Sciences (Corning, NY, USA). MLMs (Gibco™) were purchased from (Thermo-Fisher Scientific, Waltham, MA, USA). Mouse hepatocytes, thawing and incubation media were purchased from BIO-IVT (Westbury, NY, USA).

Hepatocyte incubations were carried out in a 48 well plate with a cell density of $1{\times}10^6$ cells /mL, with a final substrate concentration of 1 µM. The incubations were performed in a CO_2 incubator (5% CO_2) maintained at 37ºC. Total incubation volume was 400 µL. An aliquot of 25 µL was withdrawn at 0.5, 5, 10, 15, 30, 60, 120, 240 and 360 min time points and the reaction terminated by adding 200 µL of ice-cold MeCN containing IS and centrifuged. The microsomal incubations consisted of potassium phosphate buffer (100 mM, pH 7.4), liver microsomes (0.5 mg/mL), substrate (0.5 µM) and NADPH (1 mM). The reaction mixtures were incubated at 37°C. At 0.5, 5, 10, 15, 30 and 60 min time points an aliquot of the incubation mixture was quenched with 4 volumes of ice-cold MeCN containing IS, vortexed and centrifuged at 4000 rpm for 10 min. The supernatant was separated and subjected to LC-MS/MS analysis.

Bioanalysis of samples from hepatocytes and microsomes was performed on a LC-MS/MS system consisting of a Waters Acquity UPLC and API 7500 mass spectrometer (Applied Biosystems, MDS Sciex Toronto, Canada) equipped with an API electrospray ionization (ESI) source. A well stirred liver metabolism model was used to calculate the *in vitro* intrinsic clearance (CL_{int}) from the microsomal and hepatocyte incubations.[41] Peak area ratios (analyte to IS) were considered for calculation of percent parent compound remaining (PCR). 0.5 min time point was considered as 100% PCR, for other time points %PCR was calculated against 0.5 min. The slope (*k*) of linear range of natural logarithmic (ln) curve of percent remaining versus time was used for calculation of half-life ($t_{1/2}$) (min) = 0.693/*k*. *In vitro* intrinsic clearance (CL_{int}) (mL/min/kg) was calculated using the formula:

$$CL_{int} = \frac{k}{Cells\ in\ incubation} \times \frac{\alpha}{1g\ of\ liver\ weight} \times \frac{liver\ weight[g]}{1\ kg\ of\ body\ weigh}$$

"α" stands for *hepatocellularity* in case of hepatocyte incubations, microsomal protein/1g of liver in case of microsomal incubations. The *hepatocellularity* for mouse was considered as $130{\times}10^6$ cells/gram of liver. The microsomal protein/g of liver weight was considered as 45 mg for both mouse and human liver microsomes. The liver weight/kg of body is taken as 88 g and 20 g for mouse and human, respectively.[42]

Toxicity assays. The luminescent CYP3A4 P450-Glo™, CYP2D6 P450-Glo™ and CYP2C9 P450-Glo™ (Promega, Madison, WI, USA) were used for estimation of drug-drug interactions. All tests were performed following procedures provided by manufacturer. The compounds were tested at final concentrations of 0.1, 1, 10 and 25 µM. Neurotoxicity and hepatotoxicity assays were done in

hepatoma HepG2 and SH-SY5Y cell lines, respectively. The cell lines were provided directly from ATCC® (American Type Culture Collection, Manassas, VA, USA). All tests were performed in the same way as described previously for compound **(*R*)-AS-1**.[6]

Phospholipidosis induction assay. The HepG2 cells were treated by 10 µM and 50 µM of phospholipidosis inductor verapamil and by 50 µM and 100 µM of **d_6-(*R*)-AS-7**. The cells were stained next by LYSO-ID® Red cytotoxicity kit containing Dual Color Detection Reagent (Enzo Biochem, Inc. NY, USA). The red fluorescent lysosomal signal and the blue nuclear signal were registered by microscope Leica DMi8 (Leica, Wetzlar, Germany).

References

(1) Rigaku-Oxford Diffraction; CrysAlisPro Oxford Diffraction Ltd, Abingdon, England V 1. 171. 36. 2. (Release 27-06-2012 CN) 2006.

(2) Burla, M. C.; Caliandro, R.; Carrozzini, B.; Cascarano, G. L.; Cuocci, C.; Giacovazzo, C.; Mallamo, M.; Mazzone, A.; Polidori, G. Crystal Structure Determination and Refinement via SIR2014. *J. Appl. Crystallogr.* **2015**, *48* (1), 306–309. https://doi.org/10.1107/S1600576715001132.

(3) Sheldrick, G. M. A Short History of SHELX. *Acta Crystallogr. A* **2008**, *64* (1), 112–122. https://doi.org/10.1107/S0108767307043930.

(4) Macrae, C. F.; Sovago, I.; Cottrell, S. J.; Galek, P. T. A.; McCabe, P.; Pidcock, E.; Platings, M.; Shields, G. P.; Stevens, J. S.; Towler, M.; Wood, P. A. Mercury 4.0: From Visualization to Analysis, Design and Prediction. *J. Appl. Crystallogr.* **2020**, *53* (Pt 1), 226–235. https://doi.org/10.1107/S1600576719014092.

(5) Parsons, S.; Flack, H. D.; Wagner, T. Use of Intensity Quotients and Differences in Absolute Structure Refinement. *Acta Crystallogr. Sect. B Struct. Sci. Cryst. Eng. Mater.* **2013**, *69* (3), 249–259. https://doi.org/10.1107/S2052519213010014.

(6) Abram, M.; Jakubiec, M.; Reeb, K.; Cheng, M. H.; Gedschold, R.; Rapacz, A.; Mogilski, S.; Socała, K.; Nieoczym, D.; Szafarz, M.; Latacz, G.; Szulczyk, B.; Kalinowska-Tłuścik, J.; Gawel, K.; Esguerra, C. V.; Wyska, E.; Müller, C. E.; Bahar, I.; Fontana, A. C. K.; Wlaź, P.; Kamiński, R. M.; Kamiński, K. Discovery of (R)-N-Benzyl-2-(2,5-Dioxopyrrolidin-1-Yl)Propanamide [(R)-AS-1], a Novel Orally Bioavailable EAAT2 Modulator with Drug-like Properties and Potent Antiseizure Activity In Vivo. *J. Med. Chem.* **2022**, *65* (17), 11703–11725. https://doi.org/10.1021/acs.jmedchem.2c00534.

(7) Singh, T.; Mishra, A.; Goel, R. K. PTZ Kindling Model for Epileptogenesis, Refractory Epilepsy, and Associated Comorbidities: Relevance and Reliability. *Metab. Brain Dis.* **2021**, *36* (7), 1573–1590. https://doi.org/10.1007/s11011-021-00823-3.

(8) Mazzitelli, M.; Presto, P.; Antenucci, N.; Meltan, S.; Neugebauer, V. Recent Advances in the Modulation of Pain by the Metabotropic Glutamate Receptors. *Cells* **2022**, *11* (16), 2608. https://doi.org/10.3390/cells11162608.

(9) Temmermand, R.; Barrett, J. E.; Fontana, A. C. K. Glutamatergic Systems in Neuropathic Pain and Emerging Non-Opioid Therapies. *Pharmacol. Res.* **2022**, *185*, 106492. https://doi.org/10.1016/j.phrs.2022.106492.

(10) Salinas-Abarca, A. B.; Avila-Rojas, S. H.; Barragán-Iglesias, P.; Pineda-Farias, J. B.; Granados-Soto, V. Formalin Injection Produces Long-Lasting Hypersensitivity with Characteristics of Neuropathic Pain. *Eur. J. Pharmacol.* **2017**, *797*, 83–93. https://doi.org/10.1016/j.ejphar.2017.01.018.

(11) Muley, M. M.; Krustev, E.; McDougall, J. J. Preclinical Assessment of Inflammatory Pain. *CNS Neurosci. Ther.* **2015**, *22* (2), 88–101. https://doi.org/10.1111/cns.12486.

(12) Ling, B.; Coudoré-Civiale, M.-A.; Balayssac, D.; Eschalier, A.; Coudoré, F.; Authier, N. Behavioral and Immunohistological Assessment of Painful Neuropathy Induced by a Single Oxaliplatin Injection in the Rat. *Toxicology* **2007**, *234* (3), 176–184. https://doi.org/10.1016/j.tox.2007.02.013.

(13) Cheng, K.-I.; Wang, H.-C.; Tseng, K.-Y.; Wang, Y.-H.; Chang, C.-Y.; Chen, Y.-J.; Lai, C.-S.; Chen, D.-R.; Chang, L.-L. Cilostazol Ameliorates Peripheral Neuropathic Pain in Streptozotocin-Induced Type I Diabetic Rats. *Front. Pharmacol.* **2021**, *12*, 771271. https://doi.org/10.3389/fphar.2021.771271.

(14) Chen, X.; Murawski, A.; Patel, K.; Crespi, C. L.; Balimane, P. V. A Novel Design of Artificial Membrane for Improving the PAMPA Model. *Pharm. Res.* **2008**, *25* (7), 1511–1520. https://doi.org/10.1007/s11095-007-9517-8.

(15) Jakubiec, M.; Abram, M.; Zagaja, M.; Socała, K.; Panic, V.; Latacz, G.; Mogilski, S.; Szafarz, M.; Szala-Rycaj, J.; Saunders, J.; West, P. J.; Nieoczym, D.; Przejczowska-Pomierny, K.; Szulczyk, B.; Krupa, A.; Wyska, E.; Wlaź, P.; Metcalf, C. S.; Wilcox, K.; Andres-Mach, M.; Kamiński, R. M.; Kamiński, K. Discovery and Profiling of New Multimodal Phenylglycinamide Derivatives as

Potent Antiseizure and Antinociceptive Drug Candidates. *ACS Chem. Neurosci.* **2024**, *15* (17), 3228–3256. https://doi.org/10.1021/acschemneuro.4c00438.
(16) Jakubiec, M.; Abram, M.; Zagaja, M.; Andres-Mach, M.; Szala-Rycaj, J.; Latacz, G.; Honkisz-Orzechowska, E.; Mogilski, S.; Kubacka, M.; Szafarz, M.; Pociecha, K.; Przejczowska-Pomierny, K.; Wyska, E.; Socała, K.; Nieoczym, D.; Szulczyk, B.; Wlaź, P.; Metcalf, C. S.; Wilcox, K.; Kamiński, R. M.; Kamiński, K. Novel Alaninamide Derivatives with Drug-like Potential for Development as Antiseizure and Antinociceptive Therapies–In Vitro and In Vivo Characterization. *ACS Chem. Neurosci.* **2024**, *15* (11), 2198–2222. https://doi.org/10.1021/acschemneuro.4c00013.
(17) Socała, K.; Mogilski, S.; Pieróg, M.; Nieoczym, D.; Abram, M.; Szulczyk, B.; Lubelska, A.; Latacz, G.; Doboszewska, U.; Wlaź, P.; Kamiński, K. KA-11, a Novel Pyrrolidine-2,5-Dione Derived Broad-Spectrum Anticonvulsant: Its Antiepileptogenic, Antinociceptive Properties and in Vitro Characterization. *ACS Chem. Neurosci.* **2019**, *10* (1), 636–648. https://doi.org/10.1021/acschemneuro.8b00476.
(18) Uttry, A.; Mal, S.; van Gemmeren, M. Late-Stage β-C(Sp3)–H Deuteration of Carboxylic Acids. *J. Am. Chem. Soc.* **2021**, *143* (29), 10895–10901. https://doi.org/10.1021/jacs.1c06474.
(19) Litchfield, J. T.; Wilcoxon, F. A Simplified Method of Evaluating Dose-Effect Experiments. *J. Pharmacol. Exp. Ther.* **1949**, *96* (2), 99–113.
(20) Meeker, S.; Beckman, M.; Knox, K. M.; Treuting, P. M.; Barker-Haliski, M. Repeated Intraperitoneal Administration of Low-Concentration Methylcellulose Leads to Systemic Histologic Lesions Without Loss of Preclinical Phenotype. *J. Pharmacol. Exp. Ther.* **2019**, *371* (1), 25–35. https://doi.org/10.1124/jpet.119.257261.
(21) Barton, M. E.; Klein, B. D.; Wolf, H. H.; White, H. S. Pharmacological Characterization of the 6 Hz Psychomotor Seizure Model of Partial Epilepsy. *Epilepsy Res.* **2001**, *47* (3), 217–227. https://doi.org/10.1016/s0920-1211(01)00302-3.
(22) Barker-Haliski, M. L.; Johnson, K.; Billingsley, P.; Huff, J.; Handy, L. J.; Khaleel, R.; Lu, Z.; Mau, M. J.; Pruess, T. H.; Rueda, C.; Saunders, G.; Underwood, T. K.; Vanegas, F.; Smith, M. D.; West, P. J.; Wilcox, K. S. Validation of a Preclinical Drug Screening Platform for Pharmacoresistant Epilepsy. *Neurochem. Res.* **2017**, *42* (7), 1904–1918. https://doi.org/10.1007/s11064-017-2227-7.
(23) Koneval, Z.; Knox, K. M.; Memon, A.; Zierath, D. K.; White, H. S.; Barker-Haliski, M. Antiseizure Drug Efficacy and Tolerability in Established and Novel Drug Discovery Seizure Models in Outbred vs Inbred Mice. *Epilepsia* **2020**, *61* (9), 2022–2034. https://doi.org/10.1111/epi.16624.
(24) Pincus, J. H. Experimental Models of Epilepsy. A Manual for the Laboratory Worker. *Yale J. Biol. Med.* **1974**, *47* (4), 303.
(25) Socała, K.; Nieoczym, D.; Kowalczuk-Vasilev, E.; Wyska, E.; Wlaź, P. Increased Seizure Susceptibility and Other Toxicity Symptoms Following Acute Sulforaphane Treatment in Mice. *Toxicol. Appl. Pharmacol.* **2017**, *326*, 43–53. https://doi.org/10.1016/j.taap.2017.04.010.
(26) Kamiński, K.; Socała, K.; Zagaja, M.; Andres-Mach, M.; Abram, M.; Jakubiec, M.; Pieróg, M.; Nieoczym, D.; Rapacz, A.; Gawel, K.; Esguerra, C. V.; Latacz, G.; Lubelska, A.; Szulczyk, B.; Szewczyk, A.; Łuszczki, J. J.; Wlaź, P. N-Benzyl-(2,5-Dioxopyrrolidin-1-Yl)Propanamide (AS-1) with Hybrid Structure as a Candidate for a Broad-Spectrum Antiepileptic Drug. *Neurotherapeutics* **2020**, *17* (1), 309–328. https://doi.org/10.1007/s13311-019-00773-w.
(27) Socała, K.; Wlaź, P. Evaluation of the Antidepressant- and Anxiolytic-like Activity of α-Spinasterol, a Plant Derivative with TRPV1 Antagonistic Effects, in Mice. *Behav. Brain Res.* **2016**, *303*, 19–25. https://doi.org/10.1016/j.bbr.2016.01.048.
(28) Beirith, A.; Santos, A. R.; Rodrigues, A. L.; Creczynski-Pasa, T. B.; Calixto, J. B. Spinal and Supraspinal Antinociceptive Action of Dipyrone in Formalin, Capsaicin and Glutamate Tests. Study of the Mechanism of Action. *Eur. J. Pharmacol.* **1998**, *345* (3), 233–245. https://doi.org/10.1016/s0014-2999(98)00026-0.
(29) Mogilski, S.; Kubacka, M.; Redzicka, A.; Kazek, G.; Dudek, M.; Malinka, W.; Filipek, B. Antinociceptive, Anti-Inflammatory and Smooth Muscle Relaxant Activities of the Pyrrolo[3,4-

d]Pyridazinone Derivatives: Possible Mechanisms of Action. *Pharmacol. Biochem. Behav.* **2015**, *133*, 99–110. https://doi.org/10.1016/j.pbb.2015.03.019.
(30) Sałat, K.; Cios, A.; Wyska, E.; Sałat, R.; Mogilski, S.; Filipek, B.; Więckowski, K.; Malawska, B. Antiallodynic and Antihyperalgesic Activity of 3-[4-(3-Trifluoromethyl-Phenyl)-Piperazin-1-Yl]-Dihydrofuran-2-One Compared to Pregabalin in Chemotherapy-Induced Neuropathic Pain in Mice. *Pharmacol. Biochem. Behav.* **2014**, *122*, 173–181. https://doi.org/10.1016/j.pbb.2014.03.025.
(31) Furman, B. L. Streptozotocin-Induced Diabetic Models in Mice and Rats. *Curr. Protoc.* **2021**, *1* (4), e78. https://doi.org/10.1002/cpz1.78.
(32) Mogilski, S.; Kubacka, M.; Łażewska, D.; Więcek, M.; Głuch-Lutwin, M.; Tyszka-Czochara, M.; Bukowska-Strakova, K.; Filipek, B.; Kieć-Kononowicz, K. Aryl-1,3,5-Triazine Ligands of Histamine H4 Receptor Attenuate Inflammatory and Nociceptive Response to Carrageen, Zymosan and Lipopolysaccharide. *Inflamm. Res.* **2017**, *66* (1), 79–95. https://doi.org/10.1007/s00011-016-0997-z.
(33) Fontana, A. C. K. Protocols for Measuring Glutamate Uptake: Dose-Response and Kinetic Assays in In Vitro and Ex Vivo Systems. *Curr. Protoc. Pharmacol.* **2018**, *82* (1), e45. https://doi.org/10.1002/cpph.45.
(34) Kamiński, K.; Socała, K.; Abram, M.; Jakubiec, M.; Reeb, K. L.; Temmermand, R.; Zagaja, M.; Maj, M.; Kolasa, M.; Faron-Górecka, A.; Andres-Mach, M.; Szewczyk, A.; Hameed, M. Q.; Fontana, A. C. K.; Rotenberg, A.; Kamiński, R. M. Enhancement of Glutamate Uptake as Novel Antiseizure Approach: Preclinical Proof of Concept. *Ann. Neurol.* **2025**, *97* (2), 344–357. https://doi.org/10.1002/ana.27124.
(35) Forster, Y. M.; Green, J. L.; Khatiwada, A.; Liberato, J. L.; Narayana Reddy, P. A.; Salvino, J. M.; Bienz, S.; Bigler, L.; Dos Santos, W. F.; Karklin Fontana, A. C. Elucidation of the Structure and Synthesis of Neuroprotective Low Molecular Mass Components of the Parawixia Bistriata Spider Venom. *ACS Chem. Neurosci.* **2020**, *11* (11), 1573–1596. https://doi.org/10.1021/acschemneuro.0c00007.
(36) Ting, J. T.; Daigle, T. L.; Chen, Q.; Feng, G. Acute Brain Slice Methods for Adult and Aging Animals: Application of Targeted Patch Clamp Analysis and Optogenetics. *Methods Mol. Biol. Clifton NJ* **2014**, *1183*, 221–242. https://doi.org/10.1007/978-1-4939-1096-0_14.
(37) Bergles, D. E.; Jahr, C. E. Synaptic Activation of Glutamate Transporters in Hippocampal Astrocytes. *Neuron* **1997**, *19* (6), 1297–1308. https://doi.org/10.1016/S0896-6273(00)80420-1.
(38) Giangrasso, D. M.; Veros, K. M.; Timm, M. M.; West, P. J.; Wilcox, K. S.; Keefe, K. A. Glutamate Dynamics in the Dorsolateral Striatum of Rats with Goal-Directed and Habitual Cocaine-Seeking Behavior. *Front. Mol. Neurosci.* **2023**, *16*, 1160157. https://doi.org/10.3389/fnmol.2023.1160157.
(39) Bhatt, M.; Di Iacovo, A.; Romanazzi, T.; Roseti, C.; Cinquetti, R.; Bossi, E. The “Www” of Xenopus Laevis Oocytes: The Why, When, What of Xenopus Laevis Oocytes in Membrane Transporters Research. *Membranes* **2022**, *12* (10), 927. https://doi.org/10.3390/membranes12100927.
(40) Obach, R. S. Prediction of Human Clearance of Twenty-Nine Drugs from Hepatic Microsomal Intrinsic Clearance Data: An Examination of In Vitro Half-Life Approach and Nonspecific Binding to Microsomes. *Drug Metab. Dispos.* **1999**, *27* (11), 1350–1359.
(41) Houston, J. B.; Carlile, D. J. Prediction of Hepatic Clearance from Microsomes, Hepatocytes, and Liver Slices. *Drug Metab. Rev.* **1997**, *29* (4), 891–922.
(42) Davies, B.; Morris, T. Physiological Parameters in Laboratory Animals and Humans. *Pharm. Res.* **1993**, *10* (7), 1093–1095. https://doi.org/10.1023/a:1018943613122.

UPLC/HRMS traces for final compounds

(*R*)-*N*-benzyl-2-(2,5-dioxopyrrolidin-1-yl-3,3,4,4-d_4)propanamide - **d_4-(*R*)-AS-1**

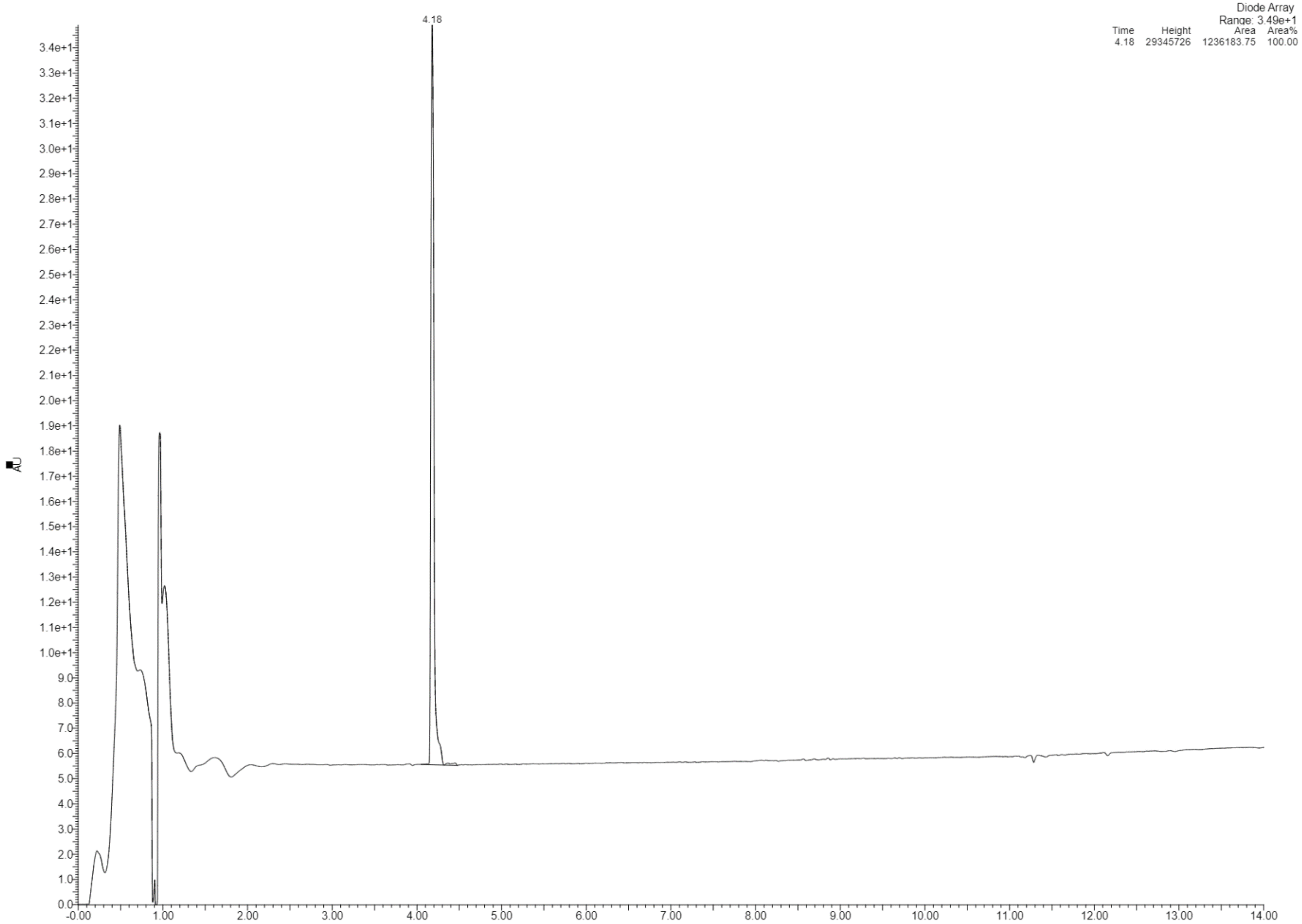

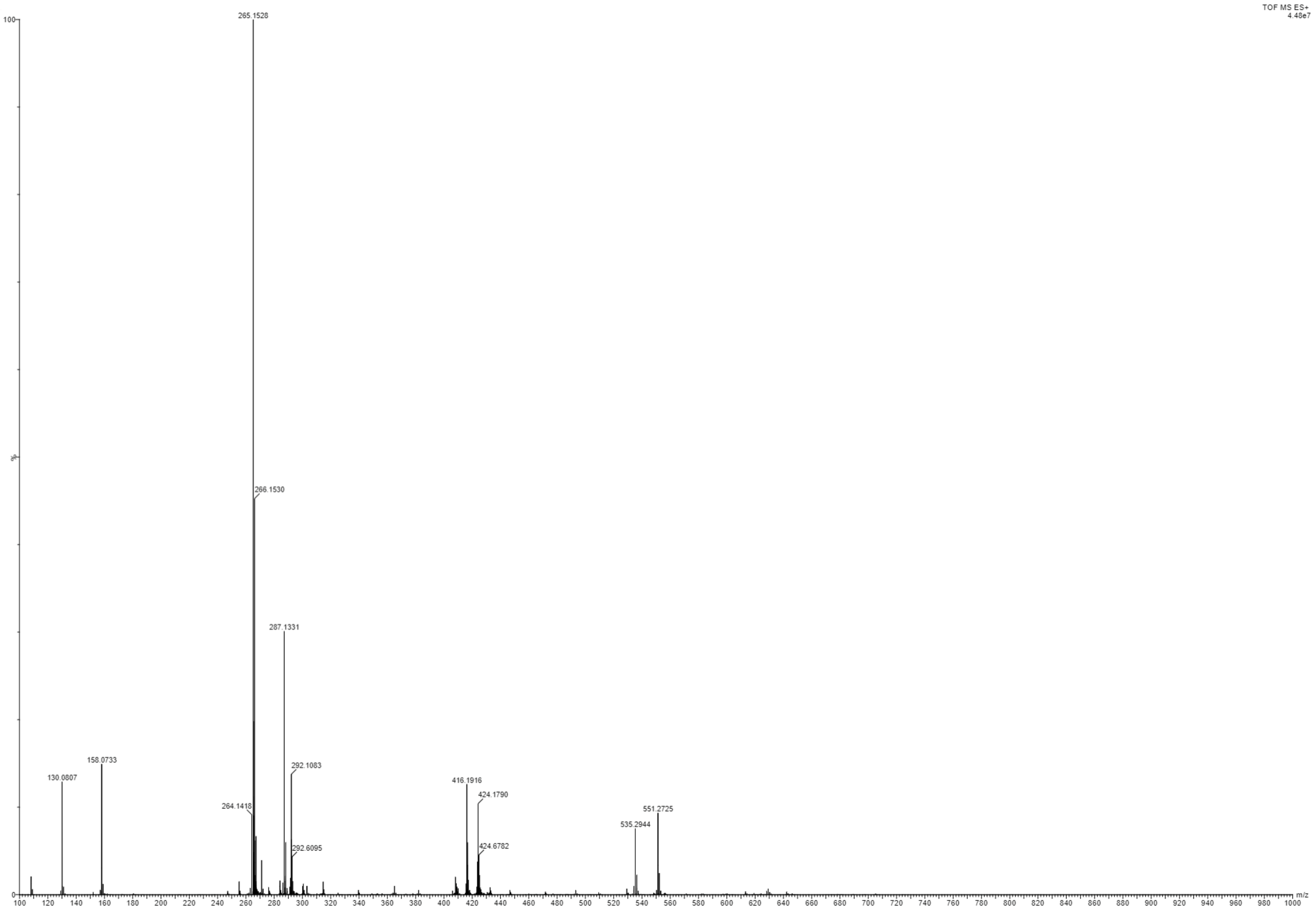
TOF MS ES+
4.48e7
265.1528
266.1530
287.1331
292.1083
292.6095
264.1418
158.0733
130.0807
416.1916
424.1790
424.6782
551.2725
535.2944
%
m/z

(R)-2-(2,5-dioxopyrrolidin-1-yl)-N-((phenyl-d_5)methyl)propanamide - **d_5-(R)-AS-1**

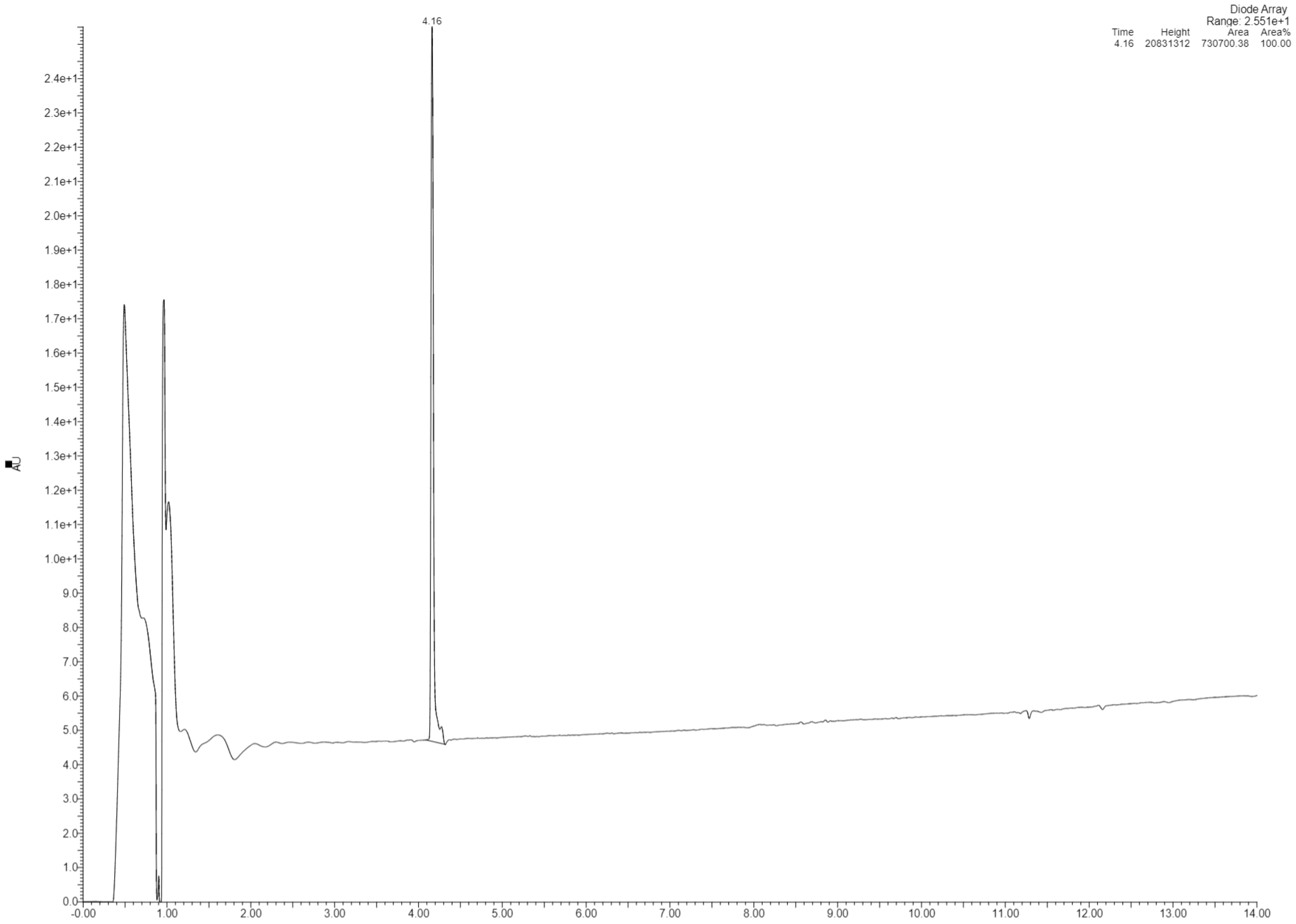

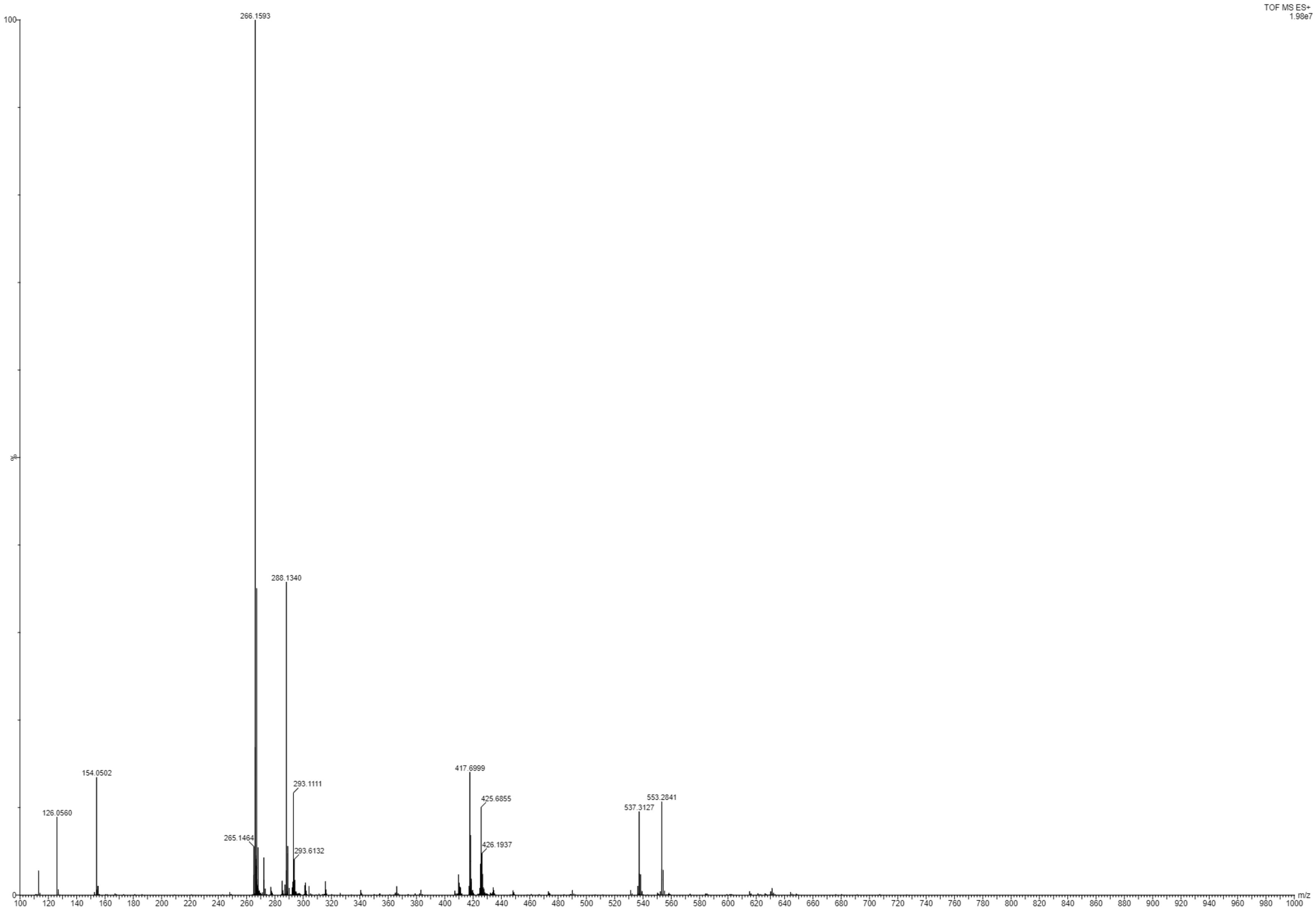

TOF MS ES+
1.98e7
100
%
0
266.1593
288.1340
154.0502
126.0560
293.1111
265.1464
293.6132
417.6999
425.6855
426.1937
537.3127
553.2841
m/z
100 120 140 160 180 200 220 240 260 280 300 320 340 360 380 400 420 440 460 480 500 520 540 560 580 600 620 640 660 680 700 720 740 760 780 800 820 840 860 880 900 920 940 960 980 1000

(*R*)-2-(2,5-dioxopyrrolidin-1-yl-3,3,4,4-d_4)-*N*-(phenylmethyl-d_2)propanamide - **d_6-(*R*)-AS-1**

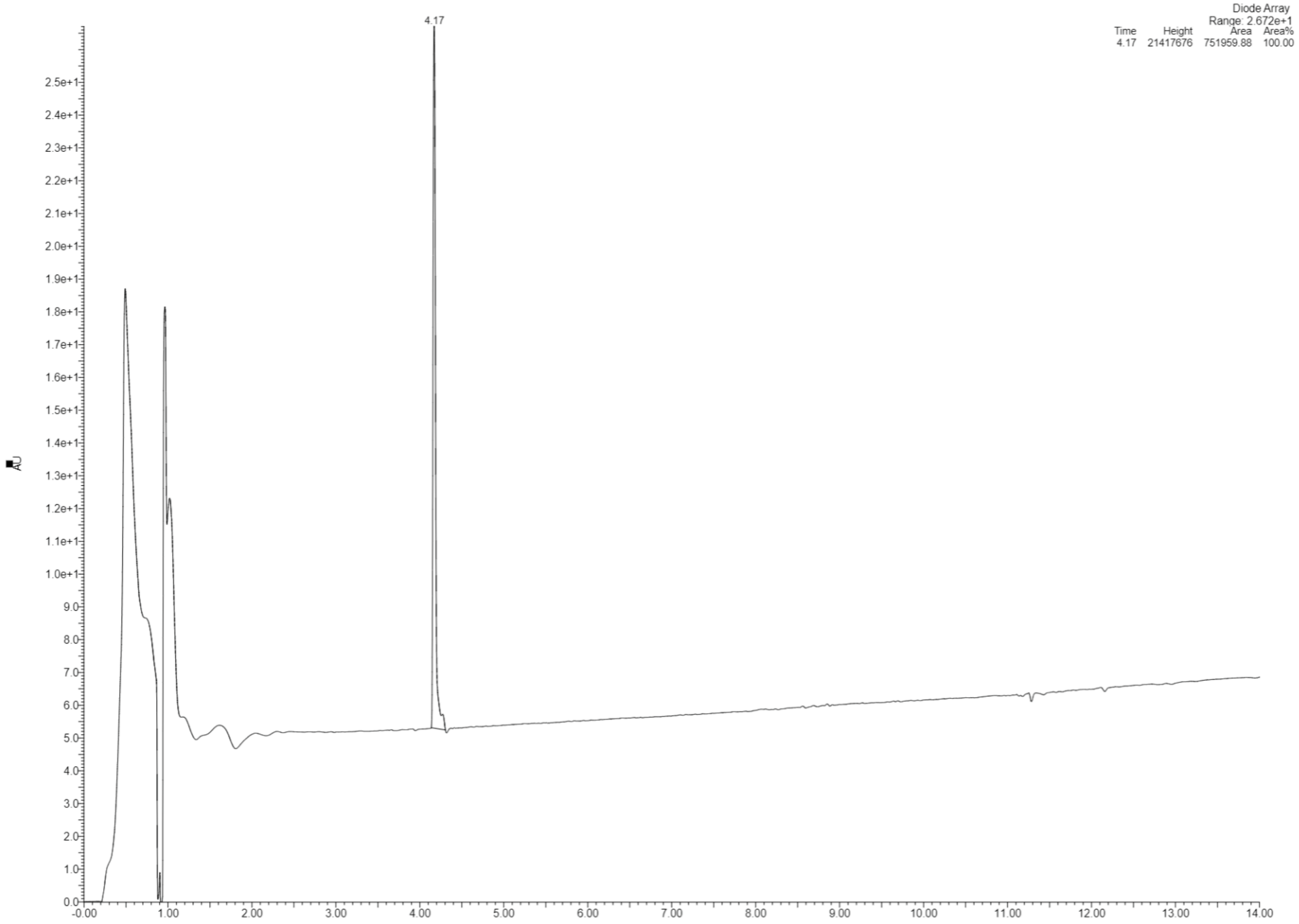

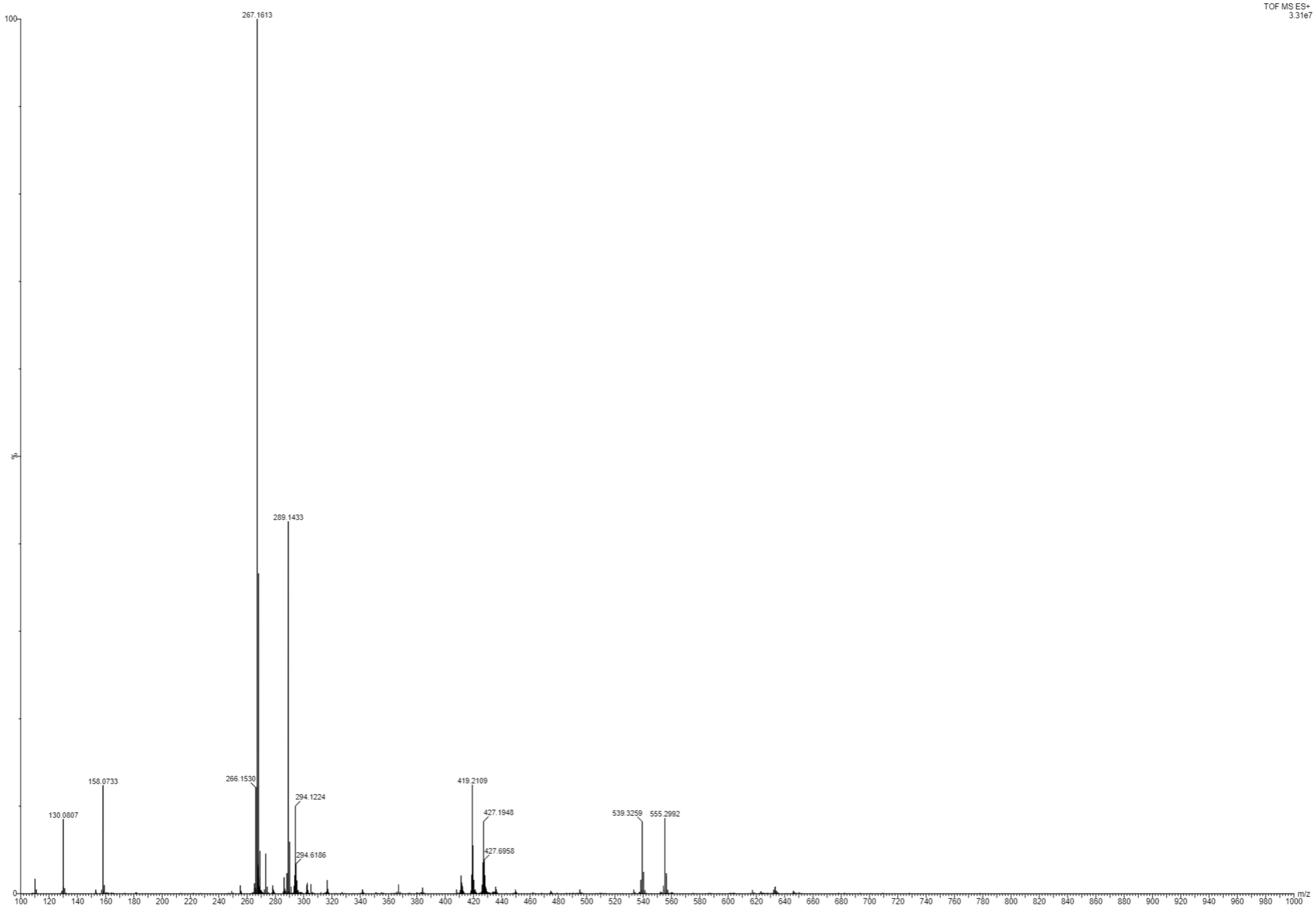
TOF MS ES+
3.31e7
100
%
0
267.1613
289.1433
266.1530
158.0733
130.0807
294.1224
294.6186
419.2109
427.1948
427.6958
539.3259
555.2992
m/z
100 120 140 160 180 200 220 240 260 280 300 320 340 360 380 400 420 440 460 480 500 520 540 560 580 600 620 640 660 680 700 720 740 760 780 800 820 840 860 880 900 920 940 960 980 1000

(R)-2-(2,5-dioxopyrrolidin-1-yl-3,3,4,4-d_4)-N-((phenyl-d_5)methyl)propanamide - **d_9-(R)-AS-1**

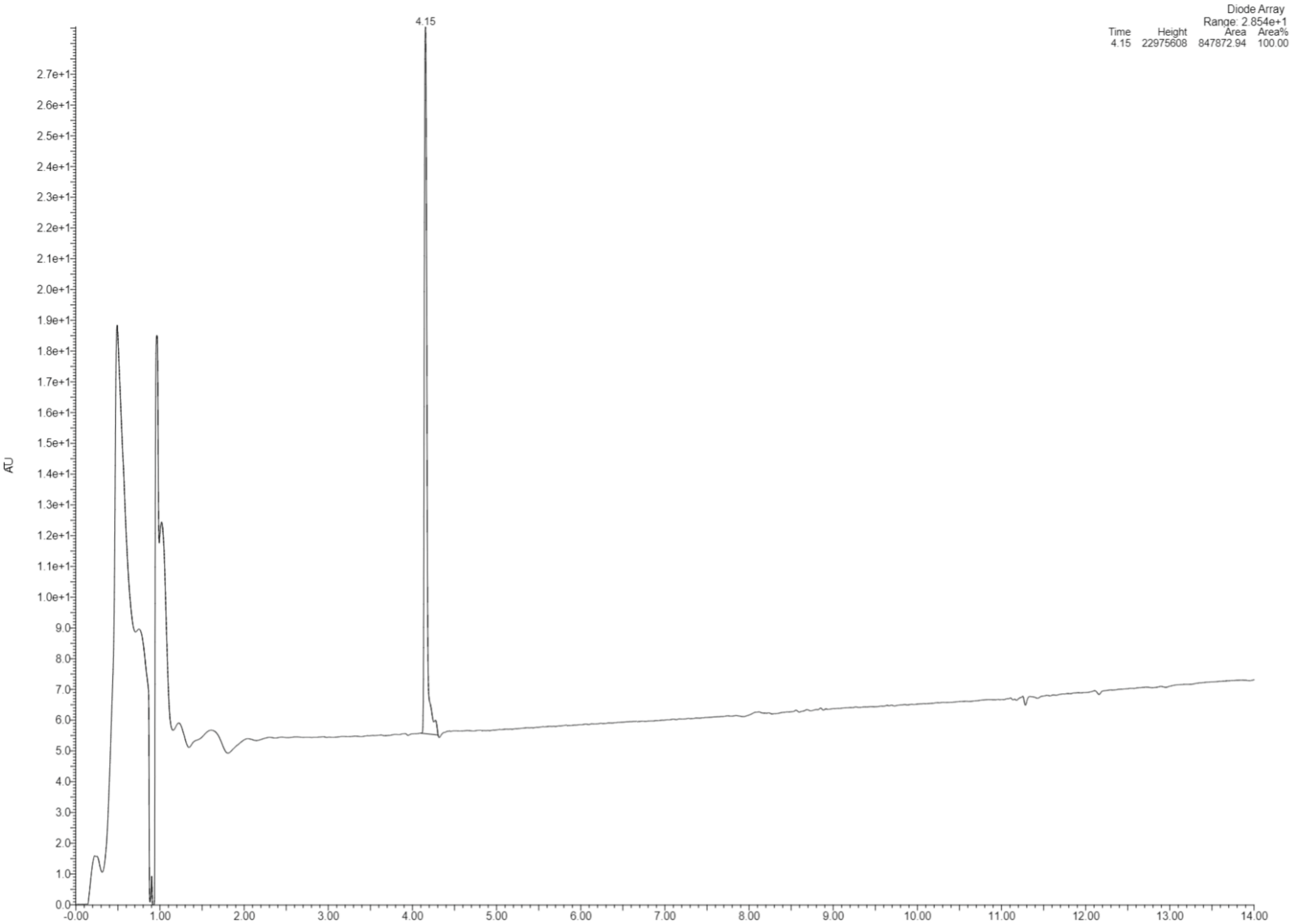

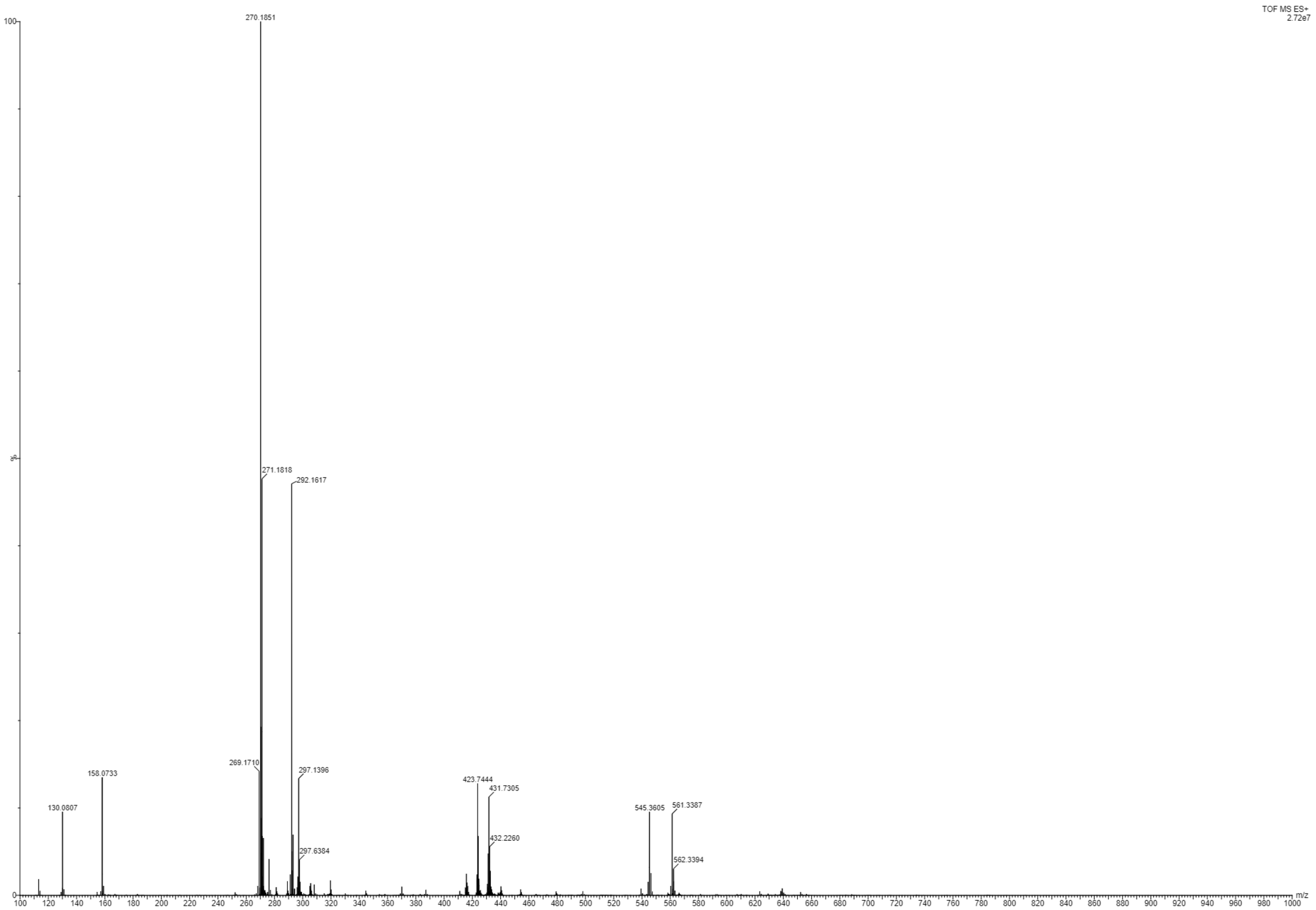
TOF MS ES+
2.72e7
270.1851
271.1818
292.1617
269.1710
297.1396
158.0733
130.0807
297.6384
423.7444
431.7305
432.2260
545.3605
561.3387
562.3394
100
%
0
m/z

(*R*)-2-(2,5-dioxopyrrolidin-1-yl-3,3,4,4-d_4)-*N*-((phenyl-d_5)methyl-d_2)propanamide - **d_{11}-(*R*)-AS-1**

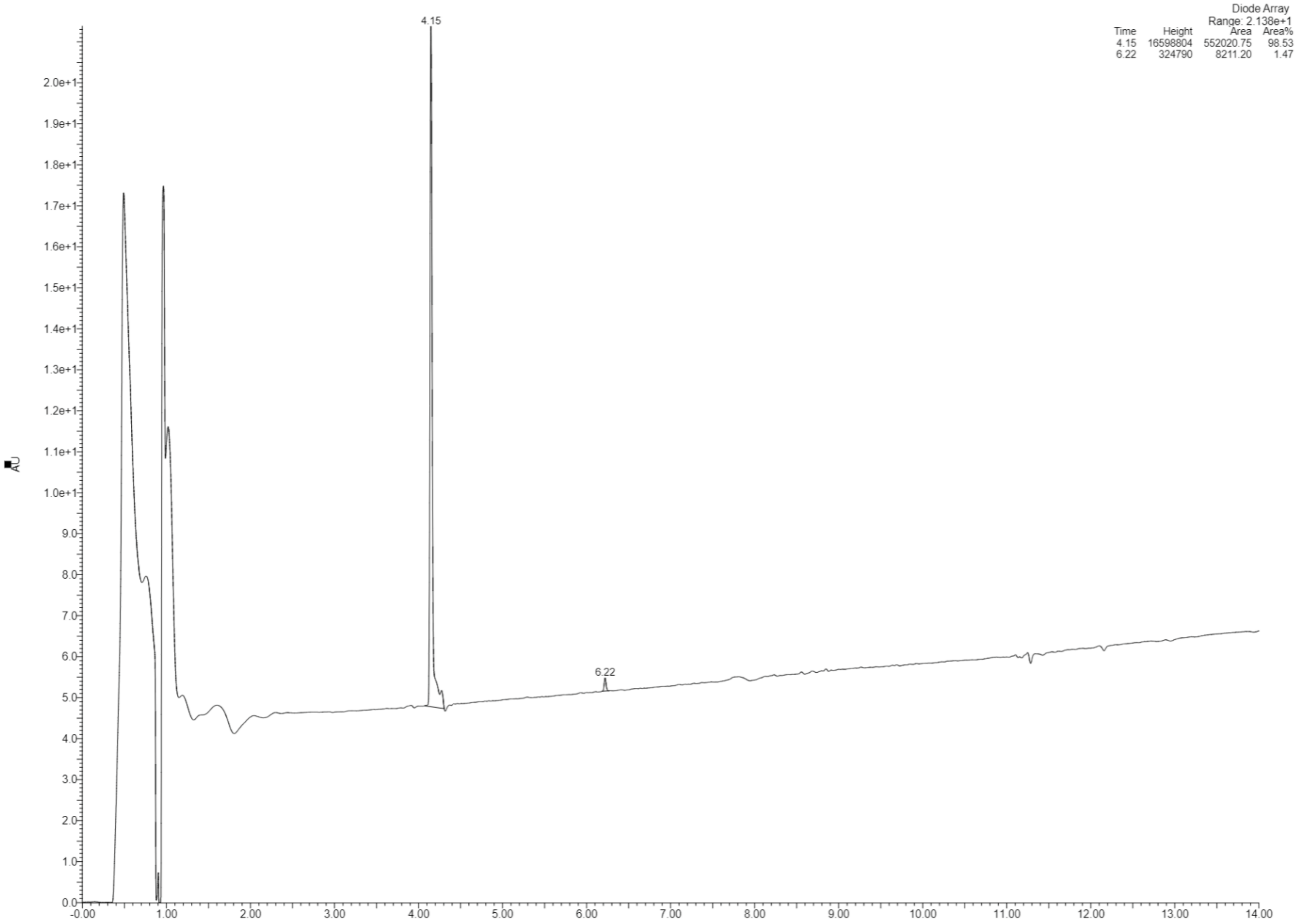

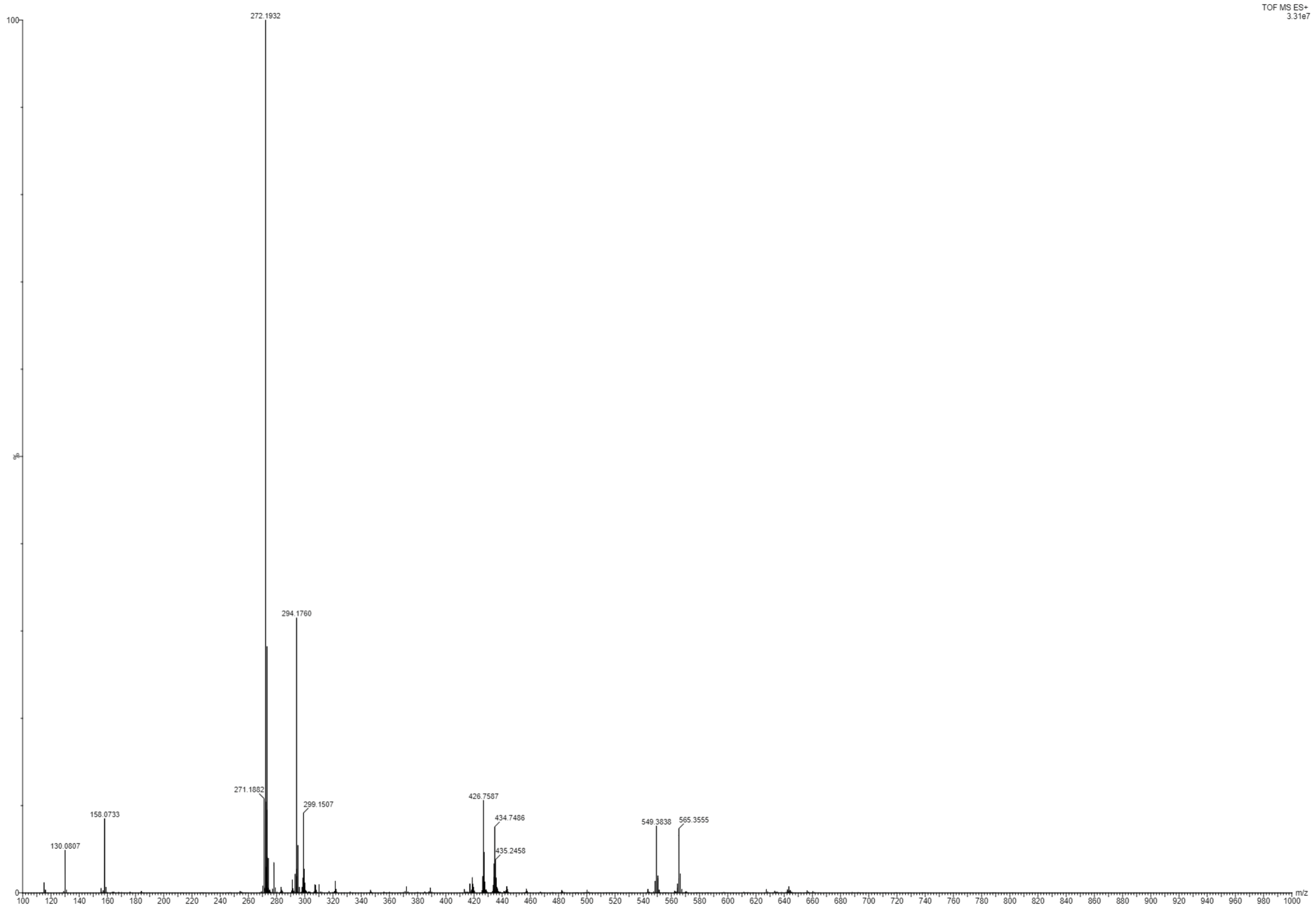

TOF MS ES+
3.31e7
100
%
0
272.1932
294.1760
271.1882
299.1507
158.0733
130.0807
426.7587
434.7486
435.2458
549.3838
565.3555
m/z
100
120
140
160
180
200
220
240
260
280
300
320
340
360
380
400
420
440
460
480
500
520
540
560
580
600
620
640
660
680
700
720
740
760
780
800
820
840
860
880
900
920
940
960
980
1000

R)-2-(2,5-dioxopyrrolidin-1-yl-3,3,4,4-d_4)-*N*-(3-fluorobenzyl)propanamide - **d_4-(*R*)-AS-7**

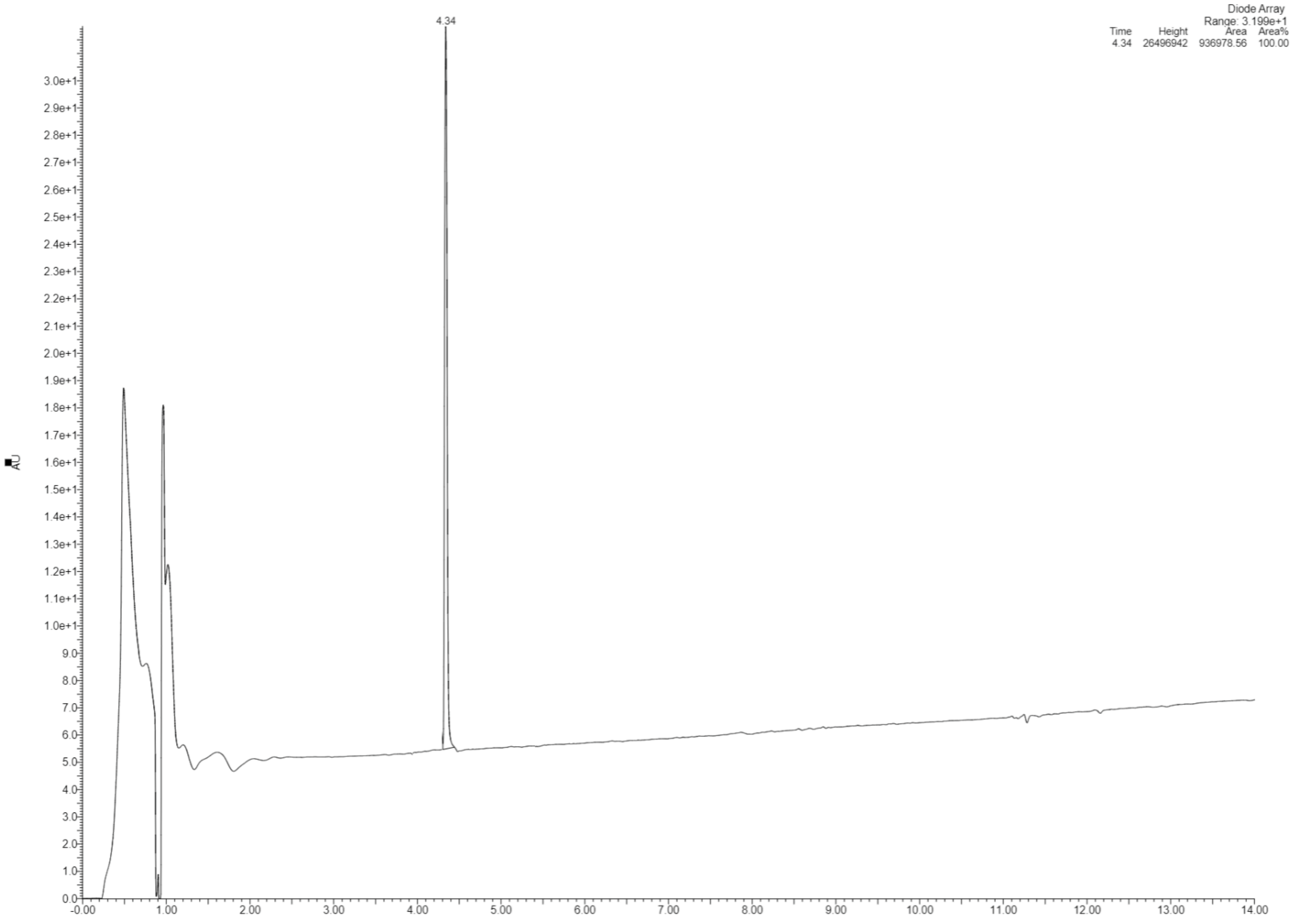

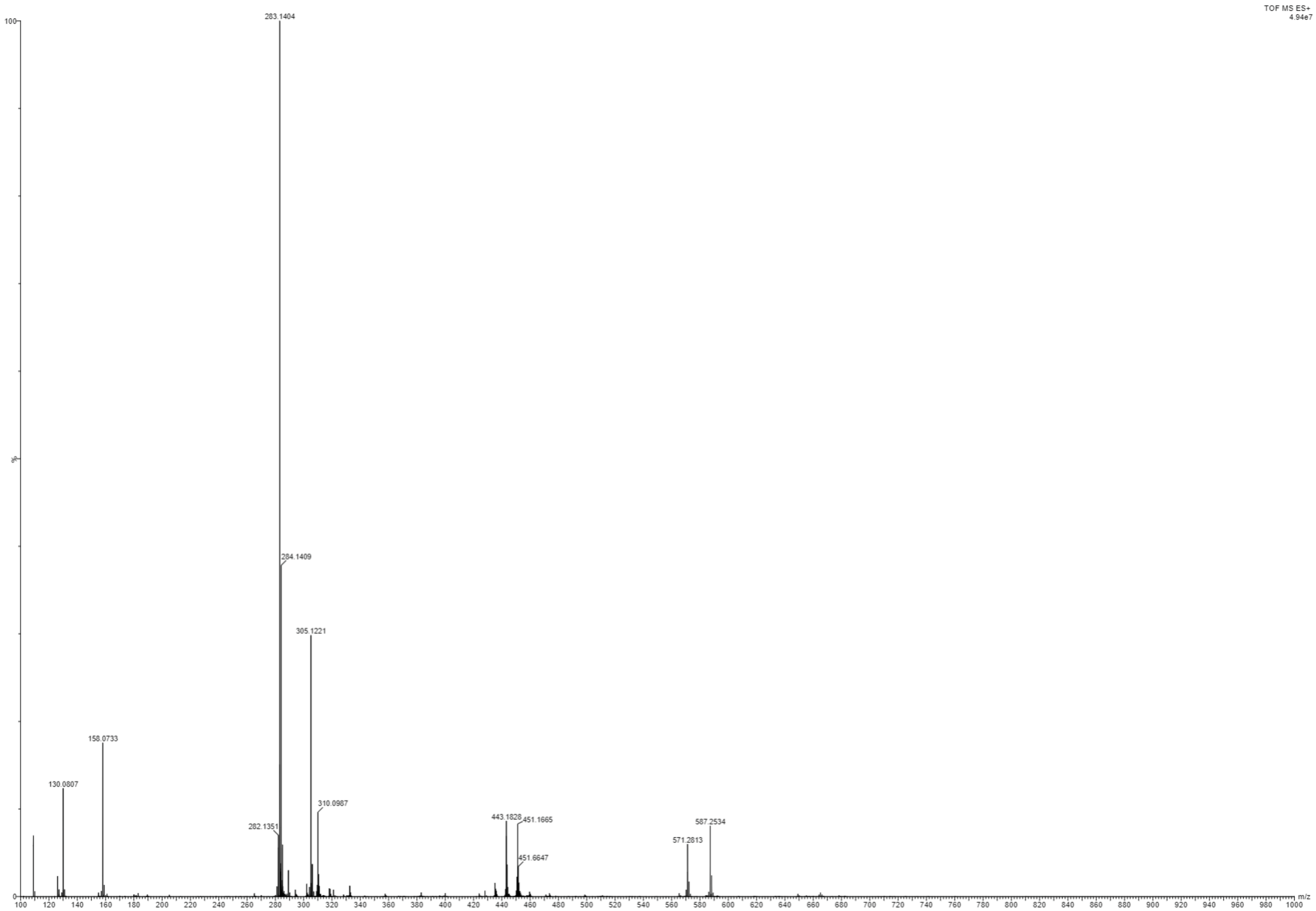
TOF MS ES+
4.94e7
283.1404
284.1409
305.1221
158.0733
130.0807
310.0987
282.1351
443.1828
451.1665
451.6647
571.2813
587.2534
%
m/z

(R)-2-(2,5-dioxopyrrolidin-1-yl-3,3,4,4-d_4)-*N*-((3-fluorophenyl)methyl-d_2)propanamide - **d_6-(R)-AS-7**

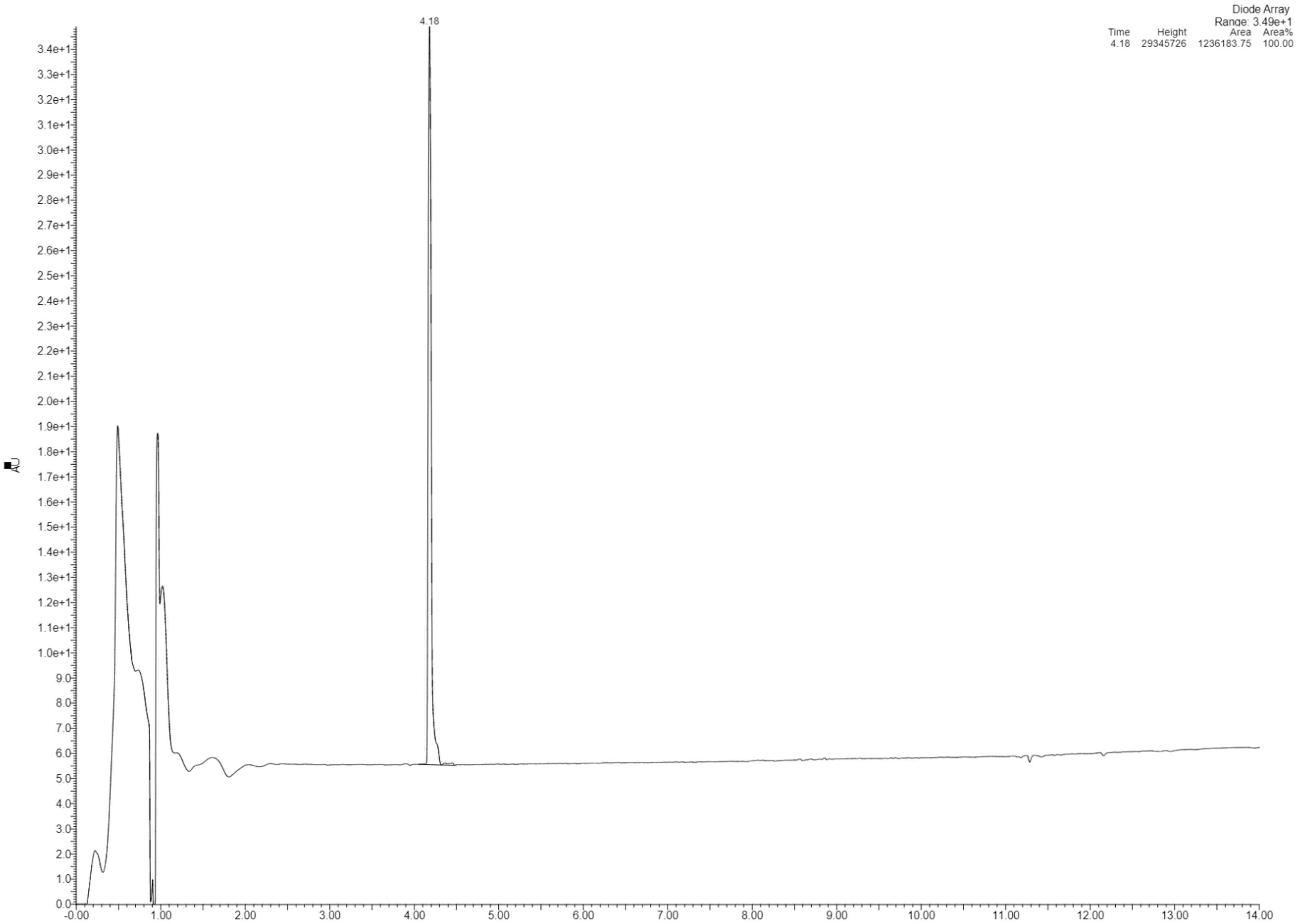

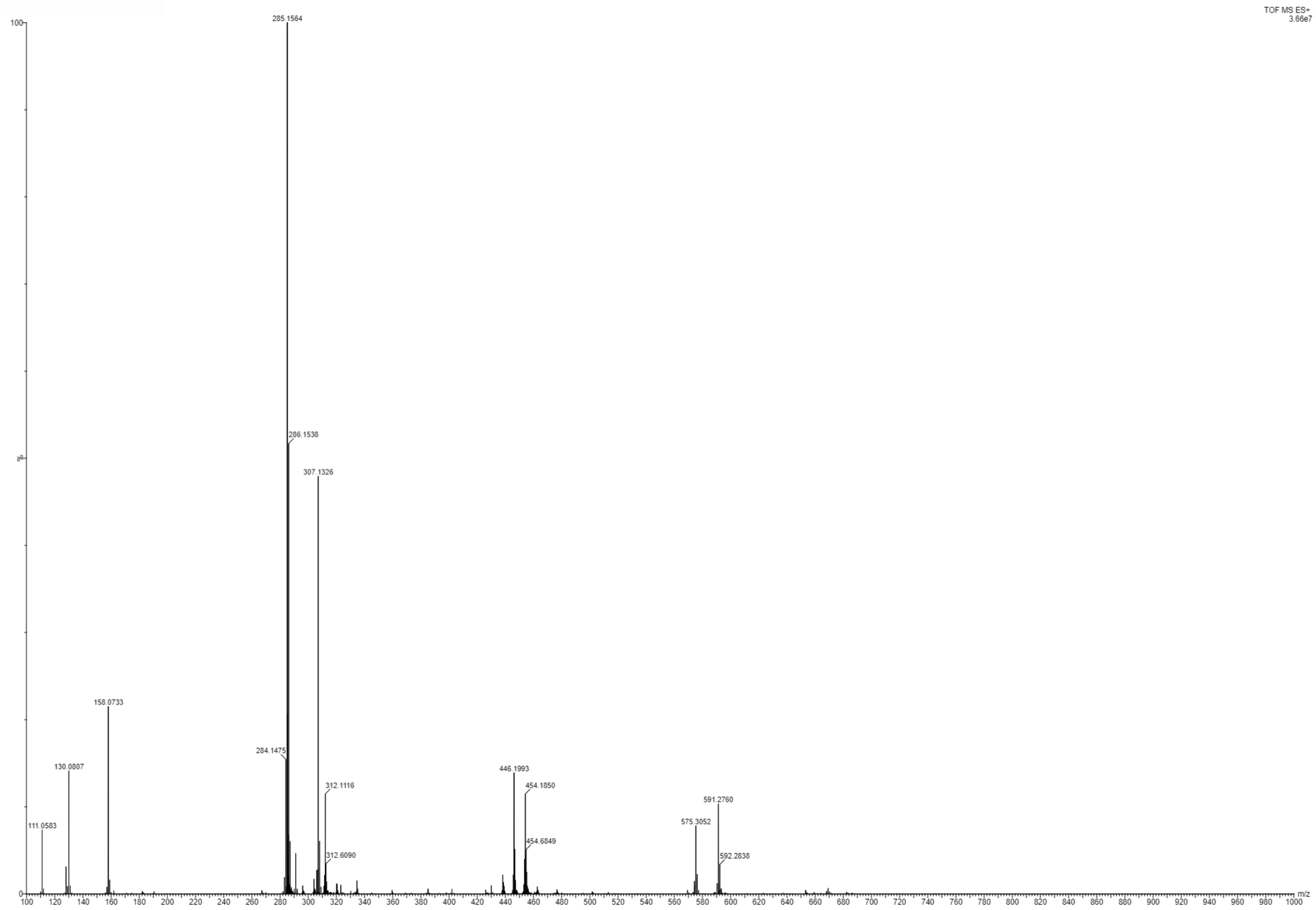
TOF MS ES+
3.66e7
285.1564
286.1538
307.1326
158.0733
284.1475
130.0807
312.1116
111.0583
446.1993
454.1850
454.6849
312.6090
575.3052
591.2760
592.2838
m/z
100
%
0

^{1}H NMR and ^{13}C NMR spectra for final compounds

Overlay of ^{1}H NMR spectra for **(*R*)-AS-1**, **d_4-(*R*)-AS-1** and **d_{11}-(*R*)-AS-1**, with magnified regions highlighting signal differences resulting from deuterium incorporation.

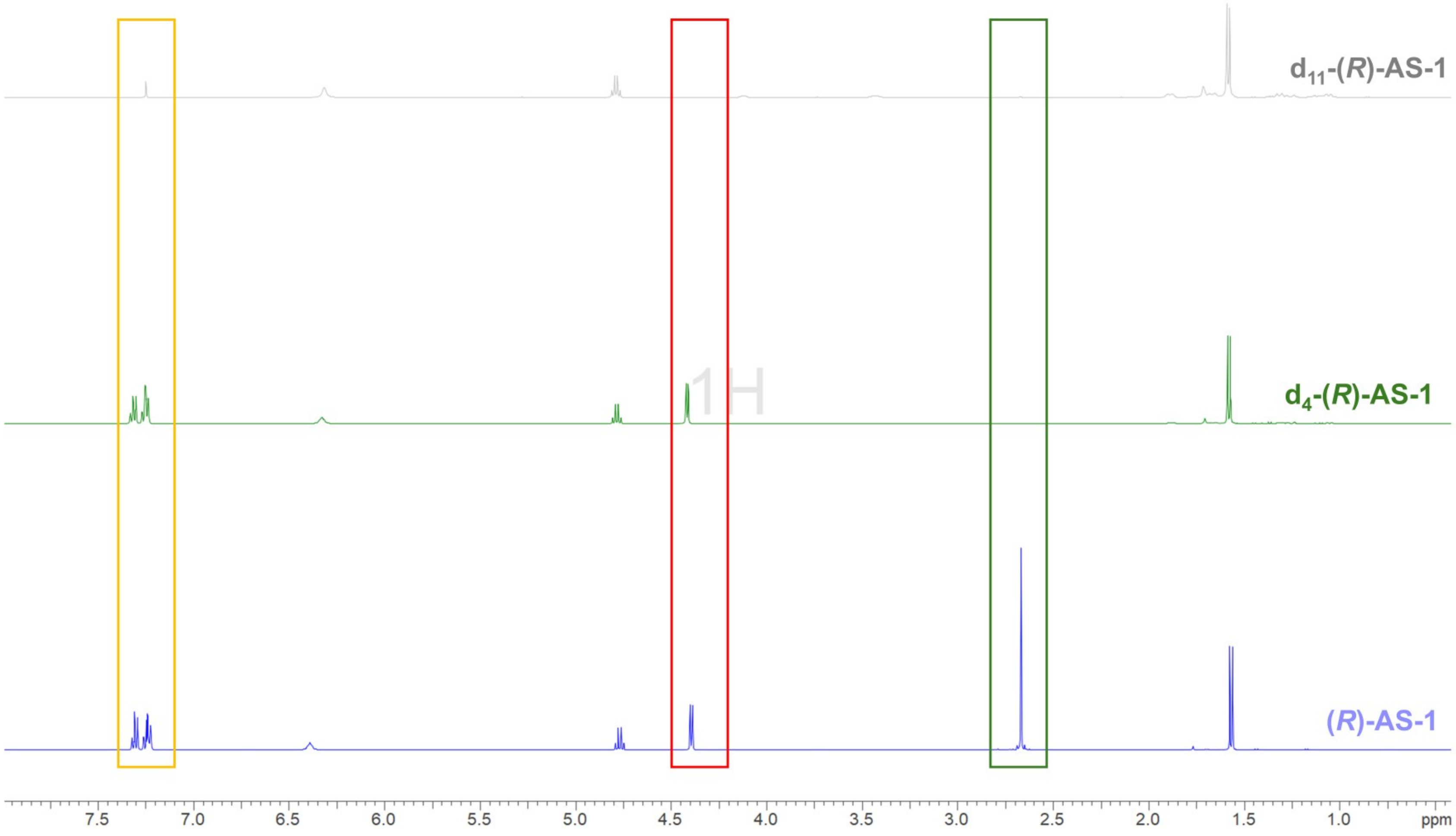

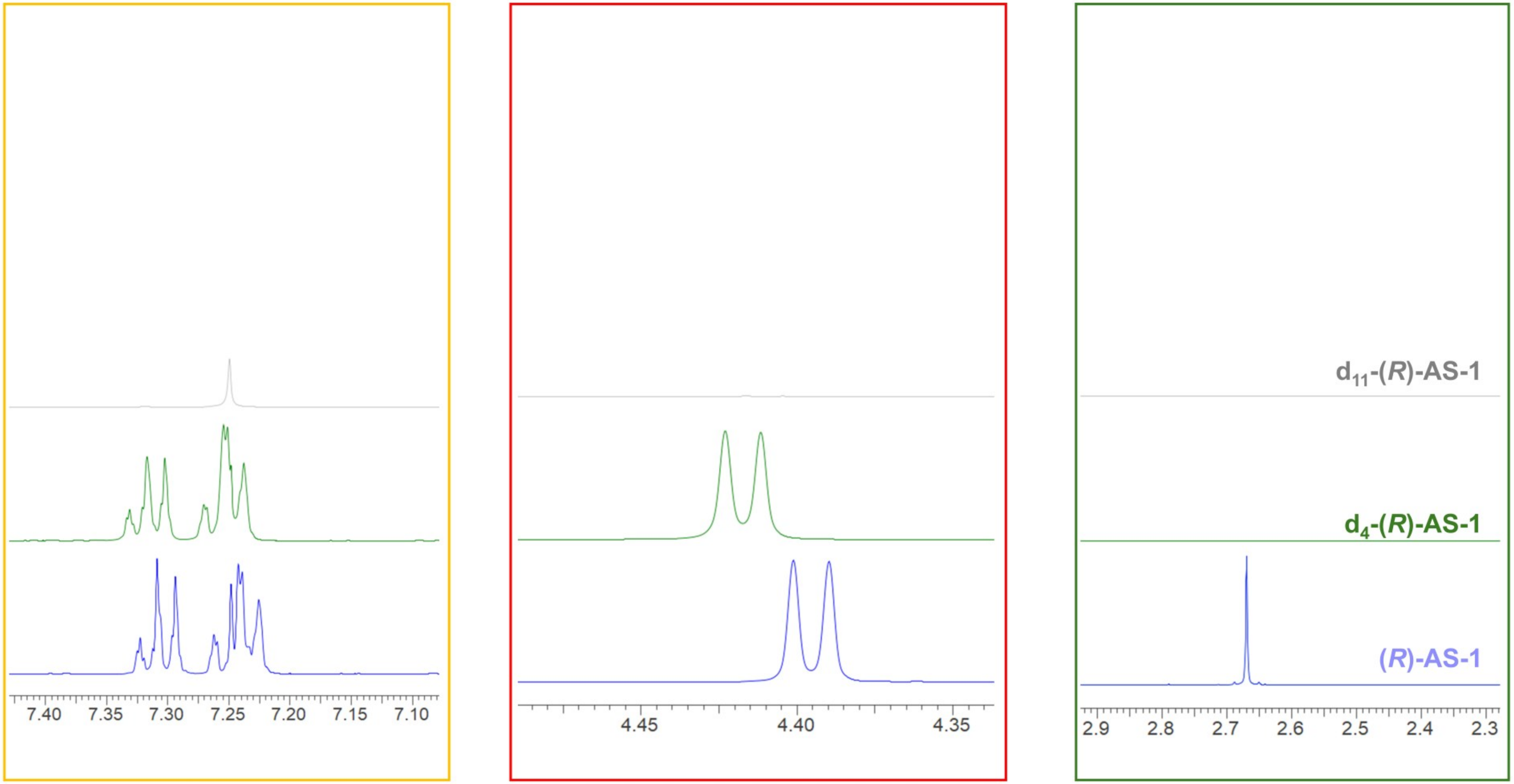

7.40 7.35 7.30 7.25 7.20 7.15 7.10
4.45 4.40 4.35
d11-(R)-AS-1
d4-(R)-AS-1
(R)-AS-1
2.9 2.8 2.7 2.6 2.5 2.4 2.3

(R)-N-benzyl-2-(2,5-dioxopyrrolidin-1-yl-3,3,4,4-d_4)propanamide - **d_4-(R)-AS-1** ^{1}HNMR

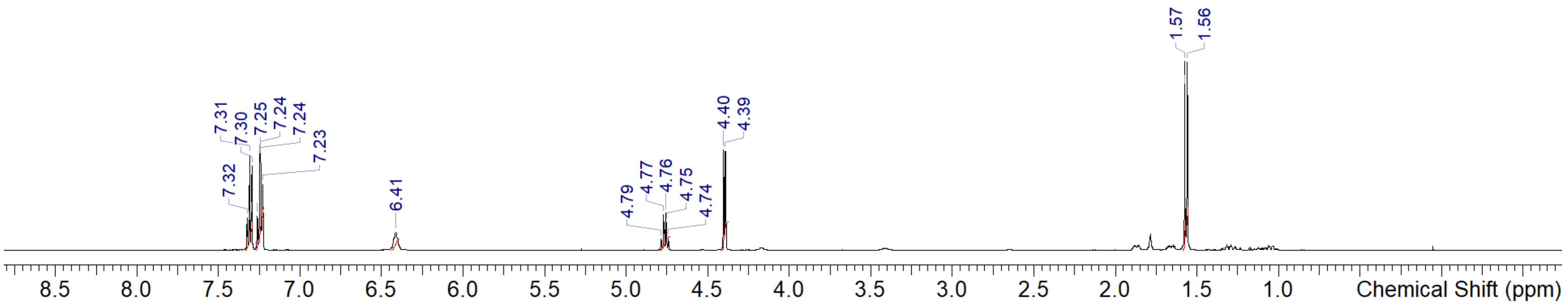

(*R*)-*N*-benzyl-2-(2,5-dioxopyrrolidin-1-yl-3,3,4,4-d_4)propanamide - **d_4-(*R*)-AS-1** ^{13}CNMR

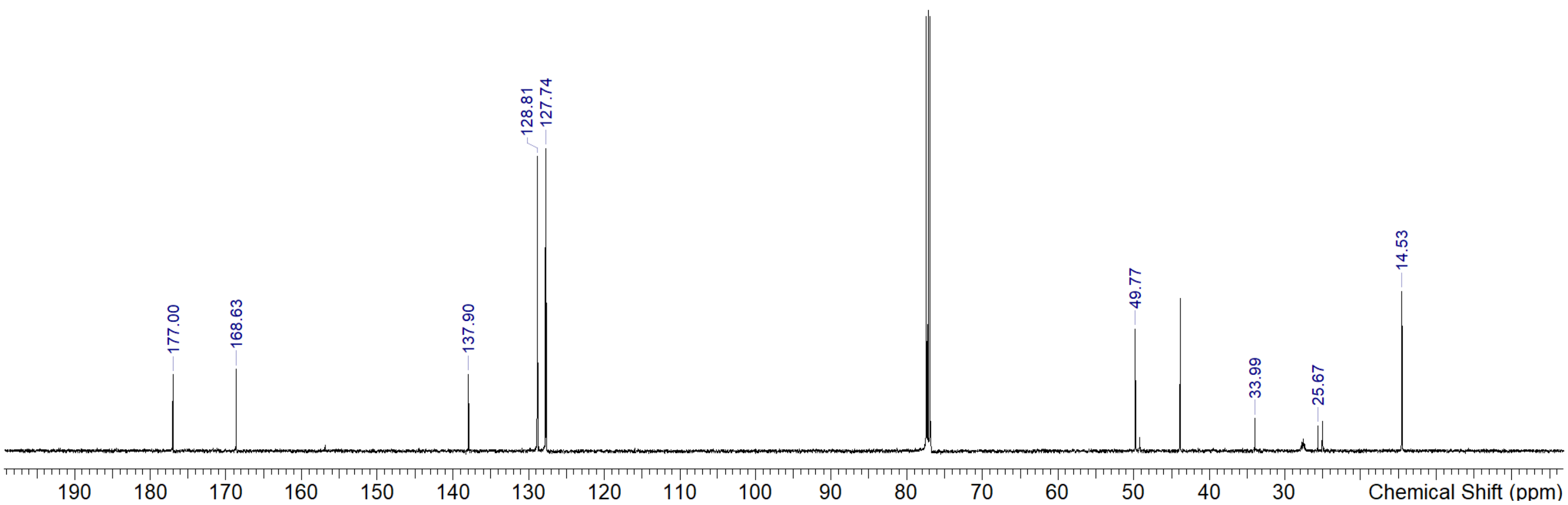

(*R*)-2-(2,5-dioxopyrrolidin-1-yl)-*N*-((phenyl-d_5)methyl)propanamide - **d_5-(*R*)-AS-1** ^{1}HNMR

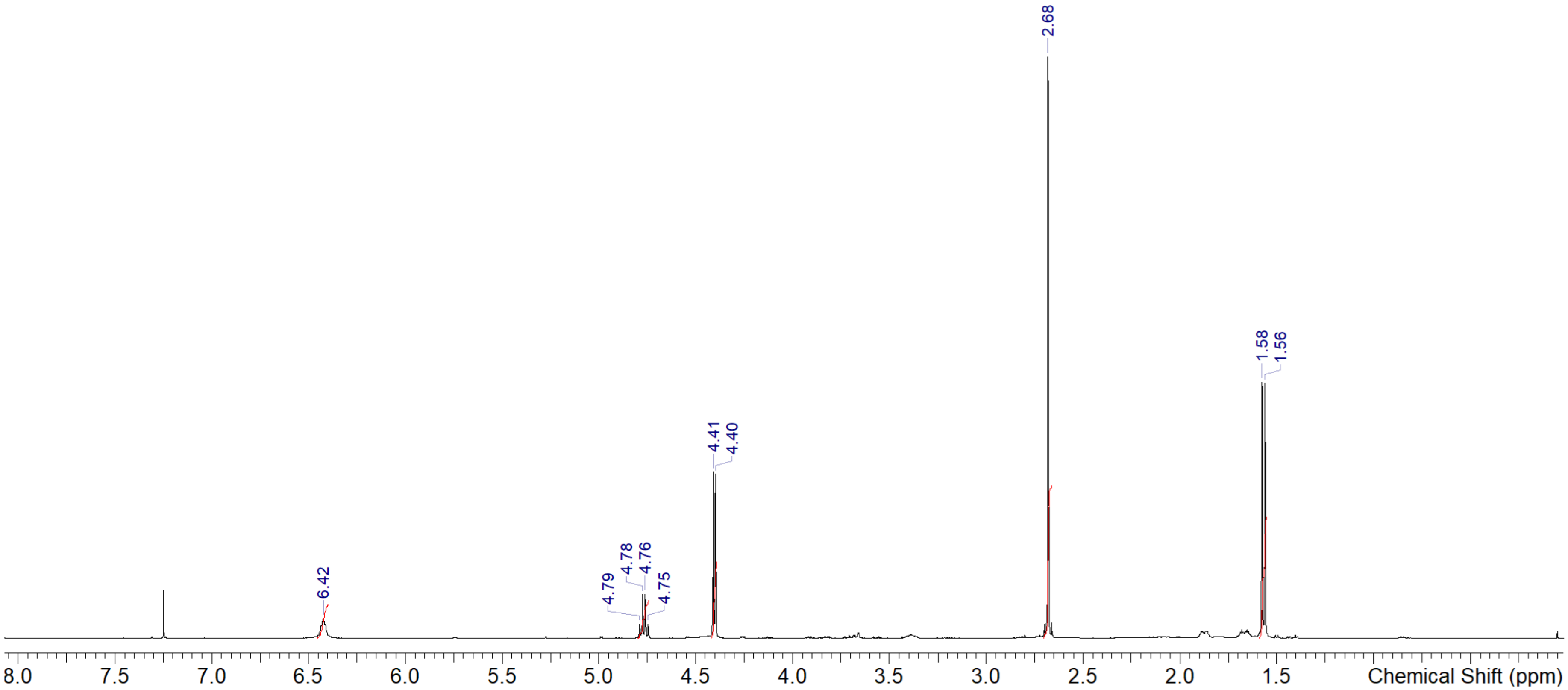

(*R*)-2-(2,5-dioxopyrrolidin-1-yl)-*N*-((phenyl-d_5)methyl)propanamide - **d_5-(*R*)-AS-1** ^{13}CNMR

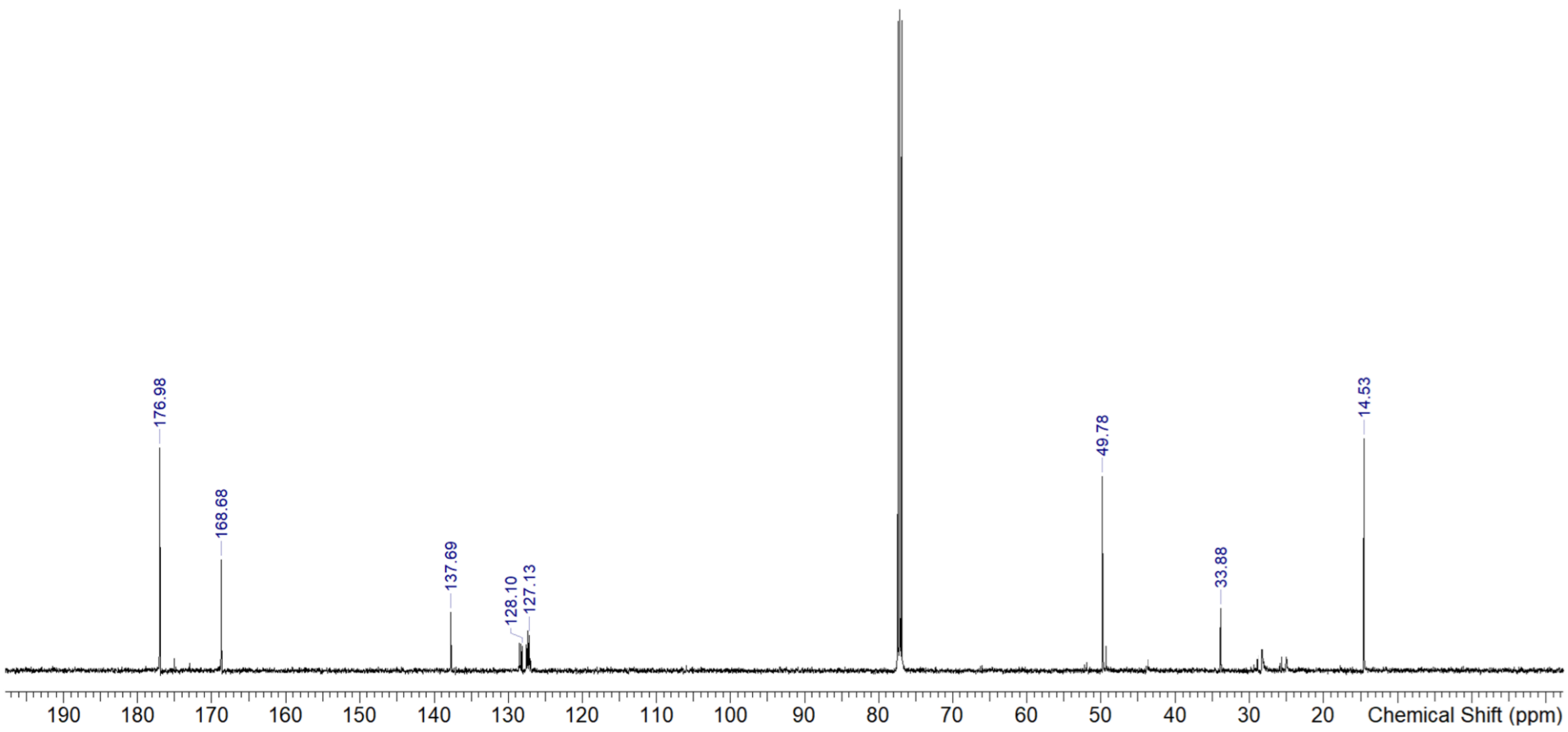

(*R*)-2-(2,5-dioxopyrrolidin-1-yl-3,3,4,4-d_4)-*N*-(phenylmethyl-d_2)propanamide - **d_6-(*R*)-AS-1** ^{1}HNMR

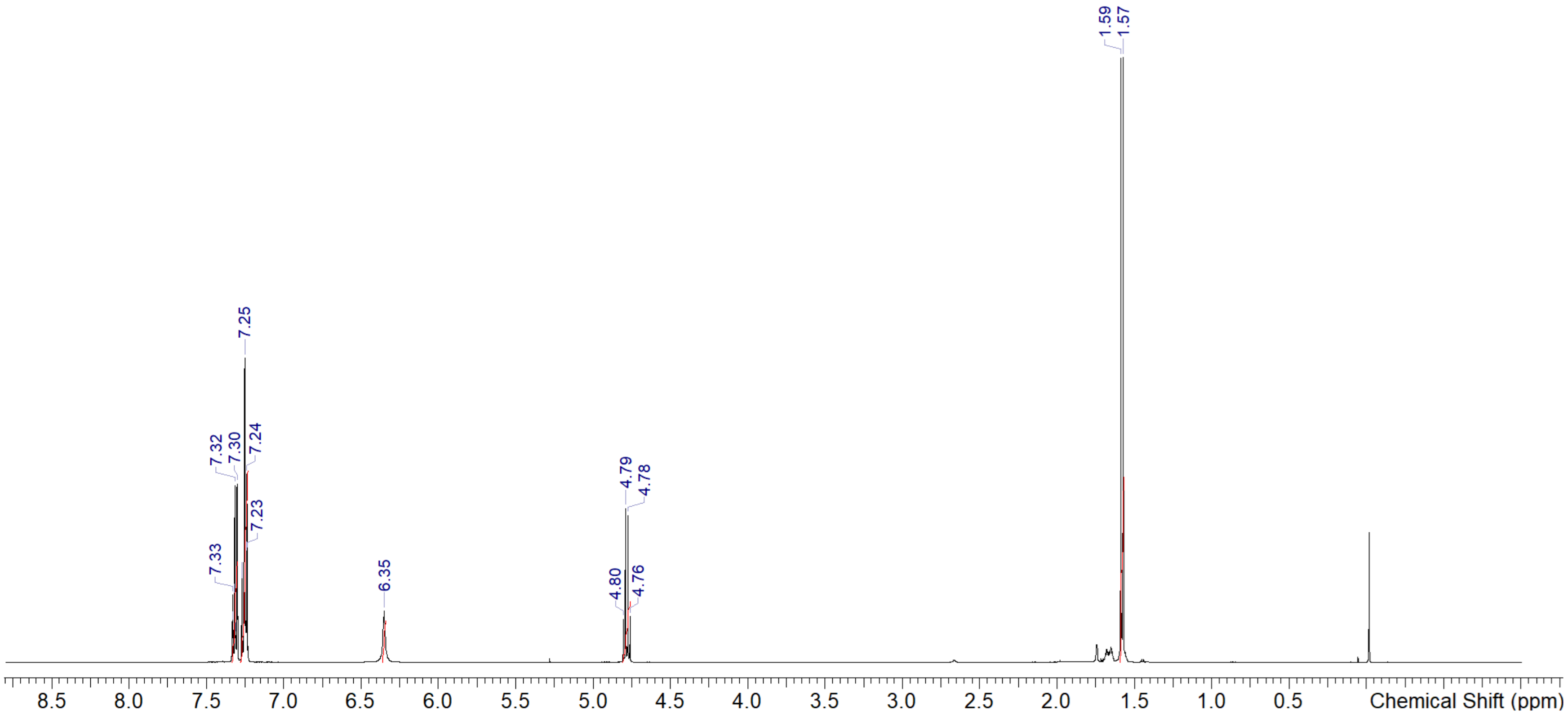

(R)-2-(2,5-dioxopyrrolidin-1-yl-3,3,4,4-d_4)-N-(phenylmethyl-d_2)propanamide - **d_6-(R)-AS-1** ^{13}CNMR

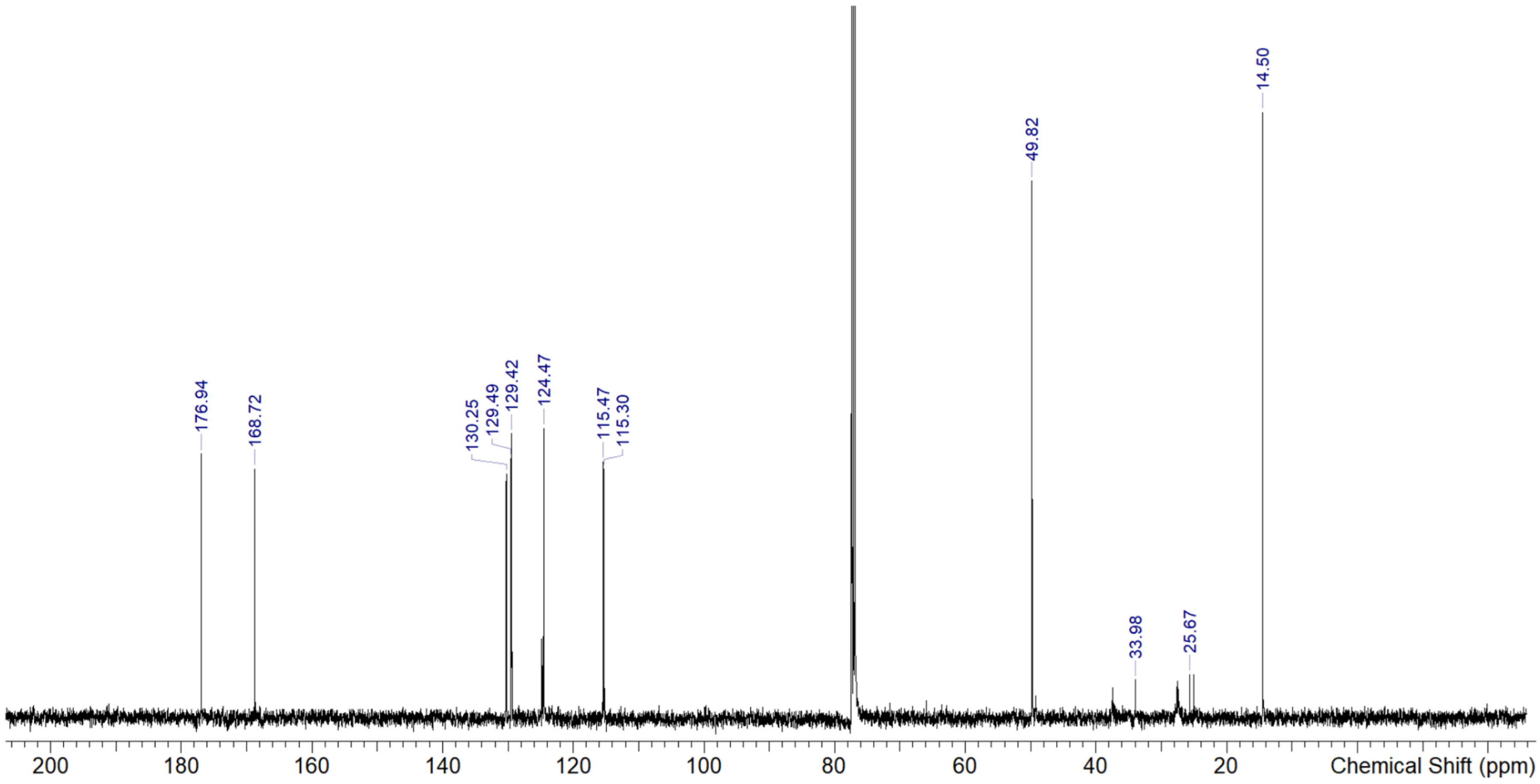

(*R*)-2-(2,5-dioxopyrrolidin-1-yl-3,3,4,4-d_4)-*N*-((phenyl-d_5)methyl)propanamide - **d_9-(*R*)-AS-1** ^{1}HNMR

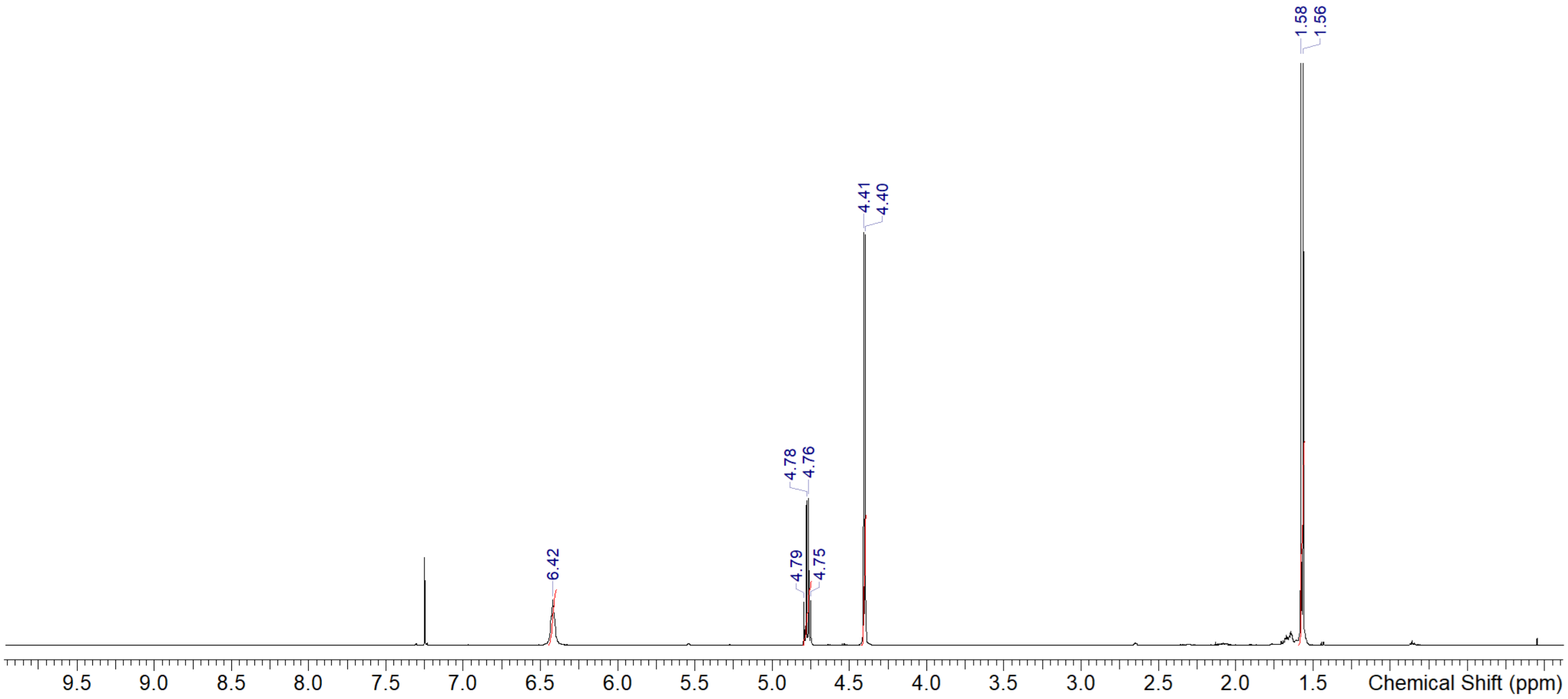

(*R*)-2-(2,5-dioxopyrrolidin-1-yl-3,3,4,4-d_4)-*N*-((phenyl-d_5)methyl)propanamide - **d_9-(*R*)-AS-1** ^{13}CNMR

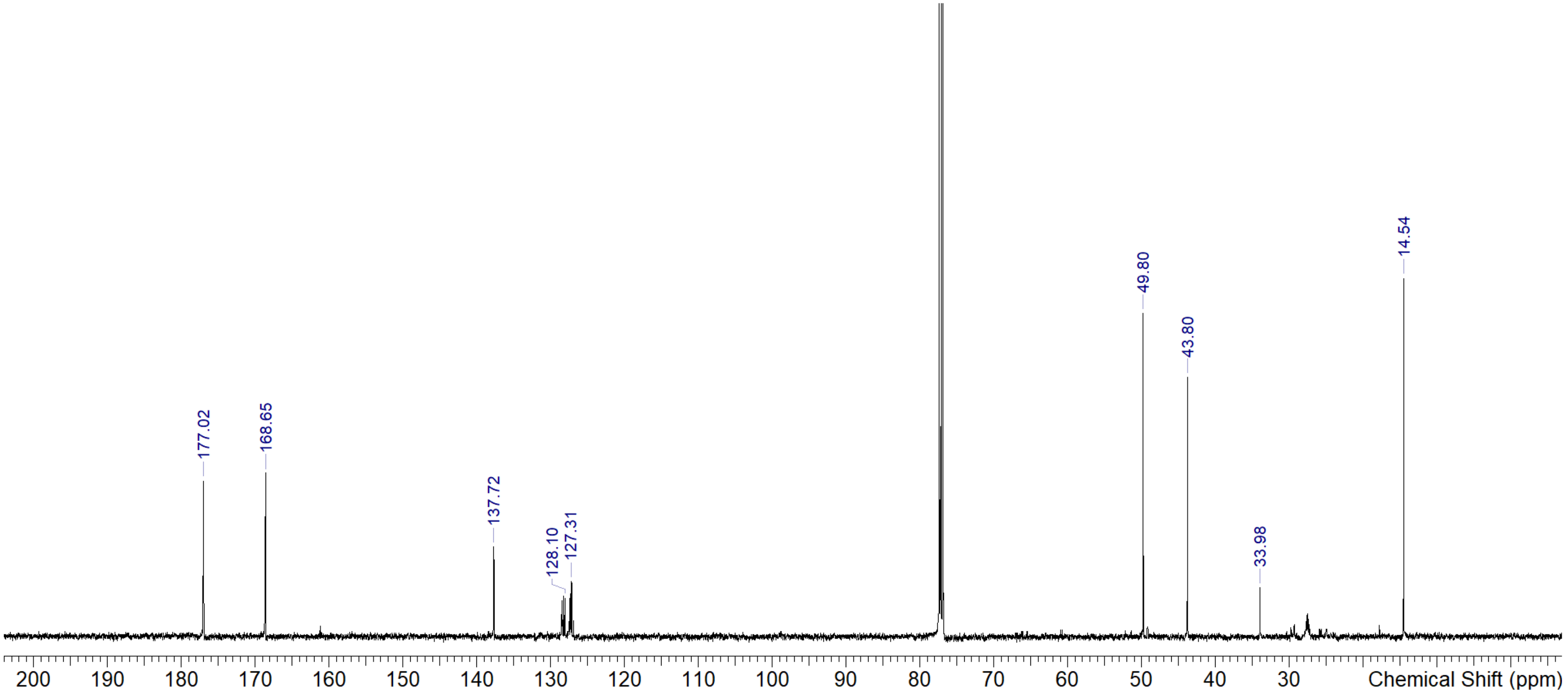

(*R*)-2-(2,5-dioxopyrrolidin-1-yl-3,3,4,4-d_4)-*N*-((phenyl-d_5)methyl-d_2)propanamide - **d_{11}-(*R*)-AS-1** ^{1}HNMR

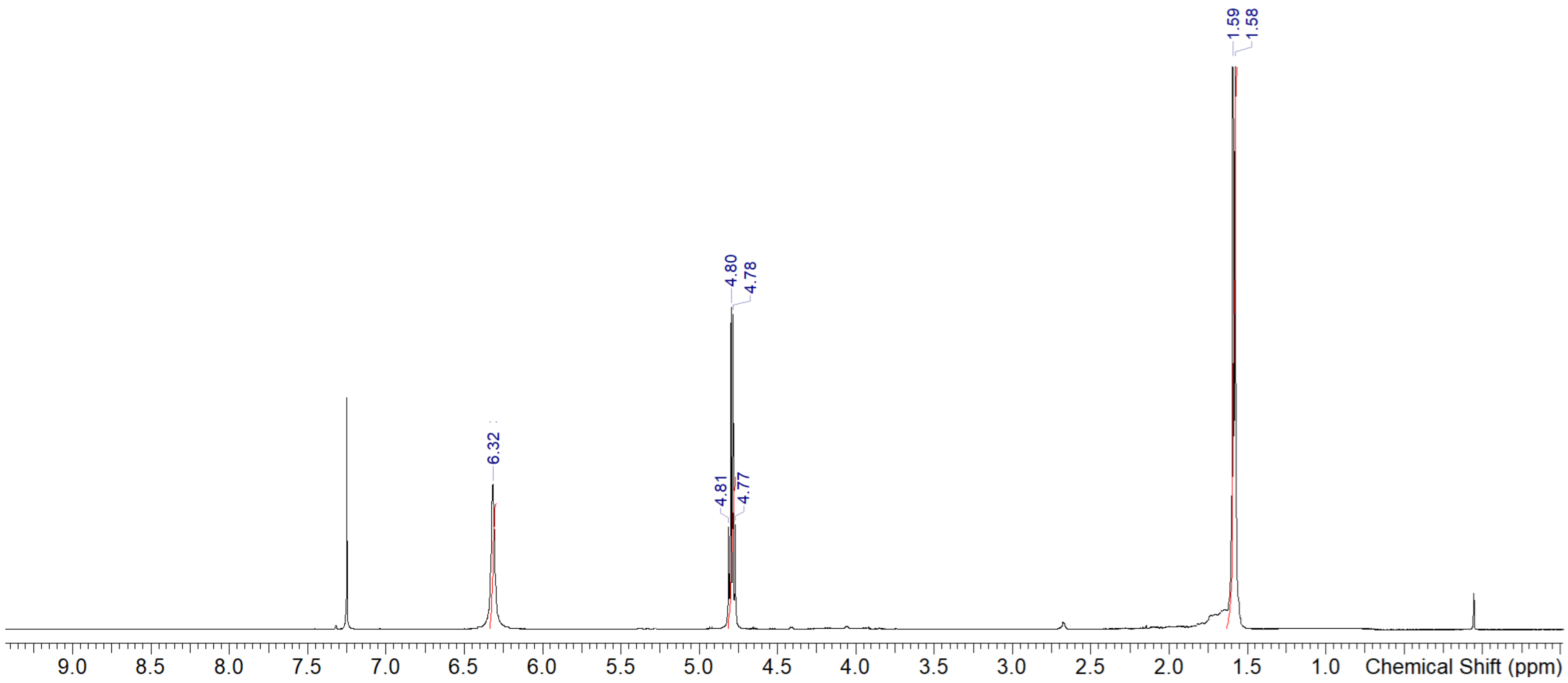

(*R*)-2-(2,5-dioxopyrrolidin-1-yl-3,3,4,4-d_4)-*N*-((phenyl-d_5)methyl-d_2)propanamide - **d_{11}-(*R*)-AS-1** ^{13}CNMR

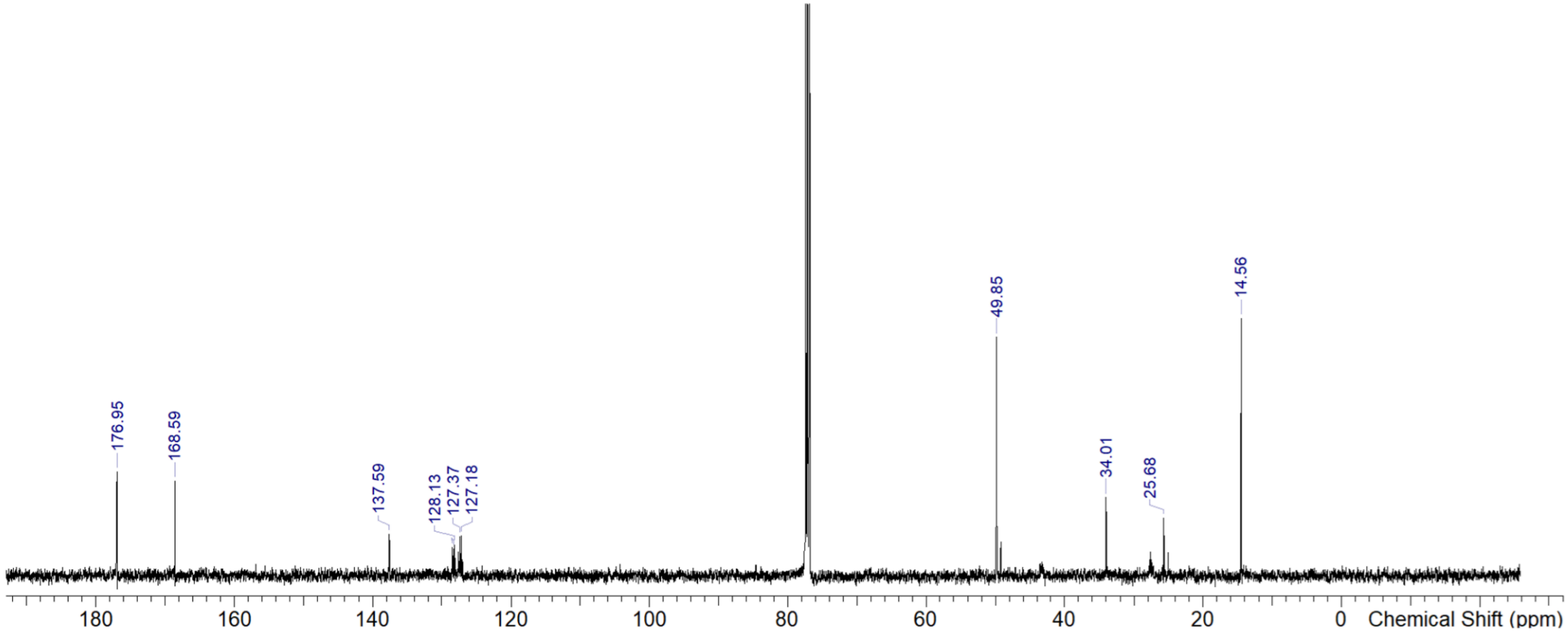

(*R*)-2-(2,5-dioxopyrrolidin-1-yl-3,3,4,4-d_4)-*N*-(2-fluorobenzyl)propanamide - **d_4-(*R*)-AS-7** ^{1}HNMR

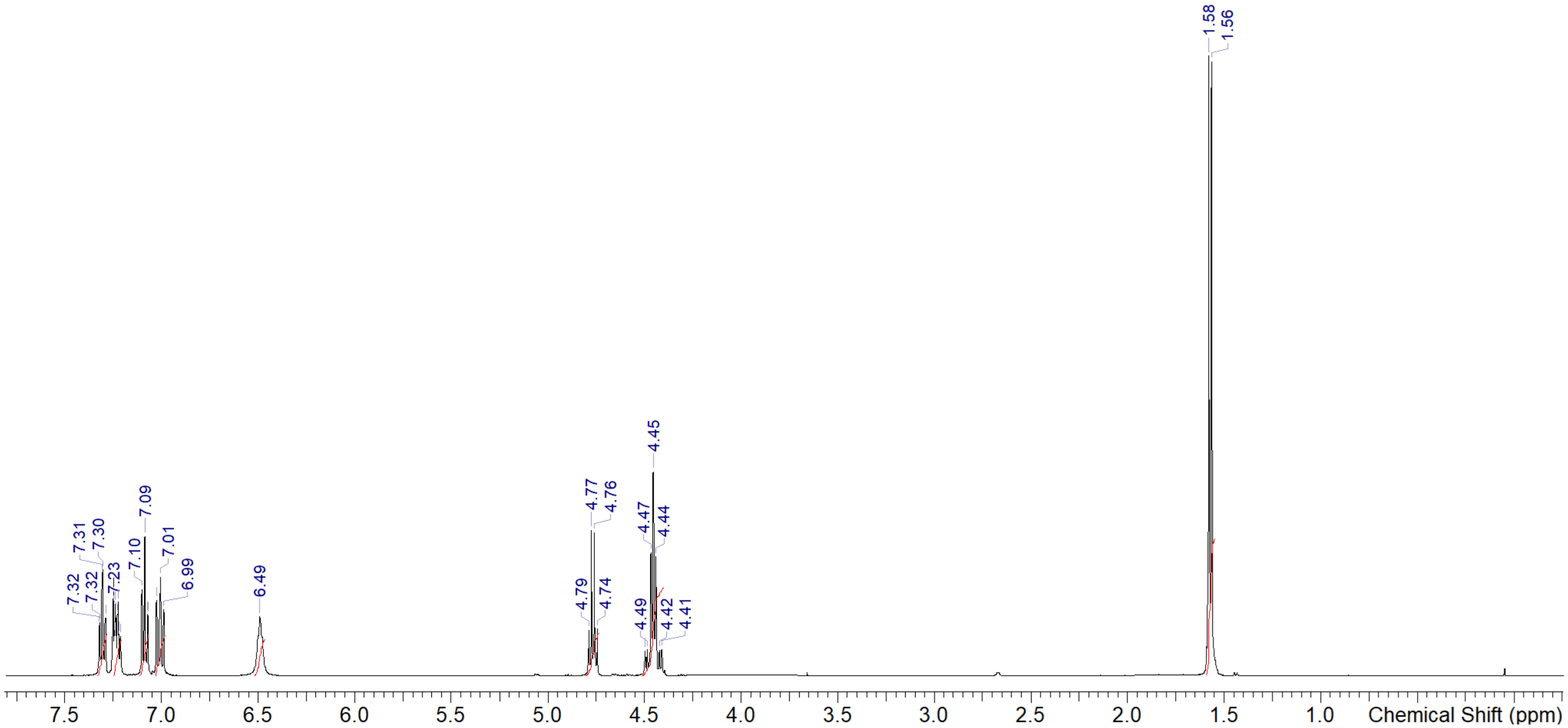

(*R*)-2-(2,5-dioxopyrrolidin-1-yl-3,3,4,4-d_4)-*N*-(2-fluorobenzyl)propanamide - **d_4-(*R*)-AS-7** ^{13}CNMR

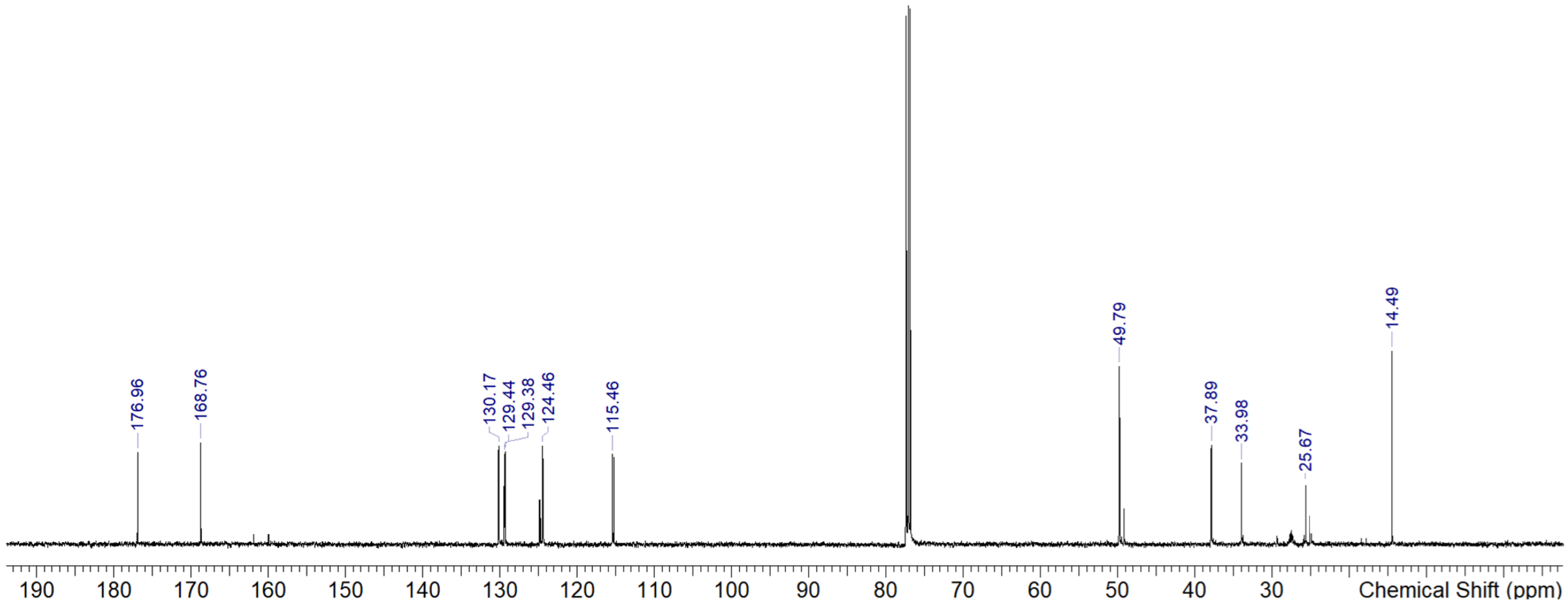

(*R*)-2-(2,5-dioxopyrrolidin-1-yl-3,3,4,4-d_4)-*N*-((2-fluorophenyl)methyl-d_2)propanamide - **d_6-(*R*)-AS-7** ^{1}HNMR

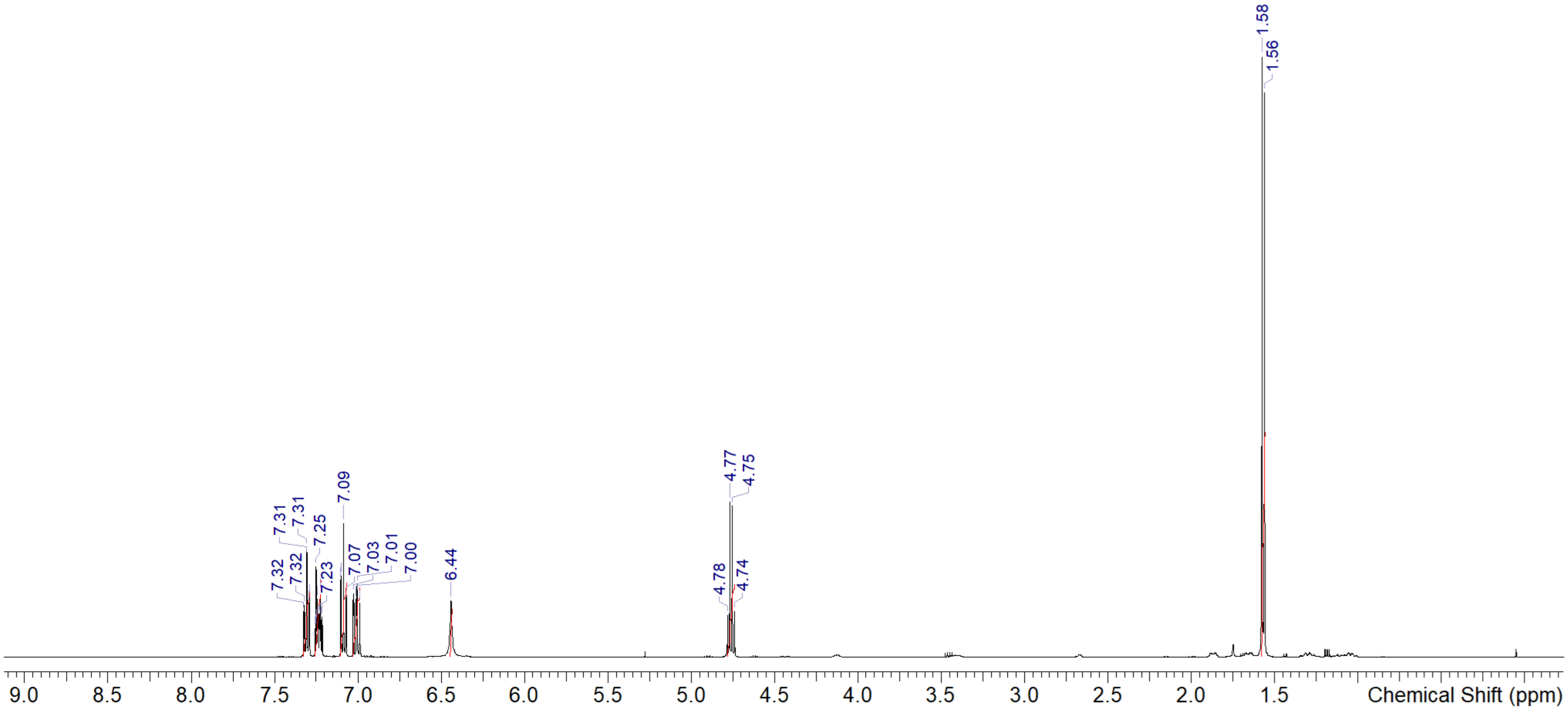

(*R*)-2-(2,5-dioxopyrrolidin-1-yl-3,3,4,4-d_4)-*N*-((2-fluorophenyl)methyl-d_2)propanamide - **d_6-(*R*)-AS-7** ^{13}CNMR

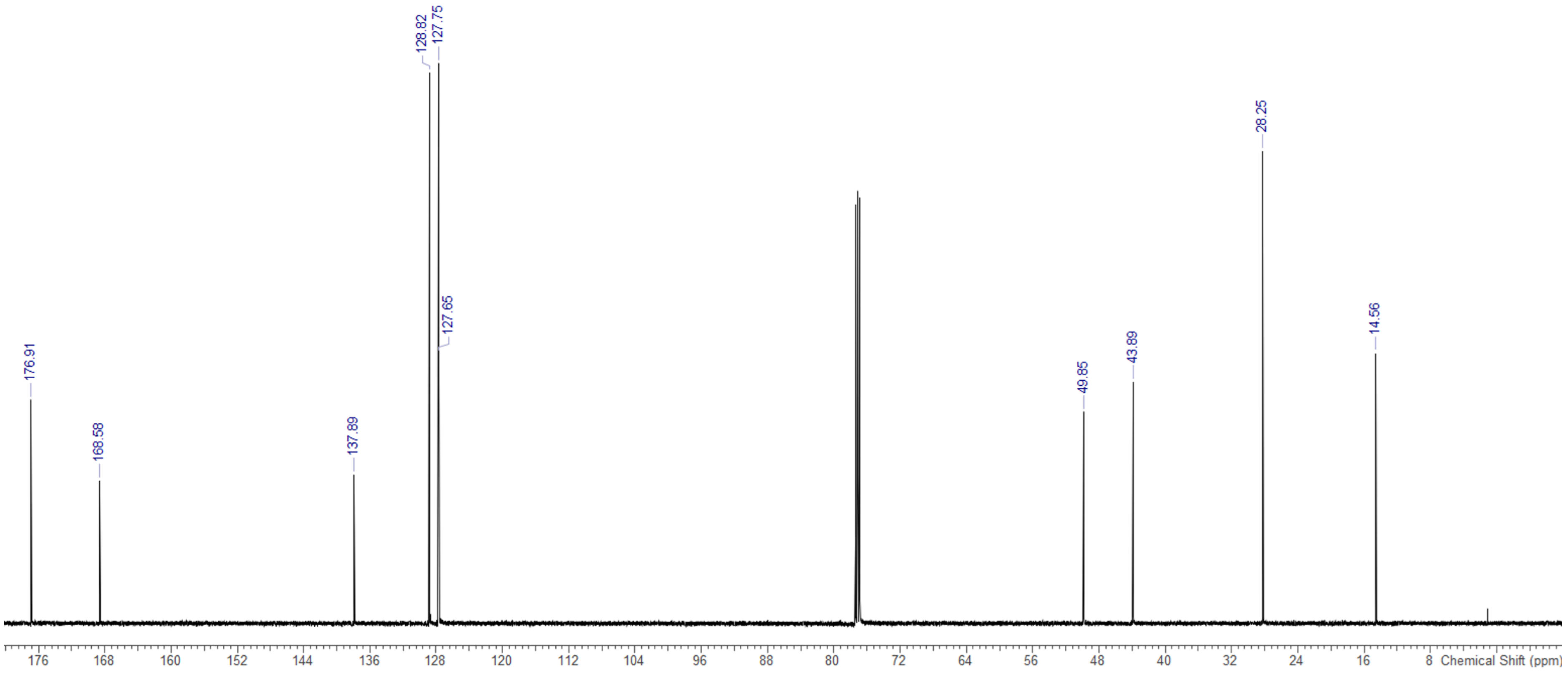

Chiral SFC chromatograms

(*R*)-*N*-benzyl-2-(2,5-dioxopyrrolidin-1-yl-3,3,4,4-d_4)propanamide - **d_4-(*R*)-AS-1**

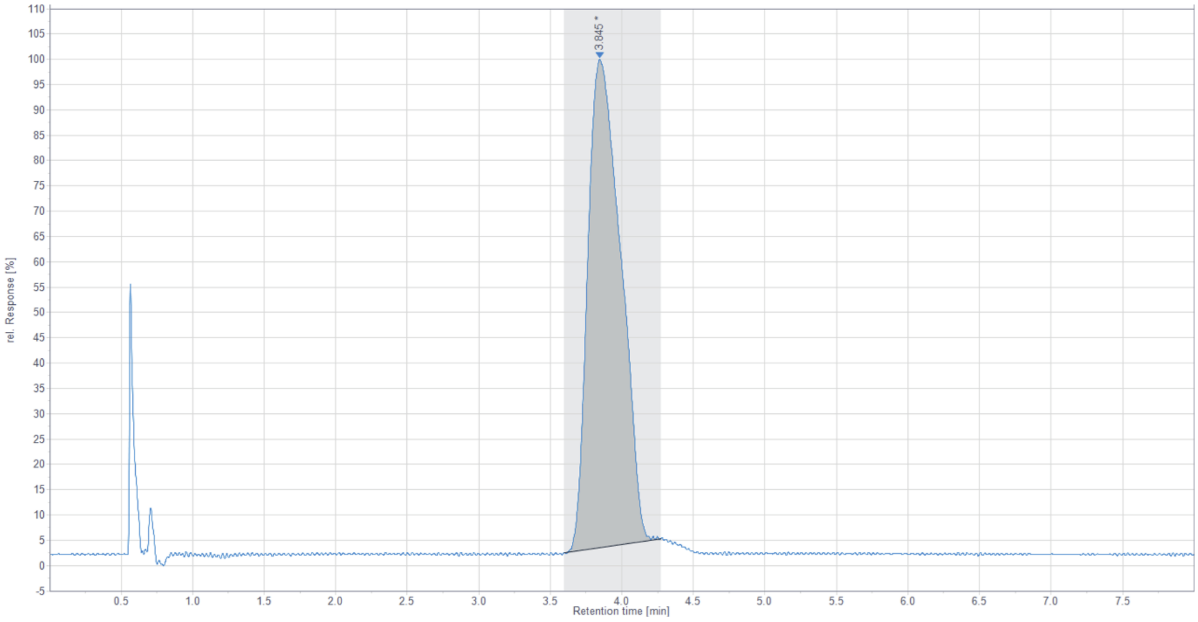

(*R*)-*N*-benzyl-2-(2,5-dioxopyrrolidin-1-yl-3,3,4,4-d_4)propanamide - **d_4-(*R*,*S*)-AS-1**

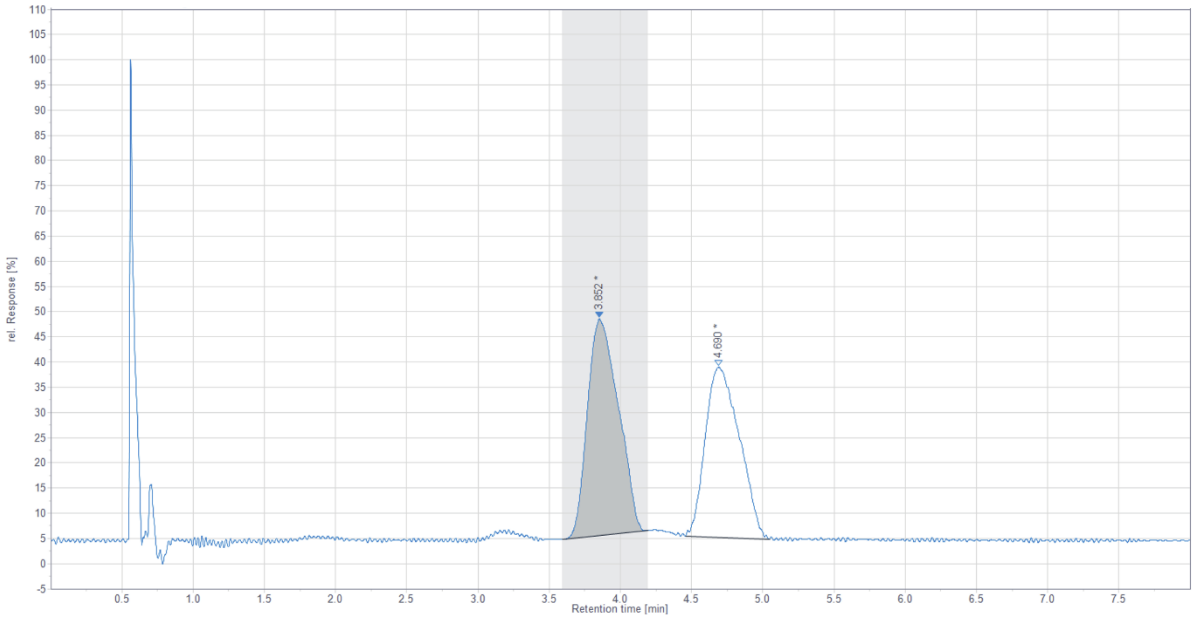

(*R*)-2-(2,5-dioxopyrrolidin-1-yl)-*N*-((phenyl-d_5)methyl)propanamide - **d_5-(*R*)-AS-1**

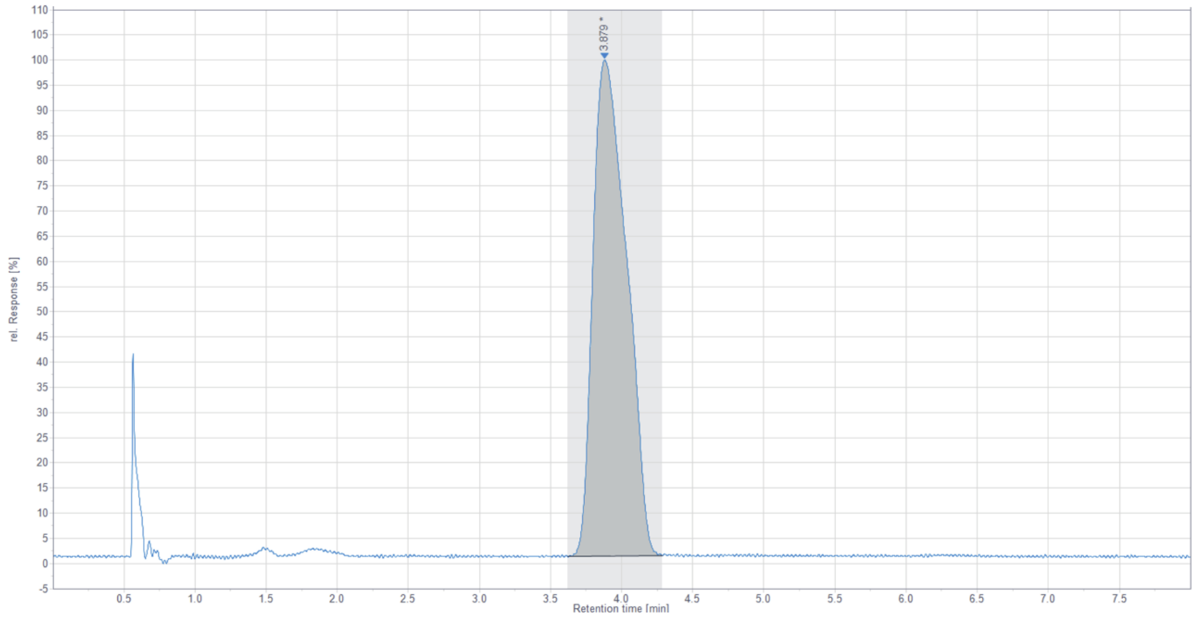

(*R*)-2-(2,5-dioxopyrrolidin-1-yl)-*N*-((phenyl-d_5)methyl)propanamide - **d_5-(*R*,*S*)-AS-1**

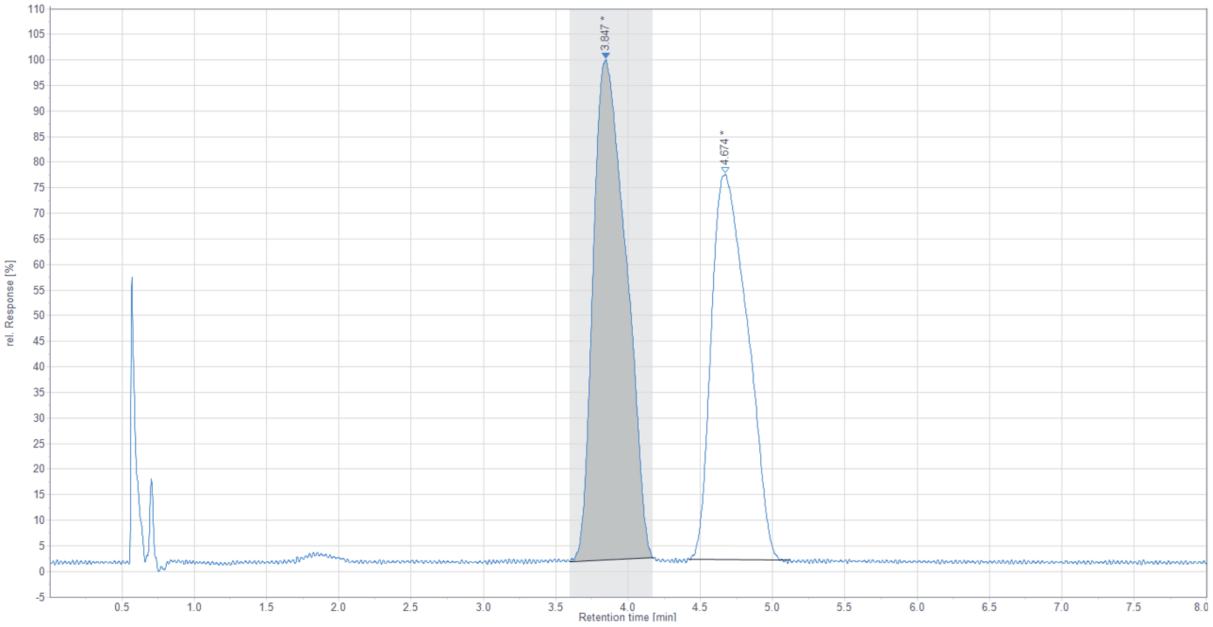

(*R*)-2-(2,5-dioxopyrrolidin-1-yl-3,3,4,4-d_4)-*N*-(phenylmethyl-d_2)propanamide - **d_6-(*R*)-AS-1**

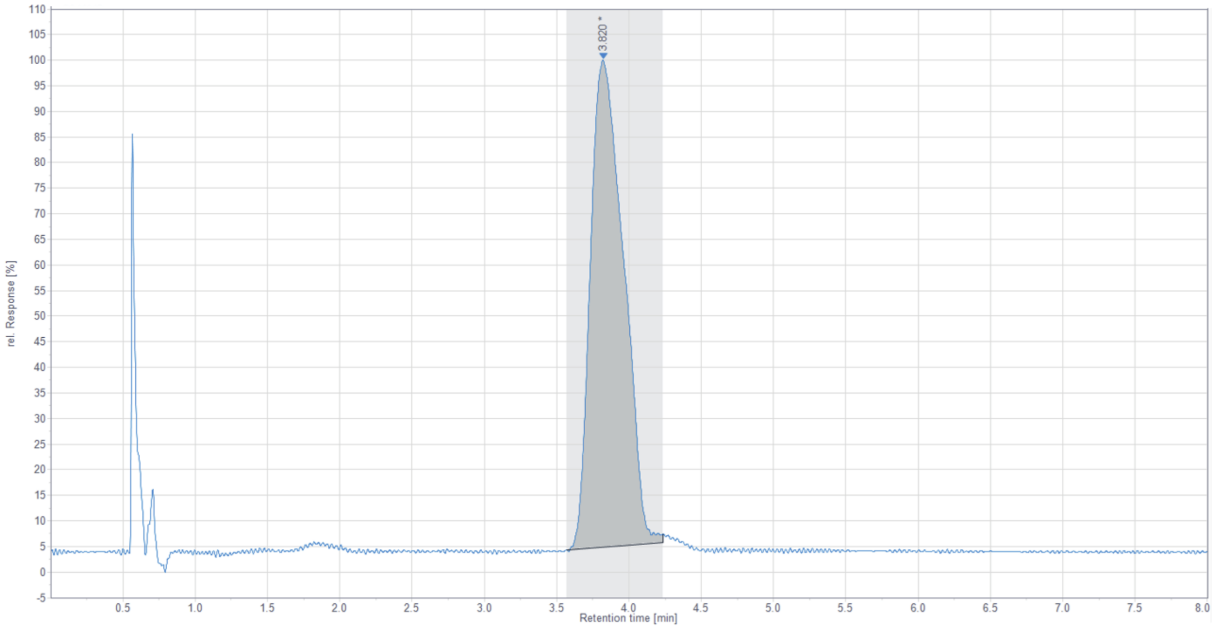

(*R*)-2-(2,5-dioxopyrrolidin-1-yl-3,3,4,4-d_4)-*N*-(phenylmethyl-d_2)propanamide - **d_6-(*R*,*S*)-AS-1**

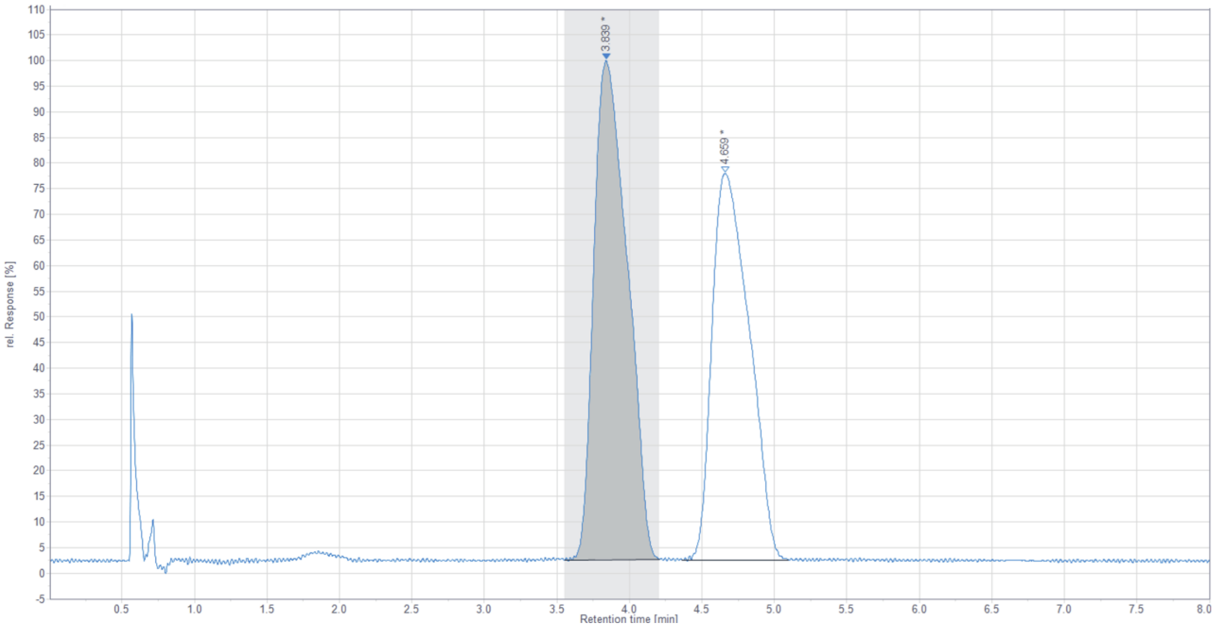

(*R*)-2-(2,5-dioxopyrrolidin-1-yl-3,3,4,4-d_4)-*N*-((phenyl-d_5)methyl)propanamide - **d_9-(*R*)-AS-1**

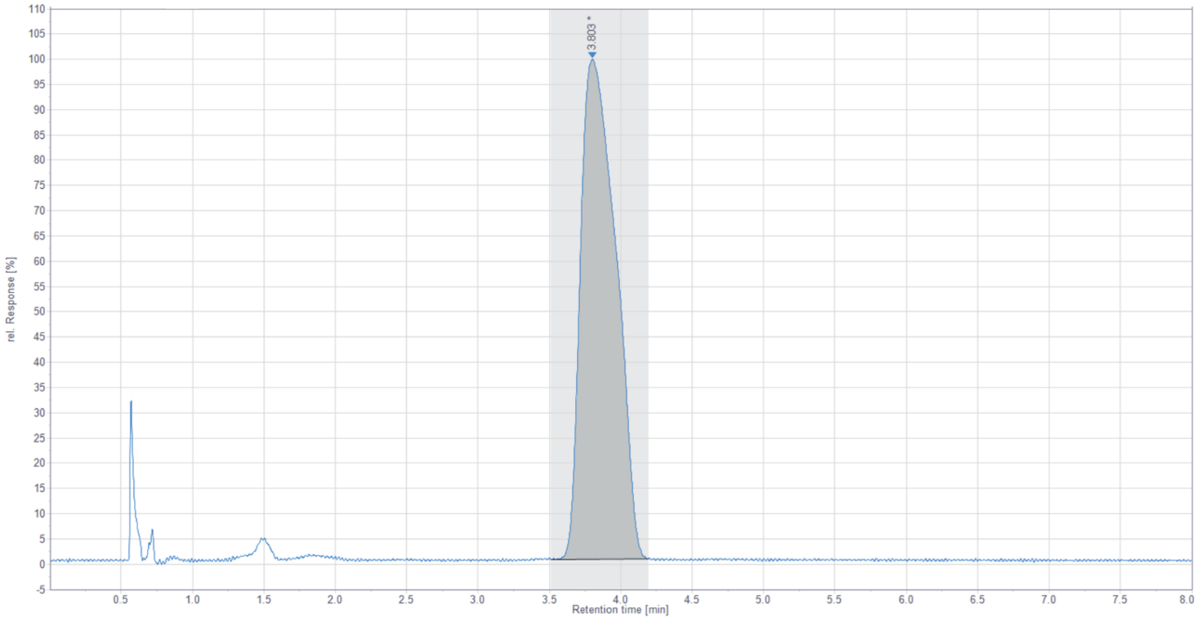

(*R*)-2-(2,5-dioxopyrrolidin-1-yl-3,3,4,4-d_4)-*N*-((phenyl-d_5)methyl)propanamide - **d_9-(*R*,*S*)-AS-1**

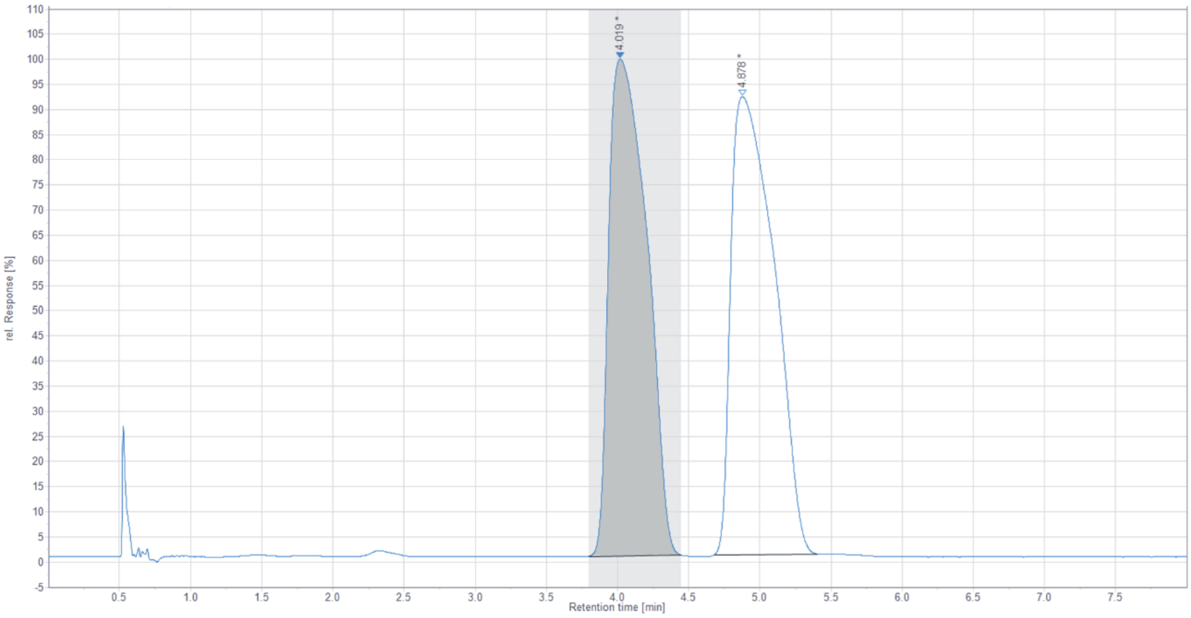

(R)-2-(2,5-dioxopyrrolidin-1-yl-3,3,4,4-d_4)-N-((phenyl-d_5)methyl-d_2)propanamide - **d_{11}-(R)-AS-1**

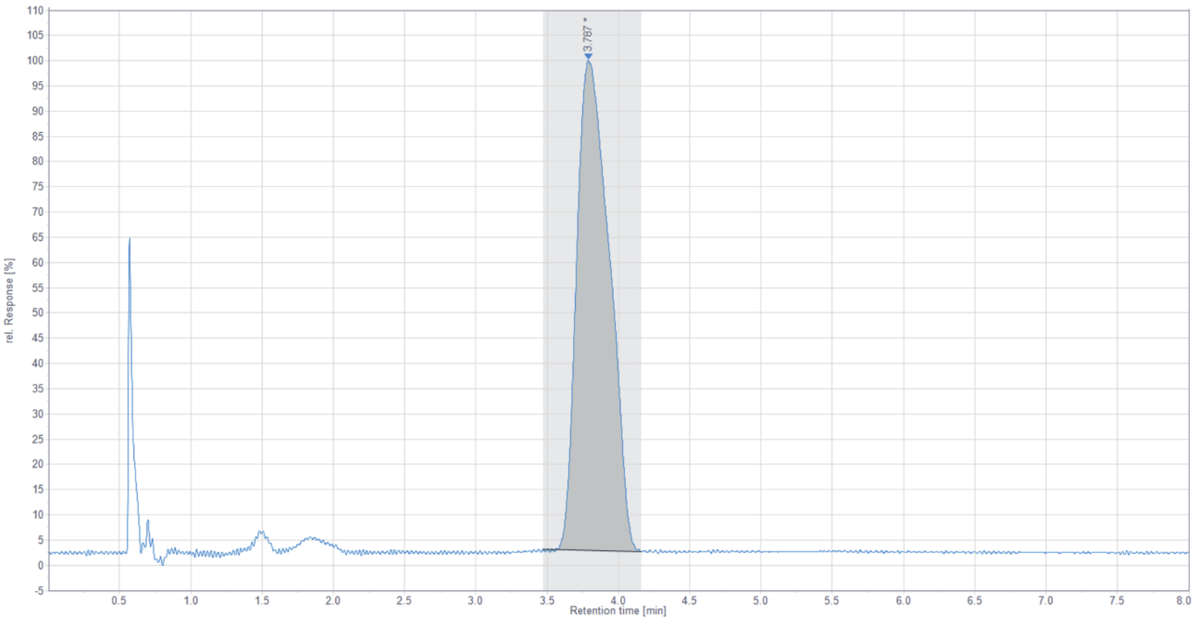

(R)-2-(2,5-dioxopyrrolidin-1-yl-3,3,4,4-d_4)-N-((phenyl-d_5)methyl-d_2)propanamide - **d_{11}-(R,S)-AS-1**

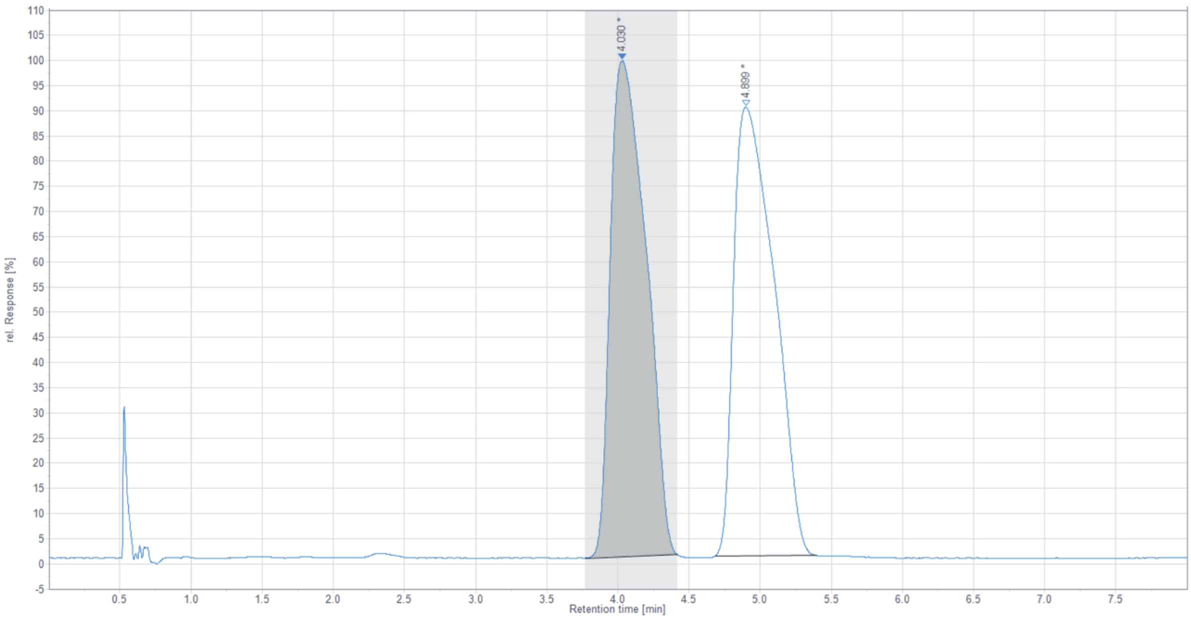

(R)-2-(2,5-dioxopyrrolidin-1-yl-3,3,4,4-d_4)-N-(2-fluorobenzyl)propanamide - **d_4-(R)-AS-7**

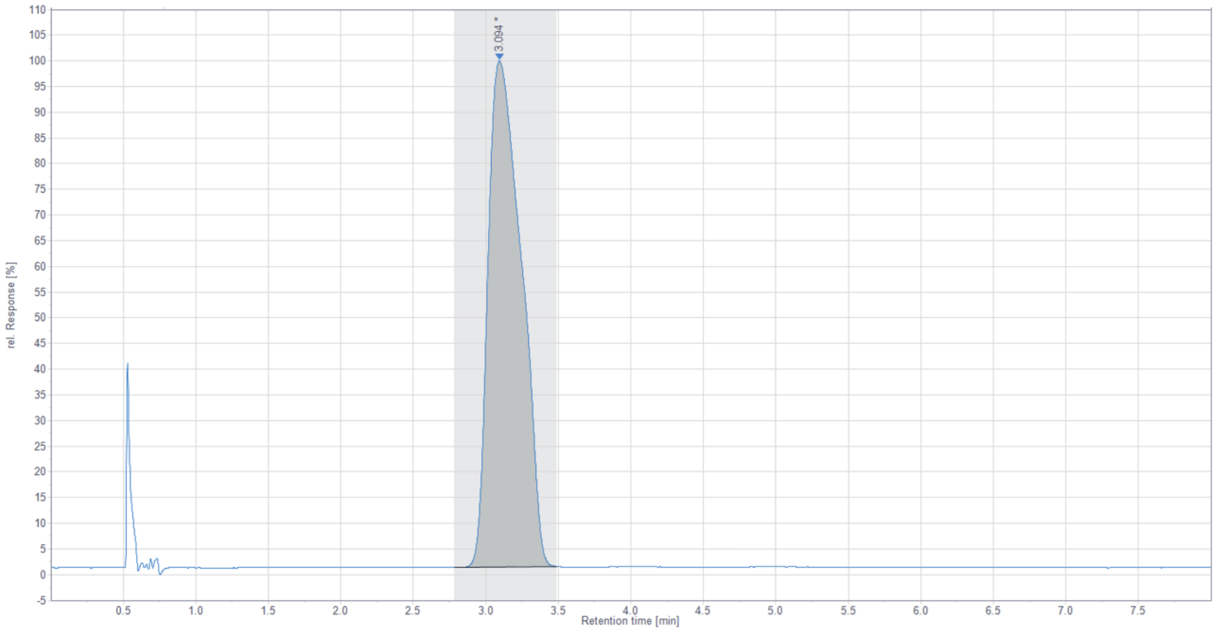

(R)-2-(2,5-dioxopyrrolidin-1-yl-3,3,4,4-d_4)-N-(2-fluorobenzyl)propanamide - **d_4-(R,S)-AS-7**

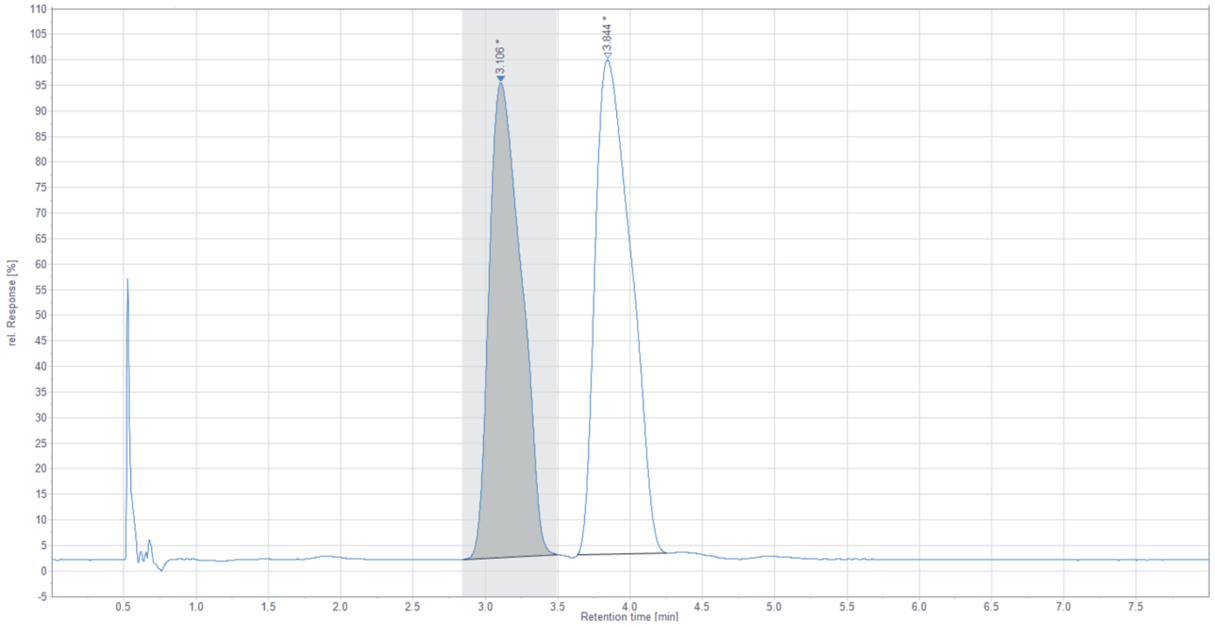

(*R*)-2-(2,5-dioxopyrrolidin-1-yl-3,3,4,4-d$_4$)-*N*-((2-fluorophenyl)methyl-d$_2$)propanamide - **d$_6$-(*R*)-AS-7**

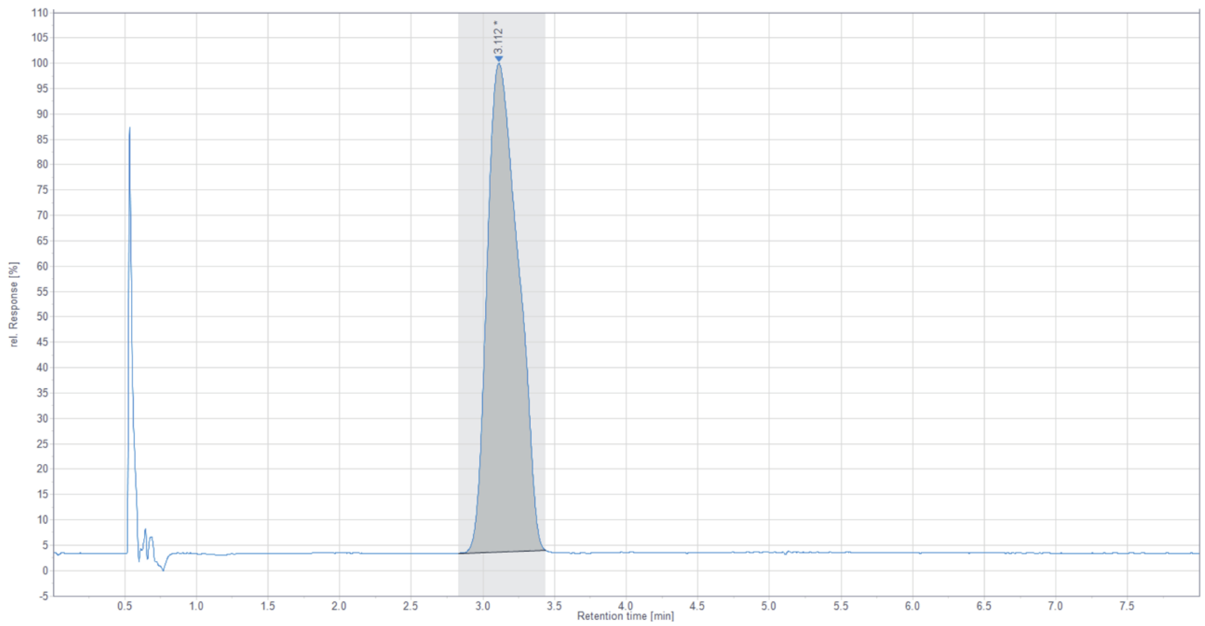

(*R*)-2-(2,5-dioxopyrrolidin-1-yl-3,3,4,4-d$_4$)-*N*-((2-fluorophenyl)methyl-d$_2$)propanamide - **d$_6$-(*R*,*S*) -AS-7**

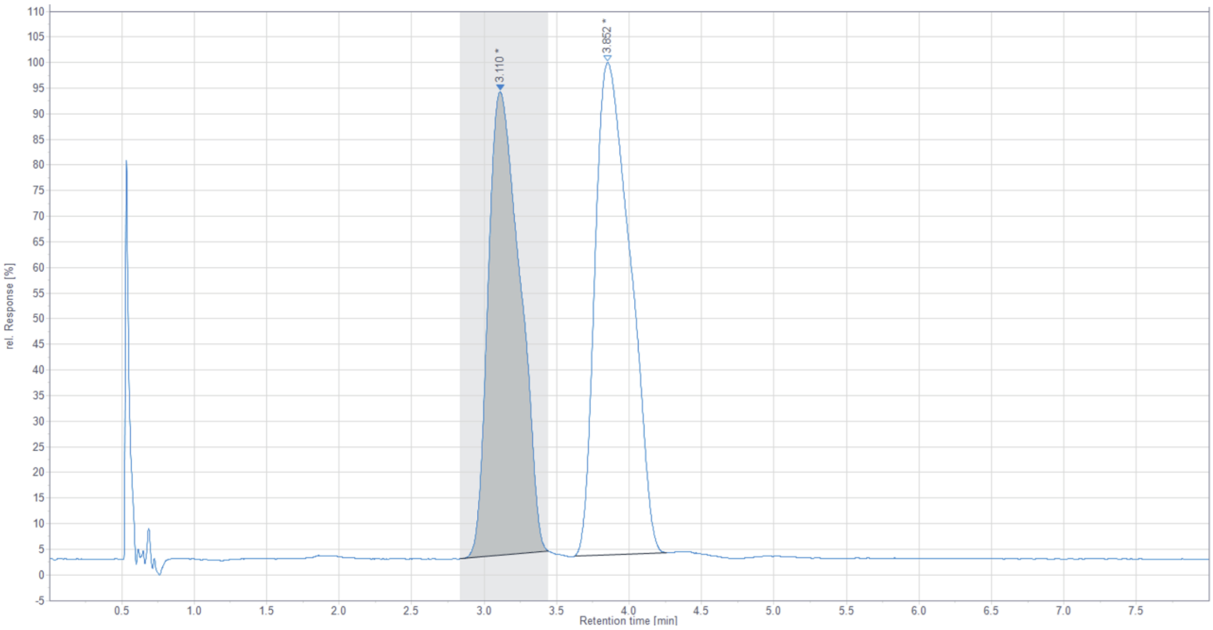

Supplement: Supplementary file 1 [file oc6c00080_si_001.pdf]
